# 5S Ribosomal DNA of Genus *Solanum*: Molecular Organization, Evolution, and Taxonomy

**DOI:** 10.3389/fpls.2022.852406

**Published:** 2022-04-13

**Authors:** Yurij O. Tynkevich, Antonina Y. Shelyfist, Liudmyla V. Kozub, Vera Hemleben, Irina I. Panchuk, Roman A. Volkov

**Affiliations:** ^1^Department of Molecular Genetics and Biotechnology, Yuriy Fedkovych Chernivtsi National University, Chernivtsi, Ukraine; ^2^Center of Plant Molecular Biology (ZMBP), Eberhard Karls University of Tübingen, Tübingen, Germany

**Keywords:** 5S rDNA, genomics, molecular evolution, hybridization, polyploidy, taxonomy, *Solanum*

## Abstract

The *Solanum* genus, being one of the largest among high plants, is distributed worldwide and comprises about 1,200 species. The genus includes numerous agronomically important species such as *Solanum tuberosum* (potato), *Solanum lycopersicum* (tomato), and *Solanum melongena* (eggplant) as well as medical and ornamental plants. The huge *Solanum* genus is a convenient model for research in the field of molecular evolution and structural and functional genomics. Clear knowledge of evolutionary relationships in the *Solanum* genus is required to increase the effectiveness of breeding programs, but the phylogeny of the genus is still not fully understood. The rapidly evolving intergenic spacer region (IGS) of 5S rDNA has been successfully used for inferring interspecific relationships in several groups of angiosperms. Here, combining cloning and sequencing with bioinformatic analysis of genomic data available in the SRA database, we evaluate the molecular organization and diversity of IGS for 184 accessions, representing 137 species of the *Solanum* genus. It was found that the main mechanisms of IGS molecular evolution was step-wise accumulation of single base substitution or short indels, and that long indels and multiple base substitutions, which arose repeatedly during evolution, were mostly not conserved and eliminated. The reason for this negative selection seems to be association between indels/multiple base substitutions and pseudogenization of 5S rDNA. Comparison of IGS sequences allowed us to reconstruct the phylogeny of the *Solanum* genus. The obtained dendrograms are mainly congruent with published data: same major and minor clades were found. However, relationships between these clades and position of some species (*S. cochoae, S. clivorum, S. macrocarpon*, and *S. spirale*) were different from those of previous results and require further clarification. Our results show that 5S IGS represents a convenient molecular marker for phylogenetic studies on the *Solanum* genus. In particular, the simultaneous presence of several structural variants of rDNA in the genome enables the detection of reticular evolution, especially in the largest and economically most important sect. *Petota*. The origin of several polyploid species should be reconsidered.

## Introduction

Regions coding for 5S rRNA (rDNA) are present in genomes of all cellular organisms. In eukaryotes, 5S rDNA belongs to the class of moderately repeated sequences and is represented by hundreds or thousands of copies of tandemly arranged repeated units (repeats). 5S rDNA clusters are mostly located in one or two chromosomes, although multiple loci are also found (Volkov et al., 2004; Hasterok et al., 2006; Garcia et al., 2012; Bustos et al., 2020; Vozárová et al., 2021). In contrast to majority of repeated sequences whose functions largely remain uncertain, the activity of 5S rDNA is vital for cells, providing rRNA indispensable for assembly of functional ribosomes. The copy number of rDNA repeats is higher than what is required for rRNA synthesis, and redundant copies of rDNA are transcriptionally silenced (Volkov et al., 2004; Tucker et al., 2010; Layat et al., 2012; Matyasek et al., 2016).

Each 5S rDNA repeat consists of an evolutionarily conserved region encoding 5S rRNA (coding sequence, CDS) and a rapidly evolving intergenic spacer (IGS) (Volkov et al., 2001; Ishchenko et al., 2018; Simon et al., 2018; Qin et al., 2019). The high evolutionary stability of the CDS is the result of purifying selection to maintain the function of 5S rRNA as a component of a large ribosome subunit (Vizoso et al., 2011; Mahelka et al., 2013).

Transcription of 5S rDNA is provided by RNA polymerase III (Pol III) and corresponding transcription factors (TFs). The Pol III promoter consists of internal and external elements. The internal elements of the promoter, A-Box, IE, and C-Box, are located in the CDS and represent targets for TFs, which are necessary for the recruitment of Pol III to the transcription initiation complex (Douet and Tourmente, 2007; Layat et al., 2012). Respectively, mutations in internal elements of the promoter should not only disturb the structure of 5S rRNA and ribosome but also affect binding of TFs to the promoter and, thus, the expression of 5S rDNA.

In contrast to the CDS, the main part of the IGS is not transcribed and probably does not have any function. Accordingly, it is believed that any mutation in the IGS is selectively neutral; therefore, this region evolves with a high rate. However, it was found that the IGS of *Arabidopsis thaliana* contains short-sequence motifs involved in initiation (external elements of the promoter) and termination (terminator) of 5S rDNA transcription (Douet and Tourmente, 2007; de Souza et al., 2020). Thus, one would expect that these regions are to be relatively conservative, as has been demonstrated in several taxonomic groups (Falistocco et al., 2007; Tynkevich and Volkov, 2014; Tynkevich et al., 2015; Mlinarec et al., 2016; Ishchenko et al., 2018; Alexandrov et al., 2021). However, the existing knowledge of the organization of external promoter elements and their molecular evolution is still incomplete.

In many diploid species, numerous copies of rDNA repeats in the same genome tend to be nearly identical because of sequence homogenization (Volkov et al., 1999b,^2001^, ^2003^), i.e., individual copies of repeated elements do not evolve independently but in a concerted manner (Arnheim et al., 1980; Coen et al., 1982). To explain the high intragenomic similarity of 5S rDNA repeats, the “concerted evolution” and “birth and death” hypotheses were proposed. The mechanisms and intensity of homogenization may differ in different taxa and for different groups of repeated sequences, e.g., for 5S and 35S rDNA (Pinhal et al., 2011; Vizoso et al., 2011; Song et al., 2012; Mahelka et al., 2013; Galián et al., 2014; Barman et al., 2016; Volkov et al., 2007, 2017; Chen et al., 2021).

Additive inheritance of both parent variants of 5S and 35S rDNA is usually observed in the first generation of interspecific hybrids/allopolyploids. However, in ancient allopolyploids, 35S rDNA can be the subject of interlocus sequence conversion (Volkov et al., 1999a,b, 2007; Vizoso et al., 2011), while different variants of 5S rDNA can coexist in a plant genome for a long time without being homogenized (Fulnecek et al., 2002; Song et al., 2012; Mahelka et al., 2013; Mlinarec et al., 2016; Volkov et al., 2017; Ishchenko et al., 2018). Especially, a significant sequence divergence was found for spatially distant 5S rDNA variants that are located in different loci of the same chromosome set (Cronn et al., 1996; Vozárová et al., 2021) and for different parent loci in genomes of hybrid origin (Fulnecek et al., 2002; Matyasek et al., 2002), while repeats from the same locus appeared to be highly homogenized. Accordingly, 5S rDNA became an attractive focus for investigation of molecular evolution of repeated sequences, identification of hybrids, and phylogenetic studies on angiosperms (Blöch et al., 2009; Baum et al., 2012; Simeone et al., 2018; Tynkevich and Volkov, 2019; Ishchenko et al., 2020, 2021; Cardoni et al., 2021; Vozárová et al., 2021). However, 5S rDNA is still poorly characterized in many important plant groups such as the *Solanum* L. genus.

The *Solanum* (nightshade) genus is an attractive model for comparative genomics and investigation of molecular evolution of repeated sequences. With around 1,200 species, it belongs to the so-called “giant genera” and is the fifth largest genus of flowering plants (Frodin, 2004; Echeverría-Londoño et al., 2020). *Solanum* species are distributed worldwide from tropical to temperate areas and grow under diverse ecological conditions. Most *Solanum* species inhabit the New World, although secondary centers of diversity have been found in Africa, Asia, and Australia (D’Arcy, 1991). Overall, the *Solanum* genus is an example of unusual hyperdiversity in life forms, morphological features, and ecological preferences, representing a unique system for studying the diversification of plants (Knapp et al., 2004; Echeverría-Londoño et al., 2020). The genus includes important crops such as *S. tuberosum* L. (potato), *S. lycopersicum* L. (tomato), and *S. melongena* L. (brinjal eggplant, aubergine), about 20 cultivated species of local significance like *S. aethiopicum* L. (Ethiopian eggplant), *S. betaceum* Cav. (tamarillo), *S. muricatum* Aiton (pepino), and *S. quitoense* Lam. (lulo), as well as several medicinal and ornamental plants (*S. marginatum* L.f., *S. aviculare* G. Forst., *S. mammosum* L., and *S. pseudocapsicum* L.).

In the Solanaceae family, the *Solanum* genus belongs to the strongly supported large “x = 12” clade (Olmstead et al., 2008). The most common chromosome number in *Solanum* is x = 12, which occurs in 97% of species examined, such as diploids (77%), tetraploids (14%), hexaploids (4%), triploids (2%), and octoploids (0.2%). Application of *in situ* hybridization showed that 55 out of 64 (85.9%) diploid species possess only one 5S locus per chromosome set (Chiarini et al., 2018). Up to now, the molecular organization and evolution of the 5S rDNA in the genus *Solanum* have only been analyzed in about 35 species and breeding lines (Volkov et al., 2001; Davidjuk et al., 2010; Sun et al., 2014). In this study, combining cloning and sequencing with analysis of available genomic data in the Sequence Read Archive (SRA) public database, we evaluate the molecular organization, diversity, and evolution of the IGS for 184 plant accessions, representing 137 species across the *Solanum* genus. Especially, our results shed a new light on the phylogeny of the genus and reticulate evolution of the largest and economically most important sect. *Petota*.

## Materials and Methods

### Plant Material and DNA Extraction

A plant material of the *Solanum* species was obtained from several collections (see Tables 1, 2). A plant material of out-group species, *Lycianthes lycioides* (L.) Hassl. and *Physalis peruviana* L. (acc. no. NK-03), was obtained from Orto Botanico di Padova (Italy) and National Botanical Garden of National Academy of Sciences of Ukraine (Kyiv, Ukraine), respectively.

**TABLE 1:** List of *Solanum* species analyzed (excluding sect. *Petota*).

Species name	Taxonomy		Chromosome number, 2 n	Abbreviation	Plant material	
	Nee, 1999 (Subgenus-Section)	Särkinen et al., 2013 (Clades)			Accession No	Source
*S. abutiloides* (Griseb.) Bitter and Lillo	Solanum-Brevantherum	Leptostemonum-Brevantherum	24	abu	19682363	MBG
*S. aculeatissimum* Jacq.	Leptostemonum-Acanthophora	Leptostemonum-Acanthophora	24	acu	79p515	WABG
*S. aethiopicum* L.	Leptostemonum-Melongena	Leptostemonum Old World	24	aet	SAMN 10986202	PRJNA 523664
*S. albostellatum* R.W. Davis and P.J.H. Hurter	Not indicated	Not indicated	nd	als	SAMN 10969051	PRJNA 522689
*S. americanum* Mill.	Solanum-Solanum	Morelloid	24	ame1	SAMEA 3486921	PRJEB 9916
				ame2	SAMEA 3486922	PRJEB 9916
				ame3	SAMEA 7573861	PRJEB 38240
*S. anguivi* Lam.	Not indicated	Leptostemonum Old World	24	ang	SAMN 16746499	PRJNA 676007
*S. anomalostemon* S. Knapp and M. Nee	Not indicated	Not indicated	24	ano	SAMEA 7820352	PRJEB 42506
*S. appendiculatum* Dunal	Solanum-Anarrhichomenum	Potato-Anarrhichomenum	24	ape1	SAMN 12623209	PRJNA 561636
				ape2	SAMN 12623212	PRJNA 561636
*S. aviculare* G. Forst.	Solanum-Archaesolanum	Archaesolanum	46	avi	19771009	MBG
*S. betaceum* Cav.	Bassovia-Pachyphylla	Leptostemonum-Cyphomandra	24	bet	–	BGUT
*S. chrysotrichum* Schltdl.	Not indicated	Not indicated	24	chr	SAMN 08770449	PRJNA 438407
*S. clarkiae* Symon	Not indicated	Leptostemonum Old World	24	cla	SAMN 12161630	PRJNA 551615
*S. cleistogamum* Symon	Leptostemonum-Melongena	Leptostemonum Old World	24	cle	SAMN 10969163	PRJNA 522689
*S. clivorum S. Knapp*	Solanum-Holophylla	Not indicated	nd	cli	SAMEA 7820346	PRJEB 42506
*S. cochoae* G.J. Anderson and Bernardello	Solanum-Basarthrum	Not indicated	24	coc	SAMEA 7820347	PRJEB 42506
*S. crinitum* Lam.	Leptostemonum-Crinitum	Leptostemonum-Androceras/Crinitum	24	cri	74s1231	WABG
*S. dimorphandrum* S. Knapp	Not indicated	Not indicated	24	dim	SAMEA 7820348	PRJEB 42506
*S. diversiflorum* F. Muell.	Not indicated	Leptostemonum Old World	24	div	SAMN 10969025	PRJNA 522689
*S. dulcamara* L.	Solanum-Dulcamara	Dulcamaroid	24	dul	96065	BGUT
*S. elatius* A.R. Bean	Not indicated	Not indicated	24	ela	SAMN 10969339	PRJNA 522689
*S. erianthum* D. Don	Solanum-Brevantherum	Not indicated	24	eri	SAMN 08770591	PRJNA 438407
*S. esuriale* Lindl.	Not indicated	Leptostemonum Old World	24, 48	esu	SAMN 10969026	PRJNA 522689
*S. ferocissimum* Lindl.	Leptostemonum-Melongena	Leptostemonum Old World	24, 48	fer	SAMN 10969027	PRJNA 522689
*S. guamense* Merr.	Not indicated	Not indicated	nd	gua	81s39	WABG
*S. hindsianum* Benth.	Leptostemonum-Melongena	Leptostemonum-Elaeagnifolium	24	hin	–	LDZG
*S. horridum* Dunal ex Poir.	Not indicated	Leptostemonum Old World	24	hor	SAMN 10969028	PRJNA 522689
*S. incanum* L.	Not indicated	Leptostemonum Old World	24	inc	SAMN 07303451	PRJNA 392603
*S. laciniatum* Aiton	Solanum-Archaesolanum	Archaesolanum	92	lac	SAMEA 7820351	PRJEB 42506
*S. lasiophyllum* Humb. and Bonpl. ex Dunal	Not indicated	Not indicated	24, 48	las	SAMN 10969030	PRJNA 522689
*S. linnaeanum* Hepper and P.-M. L. Jaeger	Not indicated	Leptostemonum Old World	24	lin	SAMN 13023229	PRJNA 577305
*S. macrocarpon* L.	Leptostemonum-Melongena	Leptostemonum Old World	24	mac	SAMN 16746492	PRJNA 676007
*S. mammosum* L.	Leptostemonum-Acanthophora	Leptostemonum-Acanthophora	22	mam	–	BGUT
*S. medicagineum* A.R. Bean	Not indicated	Not indicated	nd	mdg	SAMN 12096241	PRJNA 533457
*S. melongena* L.	Leptostemonum-Melongena	Leptostemonum Old World	24	mel1	cultivar Black Beauty	VASSMA Ltd.
				mel2	SAMN 13023228	PRJNA 577305
				mel3	SAMN 07303456	PRJNA 392603
*S. muricatum* Aiton	Solanum-Basarthrum	Potato-Basarthrum	24	mur	–	BGUT
*S. nigrum* L.	Solanum-Solanum	Morelloid	24, 48, 72	nig	SAMN 17035829	PRJNA 683719
*S. ossicruentum* Martine and J. Cantley	Not indicated	Not indicated	48	oss	SAMN 12161629	PRJNA 533451
*S. pachyandrum* Bitter	Leptostemonum-Herposolanum	Not indicated	24	pac	SAMEA 7820344	PRJEB 42506
*S. paposanum*Phil.	Not indicated	Potato-Regmandra	24	pap	SAMEA 7820349	PRJEB 42506
*S. phlomoides* A. Cunn. ex Benth.	Not indicated	Leptostemonum Old World	24, 48	phl	SAMN 10969029	PRJNA 522689
*S. pseudocapsicum* L.	Solanum-Holophylla	Leptostemonum-Geminata	24	pse	–	BGChNU
*S. pseudolulo* Heiser	Not indicated	Leptostemonum-Lasiocarpa	24	psl	XX-GZU-88100737	BGUG
*S. quitoense* Lam.	Not indicated	Leptostemonum-Lasiocarpa	24	qui	XX-GZU-00120822	BGUG
*S. scabrum* Mill.	Solanum-Solanum	Morelloid	72	sca	SAMN 08456262	PRJNA 432637
*S. seaforthianum*Andrews	Solanum-Dulcamara	Not indicated	24	sea	74p1254	WABG
*S. sejunctum* Brennan, Martine and Symon		Leptostemonum Old World	24	sej	SAMN 12161632	PRJNA 551616
*S. sisymbriifolium* Lam.	Leptostemonum-Melongena	Leptostemonum-Sisymbriifolium	24	sis	SAMN 16746501	PRJNA 676007
*S. spirale* Roxb.	Not indicated	Not indicated	48	spi	SAMN 08770592	PRJNA 438407
*S. torvum* Sw.	Leptostemonum-Torva	Leptostemonum-Torva	24, 48	trv	SAMN 16746498	PRJNA 676007
*S. valdiviense* Dunal	Not indicated	Unclear	24	val	SAMEA 7820350	PRJEB 42506
*S. vespertilio* ssp. *vespertilio* Aiton	Leptostemonum-Melongena	Leptostemonum Old World	24	ves	–	BGUT
*S. villosum* Mill.	Solanum-Solanum	Morelloid	48	vil	–	BGChNU
*S. wendlandii* Hook.f.	Leptostemonum-Herposolanum	Leptostemonum-Allophyllum and Wendlandii	24	wen	77c37	WABG
*S. wrightii* Benth.	Leptostemonum-Crinitum	Leptostemonum-Androceras/Crinitum	24	wri	SAMN 16746495	PRJNA 676007

*Taxonomy is shown according to Nee (1999) and Särkinen et al. (2013). Chromosome numbers are presented according to the Chromosome Counts Database (CCDB; http://ccdb.tau.ac.il/). Plant material sources: BGChNU, Botanical Garden of the Chernivtsi National University, Ukraine; BGUG, Botanical Garden of the University of Graz, Austria; BGUT, Botanical Garden of the University of Tübingen, Germany; LDZG, Living Desert Zoo and Gardens, California, United States; MBG, Meise Botanical Garden, Belgium; WABG, Waimea Arboretum and Botanical Garden, Hawaii, United States. PRJNA and PRJEB are the BioProject accession numbers in GenBank (https://www.ncbi.nlm.nih.gov/bioproject/).*

**TABLE 2:** List of analyzed *Solanum* species of sect. *Petota*.

Species name	Taxonomy		Chromosome number, 2 n	Plant material		
	Hawkes, 1990, Nee, 1999 (Subsection-Series)	Huang et al., 2019 (Clade)		Abbre-viation	Accession No	Source
*S. abancayense* Ochoa	Potatoe-Tuberosa (ii)	Clade 4 north	24	abn	SAMN 07540430	PRJNA 394943
*S. acaule* Bitter	Potatoe-Acaulia	Not indicated	48	acl	–	CIP
*S. achacachense* Cardenas	Potatoe-Tuberosa (iii)	Clade 4 north	24	ach	SAMN 07540512	PRJNA 394943
*S. acroglossum* Juz.	Potatoe-Piurana	Clade 3	24	acg	SAMN 07540377	PRJNA 394943
*S. acroscopicum* Ochoa	Potatoe-Tuberosa (ii)	Clade 3	24	acs	SAMN 07540369	PRJNA 394943
*S. ahanhuiri* Juz. and Bukasov	Potatoe-Tuberosa (cult.)	Not indicated	24	ajh	SAMN 12684889	PRJNA 556263
*S. albornozii* Correll	Potatoe-Piurana	Clade 3	24	abz	SAMN 07540378	PRJNA 394943
*S. ambosinum* Ochoa	Potatoe-Tuberosa (ii)	Clade 4 north	24	amb1	SAMN 07540480	PRJNA 394943
				amb2	SAMN 07540482	PRJNA 394943
*S. andreanum* Baker	Potatoe-Tuberosa (i)	Clade 3	24, 48	adr	SAMN 07540382	PRJNA 394943
*S. arcanum* Peralta	Estolonifera-Neolycopersicon	Not indicated	24	arc	SAMEA 2335233	PRJEB 5226
*S. avilesii* Hawkes and Hjert.	Potatoe-Tuberosa (iii)	Clade 4 south	24	avl	SAMN 07540476	PRJNA 394943
*S. berthaultii* Hawkes	Potatoe-Tuberosa (iii)	Clade 4 south	24	ber1	BGRC 18548	GDC
				ber2	SAMN 07540477	PRJNA 394943
*S. blanco-galdosii* Ochoa	Potatoe-Piurana	Clade 3	24	blg	SAMN 07540379	PRJNA 394943
*S. boliviense* Dunal	Potatoe-Megistacroloba	Not indicated	24	blv	SAMN 06564709	PRJNA 378971
*S. brevicaule* Bitter	Potatoe-Tuberosa (iii)	Clade 4 south	24, 48, 72	brc	SAMN 07540508	PRJNA 394943
*S. bukasovii* Juz. ex Rybin	Potatoe-Tuberosa (ii)	Clade 4 north	24	buk1	BGRC N 15424	GDC
				buk2	SAMN 07540400	PRJNA 394943
				buk3	SAMN 07540415	PRJNA 394943
				buk4	SAMN 07540419	PRJNA 394943
				buk5	SAMN 07540466	PRJNA 394943
				buk6	SAMN 07540519	PRJNA 394943
				buk7	SAMN 07540520	PRJNA 394943
*S. bukasovii* f. *multidissectum* (Hawkes) Ochoa	Potatoe-Tuberosa (ii)	Clade 4 north	24	bukm1	SAMN 07540390	PRJNA 394943
				bukm2	SAMN 07540456	PRJNA 394943
				bukm3	SAMN 07540457	PRJNA 394943
*S. bulbocastanum* Dunal	Potatoe-Bulbocastana	Clade 1+2	24	blb1	BGRC N 08006	GDC
				blb2	SAMN 07540359	PRJNA 394943
*S. cajamarquense* Ochoa	Potatoe-Tuberosa (ii)	Clade 4 south	24	cjm	SAMN 07540364	PRJNA 394943
*S. canasense* Hawkes	Potatoe-Tuberosa (ii)	Clade 4 north	24	can	SAMN 07540552	PRJNA 394943
*S. candolleanum* P. Berthault	Potatoe-Tuberosa (iii)	Not indicated	24	cnd	SAMN 06564692	PRJNA 378971
*S. cardiophyllum* Lindl.	Potatoe-Pinnatisecta	Clade 1+2	24, 36	cph	SAMN 07540547	PRJNA 394943
*S. chacoense* Bitter	Potatoe-Yungasensa	Clade 4 south	24	chc1	B2	MPI
			24, 36	chc2	SAMN 07540432	PRJNA 394943
*S. chaucha* Juz. and Bukasov	Potatoe-Tuberosa (cult.)	Not indicated	72	cha	SAMN 12684891	PRJNA 556263
*S. cheesmaniae* (L. Riley) Fosberg	Estolonifera-Neolycopersicon	Not indicated	24	che	SAMEA 2340812	PRJEB 5235
*S. chilense* (Dunal) Reiche	Estolonifera-Neolycopersicon	Not indicated	24	chi	SAMEA 2340822	PRJEB 5235
*S. chmielewskii* (C.M. Rick et al.) D.M. Spooner et al.	Estolonifera-Neolycopersicon	Not indicated	24	cml	SAMEA 2340810	PRJEB 5235
*S. chomatophilum* Bitter	Potatoe-Conicibaccata	Clade 3	24	chm	SAMN 07540374	PRJNA 394943
*S. circaeifolium* subsp. *quimense* Hawkes and Hjert.	Potatoe-Circaeifolia	Not indicated	24	crc	BGRC N 27036	GDC
*S. commersonii* Dunal	Potatoe-Commersoniana	Not indicated	24	cmm1	BGRC N 17654	GDC
			24, 36	cmm2	SAMN 06564712	PRJNA 378971
*S. corneliomuelleri* J.F. Macbr.	Estolonifera-Neolycopersicon	Not indicated	24	crm	SAMEA 2340786	PRJEB 5235
*S. curtilobum* Juz. and Bukasov	Potatoe-Tuberosa (cult.)	Not indicated	60	cur	SAMN 12684896	PRJNA 556263
*S. demissum* Lindl.	Potatoe-Demissa	Not indicated	72	dms	–	CIP
*S. ehrenbergii* (Bitter) Rydb.	Potatoe-Pinnatisecta	Not indicated	24	ehr	SAMN 06564745	PRJNA 378971
*S. etuberosum* Lindl.	Estolonifera-Etuberosa	Outgroup	24	etb	SAMN 07540542	PRJNA 394943
*S. galapagense* S.C. Darwin and Peralta	Estolonifera-Neolycopersicon	Not indicated	24	gal	SAMEA 2340846	PRJEB 5235
*S. gourlayi* Hawkes	Potatoe-Tuberosa (iii)	Clade 4 south	24	grl1	5.6	GFP
				grl2	SAMN 07540506	PRJNA 394943
*S. habrochaites* S. Knapp and D.M. Spooner	Estolonifera-Neolycopersicon	Not indicated	24	hab	SAMEA 2340830	PRJEB 5235
*S. hondelmannii* Hawkes and Hjert.	Potatoe-Tuberosa (iii)	Clade 4 south	24	hdm	SAMN 07540500	PRJNA 394943
*S. huaylasense* Peralta	Estolonifera-Neolycopersicon	Not indicated	24	hua	SAMEA 2340821	PRJEB 5235
*S. hypacrarthrum* Bitter	Potatoe-Piurana	Clade 3	24	hcr	SAMN 07540375	PRJNA 394943
*S. incamayoense* K.A. Okada and A.M. Clausen	Potatoe-Tuberosa (iii)	Clade 4 south	24	inm	SAMN 07540492	PRJNA 394943
*S. infundibuliforme* Phil.	Potatoe-Cuneoalata	Not indicated	24	ifd	SAMN 06564699	PRJNA 378971
*S. iopetalum* (Bitter) Hawkes	Potatoe-Demissa	Not indicated	72	iop	GLSK 161	IPK
*S. jamesii* Torr.	Potatoe-Pinnatisecta	Clade 1+2	24	jam1	BGRC N 10054	GDC
				jam2	SAMN 07540363	PRJNA 394943
*S. juzepczukii* Bukasov	Potatoe-Tuberosa (cult.)	Not indicated	36	juz	SAMN 12684892	PRJNA 556263
*S. kurtzianum* Bitter and Wittm.	Potatoe-Tuberosa (iii)	Clade 4 south	24	ktz	SAMN 07540435	PRJNA 394943
*S. laxissimum* Bitter	Potatoe-Conicibaccata	Clade 4 north	24	lxs1	GLKS 154.3	IPK
				lxs2	SAMN 07540550	PRJNA 394943
*S. leptophyes* Bitter	Potatoe-Tuberosa (ii)	Clade 4 north	24	lph	8.27	GFP
*S. limbaniense* Ochoa	Potatoe-Conicibaccata	Clade 4 north	24	lmb	SAMN 07540465	PRJNA 394943
*S. lycopersicoides* Dunal	Estolonifera-Juglandifolia	Not indicated	24	lpd	SAMN 10809628	PRJNA 516877
*S. lycopersicum* L.	Estolonifera-Neolycopersicon	Outgroup	24	lyc1	-	-
				lyc2	SAMN 15097861	PRJNA 637170
				lyc3	SAMN 11163599	PRJNA 527863
*S. lycopersicum* var. *cerasiforme* (Dunal) D.M. Spooner et al.	Estolonifera-Neolycopersicon	Not indicated	24	lycc1 lycc2	SAMN 09229594 SAMN 09229698	PRJNA 454805 PRJNA 454805
*S. maglia* Schltdl.	Potatoe-Maglia	Clade 4 south	36	mag	BGRC N032571	GDC
*S. marinasense* Vargas	Potatoe-Tuberosa (ii)	Clade 4 north	24	mrn	SAMN 07540408	PRJNA 394943
*S. medians* Bitter	Potatoe-Tuberosa (ii)	Clade 4 north	24, 36	med	SAMN 07540469	PRJNA 394943
*S. megistacrolobum* Bitter	Potatoe-Megistacroloba	Clade 4 south	24	mga	SAMN 07540385	PRJNA 394943
*S. microdontum* Bitter	Potatoe-Tuberosa (iii)	Clade 4 south	24	mcd1	BGRC 27351	GDC
			24, 36	mcd2	SAMN 07540501	PRJNA 394943
*S. multiinterruptum* Bitter	Potatoe-Tuberosa (ii)	Clade 3	24	mtp	SAMN 07540388	PRJNA 394943
*S. neorickii* D.M. Spooner, G.J. Anderson and R.K. Jansen	Estolonifera-Neolycopersicon	Not indicated	24	neo	SAMEA 2340816	PRJEB 5235
*S. neorossii* Hawkes and Hjert.	Potatoe-Tuberosa (iii)	Not indicated	24	nrs	11.42	GFP
*S. okadae* Hawkes and Hjert.	Potatoe-Tuberosa (iii)	Not indicated	24	oka1	BGRC 17550	GDC
				oka2	BGRC 24719	GDC
				oka3	SAMN 06564702	PRJNA 378971
*S. palustre* Poepp. ex Schltdl.	Estolonifera-Etuberosa	Outgroup	24	pal1	BGRC N 17441	GDC
				pal2	SAMN 07540543	PRJNA 394943
*S. pampasense* Hawkes	Potatoe-Tuberosa (ii)	Clade 4 north	24	pam	SAMN 07540427	PRJNA 394943
S. *paucissectum* Ochoa	Potatoe-Piurana	Clade 3	24	pcs	SAMN 07540376	PRJNA 394943
*S. pennellii* Correll	Estolonifera-Neolycopersicon	Not indicated	24	pen	SAMN 14984469	PRJNA 557253
*S. peruvianum* L.	Estolonifera-Neolycopersicon	Not indicated	24	per	SAMEA 2340809	PRJEB5235
*S. phureja* Juz. and Bukasov	Potatoe-Tuberosa (cult.)	Cultivated	24	phu1	IVP 101	CPBR
				phu2	SAMN 07540523	PRJNA 394943
*S. pimpinellifolium* L.	Estolonifera-Neolycopersicon	Not indicated	24	pim	SAMN 09229654	PRJNA 454805
*S. pinnatisectum* Dunal	Potatoe-Pinnatisecta	Clade 1+2	24	pnt1	BGRC N 08168	GDC
				pnt2	SAMN 07540354	PRJNA 394943
*S. polyadenium* Greenm.	Potatoe-Polyadenia	Clade 1+2	24	pld1	BGRC N 08176	GDC
				pld2	SAMN 07540357	PRJNA 394943
*S. raphanifolium* Cardenas and Hawkes	Potatoe-Megistacroloba	Not indicated	24	rap1	BGRC N 07207	GDC
				rap2	BGRC N 08189	GDC
				rap3	SAMN 06564696	PRJNA 378971
*S. sitiens* I.M. Johnst.	Estolonifera-Juglandifolia	Not indicated	24	sit	SAMN 14932980	PRJNA 633104
*S. sogarandinum* Ochoa	Potatoe-Megistacroloba	Clade 3	24	sgr1	SAMN 07540395	PRJNA 394943
				sgr2	SAMN 07540416	PRJNA 394943
*S. sparsipilum* (Bitter) Juz. and Bukasov	Potatoe-Tuberosa (ii)	Clade 4 south	24, 48	spl1	14.9	GFP
				spl2	SAMN 07540479	PRJNA 394943
*S. spegazzinii* Bitter	Potatoe-Tuberosa (iii)	Clade 4 south	24	spg1	17.45	GFP
				spg2	SAMN 07540411	PRJNA 394943
*S. stenophyllidium* Bitter	Potatoe-Pinnatisecta	Clade 1+2	24	ste	SAMN 07540355	PRJNA 394943
*S. stenotomum* Juz. and Bukasov	Potatoe-Tuberosa (cult.)	Cultivated	24	stn1	–	CIP
				stn2	SAMN 07540540	PRJNA 394943
*S. stenotomum* subsp. *goniocalyx* (Juz. and Bukasov) Hawkes	Potatoe-Tuberosa (cult.)	Cultivated	24	gon	SAMN 07540541	PRJNA 394943
*S. stoloniferum* Schltdl. and C.D.Bouché	Potatoe-Longipedicellata	Not indicated	48	sto	SAMEA 4949197	PRJEB 28862
*S. tarijense* Hawkes	Potatoe-Yungasensa	Clade 4 south	24	trj	SAMN 07540392	PRJNA 394943
*S. tuberosum* L.	Potatoe-Tuberosa (iii)	Breading lines	24	tbr1	B15	BLBP
			24	tbr2	R1	RAGIS
			24	tbr3	BP1076	Bio
			24	tbr4	B1	BLBP
*S. tuberosum* subsp. andigena (Juz. and Bukasov) Hawkes	Potatoe-Tuberosa (iii)	Not indicated	24, 36, 48	tbrA1	SAMN 06564721	PRJNA 378971
				tbrA2	SAMN 06564717	PRJNA 378971
*S. venturii* Hawkes and Hjert.	Potatoe-Tuberosa (iii)	Clade 4 south	24	vnt	SAMN 07540366	PRJNA 394943
				vrn1	–	GDC
				vrn2	SAMN 07540493	PRJNA 394943
				vrn3	SAMN 07540514	PRJNA 394943
*S. verrucosum* Schltdl.	Potatoe-Tuberosa (i)	Clade 4 south	24, 36, 48	ver	SAMN 07540496	PRJNA 394943
*S. violaceimarmoratum* Bitter	Potatoe-Conicibaccata	Clade 4 north	24	vio	SAMN 07540551	PRJNA 394943

*Taxonomy and species name abbreviations are shown according to Hawkes (1990), Nee (1999), and Huang et al. (2019). Chromosome numbers are presented according to the Chromosome Counts Database (CCDB; http://ccdb.tau.ac.il/). Plant material sources: IPK, the Institut für Pflanzengenetik und Kulturpflanzenforschung, Gatersleben, Germany; GDC, German-Dutch Curatorium for Plant Genetic Resources, Braunschweig, Germany; MPI, Max-Planck-Institute für Züchtungforschung, Köln; GFP, Gesellschaft zur Förderung der Pflanzenzüchtung, Bonn, Germany; CPBR, Center for Plant Breeding and Reproduction Research CPRO, Wageningen, The Netherlands; CIP, Centro Internacional de la Papa, Lima Peru; BLBP, Bayrische Landesanstalt für Bodenkultur und Pflanzenbau, Freising, Germany. PRJNA and PRJEB are the BioProject accession numbers in GenBank (https://www.ncbi.nlm.nih.gov/bioproject/).*

Total genomic DNA was isolated from herbarium specimens according to the CTAB method of DNA extraction (Porebski et al., 1997). In addition, DNA was treated with Proteinase K (Sigma-Aldrich, United States).

### Amplification, Cloning, and Sequencing of 5S rDNA Repeats

Repeated units of 5S rDNA were amplified using the primers Pr5S-L and Pr5S-R, complementary to the 5S rRNA CDS. These primers provide amplification of complete 5S IGS and flanking regions of the CDS (Volkov et al., 2001). PCR amplification was performed as described previously (Tynkevich and Volkov, 2019). PCR products were ligated into plasmid vector pJET 1.2/blunt using CloneJET PCR Cloning Kit (Thermo Fisher Scientific, United States). Screening of recombinant clones and size selection of inserts were performed by colony PCR with pJET 1.2 forward and reverse primers. Two to eight clones per plant accession were Sanger-sequenced by LGC Genomics (Germany). Primary processing of nucleotide sequences and calculation of sequence similarity levels were performed using the Chromas software and the DNASTAR software package. The obtained sequences were deposited in the GenBank database under the accession numbers listed in Table 3.

**TABLE 3:** Characteristics of the 5S intergenic spacer region (IGS) of *Solanum* species analyzed (excluding sect. *Petota*).

Species name	Abbreviation	Clade—Figure 7	Sequencing	TNS	SRA/clone No	GC content, %	IGS length, bp	SIM, %
*S. abutiloides*	abu	2.1	CS	2	OM100771-2	54.7	214	96.7
*S. aculeatissimum*	acu	2.3.1	CS	5	OM100773-7	48.24	189	96.8–100
*S. aethiopicum*	aet	2.3.3D	GA	4	SRX5438534	48.25	165	90.3–98.9
*S. albostellatum*	als	2.3.3B	GA	3	SRX5462807	55.37	193	92.4–94.4
*S. americanum*	ame1	1.1	GA	7	ERX1043111	49.41	225	93.4–98.2
	ame2	1.1	GA	6	ERX1043123	48.8	223	92.5–99.6
	ame3	1.1	GA	2	ERX4706760	49.8	226	99.6
*S. anguivi*	ang	2.3.3D	GA	8	SRX9473543	49.3	189	89.6–99
*S. anomalostemon*	ano	2.1	GA	5	ERX4907182	57.02	215	96.7–99.5
*S. appendiculatum*	ape1	1.4.1	GA	3	SRX6763530	47.57	219	85.5–96.4
	ape2	1.4.1	GA	6	SRX6763552	47.42	220	84.1–99.1
*S. aviculare*	avi	1.3	CS	3	OM100778-80	42.13	208	98.6–99.5
*S. betaceum*	bet	2.2	CS	2	OM100795-6	53.2	187	98.4
*S. chrysotrichum*	chr	2.3.2	GA	3	SRX4043085	49	185	94.1–97.8
*S. clarkiae*	cla	2.3.3A	GA	5	SRX6376308	56.14	199	97.5–99
*S. cleistogamum*	cle	2.3.3C	GA	4	SRX5462725	52.48	174	78.8–95
*S. clivorum*	cli	1.4.1	GA	4	ERX4907176	43.75	209	98.6–99.5
*S. cochoae*	coc	2.2	GA	5	ERX4907177	61.28	156	96.2–99.4
*S. crinitum*	cri	2.3	CS	2	OM100781-2	53	184	97.3
*S. dimorphandrum*	dim	1.2	GA	4	ERX4907178	50.25	167	90.8–99.4
*S. diversiflorum*	div	2.3.3A	GA	4	SRX5462955	56.1	198	97–98.5
*S. dulcamara*	dul	1.2	CS	5	AJ226026-30	57.68	221	96–99.6
*S. elatius*	ela	2.3.3B	GA	4	SRX5462442	53.6	199	93.5–99
*S. erianthum*	eri	2.1	GA	6	SRX4043227	53.18	213	94.8–98.1
*S. esuriale*	esu	2.3.3B	GA	4	SRX5462952	55.9	184	96.2–98.4
*S. ferocissimum*.	fer	2.3.3C	GA	3	SRX5462953	49.73	203	98.5–99
*S. guamense*	gua	2.3.2	CS	4	OM100797-800	48.35	185	93–96.8
*S. hindsianum*.	hin	2.3.3	CS	2	OM100783, OM744710	55.45	258	99
S. *horridum*	hor	2.3.3C	GA	6	SRX5462950	51.38	175	82.5–97.8
*S. incanum*	inc	2.3.3D	GA	5	SRX2977430	50.78	206	96.6–99
*S. laciniatum*	lac	1.3	GA	7	ERX4907181	43.33	206	81.8–99.5
*S. lasiophyllum*	las	2.3.3C	GA	6	SRX5462948	48	169	59.4–98.9
*S. linnaeanum*	lin	2.3.3D	GA	3	SRX6995030	49.03	207	96.2–99.5
*S. macrocarpon*	mac	2.3.3B	GA	6	SRX9473554	46.42	177	91.6–97.8
*S. mammosum*	mam	2.3.1	CS	2	OM100801-2	54.45	203	99.5
*S. medicagineum*	mdg	2.3.3C	GA	7	SRX6095227	50.84	171	97.1–99.4
*S. melongena*	mel1	2.3.3D	CS	3	HM042870-1, OM100803	49.37	198	56–99.6
	mel2	2.3.3D	GA	8	SRX6995029	49.58	338	51.4–99.7
	mel3	2.3.3D	GA	3	SRX2977427	50	349	95.2–99.4
*S. muricatum*	mur	1.4.1	CS	2	OM100804-5	45.5	209	99
*S. nigrum*	nig	1.1	GA	4	SRX9654460	49.68	226	97.8–99.6
*S. ossicruentum*	oss	2.3.3B	GA	6	SRX6376307	52.82	193	85.4–98
*S. pachyandrum*	pac	2.2	GA	6	ERX4907174	48.58	210	83.4–98.6
*S. paposanum*	pap	1	GA	4	ERX4907179	53.23	226	97.8–99.1
*S. phlomoides*	phl	2.3.3A	GA	4	SRX5462951	53.7	179	87.4–98.4
*S. pseudocapsicum*	pse	2.2	CS	3	OM100784-5	63.9	173	95.3–97.1
*S. pseudolulo*	psl	2.3.1	CS	3	OM100806-8	49.87	219	81.2–98.3
*S. quitoense*	qui	2.3.1	CS	4	OM100809-12	49.6	198	71.4–99.6
*S. scabrum*	sca	1.1	GA	4	SRX3641602	46.85	227	92.5–96
*S. seaforthianum*	sea	1	CS	3	OM100813-5	40.47	230	89.6–93
*S. sejunctum*	sej	2.3.3A	GA	4	SRX6376309	53.63	202	97–99
*S. sisymbriifolium*	sis	2.3	GA	2	SRX9473545	54.75	211	99.5
S. *spirale*	spi	2.3.3D	GA	6	SRX4043228	49.03	191	88.7–99
*S. torvum*	trv	2.3.2	GA	5	SRX9473542	49.16	185	95.1–99.5
*S. valdiviense*	val	1.3	GA	3	ERX4907180	51.5	211	98.6–99.5
*S. vespertilio*	ves	2.3.3D	CS	3	OM100816-7	49.17	204	92.2–93.7
*S. villosum*	vil	1.1	CS	2	OM100818-9	48.65	226	96.9
S. *wendlandii*	wen	2.2	CS	8	OM100786-9	48.99	220	58.4–96.9
*S. wrightii*	wri	2.3	GA	1	SRX9473557	53	183	100

*Methods used for generation of sequences: CS, cloning and sequencing; DS, direct sequencing of PCR product; GA, genomic assembly; TNS, total number of 5S IGS sequences (clones or ribotypes) analyzed in this study; SIM, intragenomic similarity between clones/ribotypes. For GC content and length of IGS, average values are shown.*

### Assembly of 5S rDNA Repeats From Illumina Short Reads

*De novo* assembly of 5S rDNA repeats was performed using libraries of pre-filtered paired or single Illumina reads from raw data of *Solanum* species genomes available in SRA (Tables 3, 4). Read filtering was carried out using the built-in tool on the sequence download page: https://trace.ncbi.nlm.nih.gov/Traces/sra/sra.cgi?view=search_seq_name. To filter reads containing 5S rDNA fragments, 20-bp long fragments of CDS were used for matching. *De novo* assembly was conducted using SeqMan NGen 14 (DNASTAR Lasergene suite). Libraries of filtered reads were automatically trimmed for quality, and the following assembly parameters were used: mer size 31, minimum match percentage 100%, and coverage threshold 100 reads. In the obtained contigs with highest coverage from 2 to 12, 5S rDNA repeats that contain one full IGS flanked by two fragments of CDS were identified and collected.

**TABLE 4:** Characteristics of the 5S IGS of *Solanum* species of sect. *Petota*.

Species name	Abbreviation	Cluster—Figure 8	Sequencing	TNS	SRA/clone No	GC content, %	Length, bp	SIM, %
*S. abancayense*	abn	A3	GA	7	SRX4645060	49.09	222	96–99.6
*S. acaule*	acl	D10	CS	4	AJ226031-34	49.43	219	97.3–99.5
*S. achacachense*	ach	D1, D7	GA	5	SRX4645061	51.06	208	87.1–99.1
*S. acroglossum*	acg	A5	GA	7	SRX4645064	51.04	223	96.4–99.6
*S. acroscopicum*	acs	A4	GA	11	SRX4645063	50.76	224	95.6–99.6
*S. ahanhuiri*	ajh	D10	GA	4	SRX6963077	49.2	219	95.9–99.5
*S. albornozii*	abz	A1	GA	5	SRX4645065	46.92	195	94.1–99
*S. ambosinum*	amb1	D1	GA	4	SRX4645068	49.18	207	89.2–98.6
	amb2	D1, D10	GA	12	SRX4645070	49.25	218	94.1–99.5
*S. andreanum*	adr	A4	GA	3	SRX4645073	48.83	214	97.7–98.6
*S. arcanum*	arc	A6	GA	3	ERX376595	45.37	231	97.4–98.3
*S. avilesii*	avl	D1	GA	7	SRX4645077	52.07	203	72.5–99.1
*S. berthaultii*	ber1	D5	CS	5	AJ226037-41	50.9	213	98.1–100
	ber2	D5	GA	7	SRX4645079	49.24	206	73.3–98.6
*S. blanco-galdosii*	blg	A5	GA	5	SRX4645082	50.28	220	96.4–99.5
*S. boliviense*	blv	D6	GA	4	SRX2646030	50.4	214	96.3–98.1
*S. brevicaule*	brc	D6	GA	4	SRX4645091	50.13	211	93.4–99.1
*S. bukasovii*	buk1	A3	DS	1	AF332130	48.2	222	nd
	buk2	A3	GA	4	SRX4645092	48.3	222	98.6–99.5
	buk3	D8, D9	GA	5	SRX4645093	49.02	214	96.7–99.5
	buk4	D8, D9	GA	8	SRX4645094	48.75	208	90.2–99.5
	buk5	D4	GA	5	SRX4645095	50.14	213	98.6–99.5
	buk6	A3, D10	GA	5	SRX4645098	48.58	220	84.7–99.6
	buk7	D6, D8, D10	GA	12	SRX4645099	50.16	214	93.2–99.1
*S. bukasovii* f. *multidissectum*	bukm1	A3, D6	GA	5	SRX4645184	49.94	212	86.9–99.5
	bukm2	D1, D10	GA	5	SRX4645190	49.86	216	94.5–99.5
	bukm3	D7	GA	4	SRX4645191	50.13	214	97.7–99.5
*S. bulbocastanum*	blb1	nd	CS	3	AJ226012-14	50.73	189	98.4–99.5
	blb2	nd	GA	3	SRX4645100	51.93	188	97.9–98.4
*S. cajamarquense*	cjm	D2	GA	4	SRX4645102	50.9	223	96–99.6
*S. canasense*	can	D1, D7	GA	4	SRX4645113	51.83	205	90.6–99.1
*S. candolleanum*	cnd	D3, D6	GA	4	SRX2646047	50.1	213	94.4–98.6
*S. cardiophyllum*	cph	A1	GA	5	SRX4645116	51.86	224	96.4–99.1
*S. chacoense*	chc1	D3	DS	1	AF331055	50.7	213	nd
	chc2	D3	GA	9	SRX4645120	53.16	213	90.1–99.1
*S. chaucha*	cha	A3, D8, D10	GA	10	SRX6966567	49.02	217	83.4–99.6
*S. cheesmaniae*	che	A6	GA	4	ERX384387	46.08	232	97.4–99.1
*S. chilense*	chi	A6	GA	4	ERX384397	46.7	230	97–99.6
*S. chmielewskii*	cml	A6	GA	5	ERX384385	44.95	232	95.3–97
*S. chomatophilum*	chm	A5	GA	5	SRX4645123	51.32	223	96.4–99.6
*S. circaeifolium* subsp. *quimense*	crc	A4	CS	8	AJ226015-22	49.73	227	94.3–100
*S. commersonii*	cmm1	A5	DS	1	AF331056	51.3	224	nd
	cmm2	D3	GA	4	SRX2646027	50.05	220	97.3–99.1
*S. corneliomuelleri*	crm	A6	GA	3	ERX384361	46.87	222	89.5–96.1
*S. curtilobum*	cur	A2, D1, D2, D9, D10	GA	8	SRX6966568	50.09	218	83.3–99.6
*S. demissum*	dms	D10	CS	3	AJ226023-25	49.03	219	98.6–99.5
*S. ehrenbergii*	ehr	A2	GA	5	SRX2645991	50.08	208	83.1–99.1
*S. etuberosum*	etb	A1	GA	7	SRX4645124	48.21	223	94.6–99.1
*S. galapagense*	gal	A6	GA	1	ERX384421	46.4	233	100
*S. gourlayi*	grl1	D6	DS	1	AF331057	51.8	213	nd
	grl2	D6	GA	3	SRX4645138	50.7	214	97.2–99.5
*S. habrochaites*	hab	A6	GA	3	ERX384405	45.63	233	96.6–97.9
*S. hondelmannii*	hdm	D6	GA	5	SRX4645145	50.56	217	90.1–98.3
*S. huaylasense*	hua	A6	GA	1	ERX384396	46.7	229	100
*S. hypacrarthrum*	hcr	A1	GA	5	SRX4645148	45.96	202	89.3–99.5
*S. incamayoense*	inm	D3, D6	GA	4	SRX4645153	49.43	215	90.4–99.1
*S. infundibuliforme*	ifd	D6	GA	4	SRX2646040	49.9	213	97.2–99.5
*S. iopetalum*	iop	D6	CS	4	AJ226042-45	49.0	212	96.2–99.1
*S. jamesii*	jam1	B	DS	1	AF331058	50.2	213	nd
	jam2	B	GA	2	SRX4645155	50.0	212	99.1
*S. juzepczukii*	juz	A2, D2	GA	8	SRX6966566	51.68	221	82.9–99.6
*S. kurtzianum*	ktz	D3	GA	6	SRX4645157	51.28	214	78.2–99.1
*S. laxissimum*	lxs1	C	CS	5	AJ226046-50	49.1	202	97–100
	lxs2	C	GA	4	SRX4645163	49.33	202	94.6–99.5
*S. leptophyes*	lph	D6	DS	1	AF331059	49.8	213	nd
*S. limbaniense*	lmb	D1, D10	GA	8	SRX4645171	50.01	219	91.5–99.6
*S. lycopersicoides*	lpd	A6	GA	2	SRX5301957	45.25	229	92.4
*S. lycopersicum*	lyc1	A6	CS	1	X55697	46	235	nd
	lyc2	A6	GA	5	SRX8467710	45.3	233	93.6–98.7
	lyc3	A6	GA	5	SRX5538725	45.66	233	98.3–99.6
*S. lycopersicum* var. *cerasiforme*	lycc1	A6	GA	4	SRX4183310	46.05	234	97.4–99.6
	lycc2	A6	GA	1	SRX4183171	45.7	234	100
*S. maglia*	mag	D3	CS	8	AF331047-54	47.35	189	72.1–100
*S. marinasense*	mrn	D9, D10	GA	4	SRX4645173	46.35	201	66.7–98.2
*S. medians*	med	A2, D1	GA	11	SRX4645178	49.6	216	73.8–99.6
*S. megistacrolobum*	mga	D2	GA	9	SRX4645179	51.3	213	88.2–99.5
*S. microdontum*	mcd1	D3	CS	9	AJ226051-59	50.27	210	91.2–100
	mcd2	D3, D6	GA	7	SRX4645250	48.43	199	68.8–98.6
*S. multiinterruptum*	mtp	A3	GA	4	SRX4645183	48.63	222	97.3–99.1
*S. neorickii*	neo	A6	GA	5	ERX384391	45.5	230	95.7–98.7
*S. neorossii*	nrs	D3	DS	1	AF331060	50.2	213	nd
*S. okadae*	oka1	D4	CS	3	AJ226060-62	50.33	220	96.4–99.5
	oka2	D1, D4	CS	4	AJ226063-66	51.28	206	72.3–98.6
	oka3	D1	GA	3	SRX2646037	52.57	204	97.1–98
*S. palustre*	pal1	A1	CS	6	AJ226035-63	53.57	174	66.4–100
	pal2	A1	GA	4	SRX4645193	47.95	224	97.3–99.1
*S. pampasense*	pam	D1, D8	GA	5	SRX4645197	50.36	210	85.5–96.7
S. *paucissectum*	pcs	A5	GA	5	SRX4645198	49.26	210	69.1–99.1
*S. pennellii*	pen	A6	GA	4	SRX8371122	47.05	229	96.9–99.1
*S. peruvianum*	per	A6	GA	4	ERX384384	46.88	230	98.3–99.6
*S. phureja*	phu1	D1	DS	1	AF331061	50.0	212	nd
	phu2	D1, D8	GA	6	SRX4645199	49.55	213	96.2–99.5
*S. pimpinellifolium*	pim	A6	GA	4	SRX4183091	46.45	229	98.3–99.1
*S. pinnatisectum*	pnt1	B	CS	5	X82779, AJ226008-11	49.24	210	92.4–96.2
	pnt2	B	GA	18	SRX3115796	50.18	211	83.5–99.5
*S. polyadenium*	pld1	A1	CS	3	AF331044-6	49.97	197	86.9–96.6
	pld2	A1	GA	6	SRX4645210	50.17	204	82.2–99.1
*S. raphanifolium*	rap1	C	DS	1	AF332131	50.0	172	nd
	rap2	A3	DS	2	AF332132-3	50.45	201	73.5
	rap3	C	GA	8	SRX2646043	50.76	176	88.8–98.9
*S. sitiens*	sit	A6	GA	5	SRX8537919	45.4	229	97.4–99.6
*S. sogarandinum*	sgr1	A1	GA	10	SRX4645211	51.15	212	54.9–99.1
	sgr2	A1	GA	6	SRX4645212	45.47	204	83.5–99.6
*S. sparsipilum*	spl1	D6	DS	1	AF331062	49.1	216	nd
	spl2	D6	GA	5	SRX4645216	50.8	206	87.3–98.6
*S. spegazzinii*	spg1	D3	DS	1	AF331063	52.2	205	nd
	spg2	D3	GA	6	SRX4645219	51.28	219	95.4–99.1
*S. stenophyllidium*	ste	A2	GA	3	SRX3115797	50.47	219	98.6–99.5
*S. stenotomum*	stn1	D6	DS	1	AF331064	47.5	200	nd
	stn2	D1, D9	GA	8	SRX4645231	50.64	215	94–99.5
*S. stenotomum* subsp. *goniocalyx*	gon	D8	GA	4	SRX4645128	49.53	213	96.7–98.6
*S. stoloniferum*	sto	D1, D3	GA	6	ERX2825240	50.48	203	86–99.5
*S. tarijense*	trj	D5	GA	7	SRX4645232	51.16	211	92.5–99.1
*S. tuberosum*	tbr1	D3	CS	3	X82780, Y16650-51	52.37	205	99.5–100
	tbr2	D10	CS	1	X82781	49.1	216	nd
	tbr3	D3	CS	4	Y16652-55	52.33	205	99.5–100
	tbr4	D1	CS	4	Y16656-59	49.45	213	92–98.1
	tbrA1	D6, D8, D9	GA	5	SRX2646018	50.12	214	93.5–99.1
	tbrA2	D3, D10	GA	4	SRX2646022	51.1	212	90.4–99.5
*S. venturii*	vnt	D3, D4	GA	8	SRX4645146	49.8	218	93.6–99.5
*S. vernei*	vrn1	D7	DS	1	AF332129	50.0	202	nd
	vrn2	D7	GA	4	SRX4645247	51.45	201	83.1–98.6
	vrn3	D7	GA	9	SRX4645251	51.63	204	70.1–99.1
*S. verrucosum*	ver	D6	GA	7	SRX4645248	49.83	213	93.9–99.1
*S. violaceimarmoratum*	vio	C	GA	4	SRX4645256	48.4	203	91.1–98.5

*Methods used for generation of sequences: CS, cloning and sequencing; DS, direct sequencing of PCR product; GA, genomic assembly; TNS, total number of 5S IGS sequences (clones or ribotypes) analyzed in this study; SIM, intragenomic similarity between clones/ribotypes. For GC content and length of IGS, average values are shown. Sequences generated by cloning for members of sect. Petota were obtained from our previous publication (Volkov et al., 2001).*

### Prediction of 5S rRNA Secondary Structure

Hypothetical secondary structures of potential 5S rRNA transcripts were predicted using the Fold online tool in the RNAstructure server (Reuter and Mathews, 2010).^1^ Lowest free energy structures were calculated using the following default parameters: temperature (in K) 310.15; maximum loop size 30; minimum helix length 3.

### Median-Joining Network and Phylogenetic Analysis

Relationships among IGS sequences of the *Solanum* species were analyzed applying the median-joining network approach implemented in SplitsTree 5 (Huson and Bryant, 2006). Alignments of the IGS sequences were performed in the MAFFT server using the G-INS-I method, which is most suitable for sequences with global homology (Katoh et al., 2019).

For alignment of the IGS sequences of *Solanum* species belonging to different taxonomic groups in the genus, we applied the E-INS-I method implemented in the MAFFT server (Katoh et al., 2019). The generated alignment was checked and adjusted manually with the UGENE software.

The best-fit nucleotide substitution model was estimated with the lowest value of Bayesian Information Criterion (BIC) using the Find Best-Fit Substitution Model tool in Mega X (Kumar et al., 2018). A maximum likelihood (ML) phylogenetic tree was generated with the PhyML plugin for Geneious Prime 2021.0.3.^2^ The IGS sequences of *L. lycioides* and *Ph. peruviana* produced in this study (acc. nos. OM100793-4 and OM744711-3) as well as those of four *Capsicum* species available in GenBank (*C. baccatum* L.: AF217951, *C. frutescens* L.: AF217952, *C. chinense* Jacq.: AF217953, and *C. pubescens* Ruiz and Pav.: AF217954) were used as outgroups. Branch support was calculated by approximate likelihood ratio tests, aLRT-Chi2 (Anisimova and Gascuel, 2006), and bootstrap analysis with 1,000 resampling replicates. Phylogenetic analysis was also performed by Bayesian inference using the MrBayes 2.2.4 plugin for Geneious Prime 2021.0.3. Four independent Monte Carlo Markov Chains (MCMCs) of 1,000,000 iterations each were run to generate phylogenetic trees with Bayesian posterior probabilities. Trees were sampled every 500 generations. The resulting trees were exported in Newick format and annotated using “Interactive tree of life” (iTOL v6).

## Results

### Cloning of 5S rDNA Repeats

5S rDNA repeats of 17 *Solanum* species representing different taxonomic groups were amplified by PCR using primers complementary to the coding region, cloned, and sequenced (Table 3 and Supplementary Material). Analysis of the obtained sequences showed that majority of the clones contained IGS flanked on both sides by fragments of the coding region including the primers used for PCR. Besides, we obtained 5S rDNA clones of *S. vespertilio* and *S. pseudocapsicum* that contain rDNA dimers, i.e., two adjacent copies of IGS, and the whole sequence of the CDS between them. Also, two clones containing 5S rDNA dimers and one clone containing a trimer were sequenced for *S. wendlandii.*

### Intragenomic Diversity of Intergenic Spacer: In-Depth Analysis of Sequence Read Archive Data

**FIGURE 1:**
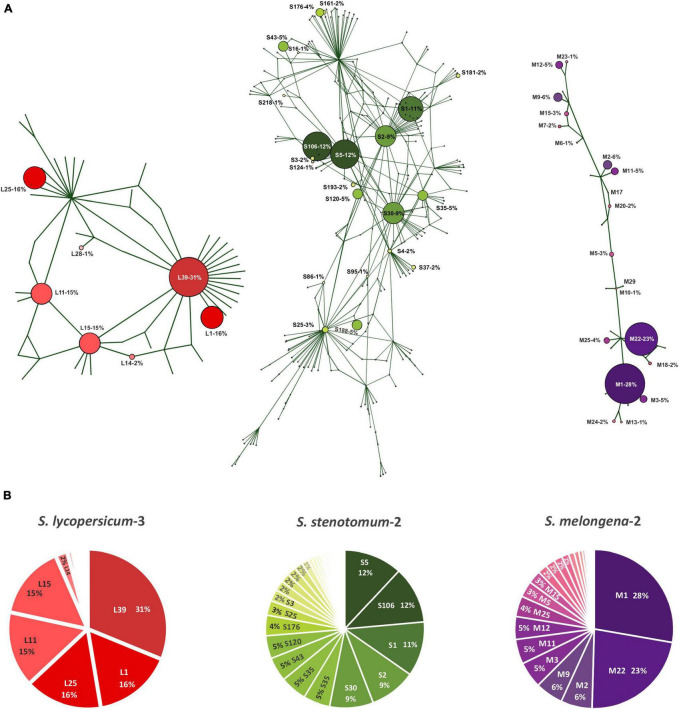
Analysis of 5S rDNA intragenomic diversity in diploid *Solanum* species. **(A)** Median joining networks for IGS types/variants (ribotypes) of *S. lycopersicum-3*, *S. stenotomum-2*, and *S. melongena-2*. Ribotypes are designated by the first letter of corresponding species name with index numbers. The size of the circles is proportional to the relative content (in %) of each ribotype in the genome. **(B)** Relative content (in %) of ribotypes.

In order to assess the intragenomic variability of 5S rDNA, we evaluated how many different types/variants of repeated units (ribotypes) are present in genomes of the *Solanum* species. Genomes of three diploid species, *S. lycopersicum-3* (SRX5538725), *S. stenotomum-2* (SRX4645231) of sect. *Petota*, and *S. melongena-2* (SRX6995029) of sect. *Melongena*, were selected for detailed analysis. For these genomes, we assembled *de novo* 5S rDNA repeats composed of complete IGS and two flanking fragments of CDS. If the CDS contained indels or several SNP, the repeat was considered a pseudogene and excluded from further analysis. Variants of IGS that differed in at least one SNP were considered as distinct ribotypes. The total number of IGS ribotypes was 45, 177, and 31 in *S. lycopersicum-3, S. stenotomum-2*, and *S. melongena-2*, respectively. In order to visualize the intragenomic diversity of the ribotypes found in the three species, median-joining networks were constructed (Figure 1).

After that, we mapped the reads of complete genomic libraries to the reference sequences of all collected ribotypes in order to estimate their relative content in the genomes. The obtained results showed that the IGS ribotypes differ significantly in this parameter. Accordingly, we classified the ribotypes as major (≥10% of all IGS copies present in the genome), minor (<10 but ≥5%), or rare (<5%). The number of major, minor, and rare ribotypes is 5, 0, and 40 in *S. lycopersicum-3*: 3, 5 and 169 in *S. stenotomum-2*; 2, 5, and 24 in *S. melongena-2* (Figure 1). Altogether, the major and minor ribotypes represent 93, 68, and 78% of all rDNA repeats present in the genomes of these three species. Based on the results obtained, in the further analysis of 5S rDNA in other species, we considered only major and minor ribotypes. The variability of IGS sequences in each examined sample is given in Supplementary Material.

### Length and GC Content of the 5S rDNA Repeated Units

Using sequences of clones and major + minor ribotypes, we determined GC content in the IGS of the *Solanum* species (Tables 3, 4) and found that this value ranges from 40.5% in *S. seaforthianum* to 63.9% in *S. pseudocapsicum*. In 90% of the species, intragenomic difference in GC content between individual ribotypes and clones was less than 4%. A greater difference was observed in repeats that were subjected to deletions, particularly in the AT-rich region of the IGS. No significant changes in GC content were found for taxonomic groups in the *Solanum* genus, suggesting that this parameter remained relatively constant during evolution.

The typical length of IGS in members of the *Solanum* genus is about 190–220 bp (Tables 3, 4). The shortest IGS were found in *S. cochoae*, 155–158 bp, and in *S. aethiopicum*, 162–175 bp. In *S. lasiophyllum*, however, one ribotype (las-C2R1) is even shorter, 115 bp, although five other ribotypes in this species are 180 bp in length. The longest IGSs were found in *S. melongena*, 344–360 bp, and in *S. lycopersicum*, 234–235 bp. The extremely long IGS length in *S. melongena* is associated with large duplication of the spacer sequence. There is no significant difference in IGS length among the taxonomic groups in the *Solanum* genus. In general, our data show that the length remained largely unchanged during the evolution of the *Solanum* genus.

### Long Duplication in the Intergenic Spacer of *S. melongena*

Two structural variants of IGS, long (∼350 bp) and short (∼200 bp) were identified in *S. melongena-2* (mel2). The long variant was found in three accessions, mel1 (analyzed by cloning and sequencing) and in mel2 and mel3 (extracted from SRA), while the short variant was only detected in mel1 and mel2. In mel2, all major and minor as well as majority of rare ribotypes belong to the long variant, while the short variant is only represented by two rare ribotypes, M17 and M29 (Figure 1B), whose relative content in the genome is below 1%.

Numerous single nucleotide polymorphisms (SNPs) and two oligonucleotide indels are present in the M29 sequence, so this ribotype appears to be a pseudogene. The ribotype M17 (mel2-C17R1, see Supplementary Material) also contains several SNPs compared to other ribotypes of *S. melongena*. Interestingly, this ribotype is identical to the most common ribotype of a closely related species, *S. linnaeanum*.

**FIGURE 2:**
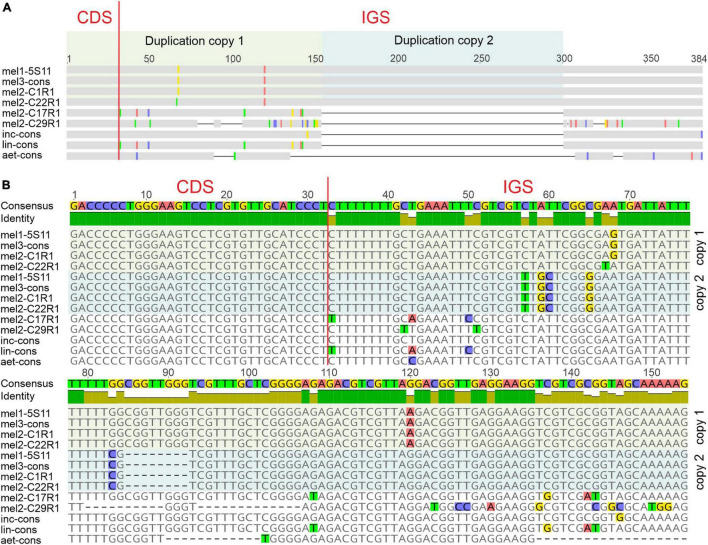
**(A)** Schematic representation of the molecular organization and **(B)** sequence alignment of the 3′ fragment of the coding sequence (CDS) and the complete intergenic spacer region (IGS) the of 5S rDNA of *S. melongena* (mel) and closely related species *S. aethiopicum* (aet), *S. incanum* (inc), *S. linnaeanum* (lin). Two copies of duplication are shown separately and highlighted in different colors. Ribotype and clone names are shown after abbreviations of species names; cons, consensus sequence.

A detailed sequence analysis showed that the long variant contains a 146-bp-long tandem duplication in the central part of the IGS (Figure 2). The duplicated region consists of a 32-bp-long 3′-fragment of the coding region and an adjacent 114-bp fragment of the IGS. Two copies of the duplicated segment differ by 6 SNPs and one 8-bp-long indel. All mutations are localized in the fragment of the IGS, not in the coding region.

### High Diversity of 5S rDNA in *S. wendlandii*

**FIGURE 3:**
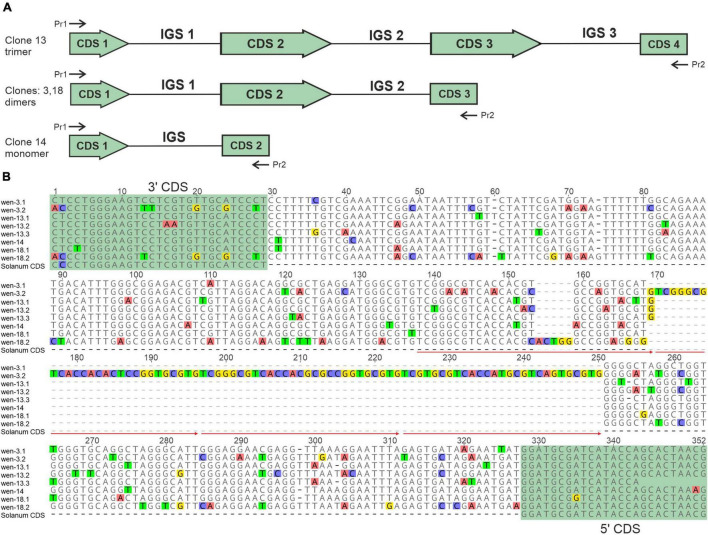
Molecular organization of 5S rDNA repeats in *S. wendlandii* (wen). **(A)** General organization of 5S rDNA clones. Pr1 and Pr2, position of primers Pr5S-L and Pr5S-R used for PCR/cloning. **(B)** Sequences alignment of IGS and flanking fragments of CDS. The consensus sequence of CDS of the genus *Solanum* (Solanum CDS) is shown for comparison. The arrows indicate the location of repeated motifs.

For the 5S rDNA of *S. wendlandii*, we sequenced four clones, pSowen-3,-13,-14, and-18, which bore inserts of different lengths, 732, 912, 319, and 657 bp, respectively. Sequence analysis showed that the shortest insert contains one copy of IGS flanked by CDS fragments. The longer inserts represent two dimers and a trimer composed of adjacent copies of 5S rDNA repeats (Figure 3A).

The sequence alignment revealed an obvious difference among IGS sequences of the adjacent 5S rDNA copies (Figure 3B), which is due to numerous nucleotide substitutions and insertions of different lengths of 1–82 bp. The 82-bp-long insertion harbors three tandem copies of the adjacent sequence, which is normally present once in the IGS. The level of sequence similarity among the compared IGS copies ranges from 58.4 to 96.9%, which indicates high intragenomic heterogeneity of the IGS in *S. wendlandii*.

**FIGURE 4:**
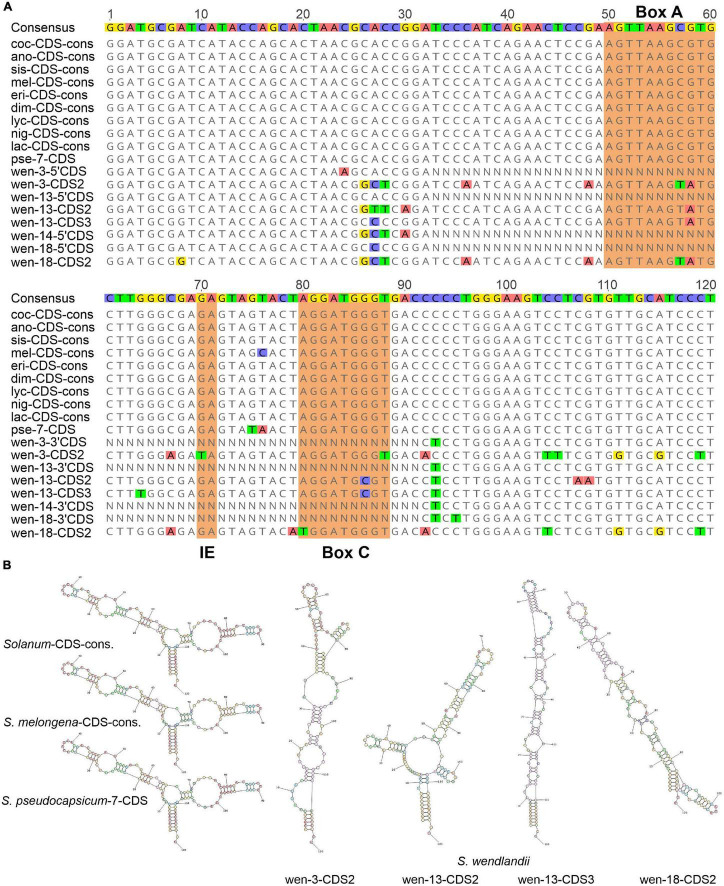
Comparison of *Solanum* 5S rRNA CDS. **(A)** Alignment of the CDS of distantly related *Solanum* species and 5S rDNA clones of *S. wendlandii*. **(B)** Predicted secondary structures of 5S rRNA transcripts. Abbreviations of species names are given in Table 1.

Comparison of the 5S rRNA CDS of *S. wendlandii* and several *Solanum* species representing different intrageneric clades revealed that the CDS is, as expected, highly conserved in the genus. Analysis of the 5S rDNA clones/ribotypes of several *Solanum* species showed that a single CDS usually contains no more than two mutations compared to the respective consensus sequence (data not shown), which agrees well with the observation on other plant taxa (Park et al., 2000; Mahelka et al., 2013). In contrast, the complete CDS sequences of *S. wendlandii* each contain 5–16 base substitutions (Figure 4A).

The presence of numerous mutations in the CDS suggests its transformation into a pseudogene. To test this possibility, we calculated the secondary structure for transcripts of the complete CDS from the clones pSowen-3,-13, and-18. For comparison, the secondary structure was also calculated for (i) the total consensus CDS of the *Solanum* genus, (ii) consensus CDS of *S. melongena*, which differs from the total consensus by one base substitution, and (iii) CDS of *S. pseudocapsicum* (dimer clone pSpse-5S7), which contains two base substitutions (Figure 4B). The sequences examined formed a secondary structure typical for 5S rRNA (Sun and Caetano-Anollés, 2009), with the exception of the CDS of *S. wendlandii*, which appeared to be significantly changed, suggesting that the transcripts cannot fulfill their function in the ribosome.

Hence, the 5S rDNA of *S. wendlandii* appears to be very heterogeneous in both the IGS and CDS regions and likely contains numerous pseudogenes. Unfortunately, the complete genome sequence of *S. wendlandii* is currently not presented in the GeneBank and cannot, therefore, be used to further elucidate the unusual organization of 5S rDNA in this species.

### Intergenic Spacer Organization in Distantly Related *Solanum* Species

**FIGURE 5:**
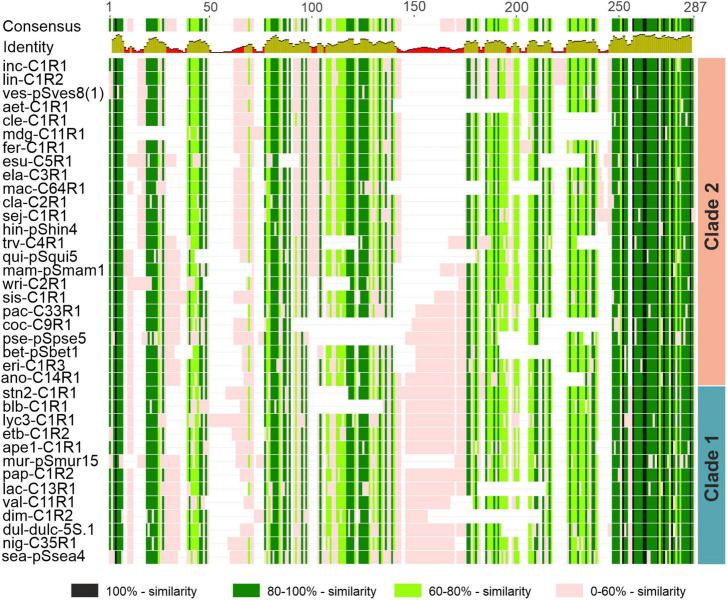
Schematic representation of the sequence alignment of the 5S rDNA IGS of distantly related *Solanum* species. The level of sequence similarity is shown in different colors. Abbreviations of species names are given in Table 1. Taxonomic assignment of species to Clades 1 and 2 in the maximum likelihood (ML) dendrogram (see Figure 7) is shown.

**FIGURE 6:**
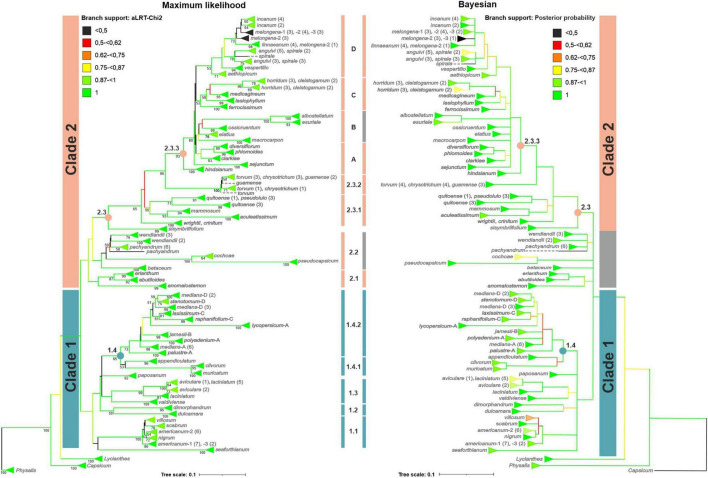
Phylogenetic ML and Bayesian dendrograms constructed by comparison of 5S IGS sequences of *Solanum* species. The aLRT support and posterior probabilities are represented by different branch colors. Numbers in the ML dendrogram represent bootstrap support values. Terminal clades are collapsed to species level. Numbers in brackets near the collapsed nodes indicate the number of ribotypes used. IGS structural variants A-D are given for members of sect. *Petota* (Clade 1.4.2). ML clades not confirmed by Bayesian analysis are highlighted in gray color.

To reveal the molecular organization and evolution of IGS in *Solanum*, we compared the IGS sequences of 37 species representing distantly related groups (D’Arcy, 1991; Nee, 1999; Bohs, 2005; Särkinen et al., 2013) of the genus (Figure 5). The total length of the alignment obtained is 287 bp. Only 9 identical nucleotides were found in the compared sequences, and average pairwise identity value was 55.1%, indicating significant divergence of the IGS in the genus. Multiple base substitutions and indels of various lengths are scattered along the entire IGS in the species studied compared to the consensus sequence. The largest 31-bp-long indel is located in the central part of the IGS between the positions 144 and 174 bp. Despite numerous species-specific mutations, the sequence of the central indel shows an obvious sequence similarity in the species compared. Analysis of the phylogenetic dendrogram obtained by comparing IGS sequences (Figure 6, see also below) revealed that the central indel is present in the species belonging to major clade 1 (with the exception of *S. muricatum*) but is partially or completely absent in members of clade 2 (with the exception of *S. anomalostemon*). Hence, the central indel was present in the common ancestor of the *Solanum* genus and was later lost in some species during the course of evolution.

### Intergenic Spacer Organization in Sect. *Petota*

**FIGURE 7:**
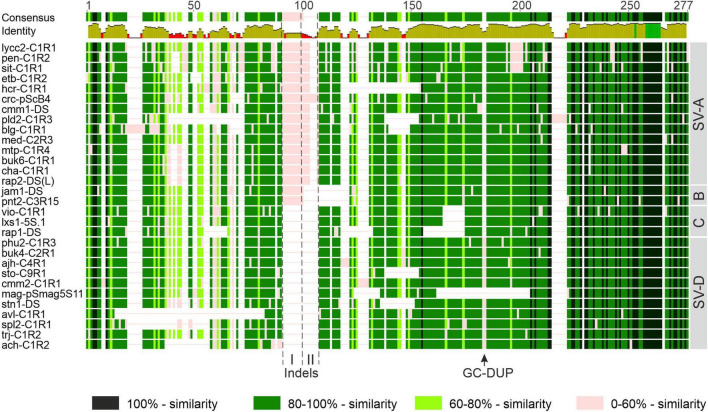
Schematic representation of sequence alignment of the 5S IGS of sect. *Petota* members. The level of sequence similarity is shown in different colors. Positions of group-specific indels I and II and GC-duplication (GC-DUP) are indicated. Abbreviations of species names are given in Table 2. Structural features of presented IGS sequences are indicated as structural variants (SVs) A-D.

Analysis of IGS molecular organization in the species-rich sect. *Petota* was performed separately. By sequence comparison, numerous base substitutions and indels were detected, which appear to be randomly distributed along the IGS (Figure 7), except for the presumptive external promoter region just upstream of the CDS (see section “Discussion”).

The alignment of the sequences revealed that in the central part of the IGS there are two group-specific indels, I and II. Also, a lot of species contain a GC-duplication (GC-DUP) in the IGS (Figure 7). It is likely that these structural rearrangements occurred in different stages during the evolution of sect. *Petota.* With regard to the presence/absence of these molecular features, four structural variants (SVs) of the IGS can be distinguished. The evolutionary ancestral SV-A contains both specific indels, while independent deletions of indels II and I resulted in the formation of the derived SV-B and –C/D, respectively. SV-D additionally contains a GC-DUP.

### Phylogenetic Analysis

The IGS sequences obtained by cloning as well as major and minor ribotypes extracted from SRA were used to reconstruct the phylogenetic relationships among *Solanum* species representing different taxonomic groups of the genus. For sect. *Petota*, seven species were selected whose IGS sequences belong to different structural classes (see section “Discussion”).

Multiple sequence alignment for the whole genus *Solanum* phylogeny was generated with the Mafft E-INS-I method and then manually corrected. The final 609-bp length alignment presented only one identical site, with an average pairwise identity of 54.7%. The best-fit phylogenetic model was estimated using Mega X to be general time-reversible (GTR) + gamma (G) (Kumar et al., 2018). The obtained maximum likelihood (ML) phylogenetic tree has 302 leaves, which correspond to the IGS sequences of 65 *Solanum* species (Figure 6). Calculating the statistical support applying the aLRT-Chi2 method and boot-strap analysis showed that majority of the tree’s nodes have a high or moderate support. The ML tree mostly matched the dendrogram generated by Bayesian inference.

On the dendrograms, all investigated species of the *Solanum* genus form a well-supported monophyletic group with *L. lycioides* as sister taxon. In the ML-tree, the *Solanum* species are divided into two major clades, 1 and 2, with high statistical support. In the clades, several well-supported minor clades were found. The monophyly of the *Solanum* genus and clade 1 is also confirmed by Bayesian inference. In contrast, clade 2 is represented by polytomy in the Bayesian dendrogram.

## Discussion

### Phylogeny of the *Solanum* Genus

Since the nineteenth century, the *Solanum* genus has been traditionally divided into two main groups, the so-called spiny and non-spiny solanums (Dunal, 1852; Seithe, 1962), which were further subdivided into sections, subsections, and series using morphological characters (D’Arcy, 1991; Nee, 1999). However, application of molecular methods shed a new light on the phylogeny of *Solanum*, demonstrating that these groups are mainly not monophyletic, and that the genus can be divided into 13 clades (Bohs, 2005; Weese and Bohs, 2007; Särkinen et al., 2013). Some of these clades have high statistical support, while the taxonomic placement and composition of the others are uncertain.

Analysis of several chloroplast genes and nuclear regions (e.g., ITS1/2 and *waxy*) is often performed in molecular phylogenetics. However, incongruence of results obtained by application of different markers is a well-known problem. Respectively, other genomic regions, particularly the 5S rDNA IGS, can additionally be used to clarify the phylogeny of lower-ranking taxa (Blöch et al., 2009; Tynkevich and Volkov, 2019; Cardoni et al., 2021; Ishchenko et al., 2021), including sect. *Petota* of the *Solanum* genus (Volkov et al., 2001). To evaluate the possibility of using this region to reconstruct phylogenetic relationships in the *Solanum* genus, we constructed an ML dendrogram that embraces 68 accessions from 63 species.

The ML dendrogram includes two major clades (Figure 6). Similar to our data, two clades in the *Solanum* genus were found by comparing sequences of plastid, nuclear ribosomal ITS and low-copy nuclear (*waxy*) genes (Särkinen et al., 2013, 2015). Particularly, four species, *S. abutiloides, S. erianthum, S. cochoa*, and *S. pseudocapsicum*, are included in Clade 2 of our dendrogram, which is in agreement with recent molecular data (Särkinen et al., 2013, 2015) but in contrast to the previous taxonomy of Nee (1999), who placed the species in the sections *Brevantherum, Basarthrum*, and *Holophylla* (see Table 1).

Clade 1 is composed of four smaller clades. Clade 1.1 contains four species of the *Morelloid* clade, *S. americanum, S. nigrum, S. scabrum*, and *S. villosum* (Särkinen et al., 2013, 2015). Two other species, *S. anomalostemon* and *S. valdiviense* previously associated with Morelloids are placed outside Clade 1.1, further supporting the phylogeny of the group proposed by Särkinen et al. (2015).

Clades 1.2–1.4 are combined in a polytomy. *S. dimorphandrum* of the *Thelopodium* clade (Bohs, 2005) and *S. dulcamara* of the *Dulcamaroid* clade (Bohs, 2005; Särkinen et al., 2013) belong to Clade 1.2, while another member of the *Dulcamaroid* clade, *S. seaforthianum*, occupies a basal position in Clade 1. *S. valdiviense* is included in Clade 1.3, which also comprises two species of sect. *Archaesolanum*, *S. aviculare* and *S. laciniatum.* The taxonomic position of *S. valdiviense* found in our analysis is fully consistent with previous data (Särkinen et al., 2015). *S. aviculare* and *S. laciniatum* are closely related (Figure 6): There are several ribotypes in the genome of *S. aviculare* that are very similar and even identical to those of *S. laciniatum*. These data indicate incomplete lineage sorting during speciation or subsequent hybridization among these species. The close relationship between *S. aviculare* and *S. laciniatum* confirms the taxonomy derived from sequencing of three chloroplast and two nuclear regions in which these two species represent sister taxa (Poczai et al., 2011; Särkinen et al., 2015).

Clade 1.4.1 comprises Central American *S. appendiculatum* and South American *S. clivorum*, which were previously assigned, respectively, to sect. *Anarrhichomenum* and *Holophylla* (Nee, 1999) as well as *S. muricatum* of sect. *Basarthrum* (Nee, 1999; Särkinen et al., 2013), while Clade 1.4.2 embraces numerous species of sect. *Petota* (including tomato) (Hawkes, 1990; Nee, 1999; Komarova et al., 2008). According to a recent analysis (Särkinen et al., 2015; Gagnon et al., 2021), *S. appendiculatum* and *S. muricatum*, similar to our results, belong to the potato clade, in contrast to *S. clivorum*, which was placed outside clade I.

Clade 1 also includes the South American species *S. paposanum*, which represents the Regmandra clade (Bohs, 2005; Särkinen et al., 2013). It was found that this clade was resolved in different positions in three data sets used for comparison (Särkinen et al., 2015; Gagnon et al., 2021).

Clade 2 consists of three smaller clades, 2.1–2.3. Clade 2.1 comprises two closely related species, *S. abutiloides* and *S. erianthum*, which were assigned by Nee (1999) to sect. *Brevantherum* of the *Solanum* subgenus. Later, sect. *Brevantherum* was transferred to clade II consisting of predominantly spiny and shrubby species (Särkinen et al., 2013, 2015; Gagnon et al., 2021). Similarly, the third member of Clade 2.1, *S. anomalostemon*, was assigned to the Morelloid clade (Bohs, 2005) but later transferred to clade II (Särkinen et al., 2015; Gagnon et al., 2021). Accordingly, the inclusion of *S. abutiloides, S. erianthum*, and *S. anomalostemon* in clade II is further supported by our results.

Clade 2.2 contains five species, which were previously assigned to different taxonomic groups. According to Nee (1999), two Central/South American species, *S. wendlandii* and *S. pachyandrum*, are members of sect. *Herposolanum*. Later, it was shown that *S. wendlandii* belongs to clade *Wendlandii/Allophyllum*, while the position of *S. pachyandrum* appeared unclear (Bohs, 2005; Särkinen et al., 2013, 2015). Thereafter, both species were assigned to sect. *Aculeigerum* (Clark et al., 2015). Our data also confirm the phylogenetic affinity of *S. wendlandii* and *S. pachyandrum.*

The next two species, South American *S. cochoae* and *S. pseudocapsicum*, have been previously assigned to different sections, *Basarthrum* and *Holophylla* (Anderson and Bernardello, 1991; Nee, 1999). In contrast, *S. cochoae* and *S. pseudocapsicum* are combined in a well-supported clade in our ML dendrogram.

Originally, *S. cochoae* was included in sect. *Basarthrum* on the basis of morphological analyses and crossing experiments, although all crosses with related wild species were unsuccessful. Surprisingly, the only species crossed with *S. cochoae* was cultivated *S. muricatum*, despite large differences in karyotypes of these two species (Anderson and Bernardello, 1991). However, the possibility of obtaining hybrids cannot be seen as a decisive argument for the close relationship between these two species, as it is sometimes possible to successfully cross distant *Solanum* species (Daunay et al., 2019). The close relationship between *S. cochoae* and *S. muricatum* is also supported by recent molecular data (Gagnon et al., 2021). In our dendrogram, however, *S. cochoae* does not appear to be related to *S. muricatum* but to *S. pseudocapsicum*, a member of the Geminata clade (Gagnon et al., 2021).

It should be noted that the common feature of the 5S rDNA repeats of *S. cochoae* and *S. pseudocapsicum* is short length due to deletion in the central part of the IGS. In addition, each species possesses specific deletions in other IGS regions (Figure 5). Altogether, these structural features can affect the position of the species in the dendrogram. Accordingly, we believe that the taxonomic position of *S. cochoae* close to *S. pseudocapsicum* should be interpreted with appropriate reservation in this stage, and that further studies should be carried out in order to finally clarify the question.

The last member of Clade 2.2 is *S. betaceum*, which has been previously treated as a member of separate genus *Cyphomandra* (D’Arcy, 1991) and then later placed to *Solanum* (Bohs, 1995) and assigned to sect. *Pachyphylla* of the *Bassovia* subgenus (Nee, 1999) or clade *Cyphomandra* in clade II (Särkinen et al., 2013, 2015; Gagnon et al., 2021). In our dendrogram, *S. betaceum* is a sister taxon for the other members of Clade 2.2.

Clade 2.3 includes three clades of lower ranks, 2.3.1–2.3.3. Clade 2.3.1 comprises two pairs of species, the South American *S. aculeatissimum* and *S. mammosum* of the section/clade *Acanthophora* as well as Andean cultivated species *S. quitoense* (naranjilla or lulo) and its wild relative *S. pseudolulo* of clade *Lasiocarpa* (Nee, 1999; Bohs, 2005; Levin et al., 2006; Särkinen et al., 2013). According to a molecular analysis, the clades *Acanthophora* and *Lasiocarpa* represent sister taxa (Särkinen et al., 2013; Gagnon et al., 2021). In the genome of *S. quitoense*, a ribotype similar to that of *S. pseudolulo* was detected, which could be due to hybridization between these species (Fory Sánchez et al., 2010).

Clade 2.3.2 comprises two Central/South American species, *S. chrysotrichum* and *S. torvum* of the section/clade *Torva*, as well as *S. guamense*, an endangered endemic species in Northern Mariana Islands (Stone, 1970) whose taxonomic status remains unclear (Nee, 1999; Bohs, 2005; Särkinen et al., 2013; Aubriot et al., 2016). Our analysis revealed that the three species share common ribotypes and are, therefore, unresolved in the dendrogram. The high genetic affinity of *S. chrysotrichum* and *S. torvum* agrees well with their morphological similarity. *S. guamense* also appeared to be closely related to these species.

Clade 2.3.3 includes *S. hindsianum* (clade Elaeagnifolium, Bohs, 2005; Särkinen et al., 2013), an endemic to the Sonoran Desert region of southern Arizona and northern Mexico (Knapp et al., 2017), and a well-supported monophyletic clade of 21 species, most of which have been assigned to sect. *Melongena* (Nee, 1999) or the Old World clade (Levin et al., 2006; Särkinen et al., 2013; Aubriot et al., 2016) of the *Leptostemonum* subgenus. However, the taxonomic position of five species (*S. albostellatum, S. elatius, S. lasiophyllum, S. medicagineum*, and *S. spirale*) has not yet been clarified, especially with molecular methods. In the Old World clade, there are four groups, A–D, of closely related species.

Clade 2.3.3A comprises members of “Dioicum Complex,” a set of several dioecious species (Whalen, 1984; Bean, 2004) from tropical Australia. Our data show that *S. diversiflorum* and *S. phlomoides* are closely related, and that *S. clarkiae* is a more distant species. *S. sejunctum* is placed outside clade 2.3.3A. This result agrees with the phylogeny based on the analysis of *trnK*–*matK* and ITS data sets (Martine et al., 2006, 2009).

Four other Australian species, *S. albostellatum, S. esuriale, S. elatius, S. ossicruentum*, as well as *S. macrocarpon* (African eggplant), belong to the next clade, 2.3.3B, although with a moderate statistical support. The West African species *S. macrocarpon* was previously assigned to Anguivi Grade (Aubriot et al., 2016, 2018; Gagnon et al., 2021), a group of Old World *Leptostemonum* species closely related to *S. melongena* (see our clade 2.3.3D below). Respectively, the phylogenetic affinity of *S. macrocarpon* to the Australian species seems somewhat unexpected and can be explained by the presumptive hybrid origin of *S. macrocarpon* (Daunay et al., 2019).

According to our data, *S. albostellatum* and *S. esuriale* from Western Australia show the closest relationship in Clade 2.3.3B, which is in good agreement with the high morphological similarity of these species (Davis and Hurter, 2012). *S. elatius* is also a member of the *S. esuriale* group (Bean, 2013).

*S. ossicruentum* represents a functionally dioecious bush tomato from northwestern Australia. Earlier, it was recognized as a variant of *S. dioicum*, a member of “Dioicum Complex.” However, later molecular analysis shows that *S. ossicruentum* is either a sister taxon to the rest of this group or represents an independent dioecious lineage (Martine et al., 2016). Our data further supported the second opinion and indicate a phylogenetic affinity between *S. ossicruentum* and members of the *S. esuriale* group.

Clade 2.3.3C comprises five Australian species. Two species, *S. cleistogamum* and *S. horridum*, contain very similar sets of ribotypes in their genomes and appear unresolved in the dendrogram. A sister taxon to them is *S. medicagineum*, while *S. lasiophyllum* and *S. ferocissimum* are more distantly related species. A close relationship among *S. cleistogamum, S. horridum*, and *S. medicagineum* has been shown earlier (Bean, 2004, 2012; Levin et al., 2006).

Hence, the Australian *Solanum* species studied here belong to three clades, 2.3.3A, B, and C. Similarly, monophyly of the Australian species was not supported by the analysis of seven nuclear genes (Martine et al., 2019).

Clade 2.3.3D comprises seven species naturally distributed in Africa and Asia. In particular, this clade includes two very morphologically and genetically similar domesticated plants, *S. aethiopicum* (bitter tomato, Ethiopian eggplant) and *S. melongena* as well as their presumptive wild ancestors, *S. anguivi* and *S. incanum*. The second species is very similar and can even be confused with *S. linnaeanum* (Daunay et al., 2001; Doganlar et al., 2002; Prohens et al., 2012). *S. vespertilio*, a species endemic to the Canary Islands, appears to be closely related to the other members of clade 2.3.3D.

Previously, the phylogeny of Old World “spiny solanums” was clarified using plastid and nuclear markers (Aubriot et al., 2016, 2018; Vorontsova and Knapp, 2016; Knapp et al., 2019; Gagnon et al., 2021). It was demonstrated that *S. incanum, S. linnaeanum*, and *S. melongena* are closely related and belong to the Eggplant clade, and that *S. aethiopicum*, *S. anguivi, S. vespertilio* (and *S. macrocarpon*, which is placed to clade 2.3.3B in our dendrogram) are included in Anguivi Grade outside the Eggplant clade. Hence, our novel data mainly confirm these results.

Surprisingly, *S. anguivi* and morphologically different *S. spirale*, a tetraploid (Randell and Symon, 1976) species from East Asia, are not resolved in the dendrogram (see Figure 6). A possible explanation for this result could be the allopolyploid origin of *S. spirale*. In this case, the 5S rDNA inherited from the parent related to *S. anguivi* could be retained in the genome, while the DNA of the other parent was lost. The uniparental inheritance of 5S rDNA in allopolyploids, both young and old, has been reported for several taxonomic groups including Solanaceae (Pontes et al., 2004; Volkov et al., 2017).

Clade 2.3 also comprises three species that do not belong to clades 2.3.1–2.3.3 presented above. Two species, *S. crinitum* and *S. wrightii*, represent the clade Androceras/Crinitum, while *S. sisymbriifolium* belongs to the clade Sisymbriifolium (Levin et al., 2006; Särkinen et al., 2013; Gagnon et al., 2021). The taxonomic position of these species in our dendrogram agrees well with previous results of molecular phylogenetics studies.

Majority of the clades identified in the ML tree was also recognized in the Bayesian dendrogram (Figure 6). However, the monophyly of Clade 2 was not confirmed by Bayesian inference: Clades 2.1, 2.2, and 2.3 are not combined with each other but belong to a basal polytomy in the *Solanum* genus.

Thus, in this study, we present the phylogeny of the *Solanum* genus derived from the analysis of 5S IGS sequences. Six species (*S. albostellatum, S. elatius, S. guamense, S. lasiophyllum, S. medicagineum*, and *S. spirale*) were characterized here for the first time using molecular taxonomy methods. The obtained dendrograms are mainly congruent with the published data for other regions of nuclear and plastid genomes: same major and minor clades were found for the species examined. However, taxonomic relationships between these clades and position of some species (e.g., *S. cochoae, S. clivorum, S. macrocarpon, S. spirale*) differ from previous results and require further clarification. Taken together, our results show that the 5S IGS represents a convenient molecular marker for phylogenetic studies on the *Solanum* genus. In particular, the simultaneous presence of several variants of rDNA in the genome enables the detection of cases of reticular evolution such as incomplete lineage sorting and interspecific hybridization.

### Molecular Evolution and IGS Diversity in Sect. *Petota*

One of the species-rich groups in genus *Solanum* is sect. *Petota*, which has about 250 members (Hawkes, 1990; Nee, 1999). In our ML dendrogram (Figure 6), sect. *Petota* belongs to Clade 1.4.2.

To analyze the molecular evolution of 5S rDNA in this section and in more details, we assembled IGS ribotypes for 125 accessions representing 83 species (Table 4) and compared the results with our previous data, obtained by cloning and sequencing of 5S rDNA of 32 wild species and breeding lines of sect. *Petota* (Volkov et al., 2001).

Analysis of the IGS sequences revealed that they differ in base substitutions and indels (Figure 7). Same indels mostly occur in a single or some closely related species and, therefore, represent convenient molecular markers for their identification. For example, non-tuber-bearing species *S. etuberosum* and *S. palustre* (series *Etuberosa*; Hawkes, 1990) possess a common specific deletion at the beginning of the IGS, or *S. laxissimum* and *S. violaceimarmoratum* (series *Conicibaccata*) have a deletion in the central part of it (Figure 7). Several species-specific indels in the IGS of the *Solanum* species have already been described (Volkov et al., 2001), and our actual analysis additionally identifies new ones for the novel species. This finding further confirms our earlier assumption that indels are a characteristic feature of IGS evolution in sect. *Petota*. We have also argued that because of the high frequency of indels compared to base substitutions, IGS cannot be used for phylogenetic reconstruction of this section applying standard algorithms. However, the indels represent unique evolutionary events that should be considered in taxonomic studies.

Considering the location of group-specific indels I and II as well as GC-duplication (Figure 7), four major structural variants of the IGS were identified. Accordingly, members of sect. *Petota* can be divided into four groups, A–D.

Group A comprises species that belong to the subsection *Estolonifera* including the tomato group, and to the series *Pinnatisecta, Polyadenia, Commersoniana, Circaeifolia, Megistacroloba, Conicibaccata*, and *Piurana* of the subsection Potatoe (Hawkes, 1990; Nee, 1999), or to clades 1+2 and 3 (Spooner et al., 2014; Huang et al., 2019). Also, SV-A was found in four species (*S. acroscopicum*, *S. andreanum, S. abancayense, S. multiinterruptum*) that were assigned to ser. *Tuberosa* (Hawkes, 1990; Nee, 1999) or clade 4 (Spooner et al., 2014; Huang et al., 2019). Group B includes only two species, *S. jamesii* and *S. pinnatisectum* of ser. *Pinnatisecta* (Hawkes, 1990; Nee, 1999) or clade 1+2 (Huang et al., 2019). This means that the SR-B arose relatively recently during speciation in clade 1+2, just before the divergence of *S. jamesii* and *S. pinnatisectum* but after their separation from the sister taxon, which was similar to *S. stenophyllidium*.

Group C includes accessions of three species, *S. raphanifolium, S. laxissimum*, and *S. violaceimarmoratum*, which belong to the series *Megistacroloba* and *Conicibaccata* (Hawkes, 1990) or clade 4 (Spooner et al., 2014; Huang et al., 2019). Group D embraces numerous species that belong to the series *Yungasensa, Megistacroloba, Cuneoalata, Maglia*, and *Tuberosa* (Hawkes, 1990) or clade 4 (Spooner et al., 2014; Huang et al., 2019). SV-C and D were not found outside of clade 4, which, however, also includes four species possessing SV-A. Therefore, SV-C and D arose from SV-A after separation of clades 3 and 4.

Interestingly, in some cases, rDNA repeats representing different structural variants were found in the same plant accession (see below).

We have also found that the central part of the IGS is completely deleted in *S. bulbocastanum* of the series *Bulbocastana* (Hawkes, 1990) or clade 1+2 (Huang et al., 2019). Accordingly, the structural organization of ITS characteristic of this species cannot be assessed and used for phylogenetic reconstruction.

**FIGURE 8:**
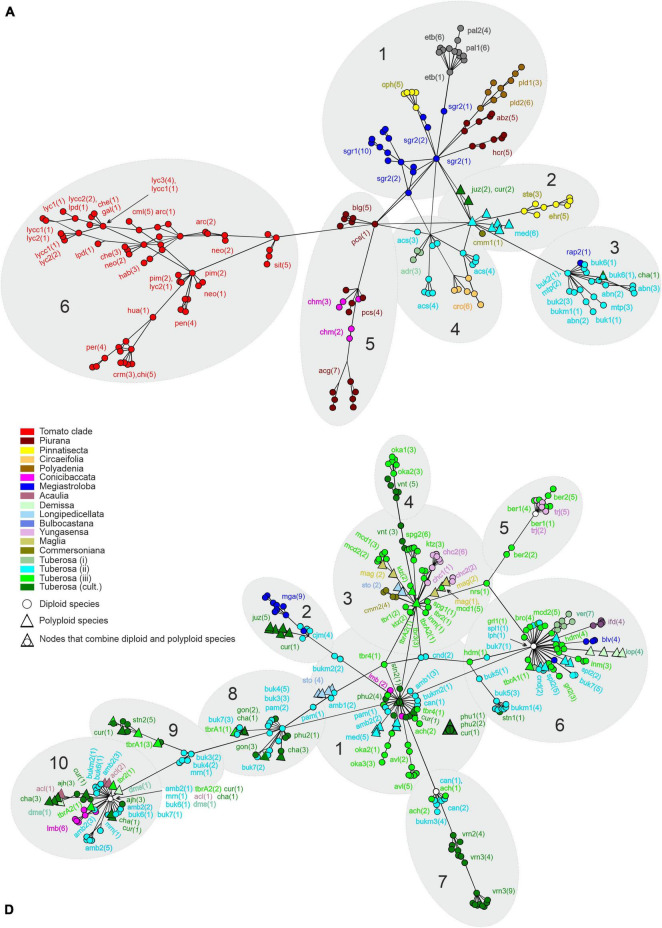
Median-joining networks depicting relationships of 5S IGS structural variants A and D of the species of sect. *Petota*. Names of main clusters are given. The affiliation of the species to the series proposed by Hawkes (1990) is indicated by different colors. Numbers in brackets indicate the number of ribotypes used. Abbreviations of species names are given in Table 2.

Analysis of our data showed that the most common IGS variants are SV-A and-D, and SV-B and-C were found only in four and three species, respectively. In order to assess the molecular diversity of SV-A and-D, we constructed median-joining networks for these two IGS variants using 204 and 353 sequences (Figure 8). In the median-joining networks, the sequences of SV-A and-D are distributed between the six and ten main clusters according to their similarity. In the vast majority of cases, each node corresponds to only one sequence, with the exception of one node in median-joining network A and seven nodes in median-joining network D. These nodes include two to nine ribotypes that mainly represent genomes of different species. Therefore, identical IGS sequence variants can be present in genomes of different species or plant accessions, suggesting their common origin.

SV-D sequences are distributed among ten clusters, D1-D10 (Figure 8). The largest clusters, D1, D3, D6, and D10, contain 50, 56, 68, and 51 sequences, respectively. The sequences included in cluster D1 are nearly identical to SV-D consensus sequence and, therefore, represent evolutionary ancestral ribotypes, while the other clusters comprise derived sequences containing specific base substitution and indels. Starting from cluster D1, five evolutionary lineages can be distinguished.

Taken together, our data agree well with modern taxonomy, which is based on the application of molecular methods (Spooner et al., 2014; Huang et al., 2019) but are less consistent with the traditional classification of Hawkes (1990). In particular, the sections proposed by Hawkes (1990) are not confirmed, because species from different sections are mixed up and belong to different clusters in the median-joining network. In contrast, our results agree well with the molecular data, since clades 4 North and 4 South (Spooner et al., 2014; Huang et al., 2019) can also be recognized in our median-joining network: members of the North and South clades belong to clusters D1, D7-D10, and D2-D6.

### Conserved Sequence Motifs in the Intergenic Spacer of *Solanum* Species

Comparative sequence analysis revealed that the most conservative regions of the IGS in *Solanum* species are the 7- and 40-bp-long fragments at the 5′ and 3′ ends (Figure 5). The evolutionary conservation of these regions has already been observed in other plants (Hemleben and Werts, 1988; Crisp et al., 1999; Tynkevich and Volkov, 2019; Ishchenko et al., 2020, 2021), and a possible reason for this seems to be their involvement in the transcription of 5S rDNA by RNA polymerase III (Pol III).

External elements of the Pol III promoter have been previously characterized in *Arabidopsis thaliana* (Douet and Tourmente, 2007; Vaillant et al., 2007; Layat et al., 2012; Simon et al., 2018). These signals include the TATATA motif (so-called TATA-box), GC-dinucleotide, and C nucleotide in positions-28,-13, and-1 bp, respectively. Similar sequences were also found in other plants (Tynkevich and Volkov, 2014; Tynkevich et al., 2015; Ishchenko et al., 2018, 2021). In representatives of the *Solanum* genus, as well as in *Quercus* (Tynkevich and Volkov, 2019) and *Rosa* (Tynkevich and Volkov, 2014), the TATA-box has a length of 7 bp and begins in position-30. Its sequence (TTTAATA) in *Solanum* is slightly different from that in other groups of plants.

Another external element of the Pol III promoter, the GC-dinucleotide (Douet and Tourmente, 2007) is duplicated in several *Solanum* species and is located, respectively, both in the typical position-12 and, additionally, in position-14. Similar to *Solanum*, duplication of this presumptive external promoter element was also found in the *Quercus* species (Tynkevich and Volkov, 2019).

The third conservative promoter element, cytosine, in position-1 (Douet and Tourmente, 2007; Simon et al., 2018), has been replaced by thymine in more than half of the *Solanum* species. In addition, we found that the dinucleotide GA in position-3 in the IGS is highly conserved, indicating its possible involvement in transcription initiation.

At the beginning of the IGS in *Solanum*, like in other genera, the oligo-T motif TTTTT was found, which probably represents a transcription termination site (Hemleben and Werts, 1988; Simon et al., 2018; de Souza et al., 2020).

The most variable central region of the IGS can be subdivided into (i) AT-rich and (ii) subrepeated regions. Previously, AT-rich regions were found in the IGS of Fabaceae (Hemleben and Werts, 1988) and Poaceae (Röser et al., 2001). AT-rich regions demonstrate a similarity to amplification-promoting sequences (Borisjuk et al., 2000), which may be involved in amplification of 5S rDNA repeats. Also, regions composed of subrepeats were described for the IGS of several plant taxa, e.g., Rosaceae (Tynkevich and Volkov, 2014) and Poaceae (Ishchenko et al., 2018, 2021). Previously, we have demonstrated that structural rearrangements of the variable central region of the IGS in *Solanum* species of sect. *Petota* as well as in distantly related *S. melongena* and *S. betaceum* are preferentially associated with four classes (A–D) of short direct subrepeats: the IGS evolved mainly by duplications of some sequence motifs, resulting in formation of several variants of subrepeats, which were independently amplified in different sections of the genus after radiation from a common ancestor (Volkov et al., 2001; Davidjuk et al., 2010, 2013).

### Intragenomic Heterogeneity and Molecular Evolution of 5S rDNA

It is widely believed that 5S rDNA repeats present in the same genome (at least in diploid species) should be nearly identical because of concerted evolution (Coen et al., 1982; Tynkevich and Volkov, 2014; Barman et al., 2016). In our study, we performed a detailed analysis of 5S rDNA intragenomic sequence diversity and found several ribotypes in all the species studied. Comparative analysis of all available sequences showed that the IGS sequence similarity in *Solanum* species ranges from 51.4 to 100%. The highest levels were found in *S. wrightii* and four representatives of the tomato group, namely *lycopersicum var. cerasiforme-2*, *S. galapagense*, and *S. huaylasense*; each of which had only one ribotype detected. The high intragenomic homogeneity of IGS (over 95%) is also characteristic of other representatives of the tomato group with the exception of *S. lycopersicum-2* and *S. corneliomuelleri.* The relatively low IGS similarity in *S. corneliomuelleri* (89.5%) is due to the presence of 10-bp deletion in one ribotype, while no further indels are found in any of the other members of the tomato group. Hence, deletions are very rare during the evolution of IGS in the tomato group, which is in obvious contrast to other *Solanum* groups, especially to closely related tuber-bearing species of *Petota*.

Our calculations indicated that the lowest level of intragenomic IGS sequence similarity is demonstrated by *S. melongena-1* and *-2* (56 and 51.4%), *S. sogarandinum* (54.9%), *S. wendlandii* (58.4%), and *S. lasiophyllum* (59.4%). In *S. melongena-1* and *-2*, it is due to simultaneous existence of short and long (containing extra-long duplication, see Figure 2 and Supplementary Material) repeats in the genome, while in *S. melongena-3*, which possesses only long repeats, the similarity amounts to 95.2–99.4%. Similarly, in *S. lasiophyllum*, three adjacent deletions (65 bp in total) present in one of six ribotypes is the main reason for the low level of intragenomic similarity. In contrast, two mechanisms contribute to the low similarity of IGS in *S. sogarandinum:* (i) a long deletion in one ribotype and (ii) multiple base substitutions in another. In the second case, 33 of 210 bp in the same ribotype was changed compared to the consensus sequence. Notably, these mutations are present in the 3′ IGS region, which likely contains external promoter elements, suggesting putative pseudogenization of the ribotype. Putative 5S rDNA-related pseudogenes have already been described for members of *Solanum* and other genera of Solanaceae (Volkov et al., 2001, 2017).

In *S. wendlandii*, similar to *S. sogarandinum*, two mechanisms, an insertion and a large number of base substitutions, cause increased heterogeneity of IGS sequences (Figure 3). Accordingly, we excluded long (more than 5 bp) deletions and multiple base substitutions from our calculations and found that in this case the minimum level of intragenomic similarity of the IGS in *Solanum* species is around 85–90%.

In general, our results indicate that there are two mechanisms, long indels and multiple base substitutions, that significantly affect the heterogeneity of the IGS in *Solanum* species. Multiple base substitutions are rare events: out of about 900 analyzed sequences, only five ribotypes bearing multiple base substitutions were identified in four plant accessions (*S. kurtzianum, S. pinnatisectum-2*, *S. sogarandinum-1*, *and S. vernei-3*), while long indels are much more common.

The question, “what can be the source of the IGS intragenomic polymorphism?” arises. There are at least two possible options: (i) new variants emerge in the genome itself by accumulation of mutations and (ii) new variants appear in the genome as a result of introgression of genetic material due to interspecific hybridization. It is well known that in sect. *Petota*, especially in the *S. brevicaule* complex, interspecific hybridization is widespread at both the diploid and polyploid levels (Hawkes, 1990; Spooner et al., 2014). Among the 125 examined accessions representing sect. *Petota*, two or more structural variants of the IGS were found in 25 cases, and interspecific hybridization seems to be a plausible explanation for this polymorphism, especially when structurally different IGS variants (e.g., A and D) occur in the same genome. However, further research is needed to confirm this option.

Our data suggest that long indels and multiple base substitutions appeared repeatedly during the molecular evolution of IGS in the *Solanum* genus. However, it seems that they were mostly not conserved and eliminated. Accordingly, the length of IGS and contents of GC pairs did not change significantly during the course of speciation (see above). The likely reason for this negative selection could be the association between indels/multiple base substitutions and pseudogenization of 5S rDNA. Accordingly, it looks that the main road of the IGS molecular evolution seems to be step-wise accumulation of single base substitution or short indels.

### Intraspecific 5S rDNA Heterogeneity

It could be anticipated that different accessions of same species possess identical/similar sets of ribotypes. To check this assumption, we examined two to three accessions for 25 diploid species (Tables 1, 2 and Supplementary Material), and in most cases, only one IGS variant was actually found. However, in seven species (*S. ambosinum, S. commersonii, S. microdontum, S. okadae, S. phureja, S. raphanifolium*, and *S. stenotomum*; Figure 8 and Supplementary Material), one or two structural variants were detected in different accessions, indicating presumptive interspecific hybridization.

Among the species studied, we examined the highest number of accessions in *S. bukasovii*, which was considered one of the ancestors of cultivated potato (Ugent, 1970; Hawkes, 1990; Hosaka, 1995; Spooner et al., 2005; Hardigan et al., 2015). This close relationship was also confirmed in our previous study by analyzing the 5′-external transcribed spacer (ETS) region of nuclear 35S rDNA (Volkov et al., 2003). According to the comparison of whole plastid genomes, the species belongs to clade 4 North (Huang et al., 2019). Respectively, based on its taxonomic position, it might be expected that *S. bukasovii* should possess the IGS variant D. However, SV-A3 was previously found in the buk1 accession (Volkov et al., 2001), which indicates incongruence among different phylogenetic markers. To further clarify the issue, we analyzed ten accessions of *S. bukasovii* in this study and found an extreme variability of the 5S IGS set. Two accessions (buk1 and buk2) contain only SV-A3 in the genome, while six others (buk3, buk4, buk5, buk7, bukm2, and bukm3) possess different D variants, SV-D1,-D4,-D7,-D8,-D9, and-D10 (see Table 4). Two remaining accessions (buk6 and bukm1) have both SV-A3 and –D4 or-D10. The D variants detected in accessions of *S. bukasovii* belong to different clusters in the median-joining network (Figure 8) and are identical or very similar to the D variants of several species (e.g., *S. achacachense, S. ambosinum, S. canasense, S. marinasense, S. phureja, S. stenotomum*, etc.) belonging to clade 4 North + cultivated (Huang et al., 2019). Similarly, the SV-A3 found in *S. bukasovii* is identical/similar to that of *S. abancayense, S. multiinterruptum, S. raphanifolium*, and *S. chaucha.* This unusual diversity of IGS might indicate a complex hybridogenic origin: i.e., some of the examined accessions could represent natural hybrids between *S. bukosovii* and different *Petota* species. The genetic heterogeneity of *S. bukosovii* accessions and the putative hybrid origin of some of them by crossing with *S. sparsipilum* or *S. raphanifolium* have recently been demonstrated using plastid and mitochondrial markers (Achakkagari et al., 2020, 2021; Bozan, 2021).

### Origin of Polyploid Species

There are several polyploids among the studied species. Two of them, *S. chaucha* and *S. juzepczukii*, are triploids. It was originally postulated that *S. chaucha* arose as a result of hybridization between diploid *S. phureja* and tetraploid *S. tuberosum* ssp. *andigena* (Bukasov, 1939), but later, *S. chaucha* was recognized as the autotriploid of *S. phureja* (Bukasov, 1978). In contrast, Hawkes (1962, 1990) suggested that *S. chaucha* originated from hybridization between *S. tuberosum* ssp. *andigena* and diploid species *S. stenotomum*. Our analysis indicates an allotriploid origin of *S. chaucha*, as three structural variants of IGS, SV-A3,-D8, and-D10, are found in its genome (Table 4 and Figure 8). The molecular data confirm the close relationship among *S. chaucha, S. phureja* (accession phu2 but not phu1), *S. tuberosum* ssp. *andigena*, and a diploid species, *S. stenotomum* subsp. *goniocalyx;* all of which have SV-D8. However, the origin of *S. chaucha* appeared to be more complicated, because other structural variants of the IGS had to be inherited from species belonging to clusters A3 and D10.

It is widely believed that the triploid species *S. juzepczukii* originated from a natural cross between a cultivated diploid, *S. stenotomum*, and the wild tetraploid species *S. acaule* (maternal form), and pentaploid species *S. curtilobum* arose from a combination of non-reduced gamete of *S. jusepczukii* (3×, maternal form) with a reduced (2×) gamete of tetraploid *S. tuberosum* ssp. *andigena* (Bukasov, 1939; Hawkes, 1962, 1990; Schmiediche et al., 1980). The application of nuclear molecular markers confirmed this scenario (Rodríguez et al., 2010), but an alternative origin of *S. curtilobum* by hybridization of triploid species of the Andigenum group (non-reduced gamete, maternal form) and *S. acaule* was later proposed (Gavrilenko et al., 2013; Spooner et al., 2014). We found that the above-mentioned species contain the following IGS variants: *S. acaule*: SV-D10, *S. jusepczukii*: SV-A2 and-D2; *S. tuberosum* ssp. *andigena*: SV-D6,-D8, and-D9; *S. curtilobum*: SV-A2,-D1,-D2,-D9, and-D10. Three sets of IGS variants were identified for three accessions of *S. stenotomum* (stn1: SV-D1,-D9; stn2: SV-D4; gon: SV-D8). Remarkably, five IGS structural variants have been identified for pentaploid *S. curtilobum* that demonstrate the complex hybrid nature of this species. The analysis of the results showed that several common IGS structural variants are present in genomes of the examined species, which are correspondingly co-localized in the median-joining network (see Figure 8). However, no expected additivity of IGS structural variants from the presumptive parents in the derived alloploid progeny was found. It looks probable that the origin of *S. jusepczukii* and *S. curtilobum* may involve more parental diploids and requires further clarification using more plant accessions and additional molecular markers.

Two structural variants of IGS, SV-A2, and -D1, are present in the genome of *S. medians*, indicating its origin from a cross between potato species included in clusters A2 and D1. For *S. medians*, diploid and triploid populations were reported (Hijmans et al., 2007). Unfortunately, we could not find in SRA information about the ploidy level of the plant accession analyzed here.

According to a GISH analysis of meiotic preparations, *S. stoloniferum* appears to be an allotetrapolyploid species with a genomic constitution of AABB. It has been suggested that the species originated from *S. verrucosum* as the A genome donor and another North or Central American diploid species (e.g., *S. cardiophyllum, S. ehrenbergii*, or *S. jamesii*) as the B genome donor (Hijmans et al., 2007). In that case, regarding our data (Table 4 and Figure 8), it is expected that *S. stoloniferum* inherited SV-A1, -A2, or -B from *S. cardiophyllum, S. ehrenbergii*, or *S. jamesii*, respectively, as well as SV-D4 from *S. verrucosum*. However, *S. stoloniferum* possesses only D-variants of IGS, namely, SV-D1 and SV-D3, that could be inherited from *S. ambosinum* and from one of the species that belong to cluster D3.

Previously, we have discussed the presumptive origin of a tetraploid, *S. acaule*, and *S. tuberosum* as well as hexaploids *S. demissum* and *S. iopetalum* (Volkov et al., 2001). The new data confirm the close relationship between *S. demissum* and *S. acaule* (cluster D10) and more distant position of *S. iopetalum* (cluster D6), and provide new information on putative diploid ancestors of the polyploid species. Our novel data also show that the IGS variants of two accessions of *S. tuberosum* ssp. *andigena* belong to different clusters (tbrA1: clusters D4, D8, and D9, and tbrA2: clusters D3 and D10), indicating that these accessions have an independent hybrid origin from different parental diploids.

## Data Availability Statement

The datasets presented in this study can be found in online repositories. The names of the repository/repositories and accession number(s) can be found in the article/Supplementary Material.

## Author Contributions

RV, YT, and IP conceived and designed the study. YT, AS, and LK performed the experiments and collected the material. YT, RV, AS, IP, and VH analyzed the data. RV and VH wrote the manuscript. All authors contributed to the article and approved the submitted version.

## Conflict of Interest

The authors declare that the research was conducted in the absence of any commercial or financial relationships that could be construed as a potential conflict of interest.

## Publisher’s Note

All claims expressed in this article are solely those of the authors and do not necessarily represent those of their affiliated organizations, or those of the publisher, the editors and the reviewers. Any product that may be evaluated in this article, or claim that may be made by its manufacturer, is not guaranteed or endorsed by the publisher.

## Footnotes

1
https://rna.urmc.rochester.edu/RNAstructureWeb/

2
www.geneious.com
