# Supplementary material for: 5S Ribosomal DNA of Genus Solanum: Molecular Organization, Evolution, and Taxonomy
Source: Front Plant Sci. 2022 Apr 13;13:852406. doi: 10.3389/fpls.2022.852406 (PMC9043955; doi:10.3389/fpls.2022.852406)
Supplement: Supplementary file 1 [file Data_Sheet_1.pdf]

Tynkevich YO, Shelyfist AY, Kozub LV, Hemleben V, Panchuk II and Volkov RA (2022)

5S Ribosomal DNA of Genus *Solanum*:

Molecular Organization, Evolution, and Taxonomy.

Front. Plant Sci. 13:852406.

doi: 10.3389/fpls.2022.852406

Supplementary Material

Alignment of 5S rDNA IGS sequences of *Solanum* species

Note: This file represents a comparison of sequences of major and minor variants of 5S rDNA IGS (ribotypes) present in genomes of Solanum species.

S. abancayense, abn

```
Majority CTTTTTTGTTGAAATTCGGTCGTGTAGCATTTAAATATTATATT-ATTTTTCTGCAGAAACGACATTCGGATTGAGACGT
                10        20        30        40        50        60        70        80
         ---------+---------+---------+---------+---------+---------+---------+---------+
abn-C1R1 ............................................-...................................
abn-C1R2 ............................................-...................................
abn-C1R3 ............................................-...................................
abn-C2R1 ............................................-...................................
abn-C3R1 ............................................-...................................
abn-C3R2 ......G...................AT................T......T............................
abn-C3R3 ......G...................AT................T......T............................

Majority CGTTAGGACAGGTGATGGGGGCGTTGAGGATGGGCGTGACGGGCGGCGTCATGCGTCGGTGCGTGGAGGCTAGGTCGGTG
                90       100       110       120       130       140       150       160
         ---------+---------+---------+---------+---------+---------+---------+---------+
abn-C1R1 ....G.......................................................................T...
abn-C1R2 ...G..................................................C.........................
abn-C1R3 .......GTG......................................................................
abn-C2R1 ................................................................................
abn-C3R1 ..........................................A.....................................
abn-C3R2 ........................G.......................................................
abn-C3R3 ................................................................................

Majority GGGGGCAGGCTAGGGCGTTGGAGGAAGGAGGTGTTTAATAGAATTTAGAGTGCTATGAATGAT
               170       180       190       200       210       220
         ---------+---------+---------+---------+---------+---------+---
abn-C1R1
abn-C1R2 ...................................C...........................    222 bp
abn-C1R3 ...............................................................    222 bp
abn-C2R1 ..................................CC...........................    222 bp
abn-C3R1 ...............................................................    222 bp
abn-C3R2 ...............................................................    223 bp
abn-C3R3 ...............................................................    223 bp
```

abn-C1R1	abn-C1R2	abn-C1R3	abn-C2R1	abn-C3R1	abn-C3R2	abn-C3R3	
***	97.8	97.8	98.2	98.7	96.4	96.9	abn-C1R1
	***	97.3	98.7	98.2	96.0	96.4	abn-C1R2
		***	97.8	98.2	96.0	96.4	abn-C1R3
			***	98.7	96.4	96.9	abn-C2R1
				***	96.9	97.3	abn-C3R1
					***	99.6	abn-C3R2
						***	abn-C3R3

Similarity: 96.0-99.6

S. abutiloides, abu

```
Majority  CCXTTTTGTCGAAATTCGGCACAACTTCGTCTGTTTGATGATATTTTTTTCCCCGXAGAAACGATATTTGGGCCGAGAXG
                  10        20        30        40        50        60        70        80
          ---------+---------+---------+---------+---------+---------+---------+---------+
pSabu-5S1 ..T....................................................G......................C.
pSabu-5S4 ..A....................................................C......................G.

Majority  TCGTTGGGACTGGCGATGAGGACGGGCGTXCCAGGCGTCACCACXCGCCGGTGCGTGCAGGCTAGGGCGGTGGGGTGCAG
                  90       100       110       120       130       140       150       160
          ---------+---------+---------+---------+---------+---------+---------+---------+
pSabu-5S1 .............................A..............G...................................
pSabu-5S4 .............................G..............C...................................

Majority  GCTAGGGCGGTGGGAGGAATGTGGTTTAATAXAATTTAGAGTGCAAGGAATGAX
                 170       180       190       200       210
          ---------+---------+---------+---------+---------+----
pSabu-5S1 ...............................C.....................C  214 bp
pSabu-5S4 ...............................G.....................T  214 bp
```

pSabu-5S1	pSabu-5S4	
***	96.7	pSabu-5S1
	***	pSabu-5S4

Similarity: 96.7

S. acaule, acl

```
Majority CCTTTTTGTCGAAATTCGGTCGTGTAATAGAAAAAA-TATTATTATTTATTTTTTGTAGAAACGACGTCGTTAGGACTAA
                10        20        30        40        50        60        70        80
         ---------+---------+---------+---------+---------+---------+---------+---------+
aca5S.2  ....................................-...........................................
aca5S.3  ...................................T-............A...-..........................
aca5S.4  ....................................A...........................................
aca5S.5  ....................................-...........................................

Majority CAGGTGATGGGGGCGTTGGGAGGATGGGCGTGACGGGCGGCGTCATGCGTCGGTGCGCGTGGAGGCTAGGTCGGTGGGGG
                90       100       110       120       130       140       150       160
         ---------+---------+---------+---------+---------+---------+---------+---------+
aca5S.2  ................................................................................
aca5S.3  ................................................................................
aca5S.4  ................................................................................
aca5S.5  ................................................................................

Majority G-CAGGCTAGGGCGTTGGGAGGAAGGAGGTGTTTAATAGAATTTAGAGTGCTATGAATGAT
               170       180       190       200       210       220
         ---------+---------+---------+---------+---------+---------+-
aca5S.2  .-.......................A.........................A.........   219 bp
aca5S.3  .G...........................................................   219 bp
aca5S.4  .-...........................................................   220 bp
aca5S.5  .-...........................................................   219 bp
```

aca5S.2	aca5S.3	aca5S.4	aca5S.5	
***	97.3	98.6	99.1	aca5S.2
	***	97.7	98.2	aca5S.3
		***	99.5	aca5S.4
			***	aca5S.5

Similarity: 97.3-99.5

S. achacachense, ach

```
Majority  CCTTTTTGTCGAAATTCGGTCGTGTAATT-----------ATTATTTATTTTTTGCAGAAAGTCGGTCGTTAGGACAGGT
                  10        20        30        40        50        60        70        80
          ---------+---------+---------+---------+---------+---------+---------+---------+
ach-C1R1  ....................AC.......GAAAAAAATATT....................CGAC...............
ach-C1R4  ....................AC.......GAAAAAAATATT......G.............CGAC...............
ach-C1R2  .............................-----------................T.......................
ach-C1R3  .............................-----------........................................
ach-C7R1  .........................C...-----------........................................

Majority  GATGGGGGCGTTGAGGATGGGCGTGACGGGCGG----CGTCATGCGTCGGTGCGCGTGGAGGCTAGGTCGGTGGGGGGCA
                  90       100       110       120       130       140       150       160
          ---------+---------+---------+---------+---------+---------+---------+---------+
ach-C1R1  .G...............................GGGG..........A................................
ach-C1R4  .G...............................GCGG..........A.......................G........
ach-C1R2  .................................----...........................................
ach-C1R3  .................................----...........................................
ach-C7R1  .................................----...........................................

Majority  GGCTAGGGCGTTGGGAGGAAGGAGGTGTTTAATAGAATTTAGAGTGCTATGAATGAT
                 170       180       190       200       210
          ---------+---------+---------+---------+---------+-------
ach-C1R1  T...................................................G....   217 bp
ach-C1R4  T........................................................   217 bp
ach-C1R2  ...............................G.........................   202 bp
ach-C1R3  .........................................................   202 bp
ach-C7R1  ............................A............................   202 bp
```

ach-C1R1	ach-C1R4	ach-C1R2	ach-C1R3	ach-C7R1	
***	98.2	87.6	88.5	87.6	ach-C1R1
	***	87.1	88.0	87.1	ach-C1R4
		***	99.1	98.2	ach-C1R2
			***	99.1	ach-C1R3
				***	ach-C7R1

Similarity: 87.1-99.1

S. acroglossum, acg

```
Majority  CCTTTTGGTCGAAATTCGGTCGAGTAATTGAAAAAATATATTATGATTTTTTGCAGAAACGACATTCGGATTGAGACGTC
                  10        20        30        40        50        60        70        80
          ---------+---------+---------+---------+---------+---------+---------+---------+
acg-C1R1  ..........T.................................................................C....
acg-C1R2  ...........................................................................C....
acg-C2R1  ............................................TG..................................
acg-C3R1  ................................................................................
acg-C4R1  ........A.......................................................................
acg-C5R1  ............................................T...................................
acg-C6R1  ............................................T...................................

Majority  GTTAGGACGGGTGATGGGGGCGTTGAGGAAGGGCGTGACGGGCGGCGTCATGCGTCGGTGCGTGGAGGCTAGGCCGGTGG
                  90       100       110       120       130       140       150       160
          ---------+---------+---------+---------+---------+---------+---------+---------+
acg-C1R1  ................................................................................
acg-C1R2  ................................................................................
acg-C2R1  ..C.............................................................................
acg-C3R1  ................................................................................
acg-C4R1  ................................................................................
acg-C5R1  ..C..............................................G..............................
acg-C6R1  ..C.............................................................................

Majority  GGGTCAGGCAAGGGCGTTGGGAGGAAGGAGGTGTTTAATAGAATTTAGAGTGCAATGAATGAT
                 170       180       190       200       210       220
          ---------+---------+---------+---------+---------+---------+---
acg-C1R1  ....................................G..........................    223 bp
acg-C1R2  ...............................................................    223 bp
acg-C2R1  .................C............................................C    223 bp
acg-C3R1  ...............................................................    223 bp
acg-C4R1  ...........................T...................................    223 bp
acg-C5R1  ...............................................................    223 bp
acg-C6R1  .................C.............................................    223 bp
```

acg-C1R1	acg-C1R2	acg-C2R1	acg-C3R1	acg-C4R1	acg-C5R1	acg-C6R1	
***	99.1	96.4	98.7	97.8	97.3	97.3	acg-C1R1
	***	97.3	99.6	98.7	98.2	98.2	acg-C1R2
		***	97.8	96.9	98.2	99.1	acg-C2R1
			***	99.1	98.7	98.7	acg-C3R1
				***	97.8	97.8	acg-C4R1
					***	99.1	acg-C5R1
						***	acg-C6R1

Similarity: 96.4-99.6

S. acroscopicum, acs

```
Majority  CCTTTTGGTCGAAATTCGGTCGTGTAATTGAAAAAATATATTTATTATTTTTGCAGAAACGACATTGGGATTGAGACGTC
                  10        20        30        40        50        60        70        80
          ---------+---------+---------+---------+---------+---------+---------+---------+
acs-C1R1  ......................................................................G.........
acs-C1R2  ................................................................................
acs-C1R3  ................................................................................
acs-C2R1  ....C..........C................................................................
acs-C3R1  ................................................................................
acs-C4R1  ................................................................................
acs-C5R1  ............CT.C.TT...........T.................................................
acs-C6R1  ...............C..............T.................................................
acs-C7R1  ..G.............................................................................
acs-C7R2  ...............C................................................................
acs-C8R1  .......................................G........................................

Majority  GTTAGGACAGGTGATGGGGGCGTTGAGGATGGGCGTGACGGGCGGCGTCGTGCGTCGGTGCGCGGAGGCTAGGTCGGTGG
                  90       100       110       120       130       140       150       160
          ---------+---------+---------+---------+---------+---------+---------+---------+
acs-C1R1  ................................................................................
acs-C1R2  .............................A..................................................
acs-C1R3  ................................................................................
acs-C2R1  ................................................................................
acs-C3R1  ................................................................................
acs-C4R1  ................................................................................
acs-C5R1  ................................................................................
acs-C6R1  ................................................................................
acs-C7R1  ................................................................................
acs-C7R2  ................................................................................
acs-C8R1  ................................................................................

Majority  GGGGTCAGGCTAGGGCGTTGGGAGGAAGGAGGTGTTT-AATAGAATTTAGAGTGCTATGAATGAT
                 170       180       190       200       210       220
          ---------+---------+---------+---------+---------+---------+-----
acs-C1R1  .....................................-...........................    224 bp
acs-C1R2  .....................................-...................C.......    224 bp
acs-C1R3  ..A..................................-...................C.......    224 bp
acs-C2R1  .....................................-...........................    224 bp
acs-C3R1  ...-.................................T...........................    224 bp
acs-C4R1  .....................................-...................C.......    224 bp
acs-C5R1  .....................................-...................C.......    224 bp
acs-C6R1  .....................................-..................G........    225 bp
acs-C7R1  ...-.................................T...........................    224 bp
acs-C7R2  ...-............................C....T...........................    224 bp
acs-C8R1  ...-.................................T...........................    224 bp
```

acs-C1R1	acs-C1R2	acs-C1R3	acs-C2R1	acs-C3R1	acs-C4R1	acs-C5R1	acs-C6R1	acs-C7R1	acs-C7R2	acs-C8R1	
***	98.7	98.7	98.7	98.7	99.1	96.4	98.2	98.2	97.8	98.2	acs-C1R1
	***	99.1	98.2	98.2	99.6	96.9	97.8	97.8	97.3	97.8	acs-C1R2
		***	98.2	98.7	99.6	96.9	97.8	98.2	97.8	98.2	acs-C1R3
			***	98.2	98.7	96.9	98.7	97.8	98.2	97.8	acs-C2R1
				***	98.7	96.0	97.8	99.6	99.1	99.6	acs-C3R1
					***	97.3	98.2	98.2	97.8	98.2	acs-C4R1
						***	97.3	95.6	96.0	95.6	acs-C5R1
							***	97.3	97.8	97.3	acs-C6R1
								***	98.7	99.1	acs-C7R1
									***	98.7	acs-C7R2
										***	acs-C8R1

Similarity: 95.6-99.6

S. aculeatissimum, acu

```
Majority CCTTTTTTGTCGAAATTCGGTCTGATCTCGTCTATCTATATTATTT-TTGGCTCAGGCGACATTTGTTTGGGTCGGGACG
                  10        20        30        40        50        60        70        80
         ---------+---------+---------+---------+---------+---------+---------+---------+
pSacu1   ..............................................-.................................
pSacu2   ..............................................-.................................
pSacu3   .......C......................................A.................................
pSacu4   ..............................................-.................................
pSacu5   ..............................................-.................................

Majority TCGTTAGGACGGTTGAGGAAGAAGGGCGTGGCCCTTTGGCGGTGGTGATGCAGGAGGCTAGGGCGTCGTGAGGAATGAGG
                  90       100       110       120       130       140       150       160
         ---------+---------+---------+---------+---------+---------+---------+---------+
pSacu1   .....G..........................................................................
pSacu2   ................................................................................
pSacu3   .................................T..........................................A...
pSacu4   ................................................................................
pSacu5   ................................................................................

Majority TTTAATAGAATTTAGAGTGGTAGGAATGAT
                 170       180       190
         ---------+---------+---------+
pSacu1   .C............................        189 bp
pSacu2   ..............................        189 bp
pSacu3   ..............................        190 bp
pSacu4   ..............................        189 bp
pSacu5   ..............................        189 bp
```

pSacu1	pSacu2	pSacu3	pSacu4	pSacu5	
***	98.9	96.8	98.9	98.9	pSacu1
	***	97.9	100.0	100.0	pSacu2
		***	97.9	97.9	pSacu3
			***	100.0	pSacu4
				***	pSacu5

Similarity: 96.8-100.0

S. aethiopicum, aet

```
Majority  CTTTTTTTGCCGAAATTTCGTCGTCTATTCGGCGAATGATTATTTTTTTTGGCGGTTTGGG-------------GAGAGA
                   10        20        30        40        50        60        70        80
          ---------+---------+---------+---------+---------+---------+---------+---------+
aet-C1R1  .............................................................TCGTTTGCTTGGG......
aet-C2R1  .............................................................-------------......
aet-C4R1  T............................................................-------------......
aet-C6R1  ........T....................................................-------------......

Majority  CGTCGTTAGGACGGTTGAGGAAGGGCGGTGGCGTGCATGGCTAGGGCGTGGGGGGAGGAATGAGGCTTAATAGAATTAAG
                   90       100       110       120       130       140       150       160
          ---------+---------+---------+---------+---------+---------+---------+---------+
aet-C1R1  ...............................T.................................T..............
aet-C2R1  .....................................C..........................................
aet-C4R1  ................................................................................
aet-C6R1  ......................................................A.........................

Majority  AGTGCTAGAAATGAC
                  170
          ---------+-----
aet-C1R1  ...............    175 bp
aet-C2R1  ...............    162 bp
aet-C4R1  ...............    162 bp
aet-C6R1  ...............    162 bp
```

aet-C1R1	aet-C2R1	aet-C4R1	aet-C6R1	
***	90.9	90.9	90.3	aet-C1R1
	***	98.9	98.3	aet-C2R1
		***	98.3	aet-C4R1
			***	aet-C6R1

Similarity: 90.3-98.9

S. ajanhuiri, ajh

```
Majority  CCTTTTTGTCGAAATTCGGTCGTGTAATAGAAAAAATATTATTATTTATTTTTTGTAGAAACGACGTCGTXAGGACTAAC
                   10        20        30        40        50        60        70        80
          ---------+---------+---------+---------+---------+---------+---------+---------+
ajh-C1R1  ......................................................................C.........
ajh-C2R1  .......................................A..............................T.........
ajh-C3R1  ......................................................................C.........
ajh-C4R1  ...................................................A..................T.........

Majority  AGGTGATGGGGGCGTTGGGAGGATGGGCGTGACGGGCGGCGTCATGCGTCGGTGCGCGTGGAGGCTAGGTCGGTGGGGGX
                   90        100       110       120       130       140       150       160
          ---------+---------+---------+---------+---------+---------+---------+---------+
ajh-C1R1  ..T............................................................................A
ajh-C2R1  ................................................................A..............G
ajh-C3R1  ...............................................................................A
ajh-C4R1  ...................G.............................................G.............G

Majority  CAGGCTAGGXCGTTGGGAGGAAGGAGGTGTTTAATAXAATTTAGAGTGCTATGAATGAT
                   170       180       190       200       210
          ---------+---------+---------+---------+---------+---------
ajh-C1R1  .........T..........................C......................   219 bp
ajh-C2R1  .........G...........G..............G............A.........   219 bp
ajh-C3R1  .........T..........................C......................   219 bp
ajh-C4R1  .........G..........................G......................   219 bp
```

ajh-C1R1	ajh-C2R1	ajh-C3R1	ajh-C4R1	
***	95.9	99.5	96.3	ajh-C1R1
	***	96.3	96.8	ajh-C2R1
		***	96.8	ajh-C3R1
			***	ajh-C4R1

Similarity: 95.9-99.5

S. albornozii, abz

```
Majority  CCTTTTGGTCGAAATTCGATCGTGCAATTTTAAAAATATATAATTATTATTTTTGCAGAAACGACATTCGGACGGAGACG
          ---------+---------+---------+---------+---------+---------+---------+---------+
                   10        20        30        40        50        60        70        80
abz-C1R1  .....C...................G......................................................
abz-C1R2  ................................................................................
abz-C1R3  .........................G......................................................
abz-C2R1  ................................................................................
abz-C3R1  ................................................................................

Majority  TCGTTAGGACAGGTGATGGGGGGAGTCACGCGTCGGTGCGT---------GGTCGGTGGGGGTCAGGCTAGGGCGTTGGG
          ---------+---------+---------+---------+---------+---------+---------+---------+
                   90       100       110       120       130       140       150       160
abz-C1R1  .........................................---------..............................
abz-C1R2  .........................................---------..............................
abz-C1R3  .........................................---------..............................
abz-C2R1  .......................C...............---------TA..............................
abz-C3R1  .......................C.................GGGGGGCTA..............................

Majority  AGGAAGGAGGTGTTTAATAGAATTTAGAGTGCTATGAATGAT
          ---------+---------+---------+---------+--
                  170       180       190       200
abz-C1R1  ..........................................    193 bp
abz-C1R2  ..........................................    193 bp
abz-C1R3  ...........................G..............    193 bp
abz-C2R1  ..........................................    193 bp
abz-C3R1  ..........................................    202 bp
```

abz-C1R1	abz-C1R2	abz-C1R3	abz-C2R1	abz-C3R1	
***	99.0	99.0	96.5	94.1	abz-C1R1
	***	99.0	97.5	95.0	abz-C1R2
		***	96.5	94.1	abz-C1R3
			***	95.5	abz-C2R1
				***	abz-C3R1

Similarity: 94.1-99.0

S. albostellatum, als

```
Majority  CTTTTTTT-------TGCCTTCCGGAATTTGGTGGTGTATAGGTCGGTTGAGGGAGGGCTTTAGGCGGTTGGGTCGTTTG
                   10        20        30        40        50        60        70        80
          ---------+---------+---------+---------+---------+---------+---------+---------+
als-C1R1  ........-------.................................................................
als-C2R1  ........-------.....G.................G.........................................
als-C3R1  ........ATTTTTT.................................................................

Majority  CTTGGGCAGGGACGTCGTTAGGACGGCTGAGGAAGGGCGTCGTCGGCGCGTGGAGGCTAGGGCGGTGGCATTGGGGGGAG
                   90       100       110       120       130       140       150       160
          ---------+---------+---------+---------+---------+---------+---------+---------+
als-C1R1  ........................................................A.......................
als-C2R1  ...........................C........A.....C..............................C......
als-C3R1  ...........................................................C....................

Majority  GACTAAGGTTTAATAGAATTAAGAGTCGCTGGAATGAT
                  170       180       190
          ---------+---------+---------+--------
als-C1R1  ....G....................A............    191 bp
als-C2R1  ......................................    191 bp
als-C3R1  ......................................    198 bp
```

als-C1R1	als-C2R1	als-C3R1	
***	92.4	94.4	als-C1R1
	***	92.9	als-C2R1
		***	als-C3R1

Similarity: 92.4-94.4

S. ambosinum-1, amb1

```
Majority   CCTTTTTGTCGAAATTCGGTCGTGTAATAGAAAAAATATXATTATTTATTTTTTGXAGAAACGACGTCGTTAGGACAGGT
                   10        20        30        40        50        60        70        80
           ---------+---------+---------+---------+---------+---------+---------+---------+
amb1-C1R1  .................A.....................T...............C........................
amb1-C2R2  ............T..........................T...............C........................
amb1-C2R4  .......................................G...............T........................
amb1-C4R1  ......................C................G...............T........................

Majority   GATGGGGGCGTXXX-XXXXXXXXXGACGGGCGGCGTCATGCGTCGGTGCGCGTGGAGGCTAGGTCGGTGGGGGGCAGGCT
                   90       100       110       120       130       140       150       160
           ---------+---------+---------+---------+---------+---------+---------+---------+
amb1-C1R1  ...........TGAGGATGGGCGT........................................................
amb1-C2R2  A..........TGAAGATGGGCGT.........G..............................A...............
amb1-C2R4  ...........-------------........................................................
amb1-C4R1  ...........-------------........................................................

Majority   AGGGCGTTGGGAGGAAGGAGGTGTTTAATAGAATTTAGAGTGCTATGAATGAT
                  170       180       190       200       210
           ---------+---------+---------+---------+---------+---
amb1-C1R1  .....................................................   213 bp
amb1-C2R2  ................T....................................   213 bp
amb1-C2R4  .....................................................   200 bp
amb1-C4R1  ........................C.......C....................   200 bp
```

amb1-C1R1	amb1-C2R2	amb1-C2R4	amb1-C4R1	
***	96.7	92.5	91.1	amb1-C1R1
	***	90.6	89.2	amb1-C2R2
		***	98.6	amb1-C2R4
			***	amb1-C4R1

Similarity: 89.2-98.6

S. ambosinum-2, amb2

```
Majority CCTTTTTGTCGAAATTCGGTCGTGTAATAGAAAAAATATTATTATTTATTTTTTGTAGAAACGACGTCGTTAGGACTAAC
                        10        20        30        40        50        60        70        80
         ---------+---------+---------+---------+---------+---------+---------+---------+
amb2-C1R1    .............G..................................................................
amb2-C1R2    ................................................................................
amb2-C1R3    .................T..............................................................
amb2-C1R4    ................................................................................
amb2-C1R5    .....................TC.........................................A...............
amb2-C1R6    ......................C.........................................................
amb2-C1R7    .T................A....................................C....................----
amb2-C1R8    ......................C.........................................................
amb2-C1R9    ................................................................................
amb2-C1R10   ......................C.........................................................
amb2-C1R11   ......................G.................................................C.......
amb2-C1R12   ....................................................................A...........

Majority AGGTGATGGGGGCGTTGGGAGGATGGGCGTGACGGGCGGCGTCATGCGTCGGTGCGCGTGGAGGCTAGGTCGGTGGGGGG
                        90       100       110       120       130       140       150       160
         ---------+---------+---------+---------+---------+---------+---------+---------+
amb2-C1R1    ................................................................................
amb2-C1R2    .......................G........................................................
amb2-C1R3    ................................................................................
amb2-C1R4    ..............A...-.............................................................
amb2-C1R5    ................................................................................
amb2-C1R6    ...................................................................A............
amb2-C1R7    ................--..............................................................
amb2-C1R8    .....-..........................................................................
amb2-C1R9    ................................................................................
amb2-C1R10   ...........................T....................................................
amb2-C1R11   ................................................................................
amb2-C1R12   ................................................................................

Majority CAGGCTAGGGCGTTGGGAGGAAGGAGGTGTTTAATAGAATTTAGAGTGCTATGAATGAT
                       170       180       190       200       210
         ---------+---------+---------+---------+---------+---------
amb2-C1R1    ....G......................C...............................    219 bp
amb2-C1R2    ...........................................................    219 bp
amb2-C1R3    T..........................................................    219 bp
amb2-C1R4    ..............C............................................    218 bp
amb2-C1R5    .......A................T..................................    219 bp
amb2-C1R6    ......................T....................................    219 bp
amb2-C1R7    A..........................................................    213 bp
amb2-C1R8    ..........................................................G    218 bp
amb2-C1R9    ...........................................................    219 bp
amb2-C1R10   ...........................................................    219 bp
amb2-C1R11   ...........................................................    219 bp
amb2-C1R12   ............................................T..............    219 bp
```

amb2-C1R1	amb2-C1R2	amb2-C1R3	amb2-C1R4	amb2-C1R5	amb2-C1R6	amb2-C1R7	amb2-C1R8	amb2-C1R9	amb2-C1R10	amb2-C1R11	amb2-C1R12	
***	98.2	97.7	97.3	96.3	97.3	94.1	97.3	98.6	97.7	97.7	97.7	amb2-C1R1
	***	98.6	98.2	97.3	98.2	95.0	98.2	99.5	98.6	98.6	98.6	amb2-C1R2
		***	97.7	96.8	97.7	95.0	97.7	99.1	98.2	98.2	98.2	amb2-C1R3
			***	96.3	97.3	94.1	97.3	98.6	97.7	97.7	97.7	amb2-C1R4
				***	97.3	93.2	97.3	97.7	97.7	97.3	96.8	amb2-C1R5
					***	94.1	98.2	98.6	98.6	98.2	97.7	amb2-C1R6
						***	94.1	95.4	94.5	94.5	94.5	amb2-C1R7
							***	98.6	98.6	98.2	97.7	amb2-C1R8
								***	99.1	99.1	99.1	amb2-C1R9
									***	98.6	98.2	amb2-C1R10
										***	98.2	amb2-C1R11
											***	amb2-C1R12

Similarity: 94.1-99.5

S. americanum-1, ame1

```
Majority     CCTTTTTGTCGAAGTTCGGCATGATTTCGTGTATTTGATAATATATATTATTATTTTTTTGCAGAAACGGCATTCGTGCC
                     10        20        30        40        50        60        70        80
             ---------+---------+---------+---------+---------+---------+---------+---------+
ame1-C4R1    .................A..............................................................
ame1-C4R2    ................................................................................
ame1-C4R3    .A..................CAC...........................................C.............
ame1-C10R1   ...............................................G................................
ame1-C12R1   ....................................................-...........................
ame1-C17R1   ................................................................................
ame1-C20R1   .T.A......A.....................................................................

Majority     TAGACGTCGCTAGGACGGGCGACGGAGGCGCTGAGGACGGGCGTGACAGGCATGACGTCGGTGCGTGGAGGCTAGGGCGG
                     90       100       110       120       130       140       150       160
             ---------+---------+---------+---------+---------+---------+---------+---------+
ame1-C4R1    ..................................................................A.............
ame1-C4R2    .....C..........................................................................
ame1-C4R3    ..........................................................TT....................
ame1-C10R1   ........................A.............................C....A..A.................
ame1-C12R1   ............................T...................................................
ame1-C17R1   ............................A...................................................
ame1-C20R1   ................A...............................................................

Majority     CGGGGGAAATGCTATGGCGTTGGGAGGAAGGAGGTGTTTAATAGAATTTAGAGCGCAATGAATGAT
                    170       180       190       200       210       220
             ---------+---------+---------+---------+---------+---------+------
ame1-C4R1    ................................T.................................    226 bp
ame1-C4R2    ......C...........................................................    226 bp
ame1-C4R3    ..............................................C...................    226 bp
ame1-C10R1   ..............................---.................................    223 bp
ame1-C12R1   ..................................C...............................    225 bp
ame1-C17R1   ................................A.................................    226 bp
ame1-C20R1   ..................A...............................................    226 bp
```

ame1-C4R1	ame1-C4R2	ame1-C4R3	ame1-C10R1	ame1-C12R1	ame1-C17R1	ame1-C20R1	
***	97.8	95.1	95.6	97.3	98.2	96.5	ame1-C4R1
	***	95.6	95.6	97.8	98.2	96.9	ame1-C4R2
		***	93.4	95.1	95.6	94.7	ame1-C4R3
			***	95.1	96.0	94.2	ame1-C10R1
				***	98.2	96.5	ame1-C12R1
					***	96.9	ame1-C17R1
						***	ame1-C20R1

Similarity: 93.4-98.2

S. americanum-2, ame2

```
Majority      CCTTTTTGTCGAAGTTCGGCATGATTTCGTCTATTTGATAATATATATTATTATTTTTTTGCAGAAACGGCATTCGTGCC
                       10        20        30        40        50        60        70        80
              ---------+---------+---------+---------+---------+---------+---------+---------+
ame2-C1R1     ................................................................................
ame2-C1R2     ................................................................................
ame2-C2R1     ...................................................G...........................-
ame2-C10R1    ..........AC................................................TA..................
ame2-C12R1    ...............................................................................-
ame2-C19R1    ...G............................................................................

Majority      TAGACGTCGCTAGGACGGGTGACGGAGGCGCTGAGGACGGGCGTGACAGGCATGCCGTCGGTGCGTGGAGGCTAGGGCGG
                       90       100       110       120       130       140       150       160
              ---------+---------+---------+---------+---------+---------+---------+---------+
ame2-C1R1     ................................................................................
ame2-C1R2     ................................................................................
ame2-C2R1     --------........................................................A...............
ame2-C10R1    ..T.............................................................................
ame2-C12R1    --------........A...............................................................
ame2-C19R1    ........A.......................................................................

Majority      CGGGGGACATGCTATGTCGTTGGGAGAAAGGAGGTGTTTAATAGAATTTAGAGCGCAATGAATGAT
                      170       180       190       200       210       220
              ---------+---------+---------+---------+---------+---------+------
ame2-C1R1     ...............................................................A..    226 bp
ame2-C1R2     ..................................................................    226 bp
ame2-C2R1     ..................................................................    217 bp
ame2-C10R1    ..T...................................................A...........    226 bp
ame2-C12R1    ...........................................................A......    217 bp
ame2-C19R1    ...A................CT............................................    226 bp
```

ame2-C1R1	ame2-C1R2	ame2-C2R1	ame2-C10R1	ame2-C12R1	ame2-C19R1	
***	99.6	94.7	96.5	94.7	97.3	ame2-C1R1
	***	95.1	96.9	95.1	97.8	ame2-C1R2
		***	92.5	98.2	92.9	ame2-C2R1
			***	92.5	94.7	ame2-C10R1
				***	92.9	ame2-C12R1
					***	ame2-C19R1

Similarity: 92.5-99.6

S. americanum-3, ame3

```
Majority  CCTTTTTGTCGAAGTTCGGCATGATTTCGTGTATTTGATAATATATATTATTATTTTTTTXCAGAAACGGCATTCGTGCC
                   10        20        30        40        50        60        70        80
          ---------+---------+---------+---------+---------+---------+---------+---------+
ame3-C3R1 ............................................................G...................
ame3-C6R1 ............................................................A...................

Majority  TAGACGTCGCTAGGACGGGCGACGGAGGCGCTGAGGACGGGCGTGACAGGCATGACGTCGGTGCGTGGAGGCTAGGGCGG
                   90       100       110       120       130       140       150       160
          ---------+---------+---------+---------+---------+---------+---------+---------+
ame3-C3R1 ................................................................................
ame3-C6R1 ................................................................................

Majority  CGGGGGAAATGCTATGGCGTTGGGAGGAAGGAGGTGTTTAATAGAATTTAGAGCGCAATGAATGAT
                  170       180       190       200       210       220
          ---------+---------+---------+---------+---------+---------+------
ame3-C3R1 ..................................................................    226 bp
ame3-C6R1 ..................................................................    226 bp
```

ame3-C3R1	ame3-C6R1	
***	99.6	ame3-C3R1
	***	ame3-C6R1

Similarity: 99.6

S. andreanum, adr

```
Majority  CCTTTTGGTCGAAATTCGAACGTGTAATCGAAAAAATATATTTATTATTTTT-GCAGAAACGACATTCGGATGGAGACGT
                  10        20        30        40        50        60        70        80
          ---------+---------+---------+---------+---------+---------+---------+---------+
adr-C1R1  ....................................................-...........................
adr-C1R2  ................T...................................T...........................
adr-C1R3  ...............................G....................-...........................

Majority  CGTTAGGACAGGTGATGGGGGGGTTGAGGACGGGCGTCGTCATGCGTCGGTGCGTGGAGGCTAGGTCGGTGGGGGTCAGG
                  90       100       110       120       130       140       150       160
          ---------+---------+---------+---------+---------+---------+---------+---------+
adr-C1R1  ................................................................................
adr-C1R2  ................................................................................
adr-C1R3  .................................A..............................................

Majority  CTAGGGCGTTGGGAGGAGGGAGGTGTTTAATAGAATTTAGAGTGCTATGAATGAT
                 170       180       190       200       210
          ---------+---------+---------+---------+---------+-----
adr-C1R1  ................................A......................    214 bp
adr-C1R2  .......................................................    215 bp
adr-C1R3  .............................G.........................    214 bp
```

adr-C1R1	adr-C1R2	adr-C1R3	
***	98.6	98.1	adr-C1R1
	***	97.7	adr-C1R2
		***	adr-C1R3

Similarity: 97.7-98.6

S. anguivi, ang

```
Majority        CTTTTTTCGTCGTCTATTCGGCGAATGATTTTTTTTTTTTXX-GCGGTTGGGTXGTTTGCTTGGGGAGAGACGTCGTTAG
                        10        20        30        40        50        60        70        80
                ---------+---------+---------+---------+---------+---------+---------+---------+
ang-C1R1        ........................................---..........C..........................
ang-C2R1        ........................................T--..........T..........................
ang-C2R2        ........................................TT-..........T............G.............
ang-C10R1       T........G.....-..T..-..G..-.G..........TTT..........T..........................
ang-C14R1       ........................................---..........C..........................
ang-C14R2       ........................................TTT..........T..........................
ang-C15R1       .......................................----..........C..........................
ang-C15R2       .....................................------..........C..........................

Majority        GACGGTTGGGGAAGGGCGTCGCGGTAGCAAAAAGGGCTATGGCGGTGGTGTGCATGGCTAGGGCGTGGGGG-AGGAATGA
                ---------+---------+---------+---------+---------+---------+---------+---------+
                        90       100       110       120       130       140       150       160
                ---------+---------+---------+---------+---------+---------+---------+---------+
ang-C1R1        .......................A...............................................G........
ang-C2R1        .......................A...............................................G........
ang-C2R2        ........A.......................................................T......-........
ang-C10R1       ........A.............................G................................-........
ang-C14R1       .......................A...............................................GG......G
ang-C14R2       ........A....................................G.........................-........
ang-C15R1       .......................................................................-........
ang-C15R2       .......................................................................-........

Majority        GGTTTAATAGAATTAAGAGTTCTAGGAATGAT
                       170       180       190
                ---------+---------+---------+--
ang-C1R1        ....................GG..........    189 bp
ang-C2R1        ....................GG..........    190 bp
ang-C2R2        ................................    190 bp
ang-C10R1       ................................    188 bp
ang-C14R1       ....................GG..........    189 bp
ang-C14R2       ................................    191 bp
ang-C15R1       ...................G............    187 bp
ang-C15R2       ........................T.......    185 bp
```

ang-C1R1	ang-C2R1	ang-C2R2	ang-C10R1	ang-C14R1	ang-C14R2	ang-C15R1	ang-C15R2	
***	99.0	94.8	90.6	99.0	94.8	94.8	95.8	ang-C1R1
	***	95.8	91.7	97.9	95.8	93.8	94.8	ang-C2R1
		***	93.8	93.8	97.9	94.8	94.8	ang-C2R2
			***	89.6	94.8	91.7	90.6	ang-C10R1
				***	93.8	93.8	94.8	ang-C14R1
					***	95.8	94.8	ang-C14R2
						***	97.9	ang-C15R1
							***	ang-C15R2

Similarity: 89.6-99.0

S. anomalostemon, ano

```
Majority   CCTTTTTGTCGAAATTCGGCGTGATTCCGTCCATTTGATGATATTATTTTTCGCGGAAACGACATTTGGGCCGAGAGGTC
                   10        20        30        40        50        60        70        80
           ---------+---------+---------+---------+---------+---------+---------+---------+
ano-C7R1   ................................................................................
ano-C11R1  .........................C......................................................
ano-C12R1  ......................T.........................................................
ano-C14R1  ................................................................................
ano-C14R2  ...............................T................................................

Majority   GTCAGGACAGGTGACGGAGGGGCAGAGGATGGGAGCGCCAGGCGTCACCACGCGCCGGCGAGCGGAAAGGCGAGGGCGTT
                   90       100       110       120       130       140       150       160
           ---------+---------+---------+---------+---------+---------+---------+---------+
ano-C7R1   ................................................................................
ano-C11R1  .........................................C............T.........................
ano-C12R1  ...............................................................T................
ano-C14R1  ...........C....................................................................
ano-C14R2  .....................................................................T..........

Majority   GGGGCGCAGGCCGGGAGGAATGAGGTTTAATAGAATTTCGAGCGCAAGGAATGAC
                  170       180       190       200       210
           ---------+---------+---------+---------+---------+-----
ano-C7R1   .......................................................    215 bp
ano-C11R1  .......................................................    215 bp
ano-C12R1  .............................................T....G....    215 bp
ano-C14R1  .......................................................    215 bp
ano-C14R2  .......................................................    215 bp
```

ano-C7R1	ano-C11R1	ano-C12R1	ano-C14R1	ano-C14R2	
***	98.6	98.1	99.5	99.1	ano-C7R1
	***	96.7	98.1	97.7	ano-C11R1
		***	97.7	97.2	ano-C12R1
			***	98.6	ano-C14R1
				***	ano-C14R2

Similarity: 96.7-99.5

S. appendiculatum-1, ape1

```
Majority  CTTTTTTGTCGAAATTCGATCGAGTATTTTGTAATGTATATTTCTTTTTGCGGAAACGACATTCGGATCGAGACGTCGTT
                   10        20        30        40        50        60        70        80
          ---------+---------+---------+---------+---------+---------+---------+---------+
ape-C1R1  ..................................................T.............................
ape-C2R1  ................................................................................
ape-C3R1  .......A.........CG...C.......-....A..A..A.T.................C.A....T...........

Majority  AAGACAGGTGACGGGGGCGATTAAGATGGGCGTGACAATCGGCGTCATGCGTCGGTTCGGGGAGGCTAGGTCGGTGGGGG
                   90       100       110       120       130       140       150       160
          ---------+---------+---------+---------+---------+---------+---------+---------+
ape-C1R1  ........G................................................................T......
ape-C2R1  ............................A...........T.......................................
ape-C3R1  .....G.................T............GGG....A....T....A..G....A.A....T...........

Majority  GCAGGCTAGGGCGTXCGGAGGAAGGAGGTGTTTAATAGAATTTACAGTGGTAGGAAAXAT
                  170       180       190       200       210       220
          ---------+---------+---------+---------+---------+---------+
ape-C1R1  ..............A..........................................A..    220 bp
ape-C2R1  ..............T....A.....................................C..    220 bp
ape-C3R1  ..............-T....A.......................G............G..    218 bp
```

ape-C1R1	ape-C2R1	ape-C3R1	
***	96.4	85.5	ape-C1R1
	***	85.5	ape-C2R1
		***	ape-C3R1

Similarity: 85.5-96.4

S. appendiculatum-2, ape2

```
Majority       CTTTTTTGTCGAAATTCGATCGXGTATTTTGTAATGTATATTTCTTTTTGCGGAAACGACATTCGGATCGAGACGTCGTT
                       10        20        30        40        50        60        70        80
               ---------+---------+---------+---------+---------+---------+---------+---------+
ape2-C1R1      ......................A.........................................................
ape2-C2R1      .A....................C.........T....T..............A...........................
ape2-C3R1      ......................A.........................................................
ape2-C4R1      .......A.........CG...C.......T....A..A..A.T.................C.A....T...........
ape2-C5R1      .......A.........TG...C.......T....A......A.T...................A....T....G......
ape2-C6R1      ......................A.................................T.......................

Majority       AAGACAGGTGACGGGGGCGATTAAGATGXGCGTGACAATCXGCGTCATGCGTCGGTTCGGGGAGGCTAGGTCGGTGGGGG
                       90       100       110       120       130       140       150       160
               ---------+---------+---------+---------+---------+---------+---------+---------+
ape2-C1R1      ............................A...........T............................A..........
ape2-C2R1      ......A.....................G.T.........G................................T.A....
ape2-C3R1      ............................A...........T.......................................
ape2-C4R1      .....G.................G....G.......GGG.G..A....T....A..G....A.A....T...........
ape2-C5R1      .....G.................G....G.......GGG.G..A............G...........T...........
ape2-C6R1      ............................A...........T.......................................

Majority       GCAGGCTAGGGCGTXCGGAGGAAGGAGGTGTTTAATAGAATTTACAGTGGTAGGAAAGAT
                      170       180       190       200       210       220
               ---------+---------+---------+---------+---------+---------+
ape2-C1R1      ..............A.............................................    220 bp
ape2-C2R1      ..............A.............................................    220 bp
ape2-C3R1      ..............T....A.....................................C..    220 bp
ape2-C4R1      ..............-T....A.......................G...............    219 bp
ape2-C5R1      ..............-TT...A................C......G...............    219 bp
ape2-C6R1      ..............T....A..................................T..C..    220 bp
```

ape2-C1R1	ape2-C2R1	ape2-C3R1	ape2-C4R1	ape2-C5R1	ape2-C6R1	
***	94.5	98.2	85.9	87.3	97.3	ape2-C1R1
	***	93.6	84.1	85.5	92.7	ape2-C2R1
		***	85.5	86.8	99.1	ape2-C3R1
			***	95.5	84.5	ape2-C4R1
				***	85.9	ape2-C5R1
					***	ape2-C6R1

Similarity: 84.1-99.1

S. arcanum, arc

```
Majority  CCTTTTT-GTTGAAATTTGATCTCGTAATTGAG-AAAAAAATATACTAATTTATTTTTTTTGCGGAAAATACGTTCGGAT
                  10        20        30        40        50        60        70        80
          ---------+---------+---------+---------+---------+---------+---------+---------+
arc-C1R1  .......-.........................-..............................................
arc-C1R4  .......T.........................G..............................................
arc-C2R1  .......-........................--.................A........................A...

Majority  TGAGACGTCATTAGGATATGGGATGGTGGCGTCGGTGATGGGCGTGACGGGCGTCGT-CGTGCGTCGGTGCGTGGAGGGT
                  90        100       110       120       130       140       150       160
          ---------+---------+---------+---------+---------+---------+---------+---------+
arc-C1R1  .......................................................T.T......................
arc-C1R4  .........................................................-......................
arc-C2R1  ...................................G.....................-......................

Majority  TTTAAAGCGGGGGGCGGGCTAGGGCGTTGGGAGGAAGGTTGTGTTTAATAGATTTTAGAGTGCAATGAATGAC
                  170       180       190       200       210       220       230
          ---------+---------+---------+---------+---------+---------+---------+---
arc-C1R1  .........................................................................    231 bp
arc-C1R4  .........................................................................    232 bp
arc-C2R1  .........................................................................    229 bp
```

arc-C1R1	arc-C1R4	arc-C2R1	
***	98.3	97.4	arc-C1R1
	***	97.4	arc-C1R4
		***	arc-C2R1

Similarity: 97.4-98.3

S. aviculare, avi

```
Majority    CCTTTTTATCGAAATTCATCATAATTTTATCTATTTGGTGATTAATTTTATTTTTTAATTTTTGCGGAAAGGACATTCGG
                    10        20        30        40        50        60        70        80
            ---------+---------+---------+---------+---------+---------+---------+---------+
pSavi-5S2   ..............C.................................................................
pSavi-5S3   ................................T...............................................
pSavi-5S5   ................................................................................

Majority    GTGGAGACGTCGTTAGGACAAGTGATGGAGGCGTTTGAAATTGGCGTGAAAGGCGGCACCATGCGTCGGCAGGCTAGGGC
                    90       100       110       120       130       140       150       160
            ---------+---------+---------+---------+---------+---------+---------+---------+
pSavi-5S2   ....................G...........................................................
pSavi-5S3   ................................................................................
pSavi-5S5   ................................................................................

Majority    GTTGGGAGGAAGGAGGTGTTTAATAAAATTTGGAGTGCTAGGAATGAC
                   170       180       190       200
            ---------+---------+---------+---------+--------
pSavi-5S2   ................................................     208 bp
pSavi-5S3   ................................................     208 bp
pSavi-5S5   ................................................     208 bp
```

pSavi-5S2	pSavi-5S3	pSavi-5S5	
***	98.6	99.0	pSavi-5S2
	***	99.5	pSavi-5S3
		***	pSavi-5S5

Similarity: 98.6-99.5

S. avilesii, avl

```
Majority CCTTTTTGTCGAAATTCGGTCGTGTAATTGAAAAAATATATTTATTTATTTATTGCAGAAACGAAACGACGTCGTTAGGA
                  10        20        30        40        50        60        70        80
         ---------+---------+---------+---------+---------+---------+---------+---------+
avl-C1R1 ..............----------------------------------------------------....A.........
avl-C1R4 ..............----------------------------------------------------..............
avl-C1R2 .......T.T.........................................G......................C.....
avl-C1R6 .T................................................................T.............
avl-C2R1 ................................................................................
avl-C2R2 .............................................................A..................
avl-C2R3 ..................................................................T.............

Majority CAGGTGATGGGGGCGCTGGTGATGGGCGTGACGGGCGGCGTCATGCGTCGGTGCGCGTGGAGGCTAGGACGGTGGGGGGC
                  90        100       110       120       130       140       150       160
         ---------+---------+---------+---------+---------+---------+---------+---------+
avl-C1R1 ................................................................................
avl-C1R4 ................................................................................
avl-C1R2 ................................................................................
avl-C1R6 ....................C....................G.....................A................
avl-C2R1 ................................................................................
avl-C2R2 ..............................................................................C.
avl-C2R3 ...............................T................................................

Majority AGGCTAGGGCGTTGGGAGGAAGGAGGTGTTTAATAGAATTTAGAGTGCTATGAATGAT
                  170       180       190       200       210
         ---------+---------+---------+---------+---------+--------
avl-C1R1 ............................G.............................               166
avl-C1R4 ..........................................................               166
avl-C1R2 ..........................G.......................G.......               218
avl-C1R6 ........................T.................................               218
avl-C2R1 ..........................................................               218
avl-C2R2 ..........................................................               218
avl-C2R3 ..........................................................               218
```

avl-C1R1	avl-C1R4	avl-C1R2	avl-C1R6	avl-C2R1	avl-C2R2	avl-C2R3	
***	99.1	72.9	72.5	75.2	74.8	74.3	avl-C1R1
	***	73.9	73.4	76.1	75.7	75.2	avl-C1R4
		***	94.5	97.2	96.3	96.3	avl-C1R2
			***	97.2	96.3	97.2	avl-C1R6
				***	99.1	99.1	avl-C2R1
					***	98.2	avl-C2R2
						***	avl-C2R3

Similarity: 72.5-99.1

S. berthaultii-1, ber1

```
Majority  CTTTTTTGTCGAAATTCGGTCGTGTAATTGAAAGAATATATACATTTATTTTTTGCAGGAACGACGTCGTTAGGACAGGT
                  10        20        30        40        50        60        70        80
          ---------+---------+---------+---------+---------+---------+---------+---------+
ber_5S.1  ................................................................................
ber_5S.2  .........................................T......................................
ber_5S.3  ................................................................................
ber_5S.4  ................................................................................
ber_5S.5  .........................................T......................................

Majority  GATGGGGGCGTTGAGGATGGGCGTGACGGGGGGCGTCATGCGTCGGTGCGCGTGGAGGCTAGGTCGGCGGGGGGCAGGCT
                  90       100       110       120       130       140       150       160
          ---------+---------+---------+---------+---------+---------+---------+---------+
ber_5S.1  ................................................................................
ber_5S.2  .................A..............................................................
ber_5S.3  ................................................................................
ber_5S.4  ................................................................................
ber_5S.5  ................................................................................

Majority  AGGGCGTTGGGAGGAAGGAGGTGTTTAATAGAATTTAGAGTGCTATGAATGAT
                 170       180       190       200       210
          ---------+---------+---------+---------+---------+---
ber_5S.1  .....................................................    213 bp
ber_5S.2  ..........A..........................................    213 bp
ber_5S.3  .A...................................................    213 bp
ber_5S.4  .A...................................................    213 bp
ber_5S.5  .....................................................    213 bp
```

ber_5S.1	ber_5S.2	ber_5S.3	ber_5S.4	ber_5S.5	
***	98.6	99.5	99.5	99.5	ber_5S.1
	***	98.1	98.1	99.1	ber_5S.2
		***	100.0	99.1	ber_5S.3
			***	99.1	ber_5S.4
				***	ber_5S.5

Similarity: 98.1-100

S. berthaultii-2, ber2

```
Majority  CTTTTTTGTCGAAATTCGGTCGTGTAATTGAAAGAATATATTCATTTATTT----TTTGCAGGAACGACGTCGTTAGGAC
                   10        20        30        40        50        60        70        80
          ---------+---------+---------+---------+---------+---------+---------+---------+
ber2-C1R1 .........................................A.........----.........................
ber2-C1R3 .C.......G.......................A........T........----.........................
ber2-C1R4 ...................................................----.........................
ber2-C1R5 .C...............................A........T........ATTT.........................
ber2-C1R6 ...................................................----.........................
ber2-C1R7 TC...............................A......A.T........----....A....................
ber2-C1R8 ...................................................----.........................

Majority  AGGTGATGGGGGCGTTGAGGATGGGCGTGACGGGGGGCGTCATGCGTCGGTGCGCGTGGAGGCTAGGTCGGCGGGGGGCA
                   90       100       110       120       130       140       150       160
          ---------+---------+---------+---------+---------+---------+---------+---------+
ber2-C1R1 ................................................................................
ber2-C1R3 ................................................................................
ber2-C1R4 ........................................................................AA......
ber2-C1R5 ................................................................................
ber2-C1R6 .......T....................------------........................................
ber2-C1R7 .......................................-----------------------------------------
ber2-C1R8 ................................................................................

Majority  GGCTAGGGCGTTGGGAGGAAGGAGGTGTTTAATAGAATTTAGAGTGCTATGAATGAT
                   170       180       190       200       210
          ---------+---------+---------+---------+---------+-------
ber2-C1R1 .........................................................    213 bp
ber2-C1R3 .........................................................    213 bp
ber2-C1R4 .........................................................    213 bp
ber2-C1R5 .....T...A..............................G.....T...A......    217 bp
ber2-C1R6 .........................................................    201 bp
ber2-C1R7 .........................................................    172 bp
ber2-C1R8 ........................A.............G..................    213 bp
```

ber2-C1R1	ber2-C1R3	ber2-C1R4	ber2-C1R5	ber2-C1R6	ber2-C1R7	ber2-C1R8	
***	97.7	98.6	94.0	93.5	77.9	98.6	ber2-C1R1
	***	97.2	95.4	92.2	79.3	97.2	ber2-C1R3
		***	93.5	93.1	78.3	98.2	ber2-C1R4
			***	88.5	75.6	93.5	ber2-C1R5
				***	73.3	93.1	ber2-C1R6
					***	77.4	ber2-C1R7
						***	ber2-C1R8

Similarity: 73.3-98.6

S. betaceum, bet

```
Majority  CCCCTXGGAAGTCCTCGTGTTGCATCCCTCGTTTTTGTCGAAATTGGGCATAACTATTTGATGGTATTTTTTTTTCGCXG
                  10        20        30        40        50        60        70        80
          ---------+---------+---------+---------+---------+---------+---------+---------+
pBet-1    .....G..........................................................................T.
pBet-2    .....A..........................................................................A.

Majority  AAGCGACATCTGGGCCGAGACGTCGCTAGGACAGGCGCCGAGGATGGGCGTGGGTGGCGCCACCACGCGCGGGTGCGTGG
                  90       100       110       120       130       140       150       160
          ---------+---------+---------+---------+---------+---------+---------+---------+
pBet-1    ................................................................................
pBet-2    ................................................................................

Majority  AGGCTAGGGCGTTGGGAGGAATGTGGTTTAATAGAATTTAGAGTGCTAGGAATGXTGGATGCGATCATACCAGCACTAAC
                 170       180       190       200       210       220       230       240
          ---------+---------+---------+---------+---------+---------+---------+---------+
pBet-1    ......................................................A.........................
pBet-2    ......................................................G.........................

Majority  XCACCGGA
          --------
pBet-1    A.......    187 bp
pBet-2    G.......    187 bp
```

pBet-1	pBet-2	
***	98.4	pBet-1
	***	pBet-2

Similarity: 98.4

S. blanco-galdosii, blg

```
Majority  CCTTTTGGTCGAAACTTCGTGCCGAGTAAAACAATCAAAAAAATATTTATTATTTTTGCAGAAACGACATTCGGATGGAG
                  10        20        30        40        50        60        70        80
          ---------+---------+---------+---------+---------+---------+---------+---------+
blg-C1R1  .......................................................C........................
blg-C2R1  ..........................................T.....................................
blg-C2R2  ...........................C.............T.....A................................
blg-C2R3  ..........................................T.....................................
blg-C3R1  ................................................................................

Majority  ACGTCGTTAGGTCAGGTGATGGGGGATGGGCGTGACGGGCGGCGTCATGCGTCGGTGCGTGGGGGCTAGGACGGTGGGGG
                  90       100       110       120       130       140       150       160
          ---------+---------+---------+---------+---------+---------+---------+---------+
blg-C1R1  G...............................................................................
blg-C2R1  ................................................................................
blg-C2R2  ................................................................................
blg-C2R3  ...................A............................................................
blg-C3R1  ................................................................................

Majority  TCAGGCTAGGGCGTTGGGAGGAAGGAGGTGTTTAATAGAATTTAGAGTGCTATGAATGAT
                 170       180       190       200       210       220
          ---------+---------+---------+---------+---------+---------+
blg-C1R1  ..........C.....C...........................................     220 bp
blg-C2R1  ............................................................     220 bp
blg-C2R2  ................................................T...........     220 bp
blg-C2R3  ............................................................     220 bp
blg-C3R1  ........................................G...................     220 bp
```

blg-C1R1	blg-C2R1	blg-C2R2	blg-C2R3	blg-C3R1	
***	97.7	96.4	97.3	97.7	blg-C1R1
	***	97.7	99.5	99.1	blg-C2R1
		***	97.3	97.7	blg-C2R2
			***	98.6	blg-C2R3
				***	blg-C3R1

Similarity: 96.4-99.5

S. boliviense, blv

```
Majority  CCTTTTTGTCGAAATTTGGGTCGTGTAATTGAAAAAATATTATTATTTATTXTTTGCAGGGACGACGTCGTTAGGACAGG
                  10        20        30        40        50        60        70        80
          ---------+---------+---------+---------+---------+---------+---------+---------+
blv-C1R1  .................-.................................T...........................T
blv-C2R1  ...................................................C............................
blv-C3R1  .T.................................................T............................
blv-C3R2  .................T.................................C............................

Majority  TGATGGGGGCGTTGAGGATGGGCGTGACGGGCGGCGTCATGCGTCGGTGCGCGTGGAGGCTAGGTCGGTGGGGGGCAGGC
                  90       100       110       120       130       140       150       160
          ---------+---------+---------+---------+---------+---------+---------+---------+
blv-C1R1  .............................................................................T..
blv-C2R1  ................................................................................
blv-C3R1  ................................................................................
blv-C3R2  .......................................................A........................

Majority  TAGGGCGTTGGGAGGAAGGAGGTGTTTAXTAGAATTTAGAGTGCTATGAATGAT
                 170       180       190       200       210
          ---------+---------+---------+---------+---------+----
blv-C1R1  A...........................A.........................    213 bp
blv-C2R1  ............................A.........................    214 bp
blv-C3R1  ................T...........G.........................    214 bp
blv-C3R2  ............................G........C................    214 bp
```

blv-C1R1	blv-C2R1	blv-C3R1	blv-C3R2	
***	97.7	96.7	96.3	blv-C1R1
	***	98.1	98.1	blv-C2R1
		***	97.2	blv-C3R1
			***	blv-C3R2

Similarity: 96.3-98.1

S. brevicaule, brc

```
Majority  CCTTTTTGTCGAAATTTGGTCGTGTAATTGAAAAAATATTATTATTTATTTTTTGCAGGAACGACGTCGTTAGGACAGGT
                  10        20        30        40        50        60        70        80
          ---------+---------+---------+---------+---------+---------+---------+---------+
brc-C1R1  ................................................................................
brc-C1R2  A..............................................G.........T......................
brc-C1R3  ................................................................................
brc-C2R1  .................................--------.......................................

Majority  GATGGGGGCGTTGAGGATGGGCGTGACGGGCGGCGTCATGCGTCGGTGCGCGTGGAGGCTAGGTCGGTGGGGGGCAGGCT
                  90       100       110       120       130       140       150       160
          ---------+---------+---------+---------+---------+---------+---------+---------+
brc-C1R1  ...............................A................................................
brc-C1R2  ......................A.........................................................
brc-C1R3  ..........................................G.....................................
brc-C2R1  ..........G...........................................C.........................

Majority  AGGGCGTTGGGAGGAAGGAGGTGTTTAATAGAATTTAGAGTGCTATGAATGAT
                 170       180       190       200       210
          ---------+---------+---------+---------+---------+---
brc-C1R1  .....................................................    213 bp
brc-C1R2  .....................................................    213 bp
brc-C1R3  .....................................................    213 bp
brc-C2R1  .....................................................    205 bp
```

brc-C1R1	brc-C1R2	brc-C1R3	brc-C2R1	
***	97.7	99.1	94.8	brc-C1R1
	***	97.7	93.4	brc-C1R2
		***	94.8	brc-C1R3
			***	brc-C2R1

Similarity: 93.4-99.1

S. bukasovii f. multidissectum-1, bukm1

```
Majority     CCTTTTTGTCGAAATTCGGTCGTGTAATTTGTTTATTATTATTATTTATTTATTGCAGAACCGACGTCGTTAGGAGAGGT
                     10        20        30        40        50        60        70        80
             ---------+---------+---------+---------+---------+---------+---------+---------+
bukm1-C6R1   .T.......T..................A.-..A.A......-...AT...TC.......A....A.T-C....-.C.T.
bukm1-C7R1   ................................................................................
bukm1-C35R1  ................................................................................
bukm1-C35R2  ................................................................................
bukm1-C35R3  ....................................C...........................................

Majority     GATGG-GGGCGTTGAGGATGGGCGTGACGGGCGGCGTCATGCGTCGGTGCGCGTGGAGGCTAGGTCGGTGGGGGGCAGGC
                     90       100       110       120       130       140       150       160
             ---------+---------+---------+---------+---------+---------+---------+---------+
bukm1-C6R1   ..G.AT.............................................--...........................
bukm1-C7R1   .....-..........T...............................................................
bukm1-C35R1  .....-.....C....................................................................
bukm1-C35R2  .....-..........................................................................
bukm1-C35R3  .....-..........................................................................

Majority     TAGGGCGTTGGGAGGAAGGAGGTGTTTAATAGAATTTAGAGTGCTATGAATGAT
                    170       180       190       200       210
             ---------+---------+---------+---------+---------+----
bukm1-C6R1   ...........-............C.............................    207 bp
bukm1-C7R1   ......................................................    213 bp
bukm1-C35R1  ......................................................    213 bp
bukm1-C35R2  ......................................................    213 bp
bukm1-C35R3  ..............................T.......................    213 bp
```

bukm1-C6R1	bukm1-C7R1	bukm1-C35R1	bukm1-C35R2	bukm1-C35R3	
***	87.4	87.4	87.9	86.9	bukm1-C6R1
	***	99.1	99.5	98.6	bukm1-C7R1
		***	99.5	98.6	bukm1-C35R1
			***	99.1	bukm1-C35R2
				***	bukm1-C35R3

Similarity: 86.9-99.5

S. bukasovii f. multidissectum-2, bukm2

```
Majority    CCTTTTTGTCGAAATTCGGTCGTGTAATAGAAAAAATATTATTATTTATTTTTT-GCAGAAACGACGTCGTTAGGAC---
                     10        20        30        40        50        60        70        80
            ---------+---------+---------+---------+---------+---------+---------+---------+
bukm2-C1R1  ......................C...............................T......................---
bukm2-C1R2  ......................................................T......................---
bukm2-C2R2  ......................................................-......................---
bukm2-C3R1  ......................................................-.T....................TAA
bukm2-C2R1  ......................................................-.T....................TAA

Majority -AGGTGATGGGGGCGTTG--AGGATGGGCGTGACGGGCGGCGTCATGCGTCGGTGCGCGTGGAGGCTAGGTCGGTGGGGG
                     90        100       110       120       130       140       150       160
            ---------+---------+---------+---------+---------+---------+---------+---------+
bukm2-C1R1  -.................--....C.......................................................
bukm2-C1R2  -.................--............................................................
bukm2-C2R2  -.................--........................G...................................
bukm2-C3R1  C.................GG...............................................G............
bukm2-C2R1  C.................GG............................................................

Majority    GCAGGCTAGGGCGTTGGGAGGAAGGAGGTGTTTAATAGAATTTAGAGTGCTATGAATGAT
                     170       180       190       200       210       220
            ---------+---------+---------+---------+---------+---------+
bukm2-C1R1  ..........................................A.................     214 bp
bukm2-C1R2  ............................................................     214 bp
bukm2-C2R2  ..........................................A.................     213 bp
bukm2-C3R1  ............................................................     219 bp
bukm2-C2R1  ............................................................     219 bp
```

bukm2-C1R1	bukm2-C1R2	bukm2-C2R2	bukm2-C3R1	bukm2-C2R1	
***	98.6	98.2	94.5	95.0	bukm2-C1R1
	***	98.6	95.9	96.4	bukm2-C1R2
		***	95.5	95.9	bukm2-C2R2
			***	99.5	bukm2-C3R1
				***	bukm2-C2R1

Similarity: 94.5-99.5

S. bukasovii f. multidissectum-3, bukm3

```
Majority     CCTTTTTGTCGAAATTCGGTCGTGTAATTGAAAAAAXTATTATTATTTATTTTTTGCAGAAACTCGGTCGTTAGGACAGG
                      10        20        30        40        50        60        70        80
             ---------+---------+---------+---------+---------+---------+---------+---------+
bukm3-C1R1   ....................................A...........................................
bukm3-C2R1   ....................................-...........................................
bukm3-C4R1   ....................................A...........................................
bukm3-C8R1   .....A..............................-...........................................

Majority     TGXTGGGGGCGTTGAGGATGGGCGTGACGGGCGGCGTCAXGCGTCGGTGCGCGTGGAGGCTAGGTCGGTGGGGGGCAGGC
                      90       100       110       120       130       140       150       160
             ---------+---------+---------+---------+---------+---------+---------+---------+
bukm3-C1R1   ..G.....................C..............T........................................
bukm3-C2R1   ..A............................A.......G........................................
bukm3-C4R1   ..G....................................T........................................
bukm3-C8R1   ..A....................................G........................................

Majority     TAGGGCGTTGGGAGGAAGGAGGTGTTTAATAGAATTTAGAGTGCTATGAATGAT
                     170       180       190       200       210
             ---------+---------+---------+---------+---------+----
bukm3-C1R1   ......................................................   214 bp
bukm3-C2R1   ......................................................   213 bp
bukm3-C4R1   ......................................................   214 bp
bukm3-C8R1   ......................................................   213 bp
```

bukm3-C1R1	bukm3-C2R1	bukm3-C4R1	bukm3-C8R1	
***	97.7	99.5	97.7	bukm3-C1R1
	***	98.1	99.1	bukm3-C2R1
		***	98.1	bukm3-C4R1
			***	bukm3-C8R1

Similarity: 97.7-99.5

S. bukasovii-1, buk1

DIRECT SEQUENCING - 222 bp

S. bukasovii-2, buk2

```
Majority   CTTTTTTGTTGAAATTCGGTCGTGTAATATTTAAATATTATATTATTTTTCTGCAGAAACGACATTCGGATTGAGACGTC
                   10        20        30        40        50        60        70        80
           ---------+---------+---------+---------+---------+---------+---------+---------+
buk2-C1R1  ..............................................................................G.
buk2-C1R2  ...........................................................A....................
buk2-C2R1  ................................................................................
buk2-C3R1  ................................................................................

Majority   GTTAGGACAGGTGATGGGGGCGTTGAGGATGGGCGTGACGGGCGGCGTCATGCGTCGGTGCGTGGAGGCTAGGTCGGTGG
                   90       100       110       120       130       140       150       160
           ---------+---------+---------+---------+---------+---------+---------+---------+
buk2-C1R1  ................................................................................
buk2-C1R2  ................................................................................
buk2-C2R1  ................................................................................
buk2-C3R1  ................................................................................

Majority   GGGGCAGGCTAGGGCGTTGGAGGAAGGAGGTGTTTAATAGAATTTAGAGTGCTATGAATGAT
                  170       180       190       200       210       220
           ---------+---------+---------+---------+---------+---------+--
buk2-C1R1  ...................................G..........................     222 bp
buk2-C1R2  ..............................................................     222 bp
buk2-C2R1  ...C..........................................................     222 bp
buk2-C3R1  ..............................................................     222 bp
```

buk2-C1R1	buk2-C1R2	buk2-C2R1	buk2-C3R1	
***	98.6	98.6	99.1	buk2-C1R1
	***	99.1	99.5	buk2-C1R2
		***	99.5	buk2-C2R1
			***	buk2-C3R1

Similarity: 98.6-99.5

S. bukasovii-3, buk3

```
Majority    CCTTTTTGTCGAAATTCGGTCGTGTAATAGAAAAAATATTATTATTTATTTTTTGTAGAAACGACGTCGTTAGGACAGGT
                     10        20        30        40        50        60        70        80
            ---------+---------+---------+---------+---------+---------+---------+---------+
buk3-C1R1   ................................................................................
buk3-C2R1   ................................................................................
buk3-C3R1   .T..............................................................................
buk3-C5R1   ................................................................................
buk3-C5R2   .T..............................................................................

Majority    GATGGGGGCGTTGGGAGGATGGGCGTGACGGGCGGCGTCATGCGTCGGTGCGCGTGGAGGCTAGGTCGGTGGGGGGCAGG
                     90       100       110       120       130       140       150       160
            ---------+---------+---------+---------+---------+---------+---------+---------+
buk3-C1R1   .............--.................................................................
buk3-C2R1   ................................................................................
buk3-C3R1   ................................................................................
buk3-C5R1   .............--................A..........................TT....................
buk3-C5R2   ...............................................................................A

Majority    CTAGGGCGTTGGGAGGAAGGAGGTGTTTAATAGAATTTAGAGTGCTATGAATGAT
                    170       180       190       200       210
            ---------+---------+---------+---------+---------+-----
buk3-C1R1   .......................................................     213 bp
buk3-C2R1   .......................................................     215 bp
buk3-C3R1   .......................................................     215 bp
buk3-C5R1   .......................................................     213 bp
buk3-C5R2   .......................................................     215 bp
```

buk3-C1R1	buk3-C2R1	buk3-C3R1	buk3-C5R1	buk3-C5R2	
***	99.1	98.6	98.6	98.1	buk3-C1R1
	***	99.5	97.7	99.1	buk3-C2R1
		***	97.2	99.5	buk3-C3R1
			***	96.7	buk3-C5R1
				***	buk3-C5R2

Similarity: 96.7-99.5

S. bukasovii-4, buk4

```
Majority    CCTTTTTGTCGAAATTCGGTCGTGTAATAGAAAAAATATTATTATTTATTTTTTGTAGAAACGACGTCGTTAGGACAGGT
                     10        20        30        40        50        60        70        80
            ---------+---------+---------+---------+---------+---------+---------+---------+
buk4-C1R1   ................................................................................
buk4-C1R2   .........T......................................................................
buk4-C2R1   ................................................................................
buk4-C2R2   .................................T..............................................
buk4-C3R1   ..............A.................................................................
buk4-C5R1   ................................................................................
buk4-C6R2   ................................................................................
buk4-C6R1   ..............G.................................................................

Majority    GATGGGGGCGTTG--AGGATGGGCGTGACGGGCGGCGTCATGCGTCGGTGCGCGTGGAGGCTAGGTCGGTGGGGGGCAGG
                     90       100       110       120       130       140       150       160
            ---------+---------+---------+---------+---------+---------+---------+---------+
buk4-C1R1   .............--.................................................................
buk4-C1R2   .............--.................................................................
buk4-C2R1   .............GG............................................................T....
buk4-C2R2   .............--.................................................................
buk4-C3R1   .............GG.................................................................
buk4-C5R1   ...A..-----------------.........................................................
buk4-C6R2   ......-----------------.........................................................
buk4-C6R1   ...A..-----------------.........................................................

Majority    CTAGGGCGTTGGGAGGAAGGAGGTGTTTAATAGAATTTAGAGTGCTATGAATGAT
                    170       180       190       200       210
            ---------+---------+---------+---------+---------+-----
buk4-C1R1   .......................................................     213 bp
buk4-C1R2   .......................................................     213 bp
buk4-C2R1   .......................................................     215 bp
buk4-C2R2   .......................................................     213 bp
buk4-C3R1   .......................................................     215 bp
buk4-C5R1   ............A..........................................     198 bp
buk4-C6R2   .......................................................     198 bp
buk4-C6R1   ............................G..........................     198 bp
```

buk4-C1R1	buk4-C1R2	buk4-C2R1	buk4-C2R2	buk4-C3R1	buk4-C5R1	buk4-C6R2	buk4-C6R1	
***	99.5	98.6	99.5	98.6	92.1	93.0	91.6	buk4-C1R1
	***	98.1	99.1	98.1	91.6	92.6	91.2	buk4-C1R2
		***	98.1	99.1	90.7	91.6	90.2	buk4-C2R1
			***	98.1	91.6	92.6	91.2	buk4-C2R2
				***	90.7	91.6	90.7	buk4-C3R1
					***	99.1	98.6	buk4-C5R1
						***	98.6	buk4-C6R2
							***	buk4-C6R1

Similarity: 90.2-99.5

S. bukasovii-5, buk5

```
Majority   CCTTTTTGTCGAAATTCGGTCGTGTAATTTGTTTATTATTATTATTTATTTATTGCAGAACCGACGTCGTTAGGAGAGGT
                    10        20        30        40        50        60        70        80
           ---------+---------+---------+---------+---------+---------+---------+---------+
buk5-C1R1  ................T...............................................................
buk5-C1R2  ................................................................................
buk5-C1R3  ................................................................................
buk5-C2R1  ...........................................G....................................
buk5-C7R1  ............................................................................G...

Majority   GATGGGGGCGTTGAGGATGGGCGTGACGGGCGGCGTCATGCGTCGGTGCGCGTGGAGGCTAGGTCGGTGGGGGGCAGGCT
                    90       100       110       120       130       140       150       160
           ---------+---------+---------+---------+---------+---------+---------+---------+
buk5-C1R1  ................................................................................
buk5-C1R2  ................................................................................
buk5-C1R3  ................................................................................
buk5-C2R1  ................................................................................
buk5-C7R1  ................................................................................

Majority   AGGGCGTTGGGAGGAAGGAGGTGTTTAATAGAATTTAGAGTGCTATGAATGAT
                   170       180       190       200       210
           ---------+---------+---------+---------+---------+---
buk5-C1R1  .....................................................    213 bp
buk5-C1R2  .....................................................    213 bp
buk5-C1R3  .....A...............................................    213 bp
buk5-C2R1  .............A.......................................    213 bp
buk5-C7R1  .....................................................    213 bp
```

buk5-C1R1	buk5-C1R2	buk5-C1R3	buk5-C2R1	buk5-C7R1	
***	99.5	99.1	98.6	99.1	buk5-C1R1
	***	99.5	99.1	99.5	buk5-C1R2
		***	98.6	99.1	buk5-C1R3
			***	98.6	buk5-C2R1
				***	buk5-C7R1

Similarity: 98.6-99.5

S. bukasovii-6, buk6

```
Majority       CCTTTTTGTCGAAATTCGGTCGTGTAATAGAAAAAATATTATTATTTATTTTTTGTAGAAACGACGTC---GTTAGGAC-
                       10        20        30        40        50        60        70        80
               ---------+---------+---------+---------+---------+---------+---------+---------+
buk6-C1R1      .T.......T................G..TTT...-......-...AT....C..C.........A.TCGGA..GA...G
buk6-C1R2      ....................................................................---........-
buk6-C1R3      .T.......T...................TTT...-......-...AT....C..C.........A.TCGGA..GA...G
buk6-C9R1      ....................................................................---........-
buk6-C16R1     ................AA..................................................---........-

Majority       ----TA--ACAGGTGATGGGGGCGTTGGGAGGATGGGCGTGACGGGCGGCGTCATGCGTCGGTGCGCGTGGAGGCTAGGT
                       90       100       110       120       130       140       150       160
               ---------+---------+---------+---------+---------+---------+---------+---------+
buk6-C1R1      TCGT..GG...................--.....................................--............
buk6-C1R2      ----..--........................................................................
buk6-C1R3      TCGT..GG...................--.....................................--............
buk6-C9R1      ----..--................................................................A.......
buk6-C16R1     ----..--........................................................................

Majority       CGGTGGGGGGCAGGCTAGGGCGTTGGGAGGAAGGAGGTGTTTAATAGAATTTAGAGTGCTATGAATGAT
                      170       180       190       200       210       220
               ---------+---------+---------+---------+---------+---------+---------
buk6-C1R1      ..........................-..........................................    222 bp
buk6-C1R2      .....................................................................    219 bp
buk6-C1R3      .....................A....-..........................................    222 bp
buk6-C9R1      .....................................................................    219 bp
buk6-C16R1     ..............G......................................................    219 bp
```

buk6-C1R1	buk6-C1R2	buk6-C1R3	buk6-C9R1	buk6-C16R1	
***	86.0	99.1	85.6	84.7	buk6-C1R1
	***	86.0	99.6	98.7	buk6-C1R2
		***	85.6	84.7	buk6-C1R3
			***	98.3	buk6-C9R1
				***	buk6-C16R1

Similarity: 84.7-99.6

S. bukasovii-7, buk7

```
Majority    CCTTTTTGTCGAAATTXGGTCGTGTAATAGAAAAAATATTATTATTTATTTTTTGXAGXAACGACGTCGTTAGGAC----
                     10        20        30        40        50        60        70        80
            ---------+---------+---------+---------+---------+---------+---------+---------+
buk7-C1R1   ................T...........G..........................C..G.......A.........----
buk7-C1R2   ................C.................C.C.G.C..............T..AC................----
buk7-C1R3   ................T...........T......................G...C..G.................----
buk7-C2R1   ..G.............C...................C.G................T..A.................----
buk7-C2R2   ................C......................................T..A.................----
buk7-C2R3   ................T...........T..........................C..G.................----
buk7-C2R4   ................T...........T..........................C..G.......A.........----
buk7-C3R1   ................T...........T..........C.......G.......C..G.................----
buk7-C3R2   ................T...........T..........................C..G.................----
buk7-C3R3   ................C......................................T..A.................----
buk7-C3R4   ................C...................C.G................T..A.................----
buk7-C1R4   ................C......................................T..A.................TAAC

Majority    AGGTGATGGGGGCGTTG--AGGATGGGCGTGACGGGCGGCGTCATGCGTCGGTGCGCGTGGAGGCTAGGTCGGTGGGGGG
                     90       100       110       120       130       140       150       160
            ---------+---------+---------+---------+---------+---------+---------+---------+
buk7-C1R1   .................--.............................................................
buk7-C1R2   .................--.........G...................................................
buk7-C1R3   .................--.............................................................
buk7-C2R1   .................--..............................................G..............
buk7-C2R2   .................--....T........................................................
buk7-C2R3   .................--.............................................................
buk7-C2R4   .................--........................................A....................
buk7-C3R1   .................--..............................G..............................
buk7-C3R2   .................--......A......................................................
buk7-C3R3   .................--.............................................................
buk7-C3R4   ..............G..--...................................................G.........
buk7-C1R4   ..............G..GG.................................G...........................

Majority    CAGGCTAGGGCGTTGGGAGGAAGGAGGTGTTTAATAGAATTTAGAGTGCTATGAATGAT
                    170       180       190       200       210
            ---------+---------+---------+---------+---------+---------
buk7-C1R1   ...........................................................      213 bp
buk7-C1R2   .............................G.............................      213 bp
buk7-C1R3   ...........................................................      213 bp
buk7-C2R1   .................................................A.........      213 bp
buk7-C2R2   ..............................................G............      213 bp
buk7-C2R3   .............................G.............................      213 bp
buk7-C2R4   ...........................................................      213 bp
buk7-C3R1   ...........................................................      213 bp
buk7-C3R2   ...........................................................      213 bp
buk7-C3R3   .........................................................G.      213 bp
buk7-C3R4   A..........................................A...............      213 bp
buk7-C1R4   ...........................................................      219 bp
```

buk7-C1R1	buk7-C1R2	buk7-C1R3	buk7-C2R1	buk7-C2R2	buk7-C2R3	buk7-C2R4	buk7-C3R1	buk7-C3R2	buk7-C3R3	buk7-C3R4	buk7-C1R4	
***	94.5	98.6	95.4	96.8	98.6	99.1	97.7	98.6	97.3	95.0	94.1	buk7-C1R1
	***	94.5	96.3	95.9	95.4	94.1	93.6	94.5	96.3	95.9	93.2	buk7-C1R2
		***	95.4	96.8	99.1	98.6	98.2	99.1	97.3	95.0	94.1	buk7-C1R3
			***	96.8	95.4	95.0	94.5	95.4	97.3	96.8	94.1	buk7-C2R1
				***	96.8	96.3	95.9	96.8	98.6	96.3	95.4	buk7-C2R2
					***	98.6	98.2	99.1	97.3	95.0	94.1	buk7-C2R3
						***	97.7	98.6	96.8	94.5	93.6	buk7-C2R4
							***	98.2	96.3	94.1	93.2	buk7-C3R1
								***	97.3	95.0	94.1	buk7-C3R2
									***	96.8	95.9	buk7-C3R3
										***	94.5	buk7-C3R4
											***	buk7-C1R4

Similarity: 93.2-99.1

S. bulbocastanum-1, blb1

```
Majority CCTTTTTGTAGAAATTCGGTCGCGAAAATATATATTATTATTTATTTGGAGAAACGACGG-GGGGGGGTTTGAAAATGGG
                  10        20        30        40        50        60        70        80
         ---------+---------+---------+---------+---------+---------+---------+---------+
pSbB3.6  ............................................................-...................
pSbB3.2  ............................................................-...................
pSbB3.3  .........C..................................................A...................

Majority CGTGACGGGAGGCGTCATGCGTCGGTGCGTGGAGGCTGCGTCGGTGGGGGGCAGGCTAGGGCGTTGGGAGGAAGGAGGTG
                  90        100       110       120       130       140       150       160
         ---------+---------+---------+---------+---------+---------+---------+---------+
pSbB3.6  ..........................................................................T.....
pSbB3.2  ................................................................................
pSbB3.3  ................................................................................

Majority CTTAATAGAATTTAGAGTGCTATGAATGAC
                  170       180       190
         ---------+---------+---------+
pSbB3.6  ..............................      189 bp
pSbB3.2  ..............................      189 bp
pSbB3.3  ..............................      190 bp
```

pSbB3.6	pSbB3.2	pSbB3.3	
***	99.5	98.4	pSbB3.6
	***	98.9	pSbB3.2
		***	pSbB3.3

Similarity: 98.4-99.5

S. bulbocastanum-2, blb2

```
Majority        CATTTTTGTCGACATTCGGTCGCGAAAAAAAATATTATTATTTATTTGGAGAAACGACCGGGGGGGGTTGAGGATGGGCG
                         10        20        30        40        50        60        70        80
                ---------+---------+---------+---------+---------+---------+---------+---------+
blb2-C2R1       ................................................................................
blb2-C1R1       ........................................................T........T..............
blb2-C2R2       ................................................................................

Majority        TGACGGGGGGCGTCATGCGTCGGTGCGTGGAGGCTGCGTCGGTGGGGGGCAGGCTGGGGCATTGGGAGGAAGGAGGTGTT
                         90       100       110       120       130       140       150       160
                ---------+---------+---------+---------+---------+---------+---------+---------+
blb2-C2R1       ................T...T...........................................................
blb2-C1R1       ................................................................................
blb2-C2R2       ...........................................................................A....

Majority        TAATAGAATTTAGAGTGCTATGAATGAC
                        170       180
                ---------+---------+--------
blb2-C2R1       ............................    188 bp
blb2-C1R1       ............................    188 bp
blb2-C2R2       ............................    188 bp
```

blb2-C2R1	blb2-C1R1	blb2-C2R2	
***	97.9	98.4	blb2-C2R1
	***	98.4	blb2-C1R1
		***	blb2-C2R2

Similarity: 97.9-98.4

S. cajamarquense, cjm

```
Majority CCTTTTTGTCGAAATTCGGTCGTGCAATTGATTTTTATTATTATTTATCTTTTTGCXGAAACGGCGTCGTTAGGACAGG
                  10        20        30        40        50        60        70        80
         ---------+---------+---------+---------+---------+---------+---------+---------+
cjm-C1R1 ........................................................A.......................
cjm-C1R2 ........................................................A.......................
cjm-C3R1 ........................................................C.......................
cjm-C6R1 .T......................................................C.......................

Majority TGATGGGGCGTTGAGGATGGGCGTGACGGGCGGCGTCATGCGTCGGTGCGCGTGGAGGCTAGGTCCGGTGGGGGGCAGG
                  90       100       110       120       130       140       150       160
         ---------+---------+---------+---------+---------+---------+---------+---------+
cjm-C1R1 ................................................................................
cjm-C1R2 ................................................................................
cjm-C3R1 ................................................................................
cjm-C6R1 .......................................................T........................

Majority CTAGGGCGTTGGGAGGAAGGAAGGAAAGGAGGTGTTTAAAAGAATTTAGAGTGCTATGAATGAT
                 170       180       190       200       210       220
         ---------+---------+---------+---------+---------+---------+----
cjm-C1R1 ......................----.....................................C    220 bp
cjm-C1R2 ................................................................    224 bp
cjm-C3R1 ................................................................    224 bp
cjm-C6R1 ...........................T....................................    224 bp
```

cjm-C1R1	cjm-C1R2	cjm-C3R1	cjm-C6R1	
***	99.5	99.1	97.7	cjm-C1R1
	***	99.6	98.2	cjm-C1R2
		***	98.7	cjm-C3R1
			***	cjm-C6R1

Similarity: 97.7-99.6

S. canasense, can

```
Majority  CCTTTTTGTCGAAATTCGGTCGTGTAAT-----------TATTATTTATTTTTTGCAGAAAGTCGGTCGTTAGGACAGGT
                  10        20        30        40        50        60        70        80
          ---------+---------+---------+---------+---------+---------+---------+---------+
can-C1R1  ............................AGAAAAAATAT...........G..........CGAC...............
can-C1R2  ............................-----------.........................................
can-C1R3  ............................-----------...............................G.........
can-C1R4  ............................-----------.........................................

Majority  GATGGGGGCGTTGAGGATGGGCGTGACGGGCGGCGTCATGCGTCGGTGCGCGTGGAGGCTAGGTCGGTGGGGGGCAGGCT
                  90       100       110       120       130       140       150       160
          ---------+---------+---------+---------+---------+---------+---------+---------+
can-C1R1  ................................................................................
can-C1R2  ...............................................T................................
can-C1R3  ................................................................................
can-C1R4  ................................................................................

Majority  AGGGCGTTGGGAGGAAGGAGGTGTTTAATAGAATTTAGAGTGCTATGAATGAT
                 170       180       190       200       210
          ---------+---------+---------+---------+---------+---
can-C1R1  .........................................T...........    213 bp
can-C1R2  ...................................................G.    202 bp
can-C1R3  .......G..........................G..................    202 bp
can-C1R4  .....................................................    202 bp
```

can-C1R1	can-C1R2	can-C1R3	can-C1R4	
***	91.1	90.6	92.0	can-C1R1
	***	97.7	99.1	can-C1R2
		***	98.6	can-C1R3
			***	can-C1R4

Similarity: 90.6-99.1

S. candolleanum, cnd

```
Majority  CCTTTTTGTCGAAATTXGGTCGTGTAATTGAAAAAATATXATTATTTATTTXTTGCAGXAACGACGTCGTTAGGACAGGT
                  10        20        30        40        50        60        70        80
          ---------+---------+---------+---------+---------+---------+---------+---------+
cnd-C1R1  ................C......................AC..........A......A.....................
cnd-C3R1  ................T......................T...........T......G.....................
cnd-C3R2  .T.............AT......................T...........T......G.....................
cnd-C5R1  ................C......................AT..........A......A.....................

Majority  GATGGGGGCGTTGAGGATGGGCGTGACGGGCGGCGTCAXGCGTCGGTGCGCGTGGAGGCTAGGTCGGTGG-GGGGCAGGC
                  90       100       110       120       130       140       150       160
          ---------+---------+---------+---------+---------+---------+---------+---------+
cnd-C1R1  ......................................C...............................-.........
cnd-C3R1  ......................................T...............................T.........
cnd-C3R2  ......................................T...............................G.........
cnd-C5R1  ......................................C......................C........-.........

Majority  TAGGGCGTTGGGXGGAAGGAGGTGTTTAATAGAATTTAGAGTGCTATGAATGAT
                 170       180       190       200       210
          ---------+---------+---------+---------+---------+----
cnd-C1R1  ............T.........................................    213 bp
cnd-C3R1  ............A.........................................    214 bp
cnd-C3R2  ............A.........................................    213 bp
cnd-C5R1  ............T...........C.............................    213 bp
```

cnd-C1R1	cnd-C3R1	cnd-C3R2	cnd-C5R1	
***	96.3	95.3	98.6	cnd-C1R1
	***	98.6	95.3	cnd-C3R1
		***	94.4	cnd-C3R2
			***	cnd-C5R1

Similarity: 94.4-98.6

S. cardiophyllum, cph

```
Majority  CCTTTTTGTCGAAATTCGGTAGTGCGGTTGAAAAAATATATTATTATTTATTTGCAGAAACGACATTCGGATTGAGACGT
                  10        20        30        40        50        60        70        80
          ---------+---------+---------+---------+---------+---------+---------+---------+
cph-C2R1  ...G.............................................................CG.............
cph-C3R1  .....................................................T..........................
cph-C3R2  ..................................G........................................A....
cph-C3R3  ................................................................................
cph-C3R4  ................................................................................

Majority  CGTTAGGACAGGTGATGGGGGCGTTGAGGATGGGCGTGACGGGCGGCGTCACGCGTCGGTGCGTGGAGGCTAGGTCGGTG
                  90       100       110       120       130       140       150       160
          ---------+---------+---------+---------+---------+---------+---------+---------+
cph-C2R1  .........................................................................C......
cph-C3R1  .................T.........................T....................................
cph-C3R2  ................................................................................
cph-C3R3  ................................................................................
cph-C3R4  ...............C................................................................

Majority  GGGGGCAGGCTAGGGCGTTGGGAGGAAGGGGGTGTTTAATAGAATTTAGAGTGCTGTGAATGAC
                 170       180       190       200       210       220
          ---------+---------+---------+---------+---------+---------+----
cph-C2R1  ................................................................    224 bp
cph-C3R1  ...............................................T................    224 bp
cph-C3R2  ................................................T...............    224 bp
cph-C3R3  ................................................................    224 bp
cph-C3R4  ................................G...............................    224 bp
```

cph-C2R1	cph-C3R1	cph-C3R2	cph-C3R3	cph-C3R4	
***	96.4	96.9	98.2	97.3	cph-C2R1
	***	96.9	98.2	97.3	cph-C3R1
		***	98.7	97.8	cph-C3R2
			***	99.1	cph-C3R3
				***	cph-C3R4

Similarity: 96.4-99.1

S. chacoense-1, chc1

DIRECT SEQUENCING - 213 bp

S. chacoense-2, chc2

```
Majority   TCTTTTTGTCGAAATTCGGTCGTGTAATTGAAGAAATATATTTATCTATTTTTTGCAGAAACGGCGCCGTTAGGACAGGT
                    10        20        30        40        50        60        70        80
           ---------+---------+---------+---------+---------+---------+---------+---------+
chc2-C1R1  ...............................................................................C
chc2-C1R2  ...................G...........................................................C
chc2-C1R3  ................................................................................
chc2-C1R4  C...................G..................TA....T....................T...C.........
chc2-C1R5  ............................................................................G..C
chc2-C1R6  .......T..............................G....................GG.....G...G.........
chc2-C1R7  C...................G..................TA....T....................T.............
chc2-C1R9  ................................................C...............................
chc2-C1R10 ...............................................................................C

Majority   GATGGGGGCGTTGAGGATGGGCGTGACGGGCGGCGTCATGCGTCGGTGCGCGTGGAGGCTGGGTCGGTGGGGGGCAGGCT
                  90       100       110       120       130       140       150       160
           ---------+---------+---------+---------+---------+---------+---------+---------+
chc2-C1R1  .............G......................................C...C.............T.........
chc2-C1R2  ........................................................................T.......
chc2-C1R3  ................................................................................
chc2-C1R4  .................G..........................................A...................
chc2-C1R5  ....................................G.G.........................................
chc2-C1R6  .............G........................G.........................................
chc2-C1R7  ...T.............G..........................................A...................
chc2-C1R9  ................................................................................
chc2-C1R10 ....................................................C......................G....

Majority   AGGGCGTTGGGAGGAAGGAGGCGTTTAATAGAATTTAGAGTGCTATGAATGAT
                  170       180       190       200       210
           ---------+---------+---------+---------+---------+---
chc2-C1R1  .................A...T....G............A.............   213 bp
chc2-C1R2  .....................................................   213 bp
chc2-C1R3  .....................................................   213 bp
chc2-C1R4  .....................T...............................   213 bp
chc2-C1R5  .................................CG.CC........C..C...   213 bp
chc2-C1R6  .....................................................   213 bp
chc2-C1R7  .....................T..................G............   213 bp
chc2-C1R9  ................T....................................   213 bp
chc2-C1R10 ...........G..CG..G....G.........G.G.................   213 bp
```

chc2-C1R1	chc2-C1R2	chc2-C1R3	chc2-C1R4	chc2-C1R5	chc2-C1R6	chc2-C1R7	chc2-C1R9	chc2-C1R10	
***	95.3	95.8	92.0	92.0	93.0	91.5	94.8	93.0	chc2-C1R1
	***	98.6	93.9	94.8	94.8	93.4	97.7	94.8	chc2-C1R2
		***	95.3	95.3	96.2	94.8	99.1	95.3	chc2-C1R3
			***	90.6	92.5	98.6	94.4	90.6	chc2-C1R4
				***	92.5	90.1	94.4	92.0	chc2-C1R5
					***	91.5	95.3	91.5	chc2-C1R6
						***	93.9	90.1	chc2-C1R7
							***	94.4	chc2-C1R9
								***	chc2-C1R10

Similarity: 90.1-99.1

S. chaucha, cha

```
Majority   CCTTTTTGTCGAAATTCGGTCGTGTAATAGAAAAAATATTATTATTTATTTTTTGTAGAAACGACGTC---GTTAGGAC-
                    10        20        30        40        50        60        70        80
           ---------+---------+---------+---------+---------+---------+---------+---------+
cha-C1R1   .T.......T................G..TTT...-......-...AT....C..C.........A.TCGGA..GA...G
cha-C7R1   ....................................................................---........-
cha-C7R2   ....................................................................---........-
cha-C7R3   ....................................C.G.............................---........-
cha-C15R1  ....................................................................---........-
cha-C6R1   ....................................................................---........-
cha-C7R4   ...........C........................................................---........-
cha-C13R1  ....................................................................---........-
cha-C13R2  .........................................C..........................---........-
cha-C15R2  ....................................................................---........-

Majority   ----TA--ACAGGTGATGGGGGCGTTGXGAGGATGGGCGTGACGGGCGGCGTCATGCGTCGGTGCGCGTGGAGGCTAGGT
                    90       100       110       120       130       140       150       160
           ---------+---------+---------+---------+---------+---------+---------+---------+
cha-C1R1   TCGT..GG..................--......................................--............
cha-C7R1   ----------................--......T.............................................
cha-C7R2   ----------.........A......--.....................A..............................
cha-C7R3   ----------................--....................................................
cha-C15R1  ----------................--.............................T......................
cha-C6R1   ----..--...................G....................................................
cha-C7R4   ----..--...................G....................................T...............
cha-C13R1  ----..--...................G....................................T...............
cha-C13R2  ----..--...................G....................................................
cha-C15R2  ----..--...................G..................T.................................

Majority   CGGTGGGGGCAGGCTAGGGCGTTGGGAGGAAGGAGGTGTTTAATAGAATTTAGAGTGCTATGAATGAT
                    170       180       190       200       210       220
           ---------+---------+---------+---------+---------+---------+--------
cha-C1R1   ..........................-.........................................    222 bp
cha-C7R1   ..........................A.........................................    213 bp
cha-C7R2   ....................................................................    213 bp
cha-C7R3   ....................................................................    213 bp
cha-C15R1  ................................A...................................    213 bp
cha-C6R1   ....................................................................    219 bp
cha-C7R4   ...........................................................A........    219 bp
cha-C13R1  ...........................................................A........    219 bp
cha-C13R2  ...........................................................A........    219 bp
cha-C15R2  ....................................................................    219 bp
```

cha-C1R1	cha-C7R1	cha-C7R2	cha-C7R3	cha-C15R1	cha-C6R1	cha-C7R4	cha-C13R1	cha-C13R2	cha-C15R2	
***	83.8	83.4	83.4	83.4	86.0	84.7	85.2	85.2	85.6	cha-C1R1
	***	98.3	98.3	98.3	96.5	95.2	95.6	95.6	96.1	cha-C7R1
		***	98.3	98.3	96.5	95.2	95.6	95.6	96.1	cha-C7R2
			***	98.3	96.5	95.2	95.6	95.6	96.1	cha-C7R3
				***	96.5	95.2	95.6	95.6	96.1	cha-C15R1
					***	98.7	99.1	99.1	99.6	cha-C6R1
						***	99.6	98.7	98.3	cha-C7R4
							***	99.1	98.7	cha-C13R1
								***	98.7	cha-C13R2
									***	cha-C15R2

Similarity: 83.4-99.6

S. cheesmaniae, che

```
Majority  CCTTTTTGTTGAAATTTGATCTCGTAATTGAAAAAAAAAATATACTCATTTATTTTTTTTT-GCGGAAAATACGTTCGGA
                   10        20        30        40        50        60        70        80
          ---------+---------+---------+---------+---------+---------+---------+---------+
che-C1R1  .............................................................-..................
che-C1R2  ..................C..........................................-..................
che-C2R1  .......................................-.....................T..................
che-C3R1  ...................................................T.........-..................

Majority  TTGAGGCGTCATTAGGATATGGGATGGTGGCGTCGGGGATGGGCGTGACGGGCGTCGTCGTGCGTCGGTGCGTGGAGGGT
                   90        100       110       120       130       140       150       160
          ---------+---------+---------+---------+---------+---------+---------+---------+
che-C1R1  ................................................................................
che-C1R2  ................................................................................
che-C2R1  ...............................................................A................
che-C3R1  .............................................................C..................

Majority  TTTAAAGCGGGGGGCGGGCTAGGGCGTTGGGAGGAAGGTTGTGTTTAATAGATTTTAGAGTGCAATGAATGAC
                   170       180       190       200       210       220       230
          ---------+---------+---------+---------+---------+---------+---------+---
che-C1R1  .............-...........................................................   231 bp
che-C1R2  .........................................................................   232 bp
che-C2R1  ............................................................A............   232 bp
che-C3R1  .........................................................................   232 bp
```

che-C1R1	che-C1R2	che-C2R1	che-C3R1	
***	99.1	97.9	98.7	che-C1R1
	***	97.9	98.7	che-C1R2
		***	97.4	che-C2R1
			***	che-C3R1

Similarity: 97.4-99.1

S. chilense, chi

```
Majority CCTTTTTGTTGAAATTTGATCTCGTAGTTGAAAAAAA-TATACTCATGTATTTATTTTTGCGTTAAATACGTTCGGATTG
                10        20        30        40        50        60        70        80
         ---------+---------+---------+---------+---------+---------+---------+---------+
chi-C1R1 .....................................A..........................................
chi-C1R2 .....................................-..........................................
chi-C1R4 .....................................-..........................................
chi-C4R3 .....................................-..........................................

Majority AGAGTTCATTAGGATACGGGATGGTGGCGTCGGGGATGGGCGTGACGGGCGTCGTCGTGCGTCGGTGCGTGGAGGGTTTT
                90       100       110       120       130       140       150       160
         ---------+---------+---------+---------+---------+---------+---------+---------+
chi-C1R1 ......................................A.........................................
chi-C1R2 ................................................................................
chi-C1R4 .................................................T..............................
chi-C4R3 ................................................................................

Majority AAAGCGGGGGGCGGGCTAGGGCGTTGGGCGGAAGGTTGTGTTT--AATAGATTTTAGAGTGCAATGAATGAC
               170       180       190       200       210       220       230
         ---------+---------+---------+---------+---------+---------+---------+--
chi-C1R1 ...........................................TT...........................  232 bp
chi-C1R2 ...........................................--...........................  229 bp
chi-C1R4 .........T...........................T.....--...........................  229 bp
chi-C4R3 ...........................................--........C..................  229 bp
```

chi-C1R1	chi-C1R2	chi-C1R4	chi-C4R3	
***	98.3	97.0	97.8	chi-C1R1
	***	98.7	99.6	chi-C1R2
		***	98.3	chi-C1R4
			***	chi-C4R3

Similarity: 97.0-99.6

S. chmielewskii, cml

```
Majority CCTTTTT-GTTGAAATTTGATCTCGTAATTGGAAAAAAAA-TATACTAATTTATTTTTTTTTXGCGGAAAATACGTTCGG
                  10        20        30        40        50        60        70        80
         ---------+---------+---------+---------+---------+---------+---------+---------+
cml-C2R1 .......-................................-...........T.........T.................
cml-C2R2 .......T................................-.....................T.................
cml-C3R1 .......-.......................A........A.....................-.................
cml-C5R1 .......-................................-.....................-.................

Majority ATTGAGACGTCATTAGGATATGGGATGGTGGCGTCGGGGATGGGCGTGACGGGCGTCGTCGTGCGTCGGTGCGTGGAGGG
                  90        100       110       120       130       140       150       160
         ---------+---------+---------+---------+---------+---------+---------+---------+
cml-C2R1 .........................................................A................C.....
cml-C2R2 ................................................................................
cml-C3R1 ..AA..............C.............................................................
cml-C5R1 ................................A.........................................A.....

Majority TTTTAAAGCGGGGGGCGGGCTAGGGCGTTGGGAGGAAGGTTGTGTTTAATAGATTTTAGAGTGCAATGAATGAC
                  170       180       190       200       210       220       230
         ---------+---------+---------+---------+---------+---------+---------+----
cml-C2R1 ........................................A................................. 232 bp
cml-C2R2 ........................A................................................. 233 bp
cml-C3R1 .................................A........................................ 232 bp
cml-C5R1 .A.......A..................................................C............. 231 bp
```

cml-C2R1	cml-C2R2	cml-C3R1	cml-C5R1	
***	97.0	92.2	94.4	cml-C2R1
	***	91.8	94.4	cml-C2R2
		***	93.5	cml-C3R1
			***	cml-C5R1

Similarity: 91.8-97.0

S. chomatophilum, chm

```
Majority CCTTTTGGTCGAAATTCGGTCGAGTGATTGAAAAAATATATTATTATTTTTTGCAGAAACGACATTCGGATTGAGACGTC
                 10        20        30        40        50        60        70        80
         ---------+---------+---------+---------+---------+---------+---------+---------+
chm-C1R1 ........................G.......................................................
chm-C1R2 ......................................AT..C.....C...............................
chm-C1R3 ................................................................................
chm-C2R1 ........................G.......................................................
chm-C2R2 ......................................AT..C.....C...............................

Majority GTTAGGACGGGTGATGGGGGCGTTGAGGATGGGCGTGACGGGCGGCGTCATGCGTCGGTGCGTGGAGGCTAGGCCGGTGG
                 90       100       110       120       130       140       150       160
         ---------+---------+---------+---------+---------+---------+---------+---------+
chm-C1R1 ................................................................................
chm-C1R2 ................................................................................
chm-C1R3 .............................A..................................................
chm-C2R1 .............................A..........................A.......................
chm-C2R2 .C..............................................................................

Majority GGGTCAGGCTAGGGCGTTGGGAGGAAGGAGGTGTTTAATAGAATTTAGAGTGCAATGAATGAT
                170       180       190       200       210       220
         ---------+---------+---------+---------+---------+---------+---
chm-C1R1 ...............................C...............................   223 bp
chm-C2R2 ...............................................................   223 bp
chm-C1R3 ...............................................................   223 bp
chm-C2R1 ...............................................................   223 bp
chm-C2R2 ...............................................................   223 bp
```

chm-C1R1	chm-C1R2	chm-C1R3	chm-C2R1	chm-C2R2	
***	97.3	98.7	98.7	96.9	chm-C1R1
	***	97.8	96.9	99.6	chm-C1R2
		***	99.1	97.3	chm-C1R3
			***	96.4	chm-C2R1
				***	chm-C2R2

Similarity: 96.4-99.6

S. chrysotrichum, chr

```
Majority CCTTTTTTGCGGAAATTCCGTCGTCTATTCTATTCGGCGAATCATAATTTTTTTTGGCGGAAACGACGTTTGCTTGGGAC
                   10        20        30        40        50        60        70        80
         ---------+---------+---------+---------+---------+---------+---------+---------+
chr-C1R1 ................................................................................
chr-C5R1 ..........C...................................T.................................
chr-C5R2 ......................................................G.........................

Majority AGTTGAGGAAGGGCCAGGAACGCGCGCCGTCGCATATGGAGGCTAGGGGCGGTGGTGTGGAGGCTAGGAATGAGGCTTAA
                   90       100       110       120       130       140       150       160
         ---------+---------+---------+---------+---------+---------+---------+---------+
chr-C1R1 ................................................................................
chr-C5R1 .......................................................................A........
chr-C5R2 ..........A..............T...............G..A..........T..............A.........

Majority TAGAATTAAGAGTGCTAGGGATGAT
                  170       180
         ---------+---------+-----
chr-C1R1 .........................         185 bp
chr-C5R1 ...............T.........         185 bp
chr-C5R2 .........................         185 bp
```

chr-C1R1	chr-C5R1	chr-C5R2	
***	97.8	96.2	chr-C1R1
	***	94.1	chr-C5R1
		***	chr-C5R2

Similarity: 94.1-97.8

S. circaeifolium subsp. quimence, crc

```
Majority  CCTTTTTTGTCGAAATTCGGTCGTGTAA-CTAAGAAAATATATTTATTTTATTTGCAGAAACGACATTCGGATTGGGACG
                   10        20        30        40        50        60        70        80
          ---------+---------+---------+---------+---------+---------+---------+---------+
pScB4.1   ............................G...................................................
pScB4.5   ............................-...................................................
crc5S.2   ............................-...................................................
crc5S.3   ........................T...G........................C..........................
crc5S.4   ............................-...................................................
crc5S.5   ............................-...................................................
crc5S.6   ............................G...................................................
crc5S.7   .......................C...--...................................................

Majority  TCGTCGTTAGGAGAGGTGACGGGGGCGTTGAGGATGGGAGTGACGGGCGGCGTCATGCGTTGGTGCGTGGAGGCTAGGTC
                   90       100       110       120       130       140       150       160
          ---------+---------+---------+---------+---------+---------+---------+---------+
pScB4.1   ................................................................................
pScB4.5   ................................................................................
crc5S.2   ................................................................................
crc5S.3   ................................................................................
crc5S.4   ...............................................AA...............................
crc5S.5   ................................................................................
crc5S.6   .................................................................-------........
crc5S.7   .................................................................-------........

Majority  GGTGGGGGGCAGGCTAGGGCGTTTGGGAGGAAGGAGGTGTTTAATAGAATTTAGAGTGCTACGAATGAT
                  170       180       190       200       210       220
          ---------+---------+---------+---------+---------+---------+---------
pScB4.1   .....................................................................        229 bp
pScB4.5   .....................................................................        228 bp
crc5S.2   .....................................................................        228 bp
crc5S.3   .....................................................................        229 bp
crc5S.4   .............................................................T.......        228 bp
crc5S.5   .....................................................................        228 bp
crc5S.6   .............................................................T.......        222 bp
crc5S.7   .............................................................T.......        220 bp
```

pScB4.1	pScB4.5	crc5S.2	crc5S.3	crc5S.4	crc5S.5	crc5S.6	crc5S.7	
***	99.6	99.6	99.1	98.3	99.6	96.5	95.2	pScB4.1
	***	100.0	98.7	98.7	100.0	96.1	95.6	pScB4.5
		***	98.7	98.7	100.0	96.1	95.6	crc5S.2
			***	97.4	98.7	95.6	94.3	crc5S.3
				***	98.7	95.6	95.2	crc5S.4
					***	96.1	95.6	crc5S.5
						***	98.7	crc5S.6
							***	crc5S.7

Similarity: 94.3-100.0

S. clarkiae, cla

```
Majority  CATTTTTTGCCGAGATTTCGTCGTCTGTTCGGCGAATGATTATTTCTTTCGGCGGTTGGGTCGTTTGCTTGGGCAGAGAC
                   10        20        30        40        50        60        70        80
          ---------+---------+---------+---------+---------+---------+---------+---------+
cla-C1R1  ................................................................................
cla-C1R2  ................A...............................................................
cla-C2R1  ................................................................................
cla-C4R1  ................................................................................
cla-C5R1  ..........................A..................T..........A.......................

Majority  GTCGTTAGGACGGCTGAGGAAGGGCGTCGCCGGCGCGTGGAGGCTAGGGCGGTGGTGCGCAGGCTGGGGCGTGGGGGGG-
                   90        100       110       120       130       140       150       160
          ---------+---------+---------+---------+---------+---------+---------+---------+
cla-C1R1  ...............................................................................-
cla-C1R2  ...............................................................................-
cla-C2R1  ...............................................................................G
cla-C4R1  ........A......................................................................-
cla-C5R1  ...............................................................................-

Majority  ATGAATGAGGTTTAATAGAATTAAGAGTGCTAGGAATGAC
                   170       180       190       200
          ---------+---------+---------+---------+
cla-C1R1  .....A..................................   199 bp
cla-C1R2  ........................................   199 bp
cla-C2R1  ........................................   200 bp
cla-C4R1  ........................................   199 bp
cla-C5R1  .G......................................   199 bp
```

cla-C1R1	cla-C1R2	cla-C2R1	cla-C4R1	cla-C5R1	
***	99.0	99.0	99.0	97.5	cla-C1R1
	***	99.0	99.0	97.5	cla-C1R2
		***	99.0	97.5	cla-C2R1
			***	97.5	cla-C4R1
				***	cla-C5R1

Similarity: 97.5-99.0

S. cleistogamum, cle

```
Majority   CTTTTTTCGXXXXXXXXXXXTCGTCTATTCGXCCGATGACXATTTTTTTTTGCXGXAGGGCCXTTTGCTTGGGCAGAGXC
                    10        20        30        40        50        60        70        80
           ---------+---------+---------+---------+---------+---------+---------+---------+
cle-C1R1   .........CCGAAATTTCG...........A....C...G............G.C......T...............T.
cle-C4R1   .........CCGAAATTTCG...........A........G............G.C......T...............T.
cle-C1R2   .........-----------...........G........T............T.T......G...A...........A.
cle-C8R1   .........-----------...........G........T............T.T......G...............A.

Majority   GTCGXTAGGACGGCTGAGGAXGG-XGTCGGCGGCGCGCGGAGGCTAGGGCG-ACGGGGTGCXGTAATGAGGTTTAATAGA
                    90       100       110       120       130       140       150       160
           ---------+---------+---------+---------+---------+---------+---------+---------+
cle-C1R1   ....C........T......G..-A...A....................T.-.........T..................
cle-C4R1   ....C...............G..-C..........................-.........A.G................
cle-C1R2   ....T...............A..GC..........................T.T.......A.A................
cle-C8R1   ....T.......A.......A..TA....A...............CT....-.........T..................

Majority   ATTAAGAGTGCTACGATTGAC
                   170       180
           ---------+---------+-
cle-C1R1   .....................    179 bp
cle-C4R1   .............G.......    179 bp
cle-C1R2   .....................    170 bp
cle-C8R1   .....................    169 bp
```

cle-C1R1	cle-C4R1	cle-C1R2	cle-C8R1	
***	95.0	78.8	84.0	cle-C1R1
	***	82.9	83.4	cle-C4R1
		***	89.9	cle-C1R2
			***	cle-C8R1

Similarity: 95.0-78.8

S. clivorum, cli

```
Majority  TTTTTCCGATCGAAATTCTTCATAATTTCGTGTATTTGATAATATTTATTTATATTTTTTTGCGGAAACGTCATTCTGAT
                   10        20        30        40        50        60        70        80
          ---------+---------+---------+---------+---------+---------+---------+---------+
cli-C1R1  ................................................................................
cli-C2R1  ................................................................................
cli-C3R1  ........................................C.......................................
cli-C4R1  ................................................................................

Majority  CGAGACGTCGTTAGGACCTGTGAGGGGGGCGTGACAGGCGGCGTTGTGTGGAGGGTCGGTCGGTAGGGGTGCAGGATAGG
                   90        100       110       120       130       140       150       160
          ---------+---------+---------+---------+---------+---------+---------+---------+
cli-C1R1  ................................................................................
cli-C2R1  .............................................................................G..
cli-C3R1  ................................................................................
cli-C4R1  ................................................................................

Majority  GCGTTGGTAGGAAGGAGGTGTTTAATAAGATTAGAATGCAATTACTGAT
                   170       180       190       200
          ---------+---------+---------+---------+---------
cli-C1R1  .................................................    209 bp
cli-C2R1  .................................................    209 bp
cli-C3R1  ..T..............................................    209 bp
cli-C4R1  ..................G..............................    209 bp
```

cli-C1R1	cli-C2R1	cli-C3R1	cli-C4R1	
***	99.5	99.0	99.5	cli-C1R1
	***	98.6	99.0	cli-C2R1
		***	98.6	cli-C3R1
			***	cli-C4R1

Similarity: 98.6-99.5

S. cochoae, coc

```
Majority CCTTTTTGCCGAAATTCGGCATGATTCGGTGTGTCTGACGGCCGGTGCATTTTTTCCGCAGAAACGGGCGTGCCG---GG
                  10        20        30        40        50        60        70        80
         ---------+---------+---------+---------+---------+---------+---------+---------+
coc-C1R1 ...........................................................................---..
coc-C1R2 ...........................................................................---..
coc-C3R1 ...........................................................................CCG..
coc-C9R1 .................A...................C.....................................---..
coc-C9R2 .................A.........................................................---..

Majority GGTCGCAACGCGCCGGTGCGAGGGGGCTAGGGCGGTGGGGCGCTGAGGCTTAATAGAATTTGGAGCGCTAGGGATGAC
                  90       100       110       120       130       140       150
         ---------+---------+---------+---------+---------+---------+---------+--------
coc-C1R1 ....................G.........................................................155bp
coc-C1R2 ..............................................................................155bp
coc-C3R1 ..............................................................................158bp
coc-C9R1 ................................T.............................................155bp
coc-C9R2 ..............................................................................155bp
```

coc-C1R1	coc-C1R2	coc-C3R1	coc-C9R1	coc-C9R2	
***	99.4	97.5	97.5	98.7	coc-C1R1
	***	98.1	98.1	99.4	coc-C1R2
		***	96.2	97.5	coc-C3R1
			***	98.7	coc-C9R1
				***	coc-C9R2

Similarity: 96.2-99.4

S. commersonii-1, cmm1

DIRECT SEQUENCING - 224 bp

S. commersonii-2, cmm2

```
Majority    CCTTTTTTTGTCGAAATTCGGTCGTGCAATTGAAAAAATGTATTTATTTATTTTTTGCAGAAACXACGTCGTTAGGACAG
                    10        20        30        40        50        60        70        80
            ---------+---------+---------+---------+---------+---------+---------+---------+
cmm2-C1R1   ................................................................G...............
cmm2-C1R2   ................................................................T...............
cmm2-C2R1   ........-.......................................................G...............
cmm2-C3R1   ............A...................................................T...............

Majority    GACAGGTGATGXGGGCGTTGAGGATGGGCGTGACGGGCGGCGTCATGCGTCGGTGCGCGTGGAGGCTGGGTCGGTGGGGG
                    90       100       110       120       130       140       150       160
            ---------+---------+---------+---------+---------+---------+---------+---------+
cmm2-C1R1   ...........G....................................................................
cmm2-C1R2   ..........AT....................................................................
cmm2-C2R1   ...........G.............................A......................................
cmm2-C3R1   ....C......T....................................................................

Majority    GCAGGCTAGGGCGTTGGGAGGAAGGAGGTGTTTAATAGAATTTAGAGTGCTATGAATGAT
                   170       180       190       200       210       220
            ---------+---------+---------+---------+---------+---------+
cmm2-C1R1   ............................................................      220 bp
cmm2-C1R2   ..........................T.................................      220 bp
cmm2-C2R1   ............................................................      219 bp
cmm2-C3R1   ............................................................      220 bp
```

cmm2-C1R1	cmm2-C1R2	cmm2-C2R1	cmm2-C3R1	
***	98.2	99.1	98.2	cmm2-C1R1
	***	97.3	98.2	cmm2-C1R2
		***	97.3	cmm2-C2R1
			***	cmm2-C3R1

Similarity: 97.3-99.1

S. corneliomuelleri, crm

```
Majority  CCTTTTTGTTGAAATTTGATCTCGTAGTTGAAAAAAATATACTCATGTATTTATTTTTGCGTTAAATACGTTCGGATTGA
                   10        20        30        40        50        60        70        80
          ---------+---------+---------+---------+---------+---------+---------+---------+
crm-C1R1  .............-..................................................................
crm-C2R1  ....................................-...........................................
crm-C3R1  ................................................................T...............

Majority  GAGTTCATTAGGATATGGGATGGTGGCGTCGGGGATGGGCGTGACGGGCGTCGTCGXGCGTCGXTGCGTGGAGGGTTTTA
                   90        100       110       120       130       140       150       160
          ---------+---------+---------+---------+---------+---------+---------+---------+
crm-C1R1  ...................................................-.A..G......T................
crm-C2R1  ........................................................--------................
crm-C3R1  ...............C........................CA..............T......G................

Majority  AAGCGGGGGGCGGGCTAGGGCGTTGGGCGGAAGGTTGTGTTTAATAGATTTTAGAGTGCAATGAATGAC
          ---------+---------+---------+---------+---------+---------+---------
                   170       180       190       200       210       220
          ---------+---------+---------+---------+---------+---------+---------
crm-C1R1  .....................................................................    227 bp
crm-C2R1  ...............................G----------...........................    210 bp
crm-C3R1  .....................................................................    229 bp
```

crm-C1R1	crm-C2R1	crm-C3R1	
***	90.0	96.1	crm-C1R1
	***	89.5	crm-C2R1
		***	crm-C3R1

Similarity: 89.5-96.1

S. crinitum, cri

```
Majority CCTTTTTTTXCCCGTCGAGCATTCGGCGAATCATTCATTATTATTATTATTTTTXGGCGGAAACGACATTTCGTTAGGGC
                  10        20        30        40        50        60        70        80
         ---------+---------+---------+---------+---------+---------+---------+---------+
pScri1   .........A............................................T.........................
pScri2   .........-............................................-.........................

Majority AGCCGAGGAAGCGCGCGCCGGTGCGTGGAGGCCAGGGGCGGTGGTGTGCCGGCTAGGGCGTCGGGAGGAATGAGGTTTAA
                  90        100       110       120       130       140       150       160
         ---------+---------+---------+---------+---------+---------+---------+---------+
pScri1   ................................................................................
pScri2   ................................................................................

Majority TAGAATTTAGAGCGCTAGGAAXGAC
                  170       180
         ---------+---------+-----
pScri1   .....................A...         185 bp
pScri2   .....................G...         183 bp
```

cri1	cri2	
***	98.4	cri1
	***	cri2

Similarity: 98.4

S. curtilobum, cur

```
Majority  CCTTTTTGTCGAAATTCGGTCGTGTAATAGAAAAAATATTATTATTTAT-TTTTTGXAGAA-------------ACGACG
                   10        20        30        40        50        60        70        80
          ---------+---------+---------+---------+---------+---------+---------+---------+
cur-C1R1  .................................................-......T....-------------......
cur-C2R1  .................................................-......T....-------------......
cur-C3R1  .T..................................A............-......T....-------------......
cur-C5R1  .................................................-......T....-------------......
cur-C5R2  .................................................-......C....-------------......
cur-C10R1 ........................C...T..TTTTT.............C......C....-------------...G..
cur-C16R1 ........C..........C......G.T....C.....-......-..G......C....CGACATTCGGATTGA....
cur-C22R2 .................................................-......C....-------------......

Majority  TCGTTAGGAC----AGGTGATGGGGGCGTTXXGAGGATGGGCGTGACGGGCGGCGTCATGCGTCGGTGCGCGTGGAGGCT
                   90       100       110       120       130       140       150       160
          ---------+---------+---------+---------+---------+---------+---------+---------+
cur-C1R1  ..........TAAC................GG................................................
cur-C2R1  ..........TAAC................GG................................................
cur-C3R1  ..........----...............GGG................................................
cur-C5R1  ..........TAAC.............T..GG................................................
cur-C5R2  ..........----................--................................................
cur-C10R1 ..........----................--.G..............................................
cur-C16R1 ..........----................--.........G............................--........
cur-C22R2 ..........----................--................................................

Majority  AGGTC-GGTGGGGGGCAGGCTAGGGCGTTGGGAGGAAGGA-----GGTGTTTAATAGAATTTAGAGTGCTATGAATGAT
                  170       180       190       200       210       220       230
          ---------+---------+---------+---------+---------+---------+---------+---------
cur-C1R1  .....-..................................-----..................................219bp
cur-C2R1  .....-..............................G...-----..................................219bp
cur-C3R1  .....-......................G...........-----........................A.........215bp
cur-C5R1  .....-..................................-----..................................219bp
cur-C5R2  .....-..................................-----..................................213bp
cur-C10R1 .....C..................................AAGGA.........A........................220bp
cur-C16R1 .....-..................................-----....C.............................223bp
cur-C22R2 .....-.A................................-----..................................213bp
```

cur-C1R1	cur-C2R1	cur-C3R1	cur-C5R1	cur-C5R2	cur-C10R1	cur-C16R1	cur-C22R2	
***	99.6	96.2	99.6	97.1	90.0	85.8	96.7	cur-C1R1
	***	95.8	99.2	96.7	89.5	85.4	96.2	cur-C2R1
		***	95.8	96.7	89.5	85.4	96.2	cur-C3R1
			***	96.7	89.5	85.4	96.2	cur-C5R1
				***	92.9	88.7	99.6	cur-C5R2
					***	83.3	92.5	cur-C10R1
						***	88.3	cur-C16R1
							***	cur-C22R2

Similarity: 83.3-99.6

S. demissum, dem

```
Majority  CCTTTTTGTCGAAATTCGGTCGTGTAATAGAAAAAATATTATTATTTATTTTTTGTAGAAACGACGTCGTTAGGACTAAC
                   10        20        30        40        50        60        70        80
          ---------+---------+---------+---------+---------+---------+---------+---------+
dem_5S.4  ................................................................................
dem_5S.5  ................................................................................
dem_5S.6  ................................................................................

Majority  AGGTGATGGGGGCGTTGGGAGGATGGGCGTGACGGGCGGCGTCATGCGTCGGTGCGCGTGGAGGCTAGGTCGGTGGGGGG
                   90       100       110       120       130       140       150       160
          ---------+---------+---------+---------+---------+---------+---------+---------+
dem_5S.4  .......................................................A........................
dem_5S.5  ....A...........................................................................
dem_5S.6  ................................................................................

Majority  CAGGCTAGGGCGTTGGGAGGAAGGAGGTGTTTAATAGAATTTAGAGTGCTATGAATGAT
                  170       180       190       200       210
          ---------+---------+---------+---------+---------+---------
dem_5S.4  ...........................................................     219 bp
dem_5S.5  .................................................A.........     219 bp
dem_5S.6  ...........................................................     219 bp
```

dem_5S.4	dem_5S.5	dem_5S.6	
***	98.6	99.5	dem_5S.4
	***	99.1	dem_5S.5
		***	dem_5S.6

Similarity: 98.6-99.5

S. dimorphandrum, dim

```
Majority CTTTTTGTCGAAATTTCGGCACGGTTTCGCCTATTTGATAATATATGTTTTGGCAGAAACGGGATTTGGGCCGATACGCC
                  10        20        30        40        50        60        70        80
         ---------+---------+---------+---------+---------+---------+---------+---------+
dim-C1R1 ................................................................................
dim-C2R1 ...............................C................................................
dim-C1R2 ................................................................................
dim-C4R1 ...................A............................................................

Majority GCGAGGAAGGTGATGXXXXXXXXXXXXXCGGGCGTGCAGGCTAGGGCGGTGGGAGGAAAGAGGTTTAATAGAATTTAGAG
                       90       100       110       120       130       140       150       160
             ---------+---------+---------+---------+---------+---------+---------+---------+
dim-C1R1 ...............-------------....................................................
dim-C2R1 ...............GGGGCGCGCAGGA....................................................
dim-C1R2 ...............GGGGCGCGCAGGA....................................................
dim-C4R1 ...............-------------....................................................

Majority TGCTAGGAATGAC
                  170
         ---------+---
dim-C1R1 .............  160 bp
dim-C2R1 .............  173 bp
dim-C1R2 .............  173 bp
dim-C4R1 ...........G.  160 bp
```

dim-C1R1	dim-C2R1	dim-C1R2	dim-C4R1	
***	91.9	92.5	98.8	dim-C1R1
	***	99.4	90.8	dim-C2R1
		***	91.3	dim-C1R2
			***	dim-C4R1

Similarity: 90.8-99.4

S. diversiflorum, div

```
Majority  CTTTTTTTGCCGAAATTTCGTCGTCCAXTCGGCGAGTGATTATTTTTTCCGGCGGTCGGGTCGTTTGCTTGGGCAGAGAC
                   10        20        30        40        50        60        70        80
          ---------+---------+---------+---------+---------+---------+---------+---------+
div-C2R1  ...........................C....................................................
div-C3R1  AC.........................C....................................................
div-C3R2  ...........................T....................................................
div-C3R3  ...........................T...............C..............................G.....

Majority  GTCGTTAGGACGGTTGAGGAAGGGCGTCGCCGGCGCGTGGAGGCTAGGGCGGTGGTGCGCAGGCTAGGGCGTGGGGGGAG
                   90        100       110       120       130       140       150       160
          ---------+---------+---------+---------+---------+---------+---------+---------+
div-C2R1  ..............................A...........................................T.....
div-C3R1  ................................................................................
div-C3R2  ................................................................................
div-C3R3  ................................................................................

Majority  GAATGAGGTTTAAXAGAATTAAGAGTGCTGGAAATGAT
                   170       180       190
          ---------+---------+---------+--------
div-C2R1  .............T........................    198 bp
div-C3R1  .............C........................    198 bp
div-C3R2  .............T........................    198 bp
div-C3R3  .............C........................    198 bp
```

div-C2R1	div-C3R1	div-C3R2	div-C3R3	
***	97.5	98.5	97.0	div-C2R1
	***	98.0	97.5	div-C3R1
		***	98.5	div-C3R2
			***	div-C3R3

Similarity: 97.0-98.5

S. dulcamara, dulc

```
Majority        CCTTTTTGTCGGAATTCGGCACGGTTTCGTCTACTCGACAACAAGTATTTTTTT-GCGGACACGACATTCGGGGCCTAGA
                        10        20        30        40        50        60        70        80
                ---------+---------+---------+---------+---------+---------+---------+---------+
dulc_5S.1       ......................................................-.........................
dulc_5S.2       ......................................................-.........................
dulc_5S.3       ......................................................T.........................
dulc_5S.4       ......................................................-.........................
dulc_5S.5       ......................................................-.........................

Majority        CGCCGTTAGGACGTGCGATGGAGGCGCTTAGGACGGGCGTGACAGGCGGCAGGGTGCGTCGG-TGCGT--GGAGGGCAGG
                        90       100       110       120       130       140       150       160
                ---------+---------+---------+---------+---------+---------+---------+---------+
dulc_5S.1       ..............................................................-.....--..........
dulc_5S.2       ..............................................................-.....--..........
dulc_5S.3       ..............................................................-.....--..........
dulc_5S.4       ..............................................................G.....TT..........
dulc_5S.5       ..............................................................-.....--..........

Majority        GC-GCATGCCGGGGCGTT-GGGAGGGGAAGGGGGTGTTTAATAGAATTTAGAGCGCTAGGAATGAT
                       170       180       190       200       210       220
                ---------+---------+---------+---------+---------+---------+------
dulc_5S.1       ..-...............-....................................-..T.......    219 bp
dulc_5S.2       ..-...............-...............................................    220 bp
dulc_5S.3       ..-...............T......................................G........    222 bp
dulc_5S.4       ..T...............-..................................T....T.......    222 bp
dulc_5S.5       ..-...............T...............................................    221 bp
```

dulc_5S.1	dulc_5S.2	dulc_5S.3	dulc_5S.4	dulc_5S.5	
***	99.1	97.8	97.3	98.7	dulc_5S.1
	***	98.7	97.3	99.6	dulc_5S.2
		***	96.0	99.1	dulc_5S.3
			***	96.9	dulc_5S.4
				***	dulc_5S.5

Similarity: 96.0-99.6

S. ehrenbergii, ehr

```
Majority  CCTTTTTGTCGAAATTCGGTCGTGTAATTGAAAAAGTACATTATTATTTATTTGCAGAAACGACATTGGGATTGAGACGT
                   10        20        30        40        50        60        70        80
          ---------+---------+---------+---------+---------+---------+---------+---------+
ehr-C1R1  ...........................................C....................................
ehr-C2R1  ................................................................................
ehr-C3R1  .T..............................................................................
ehr-C4R1  ...................A............................................................
ehr-C3R2  ...................................T............................................

Majority  CGTTAGGACAGGTGATGGGGGGCGTTGAGGAGGGGCGTGACGGGXGGCGTCGTGCGTCGGTG-----AGGCTAGGTCGGT
                   90       100       110       120       130       140       150       160
          ---------+---------+---------+---------+---------+---------+---------+---------+
ehr-C1R1  ...........................................A-----------------------.............
ehr-C2R1  ...........................................A-----------------------.............
ehr-C3R1  .....................-..A...................A.................------------------
ehr-C4R1  ......................A.....................C.................------------------
ehr-C3R2  ............................................C.................CATGG.............

Majority  GGGGGGCGGGCTAGGGCGTTGGGAGGAAGGAGGTGTTTAATAGAATTTGGAGTGCTATGAATGAC
                  170       180       190       200       210       220
          ---------+---------+---------+---------+---------+---------+-----
ehr-C1R1  .................................................................    202 bp
ehr-C2R1  .......................G.........................................    202 bp
ehr-C3R1  -................................................................    205 bp
ehr-C4R1  -...............................C...............A................    206 bp
ehr-C3R2  .................................................................    225 bp
```

ehr-C1R1	ehr-C2R1	ehr-C3R1	ehr-C4R1	ehr-C3R2	
***	99.1	83.6	83.1	88.4	ehr-C1R1
	***	83.6	83.1	88.4	ehr-C2R1
		***	96.4	89.3	ehr-C3R1
			***	89.3	ehr-C4R1
				***	ehr-C3R2

Similarity: 83.1-99.1

S. elatius, ela

```
Majority  CTTTTTTTGCCGAAATTTCGTCGTCTATTCGGCGAATGATTATTTTTTTGGGCGGTTGGGTCGTTTGCTCGGGCAGAGAC
                   10        20        30        40        50        60        70        80
          ---------+---------+---------+---------+---------+---------+---------+---------+
ela-C1R1  ................................................................................
ela-C1R2  ...........................AAA..................................................
ela-C2R1  .C........G.........................C............T..................C...........
ela-C3R1  .........................................-......................................

Majority  GTCGTTAGGACGGTTGAGGAAGGGCGTCGCCGGGGCGTGGAGGCTAGGGCGGTGGTGTGCAGGTTAGGGCGTGGGGGGGA
                   90       100       110       120       130       140       150       160
          ---------+---------+---------+---------+---------+---------+---------+---------+
ela-C1R1  ................................................................................
ela-C1R2  ...............................................................................G
ela-C2R1  .................................C...............A.........................T....
ela-C3R1  ................................................................................

Majority  GGAATGAGGTTTAATAGAATTAAGAGTGCTAGGAATGAT
                  170       180       190
          ---------+---------+---------+---------
ela-C1R1  .......................................     199 bp
ela-C1R2  .......................................     199 bp
ela-C2R1  .............................C.........     199 bp
ela-C3R1  ..............................G........     198 bp
```

ela-C1R1	ela-C1R2	ela-C2R1	ela-C3R1	
***	98.0	95.5	99.0	ela-C1R1
	***	93.5	97.0	ela-C1R2
		***	94.5	ela-C2R1
			***	ela-C3R1

Similarity: 93.5-99.0

S. erianthum, eri

```
Majority  CCTTTTTGTCGAAATTCGGCACAACTTCGTCTATTTGATGATATTTTTTTCCCGCGGAAACGGCATTTGGTCCGGGAGGT
                   10        20        30        40        50        60        70        80
          ---------+---------+---------+---------+---------+---------+---------+---------+
eri-C1R1  ................................................................................
eri-C1R2  .................................................C..-...........................
eri-C1R3  .......................C........................................................
eri-C1R4  .................................................C..............................
eri-C1R5  .T..................................................A...........................
eri-C1R6  .................................................A..............................

Majority  CGTTAGGACAGGCGATGAGGACGGGCGTGCCAGGCGTCACCACGCGCCGGTGCGTGTAGGATAGGGCGGTGGGGTGCAGG
                   90       100       110       120       130       140       150       160
          ---------+---------+---------+---------+---------+---------+---------+---------+
eri-C1R1  ................................................................................
eri-C1R2  ....................................................G...C.......................
eri-C1R3  .............................................T...............................T..
eri-C1R4  ......T.........................................................................
eri-C1R5  ............................................................................T...
eri-C1R6  .........................T.....T........................................A.......

Majority  CTAGGGCGTTGGGAGGAATGTGGTTTAATAGAATTTAGAGTGCAAGGAATGAT
                  170       180       190       200       210
          ---------+---------+---------+---------+---------+---
eri-C1R1  ...T.................................................    213 bp
eri-C1R2  ......................................G..............    212 bp
eri-C1R3  ..........T...............T..........................    213 bp
eri-C1R4  ........................G............................    213 bp
eri-C1R5  ............A.................................T.....C    213 bp
eri-C1R6  .....................................................    213 bp
```

eri-C1R1	eri-C1R2	eri-C1R3	eri-C1R4	eri-C1R5	eri-C1R6	
***	97.2	97.2	98.1	96.7	97.7	eri-C1R1
	***	95.3	97.2	95.3	96.2	eri-C1R2
		***	96.2	94.8	95.8	eri-C1R3
			***	95.8	97.2	eri-C1R4
				***	95.3	eri-C1R5
					***	eri-C1R6

Similarity: 94.8-98.1

S. esuriale, esu

```
Majority   CTTTTTTTTGCCTGCCGGAATTCGGTGGTGTCGTTAGGTCGGTTGATTATGCCTTTAGGCGTTTGCTTGGGCAGGGACGT
                    10        20        30        40        50        60        70        80
           ---------+---------+---------+---------+---------+---------+---------+---------+
esu-C1R1   ................................................................................
esu-C3R1   .......G........................................................................
esu-C4R1   ................................................................................
esu-C5R1   ...............T................................................................

Majority   CGTTAGGACGGCTGAGGAAGGGCGTCGCCGGCGGGTGGAGGCTAGGGCGGTGGCGTTGGGGGGG-AGGACTGAGGTTTAA
                    90       100       110       120       130       140       150       160
           ---------+---------+---------+---------+---------+---------+---------+---------+
esu-C1R1   ................................................................G...............
esu-C3R1   ................A...............................................-...............
esu-C4R1   ......C...A.....................................................-...............
esu-C5R1   ................................................................-...........C...

Majority   TAGAATTAAGAGTGXTAGGAXTGAX
                   170       180
           ---------+---------+-----
esu-C1R1   ..............C.....A...C    185 bp
esu-C3R1   ..............A.....T...T    184 bp
esu-C4R1   ..............A.....T...T    184 bp
esu-C5R1   ..............C.....A...C    184 bp
```

esu-C1R1	esu-C3R1	esu-C4R1	esu-C5R1	
***	96.8	96.8	98.4	esu-C1R1
	***	97.8	96.2	esu-C3R1
		***	96.2	esu-C4R1
			***	esu-C5R1

Similarity: 96.2-98.4

S. etuberosum, etb

```
Majority  ACATTTTGTAGAAATTCGGTCGTGTATTTGAAAAATATATATATATTTTTTGCAGAAACGACATTCGGATTGATCGGTCG
                   10        20        30        40        50        60        70        80
          ---------+---------+---------+---------+---------+---------+---------+---------+
etb-C1R1  ..T.....................................................A....................
etb-C1R2  ..T.............................................................................
etb-C1R3  .............................A.........................G.T......................
etb-C1R4  .........G......................................................................
etb-C1R5  .................................G.....................G........................
etb-C1R6  ..T.......A.....................................................................
etb-C1R7  .......................................................G........................

Majority  TGTAGGACAGGTGACGGGGGCGCTTAGGATGGGCGTGAAAGGCGGCGTCAAGCGTCGGTGCGTGGAGGCTAGGTCGGTGG
                   90       100       110       120       130       140       150       160
          ---------+---------+---------+---------+---------+---------+---------+---------+
etb-C1R1  .....................T..........................................................
etb-C1R2  ................................................................................
etb-C1R3  ...............T.......A.............................AAA........................
etb-C1R4  .......................G........................................................
etb-C1R5  ................................................................................
etb-C1R6  ...........................................................................A....
etb-C1R7  .................................................................G..............

Majority  GGGGCAGGCTAGGGCGTTGGGAGGAAGGAGGTGTTTAATAGAATTTAGAGTGCTATGAATGAT
                  170       180       190       200       210       220
          ---------+---------+---------+---------+---------+---------+---
etb-C1R1  ...............................................................   223 bp
etb-C1R2  ...............................................................   223 bp
etb-C1R3  ......A........................................................   223 bp
etb-C1R4  ...............................................................   223 bp
etb-C1R5  A..-...........................................................   222 bp
etb-C1R6  ...............................................................   223 bp
etb-C1R7  ...............................................C...............   223 bp
```

etb-C1R1	etb-C1R2	etb-C1R3	etb-C1R4	etb-C1R5	etb-C1R6	etb-C1R7	
***	99.1	94.6	97.8	96.9	98.2	97.3	etb-C1R1
	***	95.5	98.7	97.8	99.1	98.2	etb-C1R2
		***	95.5	95.1	94.6	95.5	etb-C1R3
			***	97.3	97.8	97.8	etb-C1R4
				***	96.9	97.8	etb-C1R5
					***	97.3	etb-C1R6
						***	etb-C1R7

Similarity: 94.6-99.1

S. ferocissimum, fer

```
Majority CATTTTTTGCCGAAATTTCGTCGTCTATTCGGCGAATGATTATTTTTTTTGGCGGTTGGGTCGTTTGCTTTGGCAGAGAC
                  10        20        30        40        50        60        70        80
         ---------+---------+---------+---------+---------+---------+---------+---------+
fer-C1R1 ......................................................................G.........
fer-C2R1 ...................A............................................................
fer-C2R2 ................................................................................

Majority GTCGTTAGGACGGTCGAGTAAGGGCGTCGCCGGCGCGTGGAGGCTAGGGCATGTAATGGTGTGCAGGTTTAGGGCGTGGT
                  90       100       110       120       130       140       150       160
         ---------+---------+---------+---------+---------+---------+---------+---------+
fer-C1R1 ................................................................................
fer-C2R1 ................................................................................
fer-C2R2 ................................................................................

Majority GGGAGGAATGAGGTTTAATAGAATTAAGAGTGCTAGGAATGAC
                 170       180       190       200
         ---------+---------+---------+---------+---
fer-C1R1 ........................................... 203 bp
fer-C2R1 ........................................A.. 203 bp
fer-C2R2 .........................................C. 203 bp
```

fer-C1R1	fer-C2R1	fer-C2R2	
***	98.5	99.0	fer-C1R1
	***	98.5	fer-C2R1
		***	fer-C2R2

Similarity: 98.5-99.0

S. galapagense, gal

ONE RIBOTYPE - 233 bp

S. gourlayi-1, grl1

DIRECT SEQUENCING - 213 bp

S. gourlayi-2, grl2

```
Majority     CCTTTTTGTCGAAAATTTGGTCGTGTAATTGAAAAAATATTATTATTTATTTTTTGCAGGAACGACGTCGTTAGGACAGG
                      10        20        30        40        50        60        70        80
             ---------+---------+---------+---------+---------+---------+---------+---------+
grl2-C1R1    ................................................................................
grl2-C1R2    ................................................................................
grl2-C1R3    ..............-.............................G....G..........................G...

Majority     TGATGGGGGCGTTGAGGATGGGCGTGACGGGCGGCGTCATGCGTCGGTGCGCGTGGAGGCTAGGTCGGTGGGGGGCAGGC
                      90       100       110       120       130       140       150       160
             ---------+---------+---------+---------+---------+---------+---------+---------+
grl2-C1R1    ................................................................................
grl2-C1R2    ................................................................................
grl2-C1R3    ................................................................................

Majority     TGGGGCGTTGGGAGGAAGGAGGTGTTTAATAGAATTTAGAGTGCTATGAATGAT
                     170       180       190       200       210
             ---------+---------+---------+---------+---------+----
grl2-C1R1    ......................................................    214 bp
grl2-C1R2    C.....................................................    214 bp
grl2-C1R3    ..............................................G.......    213 bp
```

grl2-C1R1	grl2-C1R2	grl2-C1R3	
***	99.5	97.7	grl2-C1R1
	***	97.2	grl2-C1R2
		***	grl2-C1R3

Similarity: 97.2-99.5

S. guamense, gua

```
Majority       CCTTTTTTGCGGAAATTCCGTCGTCTATTCTATTCGGCGAATCATAATTTTTTTTGGCGGAAACGACGTTTGCTTGGGAC
                        10        20        30        40        50        60        70        80
               ---------+---------+---------+---------+---------+---------+---------+---------+
pSgua-5S1      ..A.........................................................................A...
pSgua-5S2      ..........C..................................T..................................
pSgua-5S3      .......................................................A........................
pSgua-5S4      ......................................................G.........................

Majority       AGTTGAGGAAGGGCCAGGAACGCGCGXCGTCGCATATGGAGGCTAGGGGCGGTGGTGTGGAGGCTAGGAATGAGGCTTAA
                        90       100       110       120       130       140       150       160
               ---------+---------+---------+---------+---------+---------+---------+---------+
pSgua-5S1      ....................T.....C.....................................................
pSgua-5S2      ..........................C.....................................................
pSgua-5S3      ..A......................ATT....................................................
pSgua-5S4      ...........A.........A....T...............G..A........................A.........

Majority       TAXAATTAAGAGTGCTAGGGATGAT
                       170       180
               ---------+---------+-----
pSgua-5S1      ..G.........A............       185 bp
pSgua-5S2      ..G......................       185 bp
pSgua-5S3      ..A......................       185 bp
pSgua-5S4      ..A............G.........       185 bp
```

pSgua-5S1	pSgua-5S2	pSgua-5S3	pSgua-5S4	
***	96.8	94.6	93.0	pSgua-5S1
	***	95.7	94.1	pSgua-5S2
		***	94.1	pSgua-5S3
			***	pSgua-5S4

Similarity: 93.0-96.8

S. habrochaites, hab

```
Majority  CCTTTTTGTTGAAATTTGATCTCGTAATTGAAAAAAAAAATATACTCATTTATTTATTTTTGCGGAAAATACGTTCGGGT
                  10        20        30        40        50        60        70        80
          ---------+---------+---------+---------+---------+---------+---------+---------+
hab-C1R1  ....C....................T......................................................
hab-C2R1  .......................................-........................................
hab-C2R2  ..............................................A.................................

Majority  TGAGACGTCATTAGGATATGGGATGGTGGCGTCGGGGATGGGCGTGACGGGCGTCGTCGTGCGTCGGTGCGTGGAGGGTT
                  90       100       110       120       130       140       150       160
          ---------+---------+---------+---------+---------+---------+---------+---------+
hab-C1R1  ............G.......................T...........................................
hab-C2R1  ................................................................................
hab-C2R2  ....................A...........................................................

Majority  TTAAAGCGAGGGGGGCGGGCTAGGGCGTTGGAAGGAAGGTTGTGTTTAATAGATTTTAGAGTGCAATGAATGAC
                 170       180       190       200       210       220       230
          ---------+---------+---------+---------+---------+---------+---------+----
hab-C1R1  ..........................................................................   234 bp
hab-C2R1  ..........................................................................   233 bp
hab-C2R2  .......--.................................................................   232 bp
```

hab-C1R1	hab-C2R1	hab-C2R2	
***	97.9	96.6	hab-C1R1
	***	97.9	hab-C2R1
		***	hab-C2R2

Similarity: 96.6-97.9

S. hindsianum, hin

```
Majority    CCTTTTTXCGCCGAAATTCCGTCGTCTATTCGGCGAATCATTATTTTTGTTGGCGGTTGGGTCGTTTGCTTGGGCCGAGG
                     10        20        30        40        50        60        70        80
            ---------+---------+---------+---------+---------+---------+---------+---------+
pShin-5S4   .......-........................................................................
pShin-5S7   .......T........................................................................

Majority    CGTCGTTAGGACGGTTGAGGAAGGGCGTCGCCGGCGCGTGGAGGCTAGGGCGGTGGTGTGCAGGCTAGGGCGTGGGTGAG
                     90       100       110       120       130       140       150       160
            ---------+---------+---------+---------+---------+---------+---------+---------+
pShin-5S4   ................................................................................
pShin-5S7   ................................................................................

Majority    GAATXAGGTTTAATAGAATTAGGAGTGCTAGGAATGAC
                     170       180       190
            ---------+---------+---------+--------
pShin-5S4   ....G.................................     197
pShin-5S7   ....A.................................     198
```

pShin-5S4	pShin-5S7	
***	99.0	pShin-5S4
	***	pShin-5S7

S. hondelmannii, hdm

```
Majority  CCTTTTTGTCGAAATTTGGTCGTGTAATTGAAAAAATATTATTATTTATTTTTTGCAGGAACGACGTCGTTAGGACAGGT
                   10        20        30        40        50        60        70        80
          ---------+---------+---------+---------+---------+---------+---------+---------+
hdm-C1R1  ................................................................................
hdm-C1R2  .............G..................................................................
hdm-C3R1  ...................................................A............................
hdm-C4R1  ................................................................................
hdm-C4R2  ......................G.........................................................

Majority  GATGGGGGCGTTGAGGATGGGCGTGACGGGCGGCGTCATGCGTCGGTGCGCGTGGAGGCTAGGTCGGTGGGGGG------
                   90       100       110       120       130       140       150       160
          ---------+---------+---------+---------+---------+---------+---------+---------+
hdm-C1R1  ..........................................................................CAGGCT
hdm-C1R2  ....................T.....................................................------
hdm-C3R1  ..........................................................................------
hdm-C4R1  ............A...............................................G.............------
hdm-C4R2  .........................G................................................------

Majority  --------------CAGGCTAGGGCGTTGGGAGGAAGGAGGTGTTTAATAGAATTTAGAGTGCTATGAATGAT
                  170       180       190       200       210       220       230
          ---------+---------+---------+---------+---------+---------+---------+---
hdm-C1R1  AGGGCGTTGGGGGG...........................................................    233 bp
hdm-C1R2  --------------..................................................G........    213 bp
hdm-C3R1  --------------..............................................G............    213 bp
hdm-C4R1  --------------...........................................................    213 bp
hdm-C4R2  --------------...........................................................    213 bp
```

hdm-C1R1	hdm-C1R2	hdm-C3R1	hdm-C4R1	hdm-C4R2	
***	90.1	90.6	90.6	90.6	hdm-C1R1
	***	97.9	97.9	97.9	hdm-C1R2
		***	98.3	98.3	hdm-C3R1
			***	98.3	hdm-C4R1
				***	hdm-C4R2

Similarity: 90.1-98.3

S. horridum, hor

```
Majority   CTTTTTTCGX-XXXXXXXX-TCGTCTATTCGGCCGATGACTATTTTTTTTT--GCXGXAGGGCCGTTTGCTTGGGCAGAG
                   10        20        30        40        50        60        70        80
           ---------+---------+---------+---------+---------+---------+---------+---------+
hor-C3R1   .........-----------...............................--..T.T......................
hor-C22R1  .........-----------...............................TT..T.T......................
hor-C23R1  .........-----------...............................T-..T.T......................
hor-C9R1   .........ATGAAATTTCG......C........................--..G.C......................
hor-C9R2   .........ACGAAATTTCG..........A........G...........--..G.C......................
hor-C11R1  .........ACGAAATTTCA.............A................---..G.C..A...................

Majority   XCGTCGXTAGGACGGCTGAGGAXGGC-GTCGGCGGCGCGGAGGCTAGGGCGXATGGGGTGCXGXAATGAGGTTTAATA
                   90       100       110       120       130       140       150       160
           ---------+---------+---------+---------+---------+---------+---------+---------+
hor-C3R1   A.....T...............A..TA..........................T...T.....A.A..............
hor-C22R1  A.....T...A...........A..TA..........................T...T.....A.A..............
hor-C23R1  A.....T........T......A..GC..A....................-.TT.........A.A..............
hor-C9R1   T.....C..C............G...-..........................-.........T.T..............
hor-C9R2   T.....C...............G...-..........................-.C.......T.T..............
hor-C11R1  T.....C...............G...-..........................-.C.......T.T..............

Majority   GAATTAAGAGTGCTAGGATTGAC
                  170       180
           ---------+---------+---
hor-C3R1   ...............T......T     170 bp
hor-C22R1  ...............T.......     172 bp
hor-C23R1  ....A..................     170 bp
hor-C9R1   .........A.......T.A...     179 bp
hor-C9R2   ...............C.......     179 bp
hor-C11R1  .......................     178 bp
```

hor-C3R1	hor-C22R1	hor-C23R1	hor-C9R1	hor-C9R2	hor-C11R1	
***	97.8	94.0	84.2	85.2	84.7	hor-C3R1
	***	94.0	83.1	84.2	83.6	hor-C22R1
		***	82.5	83.1	83.1	hor-C23R1
			***	94.5	94.0	hor-C9R1
				***	96.2	hor-C9R2
					***	hor-C11R1

Similarity: 82.5-97.8

S. huaylasense, hua

ONE RIBOTYPE - 229 bp

---

hypacrarthrum, hcr

```
Majority  CCTTTTTGTCGAAATTTGGTCGTGTAATTGAAAAAACAAGTTATGTATTTATTTTTTTGCGGAAACGAAAATTCGTATTG
                   10        20        30        40        50        60        70        80
          ---------+---------+---------+---------+---------+---------+---------+---------+
hcr-C1R1  ................................................................................
hcr-C1R2  ................................................................................
hcr-C1R3  ...........................................................T....................
hcr-C2R1  .................T..............................................................
hcr-C3R1  .........................G......................................................

Majority  AGACGTCGTTAGGAGGCGTGACGGGGGGCGTCATGCGTCGTTGCGTGGAGGCTAGGTCGGTGGGGGGCAGGCTAGGGCGT
                   90       100       110       120       130       140       150       160
          ---------+---------+---------+---------+---------+---------+---------+---------+
hcr-C1R1  ........................................G.......................................
hcr-C1R2  ................................................................................
hcr-C1R3  ........................................-------------------.....................
hcr-C2R1  ........................................................................C.......
hcr-C3R1  ................................................................................

Majority  TGGGAGGAAGGAGGTGTTTAATAGAATTTAGAGTGCTATGAATGAT
                  170       180       190       200
          ---------+---------+---------+---------+------
hcr-C1R1  ..............................................   206 bp
hcr-C1R2  ..............................................   206 bp
hcr-C1R3  ..............................................   187 bp
hcr-C2R1  ..............................................   206 bp
hcr-C3R1  ....................................C.........   206 bp
```

hcr-C1R1	hcr-C1R2	hcr-C1R3	hcr-C2R1	hcr-C3R1	
***	99.5	90.3	98.5	98.5	hcr-C1R1
	***	90.3	99.0	99.0	hcr-C1R2
		***	89.3	89.3	hcr-C1R3
			***	98.1	hcr-C2R1
				***	hcr-C3R1

Similarity: 89.3-99.5

S. incamayoense, inm

```
Majority  CCTTTTTGTCGAAATTTGGTCGTGTAATTGAAAAAATAT---TATTA--ATT-ATTTTTTGCAGGAACGACGTCGTTAGG
                   10        20        30        40        50        60        70        80
          ---------+---------+---------+---------+---------+---------+---------+---------+
inm-C1R1  .......................................---.....--...-.A............T............
inm-C1R2  .......................................---.....--...-...........................
inm-C1R3  ................C...............G......ATT.....TA...T...........A...G..........
inm-C1R4  ....................A..................---.....--...-...........................

Majority  TTAGGTGATGGGGGCGTTGAGGATGGGCGTGACGGGCGGCGTCATGCGTCGGTGCGCGTGGAGGCTAGGTCGGTGGGGGG
                   90       100       110       120       130       140       150       160
          ---------+---------+---------+---------+---------+---------+---------+---------+
inm-C1R1  .....................................T..........................................
inm-C1R2  ................................................................................
inm-C1R3  AC...C........................................................................A.
inm-C1R4  ..................................................................G.............

Majority  CAGGCTAGGGCGTTGGGAGGAAGGAGGTGTTTAATAGXATTTAGAGTGCTATGAATGAT
                  170       180       190       200       210
          ---------+---------+---------+---------+---------+---------
inm-C1R1  ............A..............G.........G...........G.........    213 bp
inm-C1R2  .....................................A.....................    213 bp
inm-C1R3  ...T.................................G.....................    219 bp
inm-C1R4  .....................................A.....................    213 bp
```

inm-C1R1	inm-C1R2	inm-C1R3	inm-C1R4	
***	96.8	90.4	95.9	inm-C1R1
	***	92.7	99.1	inm-C1R2
		***	91.8	inm-C1R3
			***	inm-C1R4

Similarity: 90.4-99.1

S. incanum, inc

```
Majority CTTTTTTTGCTGAAATTTCGTCGTCTATTCGGCGAATGATTATTTTTTTTGGCGGTTGGGTCGTTTGCTCGGGGAGAGAC
                10        20        30        40        50        60        70        80
         ---------+---------+---------+---------+---------+---------+---------+---------+
inc-C1R1 ................................................................................
inc-C1R2 ................................................................................
inc-C4R1 .............G..................................................................
inc-C4R2 ................................................................................
inc-C4R3 ...........................................................A....................

Majority GTCGTTAGGACGGTTGAGGAAGGTCGTCGCGGTGGCAAAAAGCCCTGTGGCGGTGGTGTGCATGGCTAGGGCTAGGGCGT
                90       100       110       120       130       140       150       160
         ---------+---------+---------+---------+---------+---------+---------+---------+
inc-C1R1 ..................A.............................................................
inc-C1R2 ............T.............................G.....................................
inc-C4R1 .................................A............................................C.
inc-C4R2 .................................A............................................C.
inc-C4R3 ..........................................G.....................................

Majority GGGGGGAGGAATGAGGTTTAATAGAATTAAGAGTGCTAGGAATGAC
               170       180       190       200
         ---------+---------+---------+---------+------
inc-C1R1 .....................................G........               206
inc-C1R2 ..............................................               206
inc-C4R1 ..........................G.................C.               206
inc-C4R2 .............................................T               206
inc-C4R3 ..............................................               206
```

inc-C1R1	inc-C1R2	inc-C4R1	inc-C4R2	inc-C4R3	
***	98.1	96.6	97.6	98.1	inc-C1R1
	***	96.6	97.6	99.0	inc-C1R2
		***	98.1	96.6	inc-C4R1
			***	97.6	inc-C4R2
				***	inc-C4R3q

Similarity: 96.6-99.0

S. infundibuliforme, ifd

```
Majority  CCTTTTTGTCGAAATTTGGTCGTGTAATTGAAAAAATAATATTATTTATTTTTTGCAGGAACGACGTCGTTAGGACAGGT
                  10        20        30        40        50        60        70        80
          ---------+---------+---------+---------+---------+---------+---------+---------+
ifd-C1R1  ................................................................................
ifd-C2R1  ................................................................................
ifd-C3R1  ................................................................................
ifd-C3R2  ...........................A.........G..........................................

Majority  GATGGGGGCGTTGAGGATGGGCGTGACGGGCGGCGTCATGCGTCGGTGCGCGTGGAGGCTAGGTCGGTGGGGGGCAGGCT
                  90       100       110       120       130       140       150       160
          ---------+---------+---------+---------+---------+---------+---------+---------+
ifd-C1R1  ................................................................................
ifd-C2R1  ................................C...............................................
ifd-C3R1  ................GC................................T.............................
ifd-C3R2  ............A...................................................................

Majority  AGGGCGTTGGGAGGAAGGAGGTGTTTAATAGAATTTAGAGTGCTATGAATGAT
                 170       180       190       200       210
          ---------+---------+---------+---------+---------+---
ifd-C1R1  .....................................................     213 bp
ifd-C2R1  .....................................................     213 bp
ifd-C3R1  .....................................................     213 bp
ifd-C3R2  .....................................................     213 bp
```

ifd-C1R1	ifd-C2R1	ifd-C3R1	ifd-C3R2	
***	99.5	98.6	98.6	ifd-C1R1
	***	98.1	98.1	ifd-C2R1
		***	97.2	ifd-C3R1
			***	ifd-C3R2

Similarity: 97.2-99.5

S. iopetalum, iop

```
Majority CCTTTTTGTCGAAATTTGGTCGTGTAATTGAAAAAATATAATTATTTXTTTTTTGCATGAACGACGTCGTTAGGACAGGA
               10        20        30        40        50        60        70        80
         ---------+---------+---------+---------+---------+---------+---------+---------+
iop5S.2  ...............................................A................................
iop5S.3  ...............................................A................................
iop5S.4  .......................................T.......G................................
iop5S.5  ..................CT...........................G................................

Majority GATGGGGGCGTTGAGGATGGGCGTGACGGGCGACGTCATGCGTCGGTGCGCGTGGAGGCTAGGTCGGTXGGGGGCAGGCT
               90       100       110       120       130       140       150       160
         ---------+---------+---------+---------+---------+---------+---------+---------+
iop5S.2  ....................................................................G...........
iop5S.3  ....................-...........G...................................G...........
iop5S.4  ....................................................................C...........
iop5S.5  ........--..........................................................C...........

Majority AGGGCGTTGGGAGGAAGGAGGTGTTTAATAGAATTTAGAGTGCTATGAATGAT
              170       180       190       200       210
         ---------+---------+---------+---------+---------+---
iop5S.2  .....................................................    213 bp
iop5S.3  .....................................................    212 bp
iop5S.4  .....................................................    213 bp
iop5S.5  .....................................................    211 bp
```

iop5S.2	iop5S.3	iop5S.4	iop5S.5	
***	99.1	98.6	97.2	iop5S.2
	***	97.7	96.2	iop5S.3
		***	97.7	iop5S.4
			***	iop5S.5

Similarity: 96.2-99.1

S. jamesii-1, jam1

DIRECT SEQUENCING - 213 bp

S. jamesii-2, jam2

```
Majority  CCTTTTTGTCGAAATTCGGTCGTGXAATTGAAAAAATATATTATTAATTTTTTTGCAGAAACGACATCCGGATTGATGGG
                  10        20        30        40        50        60        70        80
          ---------+---------+---------+---------+---------+---------+---------+---------+
jam2-C1R1 .......................C........................................................
jam2-C2R1 .......................T........................................................

Majority  TGATGGGGGCGTTGAGGATGGGCGTGACGGGCGGCGTCAAGCGACGGTGCGTGGAGGCTAGGTCGGXGGGGGGCAGGCTA
                  90       100       110       120       130       140       150       160
          ---------+---------+---------+---------+---------+---------+---------+---------+
jam2-C1R1 .................................................................G..............
jam2-C2R1 .................................................................T..............

Majority  GGGCGTTGGGAGGAGGGAGGTGTTTAATAGAATTTAGAGTGCTATGAATGAT
                 170       180       190       200       210
          ---------+---------+---------+---------+---------+--
jam2-C1R1 ....................................................     212 bp
jam2-C2R1 ....................................................     212 bp
```

jam2-C1R1	jam2-C2R1	
***	99.1	jam2-C1R1
	***	jam2-C2R1

Similarity: 99.1

S. juzepczukii, juz

```
Majority  CCTTTTT-GTCGAAATTCGGTCGTGCAATTGATTTTTTATTATTATTTATCTTTTTGCAGAA--AC-----------GGC
                  10        20        30        40        50        60        70        80
          ---------+---------+---------+---------+---------+---------+---------+---------+
juz-C1R1  .T.....-.C..........C....T.G....AACAA...-......-..G...........CG..ATTCGGATTGA.A.
juz-C1R2  .......-......................................................--..-----------...
juz-C2R1  .......-.C..........C....T.G....AACAA...-......-..G...........CG..ATTCGGATTGA.A.
juz-C4R1  .......-......................................................--..-----------...
juz-C5R1  .......-......................................................--..-----------...
juz-C6R1  ...C...-......................................................--..-----------...
juz-C9R1  .......T......................................................--..-----------...
juz-C11R1 .......-......................................................--..-----------...

Majority  GTCGTTAGGACAGGTGATGGGGGCGTTGGGGATGGGCGTGACGGGCGGCGTCATGCGTCGGTGCGCGTGGAGGCTAGGTC
                  90       100       110       120       130       140       150       160
          ---------+---------+---------+---------+---------+---------+---------+---------+
juz-C1R1  ............................A.......G............................--.............
juz-C1R2  ................................................................................
juz-C2R1  ............................A.......G............................--.............
juz-C4R1  ................................................................................
juz-C5R1  .................................................G..............................
juz-C6R1  ................................................................................
juz-C9R1  ................................................................................
juz-C11R1 ........................A.......................................................

Majority  CGGTGGGGGGCAGGCTAGGGCGTTGGGAGGAAGGAAAGGAGGTGCTTAAAAGAATTTAGAGTGCTATGAATGAT
                 170       180       190       200       210       220       230
          ---------+---------+---------+---------+---------+---------+---------+----
juz-C1R1  -..................................-----.........T........................   223
juz-C1R2  ..........................................................................   220
juz-C2R1  -..................................-----.........T........................   223
juz-C4R1  ............................................T.............................   220
juz-C5R1  ............................................T.............................   220
juz-C6R1  ............................................T.............................   220
juz-C9R1  ..........................................................................   221
juz-C11R1 ..........................................................................   220
```

juz-C1R1	juz-C1R2	juz-C2R1	juz-C4R1	juz-C5R1	juz-C6R1	juz-C9R1	juz-C11R1	
***	83.8	99.6	83.3	82.9	82.9	83.3	83.3	juz-C1R1
	***	84.2	99.6	99.1	99.1	99.6	99.6	juz-C1R2
		***	83.8	83.3	83.3	83.8	83.8	juz-C2R1
			***	99.6	99.6	99.1	99.1	juz-C4R1
				***	99.1	98.7	98.7	juz-C5R1
					***	98.7	98.7	juz-C6R1
						***	99.1	juz-C9R1
							***	juz-C11R1

Similarity: 82.9-99.6

S. kurtzianum, ktz

```
Majority  CCTTTTTGTCGAAATTCGGTCGTGTAATGGAAGAAATATATTTATTTAT-TTTTTGCAGAAACGGCGTCGTTAGGACAGG
                   10        20        30        40        50        60        70        80
          ---------+---------+---------+---------+---------+---------+---------+---------+
ktz-C1R1  .................................................-..G............T-.TAA.GATG.-..
ktz-C1R2  .................................................-..............................
ktz-C1R3  .................................................-..............................
ktz-C1R4  ............................A...................AA..............................
ktz-C1R5  .................................................-.C............................
ktz-C4R1  ............................A...................AA.C............................

Majority  TGACGGGGGCGTTGAGGATG-G-GCGTGACGGGCGGCGTCATGCGTCGGTGCGCGTGGAGGCTAGGTCGGTGGGGGGCAG
                   90        100       110       120       130       140       150       160
          ---------+---------+---------+---------+---------+---------+---------+---------+
ktz-C1R1  C.T.CACC.A.A.CTAC.CTC.T..CC....C.AC..TCTTCCG...T................................
ktz-C1R2  ....................-.-.................................T.......................
ktz-C1R3  ...................TC.-.........................................................
ktz-C1R4  ...T................-.-.........................................................
ktz-C1R5  ..............C.....-.-.........................................................
ktz-C4R1  ...T................-.-.........................................................

Majority  GCTAGGGCGTTGGGAGGAAGGAGGCGTTTAATAGAATTTAGAGTGCTATGAATGAT
                   170       180       190       200       210
          ---------+---------+---------+---------+---------+------
ktz-C1R1  ...T....................................................    213 bp
ktz-C1R2  ........................................................    213 bp
ktz-C1R3  ........................................................    214 bp
ktz-C1R4  ........................T..............G................    214 bp
ktz-C1R5  ......T.................................................    213 bp
ktz-C4R1  ........................T...............................    214 bp
```

ktz-C1R1	ktz-C1R2	ktz-C1R3	ktz-C1R4	ktz-C1R5	ktz-C4R1	
***	80.6	81.9	78.2	80.1	78.2	ktz-C1R1
	***	98.6	96.8	98.1	96.8	ktz-C1R2
		***	96.3	97.7	96.3	ktz-C1R3
			***	95.8	99.1	ktz-C1R4
				***	96.8	ktz-C1R5
					***	ktz-C4R1

Similarity: 78.2-99.1

S. laciniatum, lac

```
Majority    CCTTTTTATCGAAACTCATCATAATTTTATCTATTTGGTGATTAATTTTATTTTTT-AATTTTTGCGGAAAGGACATTCG
                    10        20        30        40        50        60        70        80
            ---------+---------+---------+---------+---------+---------+---------+---------+
lac-C1R1    ........................................................-.......................
lac-C3R1    ........................................................-.......................
lac-C11R1   ........................................................-.......................
lac-C13R2   .T.....G......T..GC......................A..............-..........AT...........
lac-C13R3   ........................................................T.......................
lac-C26R1   ........................................................-.......................
lac-C13R1   .T.....G......T..GC....................AG-------------..-.......A.C.............

Majority    GGTGGAGACGTCGTTAGGACAGGTGATGGAGGCGTTTGAAATTGGCGTGAAAGGCGGCACCATGCGTCGGCAGGCTAGGG
                    90       100       110       120       130       140       150       160
            ---------+---------+---------+---------+---------+---------+---------+---------+
lac-C1R1    ..............................C.................................................
lac-C3R1    ................................................................................
lac-C11R1   .............................................T..................................
lac-C13R2   AA.C.GA.........AA..T.......A..C..C...GG..G......GC............GA...............
lac-C13R3   ................................................................................
lac-C26R1   .................................A..............................................
lac-C13R1   AA.A............AAG.......C.......C...GG..G.......C...........................C.

Majority    CGTTGGGAGGAAGGAGGTGTTTAATAAAATTTGGAGTGCTAGGAATGAC
                   170       180       190       200
            ---------+---------+---------+---------+---------
lac-C1R1    ..A..............................................   208 bp
lac-C3R1    .................................................   208 bp
lac-C11R1   .................................................   208 bp
lac-C13R2   ...............A......G................G.........   208 bp
lac-C13R3   .................................................   209 bp
lac-C26R1   .................................................   208 bp
lac-C13R1   .......................................G.........   195 bp
```

lac-C1R1	lac-C3R1	lac-C11R1	lac-C13R2	lac-C13R3	lac-C26R1	lac-C13R1	
***	99.0	98.6	85.2	98.6	98.6	81.8	lac-C1R1
	***	99.5	86.1	99.5	99.5	82.8	lac-C3R1
		***	85.6	99.0	99.0	82.3	lac-C11R1
			***	85.6	85.6	84.2	lac-C13R2
				***	99.0	82.3	lac-C13R3
					***	82.3	lac-C26R1
						***	lac-C13R1

Similarity: 81.8-99.5

S. lasiophyllum, las

```
Majority  TTTTTTTCGCCGAAATTTCGTCGTCTATTCGGCTAATGATTATCTTTTTTGGCGGTTGGGTCGCTTGAXTXGGCAGAGAC
                   10        20        30        40        50        60        70        80
          ---------+---------+---------+---------+---------+---------+---------+---------+
las-C1R1  ........A...........................................................T.G.........
las-C1R2  ....................................................................A.T....T....
las-C3R1  A...................................................................A.T....T....
las-C4R1  .......G...................................A........................T.G.........
las-C4R2  C........T.........A.........A......................................T.G.........
las-C2R1  C...................................................................A.T........-

Majority  GTCGTTAGGACGGATGAGGAAGGGCGTCGCCGGCGCGCGGAGGCTAGGGCGACGGTGTGCAGGAATGACGTTTAATXGAA
                   90       100       110       120       130       140       150       160
          ---------+---------+---------+---------+---------+---------+---------+---------+
las-C1R1  ....................................................G...............G.......T...
las-C1R2  .....................T.......................................................A...
las-C3R1  .....................T........T..............................................A...
las-C4R1  .............................................T......................G.......T...
las-C4R2  .............................................................................A...
las-C2R1  -------------.....--------..-------------------------------------------.....TT..

Majority  TTAAGTGTGCTAGGAATGAC
                   170       180
          ---------+---------+
las-C1R1  ....................    180 bp
las-C1R2  ....................    180 bp
las-C3R1  ....................    180 bp
las-C4R1  ............A.......    180 bp
las-C4R2  ..........A.........    180 bp
las-C2R1  ....................    115 bp
```

las-C1R1	las-C1R2	las-C3R1	las-C4R1	las-C4R2	las-C2R1	
***	95.6	94.4	96.7	95.0	61.1	las-C1R1
	***	98.9	94.4	95.0	61.7	las-C1R2
		***	93.3	94.4	61.7	las-C3R1
			***	93.9	60.0	las-C4R1
				***	59.4	las-C4R2
					***	las-C2R1

Similarity: 59.4-98.9

S. laxissimum-1, lxs1

```
Majority  CCTTTTTGTCGAAATTCGGTCGTGCAACTGAAAAAATTTATTTATTTATTTTTTGCAGAAACGACGTCGTTAGGACAGGT
                  10        20        30        40        50        60        70        80
          ---------+---------+---------+---------+---------+---------+---------+---------+
lxs_5S.1  ........A.......................................................................
lxs_5S.2  ................................................................................
lxs_5S.3  ........A...................................................................T...
lxs_5S.4  ................................................................................
lxs_5S.5  ................................................................................

Majority  GATGGTGGGCGTTGAGGATGGGCGTGACGGGCGTCGGTGCGTGGAGGCTAGGTCGGCGGAGGGCAGGCTAGGGCGTTGGG
                  90       100       110       120       130       140       150       160
          ---------+---------+---------+---------+---------+---------+---------+---------+
lxs_5S.1  ........................................................T.......................
lxs_5S.2  ................................................................................
lxs_5S.3  ..........................T................................G....................
lxs_5S.4  .........GT................................................G....................
lxs_5S.5  ................................................................................

Majority  AGGAAGGAGGTGTTTAATAGAATTTAGAGTGCTATGAATGAT
                 170       180       190       200
          ---------+---------+---------+---------+--
lxs_5S.1  ..........................................    202 bp
lxs_5S.2  ..........................................    202 bp
lxs_5S.3  .......G..................................    202 bp
lxs_5S.4  ..........................................    202 bp
lxs_5S.5  ..........................................    202 bp
```

lxs_5S.1	lxs_5S.2	lxs_5S.3	lxs_5S.4	lxs_5S.5	
/***	99.0	97.5	97.5	99.0	lxs_5S.1
	***	97.5	98.5	100.0	lxs_5S.2
		***	97.0	97.5	lxs_5S.3
			***	98.5	lxs_5S.4
				***	lxs_5S.5

Similarity: 97.0-100

S. laxissimum-2, lxs2

```
Majority      CCTTTTTGXCGAAAXTTCGGTCGTGCAACTGAAAAAXTTTXTTTATTTATTTTTTGCAGAAACGACGTCGTTAGGACAGG
                       10        20        30        40        50        60        70        80
              ---------+---------+---------+---------+---------+---------+---------+---------+
lxs2-C1R4     ........A.....A.....................A...A.......................................
lxs2-C1R5     ........T.....-....................TT...G.......................................
lxs2-C1R6     ........T.....-C....................T...G........................T.....A......A.
lxs2-C1R10    ........A.....A.....................A...A.......................................

Majority      TGATGGTGGGCGTTGAGGATGGGCGTGACGGGCGTCGGTGCGTGGAGGCTAGGTCGGCGGGGGGCAGGCTAGGGCGTTGG
                       90       100       110       120       130       140       150       160
              ---------+---------+---------+---------+---------+---------+---------+---------+
lxs2-C1R4     ................................................................................
lxs2-C1R5     -...............................................................................
lxs2-C1R6     ..T..................................A..........................................
lxs2-C1R10    .......................................A........................................

Majority      GAGGAAGGAGGTGTTTAATAGAATTTAGAGTGCTATGAATGAT
                      170       180       190       200
              ---------+---------+---------+---------+---
lxs2-C1R4     ...........................................     203 bp
lxs2-C1R5     ....................C......................     201 bp
lxs2-C1R6     ...........................................     202 bp
lxs2-C1R10    ...........................................     203 bp
```

lxs2-C1R4	lxs2-C1R5	lxs2-C1R6	lxs2-C1R10	
***	96.6	95.1	99.5	lxs2-C1R4
	***	95.6	96.1	lxs2-C1R5
		***	94.6	lxs2-C1R6
			***	lxs2-C1R10

Similarity: 94.6-99.5

S. leptophyes, lph

DIRECT SEQUENCING - 213 bp

S. limbaniense, lmb

```
Majority CCTTTTTGTCGAAATTCGGTCGTGTAATAGAAAAAA-TATTATTATTTATTT---TTTGTAGAAACGACGTCGTTAGGAC
                  10        20        30        40        50        60        70        80
         ---------+---------+---------+---------+---------+---------+---------+---------+
lmb-C1R1 ....................................-............G..---.........................
lmb-C1R2 ....................................-...............---.........................
lmb-C1R3 ..........................G........AG...............ATT....C....................
lmb-C1R4 ....................................-.....GG........---.........................
lmb-C1R5 T...........G.......................-...............---..............A..........
lmb-C2R1 ..........................G........A................ATT....C....................
lmb-C2R2 ....................................-...C...........---.........................
lmb-C4R1 ....................................-...............---.........................

Majority  AACAGGTGATGGGGGCGTTGGGAGGACGGGCGTGACGGGCGGCGTCATGCGTCGGTGCGCGTGGAGGCTAGGTCGGTGG
                  90       100       110       120       130       140       150       160
         ---------+---------+---------+---------+---------+---------+---------+---------+
lmb-C1R1 ................................................................................
lmb-C1R2 ................................................................................
lmb-C1R3 ----................--.....T....................................................
lmb-C1R4 .T.......G.............................................................A........
lmb-C1R5 ...........C....................................................................
lmb-C2R1 ----................--....CT....................................................
lmb-C2R2 ................................................................................
lmb-C4R1 ...........................A....................................................

Majority GGGGCAGGCTAGGGCGTTGGGAGGAAGGAGGTGTTTAATAGAATTTAGAGTGCTATGAATGAT
                 170       180       190       200       210       220
         ---------+---------+---------+---------+---------+---------+---
lmb-C1R1 ........................C......................................  219 bp
lmb-C1R2 ...............................................................  219 bp
lmb-C1R3 ...............................................................  217 bp
lmb-C1R4 ...............................................................  219 bp
lmb-C1R5 ...............................................................  219 bp
lmb-C2R1 .C.............................................................  217 bp
lmb-C2R2 ....................................G..........................  219 bp
lmb-C4R1 ...............................................................  219 bp
```

lmb-C1R1	lmb-C1R2	lmb-C1R3	lmb-C1R4	lmb-C1R5	lmb-C2R1	lmb-C2R2	lmb-C4R1	
***	99.1	92.8	96.9	97.3	92.4	98.2	98.7	lmb-C1R1
	***	93.7	97.8	98.2	93.3	99.1	99.6	lmb-C1R2
		***	91.9	91.9	98.7	92.8	93.7	lmb-C1R3
			***	96.0	91.5	96.9	97.3	lmb-C1R4
				***	91.5	97.3	97.8	lmb-C1R5
					***	92.4	93.3	lmb-C2R1
						***	98.7	lmb-C2R2
							***	lmb-C4R1

Similarity: 91.5-99.6

S. linnaeanum, lin

```
Majority TTTTTTT-GCAGAAATTCCGTCGTCTATTCGGCGAATGATTATTTTTTTTGGCGGTTGGGTCGTTTGCTCGGGGATAGAC
                  10        20        30        40        50        60        70        80
         ---------+---------+---------+---------+---------+---------+---------+---------+
lin-C1R1 .......T........................................................................
lin-C2R1 .......-........................................................................
lin-C2R2 .......-.......G................................................................

Majority GTCGTTAGGACGGTTGAGGAAGGTGGTCGATGTAGCAAAAAGGCCTGTGGCGGTGGTGTGCATGGCTAGGGCTAGGGC--
                  90       100       110       120       130       140       150       160
         ---------+---------+---------+---------+---------+---------+---------+---------+
lin-C1R1 ..............................................................................--
lin-C2R1 ..............................................................................--
lin-C2R2 ..............................................................................TA

Majority ----GTGGGGGGAGGAATGAGGTTTAATAGAATTAAGAGTGCTAGGAATGAT
                 170       180       190       200       210
         ---------+---------+---------+---------+---------+--
lin-C1R1 ----................................................    206 bp
lin-C2R1 ----................................................    205 bp
lin-C2R2 GGGC................................................    211 bp
```

lin-C1R1	lin-C2R1	lin-C2R2	
***	99.5	96.2	lin-C1R1
	***	96.7	lin-C2R1
		***	lin-C2R2

Similarity: 96.2-99.5

S. lycopersicoides, lpd

```
Majority  CCTTTTTGTTGAAATTTGATCTCGTAATTGAAAAAAAAAXXTATACTCATTTATTTTTTTTXXTGCGGAAAATACGTTCG
                   10        20        30        40        50        60        70        80
          ---------+---------+---------+---------+---------+---------+---------+---------+
lpd-C1R1  .......................................--....................TT.................
lpd-C8R1  .......................................AA....................--.................

Majority  GATTXAGGCGTCATTAGGATATGGATGGTGGCGTCGGGGAXXXXXXXXXCGGGCGTCGTCGTGCGTCGGTGCGTGGAGG
                   90       100       110       120       130       140       150       160
          ---------+---------+---------+---------+---------+---------+---------+---------+
lpd-C1R1  ....G...................................TGGGCGTGA..............................
lpd-C8R1  ....T...................................---------..............................

Majority  GTTTTAAAGCGGGGGGCGGGCTAGGGCGTTGGGAGGAAGGTTGTGTTTAATAGATTTTAGAGTGCAATGAATGAC
                  170       180       190       200       210       220       230
          ---------+---------+---------+---------+---------+---------+---------+-----
lpd-C1R1  ...........................................................................  233 bp
lpd-C8R1  ...........................................................................  224 bp
```

lpd-C1R1	lph-C8R1	
***	92.4	lpd-C1R1
	***	lph-C8R1

Similarity: 92.4

S. lycopersicum var. cerasiforme-1, lycc1

```
Majority     CCTTTTTGTTGAAATTTGATCTCGTAATTGAAAAAAAAAAATATACTCATTTATTTTTTTTT-GCGGAAAATACGTTCG
                      10        20        30        40        50        60        70        80
             ---------+---------+---------+---------+---------+---------+---------+---------+
lycc1-C1R1   ...............................................................-................
lycc1-C1R2   ........................................-......................-................
lycc1-C2R1   .......T.......................................................-............G...
lycc1-C2R2   ...............................................................T................

Majority     GATTGAGGCGTCATTAGGATATGGGATGGTGGCGTCGGGGATGGGCGTGACGGGCGTCGTCGTGCGTCGGTGCGTGGAGG
                      90       100       110       120       130       140       150       160
             ---------+---------+---------+---------+---------+---------+---------+---------+
lycc1-C1R1   ................................................................................
lycc1-C1R2   ................................................................................
lycc1-C2R1   ..................................G...................................G.........
lycc1-C2R2   ................................................................................

Majority     GTTTTAAAGCGGGGGGCGGGCTAGGGCGTTGGGAGGAAGGTTGTGTTTAATAGATTTTAGAGTGCAATGAATGAC
                     170       180       190       200       210       220       230
             ---------+---------+---------+---------+---------+---------+---------+-----
lycc1-C1R1   ........................................................................... 234 bp
lycc1-C1R2   ........................................................................... 233 bp
lycc1-C2R1   ........................................................................... 234 bp
lycc1-C2R2   .........................................................................C. 235 bp
```

lycc1-C1R1	lycc1-C1R2	lycc1-C2R1	lycc1-C2R2	
***	99.6	98.3	99.1	lycc1-C1R1
	***	97.9	98.7	lycc1-C1R2
		***	97.4	lycc1-C2R1
			***	lycc1-C2R2

Similarity: 97.4-99.6

S. lycopersicum var. Cerasiforme-2, lycc2

ONE RIBOTYPE - 234 bp

S. lycopersicum-1, lyc1

ONE CLONE from GB - 235 bp

S. lycopersicum-2, lyc2

```
Majority      CCTTTTTGTTGAAATTTGATCTCGTAATTGAAAAAAAAAAATATACTCATTTATTTTTTTTTT-GCGGAAAATACGTTCG
                      10        20        30        40        50        60        70        80
              ---------+---------+---------+---------+---------+---------+---------+---------+
lyc2-C2R2     .T......................................................A......-................
lyc2-C6R2     ....................................---.......................C--...............
lyc2-C8R1     ...............................................................T................
lyc2-C10R1    ...............................................................-................
lyc2-C15R2    ........................................-...............-......T................

Majority      GATTGAGGCGTCATTAGGATATGGGATGGTGGCGTCGGGGATGGGCGTGACGGGCGTCGTCGTGCGTCGGTGCGTGGAGG
                      90       100       110       120       130       140       150       160
              ---------+---------+---------+---------+---------+---------+---------+---------+
lyc2-C2R2     ................................................................................
lyc2-C6R2     ................................................................T...............
lyc2-C8R1     ...................G....................................A.......................
lyc2-C10R1    ................................................................................
lyc2-C15R2    .............................................................A..................

Majority      GTTTTAAAGCGGGGGGCGGGCTAGGGCGTTGGGAGGAAGGTTGTGTTTAATAGATTTTAGAGTGCAATGAATGAC
                     170       180       190       200       210       220       230
              ---------+---------+---------+---------+---------+---------+---------+-----
lyc2-C2R2 ..........................................................................  234 bp
lyc2-C6R2 ...............A..A..A.....A...............................................  230 bp
lyc2-C8R1 ...........................................................................  235 bp
lyc2-C10R1 .............A............................................................. 234 bp
lyc2-C15R2 ..........................T................................................ 233 bp
```

lyc2-C2R2	lyc2-C6R2	lyc2-C8R1	lyc2-C10R1	lyc2-C15R2	
***	94.9	97.9	98.7	97.4	lyc2-C2R2
	***	94.5	95.3	93.6	lyc2-C6R2
		***	98.3	97.4	lyc2-C8R1
			***	97.4	lyc2-C10R1
				***	lyc2-C15R2

Similarity: 93.6-98.7

S. lycopersicum-3, lyc3

```
Majority    CCTTTTTGTTGAAATTTGATCTCGTAATTGAAAAAAAAAA-TATACTCATTTATTTTTTTTTT-GCGGAAAATACGTTCG
                    10        20        30        40        50        60        70        80
            ---------+---------+---------+---------+---------+---------+---------+---------+
lyc3-C1R1   ........................................-......................-................
lyc3-C11R1  ........................................A......................T................
lyc3-C15R1  ........................................-......................T................
lyc3-C25R1  ..........T.............................A.....................--................
lyc3-C39R1  .......................................--......................-................

Majority    GATTGAGGCGTCATTAGGATATGGGATGGTGGCGTCGGGGATGGGCGTGACGGGCGTCGTCGTGCGTCGGTGCGTGGAGG
                    90       100       110       120       130       140       150       160
            ---------+---------+---------+---------+---------+---------+---------+---------+
lyc3-C1R1   ................................................................................
lyc3-C11R1  ................................................................................
lyc3-C15R1  ................................................................................
lyc3-C25R1  ................................................................................
lyc3-C39R1  ................................................................................

Majority    GTTTTAAAGCGGGGGGCGGGCTAGGGCGTTGGGAGGAAGGTTGTGTTTAATAGATTTTAGAGTGCAATGAATGAC
                   170       180       190       200       210       220       230
            ---------+---------+---------+---------+---------+---------+---------+-----
lyc3-C1R1   ........................................................................T.. 233bp
lyc3-C11R1  ........................................................................... 235bp
lyc3-C15R1  ........................................................................... 234bp
lyc3-C25R1  ........................................................................... 233bp
lyc3-C39R1  ........................................................................... 232bp
```

lyc3-C1R1	lyc3-C11R1	lyc3-C15R1	lyc3-C25R1	lyc3-C39R1	
***	98.7	99.1	98.3	99.1	lyc3-C1R1
	***	99.6	98.7	98.7	lyc3-C11R1
		***	98.3	99.1	lyc3-C15R1
			***	98.3	lyc3-C25R1q
				***	lyc3-C39R1

Similarity: 98.3-99.6

S. macrocarpon, mac

```
Majority    CTTTTTTTTCCCGAAATATCXTCGTCTATTCGGCGAATGATTAATTTTTTTTGCGGTTGGGTCGTTTGTTTGGGCAGAGA
                     10        20        30        40        50        60        70        80
            ---------+---------+---------+---------+---------+---------+---------+---------+
mac-C12R1   ....................A...........................................................
mac-C12R2   --.......T..........G...................................A.......................
mac-C64R1   ....................A..................................................AT.......
mac-C91R1   ....................A...........................................................
mac-C91R2   ....................G...........T..................-............................
mac-C133R1  .........T..........G................................T..........................

Majority    CGTCGTGTCGTTAGGACGGTGGTGTGCAGGCTAGTGCGTGGCCGGCACGTGGGGGGGA-GGATTGAGGTTTAATAGAATT
                     90        100       110       120       130       140       150       160
            ---------+---------+---------+---------+---------+---------+---------+---------+
mac-C12R1   ..........................................................-........C............
mac-C12R2   ...............................................T...A......-.....................
mac-C64R1   ..........................................................A.....................
mac-C91R1   ...................G................T.........G.........A.-........C............
mac-C91R2   ........T.........................G...........G...T.......C.-...................
mac-C133R1  ............................................T.............-......G..............

Majority    AAGAGTGCTAAGAATGAT
                     170
            ---------+--------
mac-C12R1   ..................   177 bp
mac-C12R2   .............T....   175 bp
mac-C64R1   ..................   178 bp
mac-C91R1   ..................   177 bp
mac-C91R2   ..................   176 bp
mac-C133R1  .............T....   177 bp
```

mac-C12R1	mac-C12R2	mac-C64R1	mac-C91R1	mac-C91R2	mac-C133R1	
***	94.9	97.8	97.8	94.4	96.1	mac-C12R1
	***	93.8	92.7	91.6	95.5	mac-C12R2
		***	95.5	93.8	94.9	mac-C64R1
			***	93.3	93.8	mac-C91R1
				***	92.7	mac-C91R2
					***	mac-C133R1

Similarity: 91.6-97.8

S. maglia, mag

```
Majority   CCTTTTTGTCGAAATTCGGTXGTGTAATTGAAGAAATATXTATTXATTTATTTTTTGCAGAAACGGCGTCGTTAGGACXX
                   10        20        30        40        50        60        70        80
           ---------+---------+---------+---------+---------+---------+---------+---------+
pSmag5S11  ....................C..................A....T.................................--
pSmag5S12  ....................C..................A....T.................................--
pSmag5S31  ....................C..................A....T.................................--
pSmag5S33  ....................C..................A....T.................................--
pSmag5S13  ....................A..................-AT..-.................................AG
pSmag5S21  ....................A..................-....-.................................AG
pSmag5S22  ....................A..................-....-.................................AG
pSmag5S23  ....................A..................-....-.................................AG

Majority   XXXXXGGGGGXGTTGAGGAXGGGCGTGAXXXXXXXXXXXXXXXXXXXXXXXXXXXXXXXXXXXXXXXXXXXGGGGGCAGG
                   90       100       110       120       130       140       150       160
           ---------+---------+---------+---------+---------+---------+---------+---------+
pSmag5S11  -----.....C........C........-------------------------------------------.........
pSmag5S12  -----.....C........C........-------------------------------------------.........
pSmag5S31  -----.....C........C........-------------------------------------------.........
pSmag5S33  -----.....C........C........-------------------------------------------.........
pSmag5S13  GTGAT.....A........T........CGGGCGGCGTCATGCGTCGGTGCGCGTGGAGGCTAGGTCGGTG.........
pSmag5S21  GTGAT.....A........T........CGGGCGGCGTCATGCGTCGGTGCGCGTGGAGGCTAGGTCGGTG.........
pSmag5S22  GTGAT.....A........T........CGGGCGGCGTCATGCGTCGGTGCGCGTGGAGGCTAGGTCGGTG...A.....
pSmag5S23  GTGAT.....A........T........CGGGCGGCGTCATGCGTCGGTGCGCGTGGAGGCTAGGTCGGTG...A.....

Majority   CXAGGGCGTTGGGAGGAAGGAXGTGTTTAATAGAATTTAGAGTGCTATGAATGAT
                  170       180       190       200       210
           ---------+---------+---------+---------+---------+-----
pSmag5S11  .C...................G.................................  165 bp
pSmag5S12  .C...................G.................................  165 bp
pSmag5S31  .C...................G.................................  165 bp
pSmag5S33  .C...................G......G..........................  165 bp
pSmag5S13  .T...................T.................................  213 bp
pSmag5S21  .T...................T.................................  213 bp
pSmag5S22  .T...................T....A............................  213 bp
pSmag5S23  .T...................T....A............................  213 bp
```

pSmag5S11	pSmag5S12	pSmag5S31	pSmag5S33	pSmag5S13	pSmag5S21	pSmag5S22	pSmag5S23	
***	100.0	100.0	99.5	72.6	73.5	72.6	72.6	pSmag5S11
	***	100.0	99.5	72.6	73.5	72.6	72.6	pSmag5S12
		***	99.5	72.6	73.5	72.6	72.6	pSmag5S31
			***	72.1	73.0	72.1	72.1	pSmag5S33
				***	99.1	98.1	98.1	pSmag5S13
					***	99.1	99.1	pSmag5S21
						***	100.0	pSmag5S22
							***	pSmag5S23

Similarity: 72.1-100

S. mammosum, mam

```
Majority    CCTTTTTGTGTCGAAATTCGGCATAATCTCGTCTGTCTGTGTTATTTTTGGCGGAAACGGCATTTGTTTGGGCCGGGACG
                     10        20        30        40        50        60        70        80
            ---------+---------+---------+---------+---------+---------+---------+---------+
pSmam-5S1   ................................................................................
pSmam-5S8   ................................................................................

Majority    TCGTTAGGACAGTTGAGGAAGGGCGTTGCCGCGCGGCGGTGCGTGGAGGCTAGGGCGGTGGCGXTGCAGGCTAGGGCGTC
                     90       100       110       120       130       140       150       160
            ---------+---------+---------+---------+---------+---------+---------+---------+
pSmam-5S1   ...............................................................G................
pSmam-5S8   ...............................................................A................

Majority    GGGAGGAATGAGGTTTAATAGAATTTAGAGTGGTAGGAATGAC
                    170       180       190       200
            ---------+---------+---------+---------+---
pSmam-5S1   ...........................................     203 bp
pSmam-5S8   ...........................................     203 bp
```

pSmam-5S1	pSmam-5S8	
***	99.5	pSmam-5S1
	***	pSmam-5S8

Similarity: 99.5

S. marinasense, mrn

```
Majority CCTTTTTGTCGAAATTCGGTCGTGTAATAGAAAAAATATTATTATTTATTTTTTGTAGAAACGACGTCGTTAGGACTAAC
                  10        20        30        40        50        60        70        80
         ---------+---------+---------+---------+---------+---------+---------+---------+
mrn-C1R1 ................................................................................
mrn-C1R2 ................................................................................
mrn-C1R4 .T..............................................................................
mrn-C2R2 ............................................................................----

Majority AGGTGATGGGGGCGTTGGGAGGATGGGCGTGACGGGCGGCGTCATGCGTCGGTGCGCGTGGAGGCTAGGTCGGTGGGGGG
                  90       100       110       120       130       140       150       160
         ---------+---------+---------+---------+---------+---------+---------+---------+
mrn-C1R1 ................................................................................
mrn-C1R2 .............-------------------------------------------------------------------
mrn-C1R4 ................................................................................
mrn-C2R2 ................................................................................

Majority CAGGCTAGGGCGTTGGGAGGAAGGAGGTGTTTAATAGAATTTAGAGTGCTATGAATGAT
                 170       180       190       200       210
         ---------+---------+---------+---------+---------+---------
mrn-C1R1 ...........................................................   219 bp
mrn-C1R2 -..........................................................   151 bp
mrn-C1R4 ......TTT..........T.......................................   219 bp
mrn-C2R2 ...........................................................   215 bp
```

mrn-C1R1	mrn-C1R2	mrn-C1R4	mrn-C2R2	
***	68.9	97.7	98.2	mrn-C1R1
	***	66.7	67.1	mrn-C1R2
		***	95.9	mrn-C1R4
			***	mrn-C2R2

Similarity: 66.7-98.2

S. medians, med

```
Majority  CTTTTTTGTCGAAATTCGGTCGTGTAATTGAAAAAATAT-ATTATTATTTTTTGCAGAAACGACATTCGGATTGAGACG
                   10        20        30        40        50        60        70        80
          ---------+---------+---------+---------+---------+---------+---------+---------+
med-C1R1  .C.......................G....GG.......TT....ATA.................--------------.
med-C1R2  .C.......................G....GG.......TT....ATA.................--------------.
med-C2R1  .C.................G.....G....GG.......TT....ATA.................--------------.
med-C2R2  .......................................-...............A........................
med-C4R1  .................................C.....-........................................
med-C2R3  .......................................-........................................
med-C8R1  .......................................-...............A........................
med-C8R2  .......................................-...............A........................
med-C8R3  .......................................-...T....................................
med-C8R4  .C....A...................G........G...T......TA................A--------------.
med-C8R5  .C....A...................G........G...T......TA................A--------------.

Majority  TCGTTAGGACAGGTGATGGGGGCGTTGAGGATGGGCGTGACGGGCGGCGTCATGCGTCGGTGCG--TGGAGGCTAGGTCG
                   90       100       110       120       130       140       150       160
          ---------+---------+---------+---------+---------+---------+---------+---------+
med-C1R1  ................................................................CG...G..........
med-C1R2  ................................................................CG...G..........
med-C2R1  ........................G.......................................CG..............
med-C2R2  ................................................................--......A.......
med-C4R1  ................................................................--..............
med-C2R3  ................................................................--..............
med-C8R1  ................................................................--..............
med-C8R2  ................................................................--..............
med-C8R3  ...................................................C............--..............
med-C8R4  ..........G............A....................A...----.AG.......-----..G...-...CT-
med-C8R5  ..........G.T...............................A...----.AG.......-----..G...-...CT-

Majority  GTGGGGG------GGAGGCTAGGGCGTTGG-AGGAAGGAGGTGTTTAATAGAATTTAGAGTGGTAGGAATGAT
                   170       180       190       200       210       220       230
          ---------+---------+---------+---------+---------+---------+---------+---
med-C1R1  .......------.C...............G.........................G.....C..T.......   213 bp
med-C1R2  .C.....------.C...............G............G............G.....C..T.......   213 bp
med-C2R1  .......------.C...............G.........................G.....C..T.......   213 bp
med-C2R2  .......TGGGGG.................-.........T................................   229 bp
med-C4R1  .......TGGGGG.................-.........T................................   229 bp
med-C2R3  .......------.................-..........................................   223 bp
med-C8R1  .......------.................-..........................................   223 bp
med-C8R2  .......------......G..........-..........................................   223 bp
med-C8R3  .......------.................-..........................................   223 bp
med-C8R4  ---------------...............G...............................C..T.......   193 bp
med-C8R5  ---------------...............G..............................AC..T.......   193 bp
```

med-C1R1	med-C1R2	med-C2R1	med-C2R2	med-C4R1	med-C2R3	med-C8R1	med-C8R2	med-C8R3	med-C8R4	med-C8R5	
***	99.1	98.7	82.8	83.3	86.7	86.3	85.8	85.8	84.1	83.7	med-C1R1
	***	97.9	82.0	82.4	85.8	85.4	85.0	85.0	83.7	83.3	med-C1R2
		***	82.4	82.8	86.3	85.8	85.4	85.4	82.8	82.4	med-C2R1
			***	98.7	96.1	96.6	96.1	95.3	74.2	73.8	med-C2R2
				***	96.6	96.1	95.7	95.7	74.7	74.2	med-C4R1
					***	99.6	99.1	99.1	78.1	77.7	med-C2R3
						***	99.6	98.7	77.7	77.3	med-C8R1
							***	98.3	77.3	76.8	med-C8R2
								***	77.7	77.3	med-C8R3
									***	98.7	med-C8R4
										***	med-C8R5

Similarity: 73.8-99.6

S. medicagineum, mdg

```
Majority   CTTTTTTCATCTGTTCGGCCGATGATTATTTATTTTTTTTTGGCGGTTGGGTCGTTTGCTTGGGCAGAGACGTCGTTAGG
                    10        20        30        40        50        60        70        80
           ---------+---------+---------+---------+---------+---------+---------+---------+
mdg-C1R1   ................................................................................
mdg-C2R1   ........................................G...........T...........................
mdg-C3R1   ................................................................................
mdg-C7R1   ...............................T................................................
mdg-C7R2   ........................................-.......................................
mdg-C10R1  ....................................C...........................................
mdg-C11R1  .....................................................A..........................

Majority   ACGGTTGAGGAAGGGCGTCGCCGGCGCGTGGAGGCTAGGGCGACGGTGTGCAGGAATGAGGTTTAATAGAATTAAGGGTG
                    90       100       110       120       130       140       150       160
           ---------+---------+---------+---------+---------+---------+---------+---------+
mdg-C1R1   ..................................G.............................................
mdg-C2R1   ................................................................................
mdg-C3R1   ................................................................................
mdg-C7R1   ................................................................................
mdg-C7R2   ..................................G.............................................
mdg-C10R1  ..................................G.............................................
mdg-C11R1  ................................................................................

Majority   CTAGGAATGAC
                   170
           ---------+-
mdg-C1R1   ...........   171 bp
mdg-C2R1   ...........   171 bp
mdg-C3R1   ...........   171 bp
mdg-C7R1   .........G.   171 bp
mdg-C7R2   ...........   170 bp
mdg-C10R1  ....A......   171 bp
mdg-C11R1  ..........T   171 bp
```

mdg-C1R1	mdg-C2R1	mdg-C3R1	mdg-C7R1	mdg-C7R2	mdg-C10R1	mdg-C11R1	
***	98.2	99.4	98.2	99.4	98.8	98.2	mdg-C1R1
	***	98.8	97.7	98.2	97.1	97.7	mdg-C2R1
		***	98.8	98.8	98.2	98.8	mdg-C3R1
			***	97.7	97.1	97.7	mdg-C7R1
				***	98.2	97.7	mdg-C7R2
					***	97.1	mdg-C10R1
						***	mdg-C11R1

Similarity: 97.1-99.4

S. megistacrolobum (= boliviense), mga

```
Majority  CCTTTTTGTCGAAATTCGGTCGTGCAATTGATTTTTATTATTATTTATCTTTTTGCAGAAACGGCGTCGTTAGGACAGG
                   10        20        30        40        50        60        70        80
          ---------+---------+---------+---------+---------+---------+---------+---------+
mga-C1R3  ..........................T...................C........A..........T.............
mga-C1R4  ..................................................................T.............
mga-C1R5  ..................................................................T.............
mga-C1R6  ..................................................................T.............
mga-C2R1  ................................................................................
mga-C2R2  .............C..................................................................
mga-C2R3  .T...................................T..........................................
mga-C1R1  ................................................................................
mga-C1R2  ................................................................................

Majority  TGATGG-------------GGGCGTGACGGGCGGCGTCGTGCGTCGGTGCGCGTGGAGGCTAGGTCCGGTGGGGGGCAGG
                   90       100       110       120       130       140       150       160
          ---------+---------+---------+---------+---------+---------+---------+---------+
mga-C1R3  ......GGGCGTTGAGGAT..............................G......G....G..G...............
mga-C1R4  ..G...GGGCGTTGAGGAT.............................................................
mga-C1R5  ......GGGCGTTGAGGAT.............................................................
mga-C1R6  ......TGGCGTTGATGAT...................A............................A............
mga-C2R1  ......-------------.............................................................
mga-C2R2  ......-------------.............................................................
mga-C2R3  ......-------------.............................................................
mga-C1R1  ......-------------.................G...........................................
mga-C1R2  ..G...-------------.....................................G.......................

Majority  CTAGGGCGTTGGGAGGAAGGAAAGGAGGTGTTTAAAAGAATTTAGAGTGCTATGAATGAT
                  170       180       190       200       210       220
          ---------+---------+---------+---------+---------+---------+
mga-C1R3  ...............-................G...........................    219 bp
mga-C1R4  ...............................CG...........................    220 bp
mga-C1R5  ................................G...........................    220 bp
mga-C1R6  ...............................C.............G..............    220 bp
mga-C2R1  ............................................................    207 bp
mga-C2R2  T...............................G...........................    207 bp
mga-C2R3  ...................................G........................    207 bp
mga-C1R1  ............................................................    207 bp
mga-C1R2  ............................................................    207 bp
```

mga-C1R3	mga-C1R4	mga-C1R5	mga-C1R6	mga-C2R1	mga-C2R2	mga-C2R3	mga-C1R1	mga-C1R2	
***	95.5	96.4	93.2	89.5	89.1	88.2	89.1	89.5	mga-C1R3
	***	99.1	96.8	92.3	91.8	90.9	91.8	92.3	mga-C1R4
		***	96.8	93.2	92.7	91.8	92.7	92.3	mga-C1R5
			***	91.8	90.5	90.5	91.4	90.9	mga-C1R6
				***	98.6	98.6	99.5	99.1	mga-C2R1
					***	97.3	98.2	97.7	mga-C2R2
						***	98.2	97.7	mga-C2R3
							***	98.6	mga-C1R1
								***	mga-C1R2

Similarity: 88.2-99.5

S. melongena-1, mel1

```
Majority    CTTTTTTTGCTGAAATTTCGTC----------------------------------------------------------
                    10        20        30        40        50        60        70        80
            ---------+---------+---------+---------+---------+---------+---------+---------+
pSmel1-5S11 ......................GTCTATTCGGCGAGTGATTATTTTTTTTGGCGGTTGGGTCGTTTGCTCGGGGAGAGAC
pSmel1-5S-1 ......................----------------------------------------------------------
pSmel1-5S-2 ......................----------------------------------------------------------

Majority    --------------------------------------------------------------------------------
                    90       100       110       120       130       140       150       160
            ---------+---------+---------+---------+---------+---------+---------+---------+
pSmel1-5S11 GTCGTTAAGACGGTTGAGGAAGGTCGTCGCGGTAGCAAAAAGGACCCCCTGGGAAGTCCTCGTGTTGCATCCCTCTTTTT
pSmel1-5S-1 --------------------------------------------------------------------------------
pSmel1-5S-2 --------------------------------------------------------------------------------

Majority    ----------------GTTTGCTCGGGGAATGATTATTTTTTTTCGTCGTTTGCTCGGGGAGAGACGTCGTTAGGACGGT
                   170       180       190       200       210       220       230       240
            ---------+---------+---------+---------+---------+---------+---------+---------+
pSmel1-5S11 TTGCTGAAATTTCGTC................................................................
pSmel1-5S-1 ----------------................................................................
pSmel1-5S-2 ----------------................................................................

Majority    TGAGGAAGGTCGTCGCGGTAGCAAAAAGGCCTGTGGCGGTGGTGTGCATGGCTAGGGCTAGGGCGTGGGGGGAGGAATGA
                   250       260       270       280       290       300       310       320
            ---------+---------+---------+---------+---------+---------+---------+---------+
pSmel1-5S11 ................................................................................
pSmel1-5S-1 ................................................................................
pSmel1-5S-2 ................................................................................

Majority    GGTTTAATAGAATTAAGAGTGCTAAGAATGAT
                   330       340       350
            ---------+---------+---------+--
pSmel1-5S11 ........................G.......        352
pSmel1-5S-1 ................................        198
pSmel1-5S-2 ................................        198
```

pSmel1-5S11	Smel-5S-1	Smel-5S-2	
***	56.0	56.0	pSmel1-5S11
	***	100.0	Smel-5S-1
		***	Smel-5S-2

Similarity: 56.0-99.6

S. melongena-2, mel2

```
Majority     CTTTTTTTGCTGAAATTTCGTCGTCTATTCGGCGAATGATTATTTTTTTTGGCGGTTGGGTCGTTTGCTCGGGGAGAGAC
                      10        20        30        40        50        60        70        80
             ---------+---------+---------+---------+---------+---------+---------+---------+
mel2-C1R1    ...................................G............................................
mel2-C3R1    ...................................G............................................
mel2-C22R1   ..................................T.............................................
mel2-C2R1    ................................................................................
mel2-C9R1    ................................................................................
mel2-C11R1   ...................A.................................A..........................
mel2-C12R1   ............T......................G............................................
mel2-C17R1   --------------------------------------------------------------------------------

Majority     GTCGTTAAGACGGTTGAGGAAGGTCGTCGCGGTAGCAAAAAGGACCCCCTGGGAAGTCCTCGTGTTGCATCCCTCTTTTT
                      90       100       110       120       130       140       150       160
             ---------+---------+---------+---------+---------+---------+---------+---------+
mel2-C1R1    ................................................................................
mel2-C3R1    ................................................................................
mel2-C22R1   ................................................................................
mel2-C2R1    ................................................................................
mel2-C9R1    ...............................................................................A
mel2-C11R1   .......................................T.....................C.................-
mel2-C12R1   ................................................................................
mel2-C17R1   --------------------------------------------------------------------------T.....

Majority     TTGCTGAAATTTCGTCGTCTATTCGGGGAATGATTATTTTTTTTGGCGGTTGGGTCGTTTGCTCGGGGAGAGACGTCGTT
                     170       180       190       200       210       220       230       240
             ---------+---------+---------+---------+---------+---------+---------+---------+
mel2-C1R1    ..................T.GC......................C.--------..........................
mel2-C3R1    ..................T.GC......................C.--------....................A.....
mel2-C22R1   ..................T.GC......................C.--------..........................
mel2-C2R1    ............................................................A...................
mel2-C9R1    .............A................................................................C.
mel2-C11R1   ................................................................................
mel2-C12R1   .............A.................................................T..............C.
mel2-C17R1   ....A......C..............C..........................................T..........

Majority     AGGACGGTTGAGGAAGGTCGTCGCGGTAGCAAAAAGGCCTGTGGCGGTGGTGTGCATGGCTAGGGCTAGGGCGTGGGGGG
                     250       260       270       280       290       300       310       320
             ---------+---------+---------+---------+---------+---------+---------+---------+
mel2-C1R1    ................................................................................
mel2-C3R1    ................................................................................
mel2-C22R1   ................................................................................
mel2-C2R1    ................................................................................
mel2-C9R1    ................................................................................
mel2-C11R1   ......A.........................................................................
mel2-C12R1   ................................................................................
mel2-C17R1   ..................G...AT........................................................

Majority     AGGAATGAGGTTTAATAGAATTAAGAGTGCTAGGAATGAT
                     330       340       350       360
             ---------+---------+---------+---------+
mel2-C1R1    ........................................      352 bp
mel2-C3R1    ........................................      352 bp
mel2-C22R1   ........................................      352 bp
mel2-C2R1    ........................................      360 bp
mel2-C9R1    ........................................      360 bp
mel2-C11R1   ........................................      359 bp
mel2-C12R1   ........................................      360 bp
mel2-C17R1   ........................................      206 bp
```

mel2-C1R1	mel2-C3R1	mel2-C22R1	mel2-C2R1	mel2-C9R1	mel2-C11R1	mel2-C12R1	mel2-C17R1	
***	99.7	99.4	96.1	95.6	94.7	95.6	51.7	mel2-C1R1
	***	99.2	95.8	95.3	94.4	95.3	51.4	mel2-C3R1
		***	96.1	95.6	94.7	95.0	51.7	mel2-C22R1
			***	98.9	98.1	98.3	54.7	mel2-C2R1
				***	97.8	98.9	54.2	mel2-C9R1
					***	96.9	54.4	mel2-C11R1
						***	54.2	mel2-C12R1
							***	mel2-C17R1

Similarity: 51.4-99.7

Note: mel2-C17R1 represents a sequence of rare ribotype M17 that contains no duplication in IGS.

S. melongena-3, mel3

```
Majority  CTTTTTTTGCTGAAATTTCGTCGTCTATTCGGCGAGTGATTATTTTTTTTGGCGGTTGXGTCGTTTGCTCGGGGAGAGAC
                   10        20        30        40        50        60        70        80
          ---------+---------+---------+---------+---------+---------+---------+---------+
mel3-C2R1 ........................ ..................................T.....................
mel3-C3R1 ........................T.GC....G..A..............------C.--....................
mel3-C4R1 ..........................................................G.....................

Majority  GTCGTTAAGACGGTTGAGGAAGGTCGTCGCGGTAGCAAAAAGGACCCCCTGGGAAGTCCTCGTGTTGCATCCCTCTTTTT
                   90        100       110       120       130       140       150       160
          ---------+---------+---------+---------+---------+---------+---------+---------+
mel3-C2R1 ................................................................................
mel3-C3R1 .......G........................................................................
mel3-C4R1 ................................................................................

Majority  TTGCTGAAATTTCGTCGTTTGCTCGGGGAATGATTATTTTTTTTCGTCGTTTGCTCGGGGAGAGACGTCGTTAGGACGGT
                   170       180       190       200       210       220       230       240
          ---------+---------+---------+---------+---------+---------+---------+---------+
mel3-C2R1 ................................................................................
mel3-C3R1 ................................................................................
mel3-C4R1 ................................................................................

Majority  TGAGGAAGGTCGTCGCGGTAGCAAAAAGGCCTGTGGCGGTGGTGTGCATGGCTAGGGCTAGGGCGTGGGGGGAGGAATGA
                   250       260       270       280       290       300       310       320
          ---------+---------+---------+---------+---------+---------+---------+---------+
mel3-C2R1 .....................................................G..........................
mel3-C3R1 ..G.............................................................................
mel3-C4R1 ................................................................................

Majority  GGTTTAATAGAATTAAGAGTGCTAGGAATGAT
                   330       340       350
          ---------+---------+---------+--
mel3-C2R1 ................................     352 bp
mel3-C3R1 ................................     344 bp
mel3-C4R1 ................................     352 bp
```

mel3-C2R1	mel3-C3R1	mel3-C4R1	
***	95.2	99.4	mel3-C2R1
	***	95.5	mel3-C3R1
		***	mel3-C4R1

Similarity: 95.2-99.4

S. microdontum-1

```
Majority        CCTTTTTGTCGAAATTCGGTAGTGTAATTGAAGAAATAT-TATT-ATTTATTTTTTGCAGAAACGGCGTCGTTAGGACAG
                         10        20        30        40        50        60        70        80
                ---------+---------+---------+---------+---------+---------+---------+---------+
mcd_51.1_5S.1   -.C....................................-....-...................................
mcd_51.1_5S.2   .......................................-....-..................-C...............
mcd_51.1_5S.3   .......................................-....-...................................
mcd_51.1_5S.5   .......................................-....-...................................
mcd_51.2_5S.5   .......................................-....-..................-................
mcd_51.2_5S.1   ....................C..................A....T.................................--
mcd_51.2_5S.2   ....................C..................A....T.................................--
mcd_51.2_5S.3   ....................C..................A....T.................................--
mcd_51.2_5S.4   ....................C..................A....T.................................--

Majority        GTGATGGGGGAGTTGAGGATGGGCGTGACGGGCGGCGTCATGCGTCGGTGCGCGTGGAGGCTAGGTCGGTGGGGGGCAGG
                         90       100       110       120       130       140       150
                ---------+---------+---------+---------+---------+---------+---------+---------+
mcd_51.1_5S.1   ................................................................................
mcd_51.1_5S.2   ................................................................................
mcd_51.1_5S.3   ................................................................................
mcd_51.1_5S.5   ................................................................................
mcd_51.2_5S.5   ................................................................................
mcd_51.2_5S.1   -----.....C........C............................................................
mcd_51.2_5S.2   -----.....C........C............................................................
mcd_51.2_5S.3   -----...A.C........C............................................................
mcd_51.2_5S.4   -----...A.C........C............................................................

Majority        CTAGGGCGTTGGGAGGAAGGATGTGTTTAATAGAATTTAGAGTGCTATGAATGAT
                        170       180       190       200       210
                ---------+---------+---------+---------+---------+-----
mcd_51.1_5S.1   .......................................................     212 bp
mcd_51.1_5S.2   .......................................................     212 bp
mcd_51.1_5S.3   .......................................................     213 bp
mcd_51.1_5S.5   .......................................................     213 bp
mcd_51.2_5S.5   .......................................................     212 bp
mcd_51.2_5S.1   .C...................G.......................A........C     208 bp
mcd_51.2_5S.2   .C...................G..........A............A........C     208 bp
mcd_51.2_5S.3   .C...................G.......................A........C     208 bp
mcd_51.2_5S.4   .C...................G.......................A........C     208 bp
```

mcd_51.1 _5S.1	mcd_51.1 _5S.2	mcd_51.1 _5S.3	mcd_51.1 _5S.5	mcd_51.2 _5S.5	mcd_51.2 _5S.1	mcd_51.2 _5S.2	mcd_51.2 _5S.3	mcd_51.2 _5S.4	
***	98.1	99.1	99.1	98.6	91.6	91.2	91.2	91.2	mcd_51.1 _5S.1
	***	99.1	99.1	99.5	91.6	91.2	91.2	91.2	mcd_51.1 _5S.2
		***	100.0	99.5	92.6	92.1	92.1	92.1	mcd_51.1 _5S.3
			***	99.5	92.6	92.1	92.1	92.1	mcd_51.1 _5S.5
				***	92.1	91.6	91.6	91.6	mcd_51.2 _5S.5
					***	99.5	99.5	99.5	mcd_51.2 _5S.1
						***	99.1	99.1	mcd_51.2 _5S.2
							***	100.0	mcd_51.2 _5S.3
								***	mcd_51.2 _5S.4

Similarity: 91.2-100

S. microdontum-2, mcd2

```
Majority   CCTTTTTGTCGAAATTTGGTCGTGTAATTGAAAAAATAT-TATT-ATTTATTTTTTGCAGGAACGACGTCGTTAGGACAG
                    10        20        30        40        50        60        70        80
           ---------+---------+---------+---------+---------+---------+---------+---------+
mcd2-C1R1  .......................................-....-...................................
mcd2-C1R2  .......................................-....-...........A.......................
mcd2-C2R1  .......................................-....-...................................
mcd2-C2R2  .T.....................................-....-...................................
mcd2-C2R3  ...................................C...-....-...................................
mcd2-C1R3  ..C.............C...............G......A....T...............A....G.......GA.----
mcd2-C1R4  ................C...............G......A....T...............A....GA......-A.----

Majority   GTGATGGGGGCGTTGAGGATGGGCGTGACGGGCGGCGTCATGCGTCGGTGCGCGTGGAGGCTAGGTCGGTGGGGGGCAGG
                    90       100       110       120       130       140       150       160
           ---------+---------+---------+---------+---------+---------+---------+---------+
mcd2-C1R1  .........................................C......................................
mcd2-C1R2  ................................................................................
mcd2-C2R1  ..............................T....G............................................
mcd2-C2R2  ................................................................................
mcd2-C2R3  ................................................................................
mcd2-C1R3  --..C..............C........-------------------------------------------.........
mcd2-C1R4  --..C..............C........-------------------------------------------.........

Majority   CTAGGGCGTTGGGAGGAAGGAGGTGTTTAATAGAATTTAGAGTGCTATGAATGAT
                   170       180       190       200       210
           ---------+---------+---------+---------+---------+-----
mcd2-C1R1  ..................................................G....     213 bp
mcd2-C1R2  .G..........T........A...............................G.     213 bp
mcd2-C2R1  .......................................................     213 bp
mcd2-C2R2  .....................A.................................     212 bp
mcd2-C2R3  .....................A......G..........................     213 bp
mcd2-C1R3  ............................G..........................     166 bp
mcd2-C1R4  .C.........A................G..........................     165 bp
```

mcd2-C1R1	mcd2-C1R2	mcd2-C2R1	mcd2-C2R2	mcd2-C2R3	mcd2-C1R3	mcd2-C1R4	
***	96.7	98.1	98.1	97.7	71.2	70.2	mcd2-C1R1
	***	96.7	97.7	97.2	69.3	68.8	mcd2-C1R2
		***	98.1	97.7	71.6	70.7	mcd2-C2R1
			***	98.6	70.7	69.8	mcd2-C2R2
				***	71.2	70.2	mcd2-C2R3
					***	97.7	mcd2-C1R3
						***	mcd2-C1R4

Similarity: 68.8-98.6

S. multiinterruptum, mtp

```
Majority CTTTTTTGTTGAAATTCGGTCGTGTAATATTTAAATATTATATTATTTTTCTGCAGAAACGACATTCGGATTGAGACGTC
                10        20        30        40        50        60        70        80
         ---------+---------+---------+---------+---------+---------+---------+---------+
mtp-C1R1 ................................................................................
mtp-C1R2 ................................................................................
mtp-C1R4 ..G.............................................................................
mtp-C1R5 ................................................................................

Majority GTTAGGACAGGTGATGGGGGCGTTGAGGATGGGCGTGACGGGCGGCGTCATGCGTCGGTGCGTGGAGGCTAGGTCGGTGG
                90       100       110       120       130       140       150       160
         ---------+---------+---------+---------+---------+---------+---------+---------+
mtp-C1R1 ................................................................................
mtp-C1R2 ................................................................................
mtp-C1R4 ................................................................................
mtp-C1R5 ..............A...............T.....................A...........................

Majority GGGGCAGGCTAGGGCGTTGGAGGAAGGAGGTGTTXAATAGAATTTAGAGTGCTATGAATGAT
               170       180       190       200       210       220
         ---------+---------+---------+---------+---------+---------+--
mtp-C1R1 .........A........................T..C........................    222 bp
mtp-C1R2 ..................................T...........................    222 bp
mtp-C1R4 ................................CCC...........................    222 bp
mtp-C1R5 ..................................C...........................    222 bp
```

mtp-C1R1	mtp-C1R2	mtp-C1R4	mtp-C1R5	
***	99.1	97.3	97.3	mtp-C1R1
	***	98.2	98.2	mtp-C1R2
		***	97.3	mtp-C1R4
			***	mtp-C1R5

Similarity: 97.3-99.1

S. muricatum , mur

```
Majority    TTTTTCCGATCGAAATTCGTCATAATTTCGTGTATTCGATAATATTTATTTATATTTTTTTGCGGAAACXTCATTCTGAT
                     10        20        30        40        50        60        70        80
            ---------+---------+---------+---------+---------+---------+---------+---------+
pSmur-5S5   .....................................................................A..........
pSmur-5S15  .....................................................................G..........

Majority    CGAGACGTCGTTAGGACCTGTGAGGGGGGCGTGACCGGCGGCGTTGXCTGGAGGGTCGGTCGGTAGGGGTGCAGGATAGG
                     90       100       110       120       130       140       150       160
            ---------+---------+---------+---------+---------+---------+---------+---------+
pSmur-5S5   .............................................C..................................
pSmur-5S15  .............................................T..................................

Majority    GCGTTGGGAGGAAGGAGGTGTTTAATAAGATTAGAATGCAATTACTGAT
                    170       180       190       200
            ---------+---------+---------+---------+---------
pSmur-5S5   .................................................     209 bp
pSmur-5S15  .................................................     209 bp
```

pSmur-5S5	pSmur-5S15	
***	99.0	pSmur-5S5
	***	pSmur-5S15

Similarity: 99.0

S. neorickii, neo

```
Majority  CCTTTTTGTTGAAATTTGATCTCGTAATTGAAAAAAAAA-TATACTAATTTATTTTTTTT-GCGGAAAATACGTTCGGAT
                   10        20        30        40        50        60        70        80
          ---------+---------+---------+---------+---------+---------+---------+---------+
neo-C1R1  ......................................--...........-........-...................
neo-C2R1  .......................................A......C.............T...................
neo-C3R1  .......................................-....................-...............A...
neo-C4R1  .......................................-....................-...................
neo-C5R1  ......................................--....................-...................

Majority  TGAGACGTCATTAGGATATGGGATGGTGGCGTCGGGGATGGGCGTGACGGGCGTCGTCGTGCGTCGGTGCGTGGAGGGTT
                   90        100       110       120       130       140       150       160
          ---------+---------+---------+---------+---------+---------+---------+---------+
neo-C1R1  ................................A...............................................
neo-C2R1  ....G...........................................................................
neo-C3R1  .................................................A..........................T...
neo-C4R1  ................................................................................
neo-C5R1  .................................................A..............................

Majority  TTAAAGCGGGGGGCGGGCTAGGGCGTTGGGAGGAAGGTTGTGTTTAATAGATATTAGAGTGCAATGAATGAC
                   170       180       190       200       210       220       230
          ---------+---------+---------+---------+---------+---------+---------+----
neo-C1R1  ............................................A.......T...................   228 bp
neo-C2R1  .............G..........................................................   232 bp
neo-C3R1  ........................................................................   230 bp
neo-C4R1  ...................G................................T...................   230 bp
neo-C5R1  ........................................................................   229 bp
```

neo-C1R1	neo-C2R1	neo-C3R1	neo-C4R1	neo-C5R1	
***	95.7	96.6	97.9	97.9	neo-C1R1
	***	96.6	97.0	97.0	neo-C2R1
		***	97.9	98.7	neo-C3R1
			***	98.3	neo-C4R1
				***	neo-C5R1

Similarity: 95.7-98.7

S. neorossii, nrs

DIRECT SEQUENCING - 213 bp

S. nigrum, nig

```
Majority    CCTTTTTGTCGAAGTTCGGCATGATTTCGTCTATTTGATAATATATATTATTATTTTTTTGCAGAAACGGCATTCGTGCC
                     10        20        30        40        50        60        70        80
            ---------+---------+---------+---------+---------+---------+---------+---------+
nig-C1R1    ................................................................................
nig-C3R1    ...............................................................A................
nig-C4R1    ....................................................................T...........
nig-C35R1   ................................................................................

Majority    TAGACGTCGCTAGGACGGGTGACGGAGGCGCTGAGGACGGGCGTGACAGGCATGCCGTCGGTGCGTGGAGGCTXGGGAGG
                     90       100       110       120       130       140       150       160
            ---------+---------+---------+---------+---------+---------+---------+---------+
nig-C1R1    .........................................................................A......
nig-C3R1    A........................................................................A......
nig-C4R1    .........................................................................G......
nig-C35R1   .........................................................................G......

Majority    CGGGGGACATGCTATGTCGTTGGGAGGAAGGAGGTGTTTAATAGAATTTAGAGCGCAATGAATGAT
                    170       180       190       200       210       220
            ---------+---------+---------+---------+---------+---------+------
nig-C1R1    ..................................................................     226 bp
nig-C3R1    ..................................................................     226 bp
nig-C4R1    .T................................................................     226 bp
nig-C35R1   ..................................................................     226 bp
```

nig-C1R1	nig-C3R1	nig-C4R1	nig-C35R1	
***	99.1	98.7	99.6	nig-C1R1
	***	97.8	98.7	nig-C3R1
		***	99.1	nig-C4R1
			***	nig-C35R1

Similarity: 97.8-99.6

S. okadae-1, oka1

```
Majority     CCTTTTTGTCGAAATTCGGTCGTGTAATTGAAGAAATGAAATATATTAATATTTATTTTTTGCAGAAAGGGCGTCGTTAG
                      10        20        30        40        50        60        70        80
             ---------+---------+---------+---------+---------+---------+---------+---------+
oka_I_5S.1   ................................................................................
oka_I_5S.2   ................................................................................
oka_I_5S.3   ...............................T................................................

Majority     GACAGGCGATGGGGGCGTTGAGGATGGGCGTGACGGGCGGCGTCATGCGTCGGTGCGCGTGGAGGCTAGGTCGGTGGGGG
                      90       100       110       120       130       140       150       160
             ---------+---------+---------+---------+---------+---------+---------+---------+
oka_I_5S.1   ...........................................................................AA...
oka_I_5S.2   ................................................................................
oka_I_5S.3   ................................................................................

Majority     GCAGGCTAGGGCGTTGGGAGGAAGGAGGTGTTTAATAGAATTTAGAGTGCTACGAATGAT
                     170       180       190       200       210       220
             ---------+---------+---------+---------+---------+---------+
oka_I_5S.1   .A...........C.................C..........G.........T.......    220 bp
oka_I_5S.2   ............................................................    220 bp
oka_I_5S.3   ............................................................    220 bp
```

oka_I_5S.1	oka_I_5S.2	oka_I_5S.3	
***	96.8	96.4	oka_I_5S.1
	***	99.5	oka_I_5S.2
		***	oka_I_5S.3

Similarity: 96.4-99.5

S. okadae-2, oka2

```
Majority        CCTTTTTGTCGAAATTCGGTCGAGTAATTGAAGAAATGAAATATATTAATATTTATTTTTTGCAGAAAGGGCGTCGTTAG
                         10        20        30        40        50        60        70        80
                ---------+---------+---------+---------+---------+---------+---------+---------+
oka_II_5S.1     .T....................T.........................................................
oka_II_5S.2     ................T...............A...............................................
oka_II_5S.3     ..............-------------------------------------------------------...........
oka_II_5S.5     ................................................................................

Majority        GACAGGTGATGGGGGCGTTGAGGATGGGCGTGACGGGCGGCGTCATGCGACGGTGCGCGTGGAGGCTAGGTCGGTGGGGG
                         90       100       110       120       130       140       150       160
                ---------+---------+---------+---------+---------+---------+---------+---------+
oka_II_5S.1     ................................................................................
oka_II_5S.2     ......C.........................................................................
oka_II_5S.3     ................................................................................
oka_II_5S.5     .....T..........................................................................

Majority        GGAGGATAGGGCGTTGGGAGGAAGGAGGTGTTTAATAGAATTTAGAGTGCTATGAATGAC
                        170       180       190       200       210       220
                ---------+---------+---------+---------+---------+---------+
oka_II_5S.1     ............................................................   220 bp
oka_II_5S.2     .C...C.............A...............................G.......T   220 bp
oka_II_5S.3     ............................................................   165 bp
oka_II_5S.5     ............................................................   220 bp
```

oka_II_5S.1	oka_II_5S.2	oka_II_5S.3	oka_II_5S.5	
***	95.5	74.5	98.6	oka_II_5S.1
	***	72.3	95.9	oka_II_5S.2
		***	74.5	oka_II_5S.3
			***	oka_II_5S.5

Similarity: 72.3-98.6

S. okadae-3, oka3

```
Majority   CCTTTTTGTCGAAATTCGGTCGTGCAATATATTTATTTATTTATTGCAGAAAAGGCGTCGTTAGGACAGGTGATGGGGGC
                    10        20        30        40        50        60        70        80
           ---------+---------+---------+---------+---------+---------+---------+---------+
oka3-C1R1  ................................................................................
oka3-C1R2  ....C...........................................................................
oka3-C1R3  ...............................................................................T

Majority   GTTGAGGATGGGCGTGACGGGCGGCGTCATGCGACGGTGCGCGTGGAGGCTAGGTCGGTGGGGGGCAGGATAGGGCGTTG
                    90        100       110       120       130       140       150       160
           ---------+---------+---------+---------+---------+---------+---------+---------+
oka3-C1R1  .................................................................G..............
oka3-C1R2  ................................................................................
oka3-C1R3  ................................................................-...............

Majority   GGAGGAAGGAGGTGTTTAATAGAATTTAGAGTGCTAGGAATGAC
                    170       180       190       200
           ---------+---------+---------+---------+----
oka3-C1R1  ....................................T.....G.    204 bp
oka3-C1R2  ............................................    204 bp
oka3-C1R3  ............G...............................    203 bp
```

oka3-C1R1	oka3-C1R2	oka3-C1R3	
***	98.0	97.1	oka3-C1R1
	***	98.0	oka3-C1R2
		***	oka3-C1R3

Similarity: 97.1-98.0

S. ossicruentum, oss

```
Majority   CCTTTTTTGCCGAAATTTCXTCGTCTATTCAGCGAATGATTTTTT-TTTGGCGGTTGGGTCGTCTGCTTGGGCAGAGAC
                    10        20        30        40        50        60        70        80
           ---------+---------+---------+---------+---------+---------+---------+---------+
oss-C1R1   ...................A.........................-........................A.-.......
oss-C3R1   .T.......T.........G------------.............T...T..........AG.........T........
oss-C10R1  ...................A------------A............G...T..........AG.........T........
oss-C15R1  ...................G.........................-..................................
oss-C20R1  ...................A.........................-..................................
oss-C26R1  ...................G.........................-.......................-..........

Majority   GCCGTAAGGACGGTTGAGGAAGGGCGTCGCCGGCGCGTGGAGGCTAGGGCGGTGGTGTGCGGGXTATGGCGTTGGGGGAG
                    90       100       110       120       130       140       150       160
           ---------+---------+---------+---------+---------+---------+---------+---------+
oss-C1R1   ..................................A................A...........G................
oss-C3R1   .....T............................................A............C................
oss-C10R1  .....T.........................................................C................
oss-C15R1  ...............................................................C................
oss-C20R1  ...............................................................G................
oss-C26R1  .............................A.................................G.G.A............

Majority   GAATGAGGTTTAATAGAXTTAAGAGTGCTAGGAATGAT
                   170       180       190
           ---------+---------+---------+--------
oss-C1R1   .................A............T.......     196 bp
oss-C3R1   .................-....................     185 bp
oss-C10R1  .................-....................     185 bp
oss-C15R1  .................-....................     196 bp
oss-C20R1  .................A............T.......     197 bp
oss-C26R1  .................A...G................     196 bp
```

oss-C1R1	oss-C3R1	oss-C10R1	oss-C15R1	oss-C20R1	oss-C26R1	
***	85.4	86.9	96.0	98.0	94.4	oss-C1R1
	***	97.0	89.4	87.4	85.9	oss-C3R1
		***	89.9	88.9	86.4	oss-C10R1
			***	98.0	96.5	oss-C15R1
				***	96.5	oss-C20R1
					***	oss-C26R1

Similarity: 85.4-98.0

S. pachyandrum, pac

```
Majority   CTTTTTTGTCGAAATTCGGCATAATTTCGTCXATTCGATXGTAT-TTTTTCGCAGAAACGACXTTTGAGCGGAXACGTCG
                  10        20        30        40        50        60        70        80
           ---------+---------+---------+---------+---------+---------+---------+---------+
pac-C1R1   .....................C.........T.......T....T......AA.........A....G....GG......
pac-C2R1   ......-....T...............T...A...-...G....-.................G..........C......
pac-C11R1  .................AA...........TT.......CA...A.....T...........A........A.G......
pac-C30R1  .................AA...........TT.......CA...A.....T...........A........A.G......
pac-C33R1  ...............................A.......T....-......A......T...G...A......C......
pac-C39R1  ......-....T...................A...-A..G....-.................G..........C..T...

Majority   TTAGGACAGGCXCTGAGGATGGGCGTGTCXGGCGTGACCACGCGCCGGTGCGTGGGGGCTAGTGCGGTGTGGTGCAGGCT
                  90       100       110       120       130       140       150       160
           ---------+---------+---------+---------+---------+---------+---------+---------+
pac-C1R1   ...........G.....T...........G.....C..........................G......G....T.....
pac-C2R1   ...........A.................A..........T.......................................
pac-C11R1  ..........TGA...T.......A....G...A............................................T.
pac-C30R1  ..........TGA...T.......A....G................................................T.
pac-C33R1  ......A....A........A........A.....................A.....T......TA..............
pac-C39R1  ...........A.................A................A..........T......T...............

Majority   AGGGCGTTGGGAGGAATGAGGTTTAATAGAATTTAGAGTGCTAXGAATGAT
                 170       180       190       200       210
           ---------+---------+---------+---------+---------+-
pac-C1R1   ............AA.........C.............AAT...G.......     211 bp
pac-C2R1   ............................C..............G..G....     208 bp
pac-C11R1  ................A.........A................A.....C.     211 bp
pac-C30R1  ..........................A................A.....T.     211 bp
pac-C33R1  ...............T............C..............G..G....     210 bp
pac-C39R1  .....A...............................A.....A.......     208 bp
```

pac-C1R1	pac-C2R1	pac-C11R1	pac-C30R1	pac-C33R1	pac-C39R1	
***	85.8	83.9	84.8	84.8	84.8	pac-C1R1
	***	85.8	86.7	92.4	94.3	pac-C2R1
		***	98.6	83.4	84.8	pac-C11R1
			***	84.4	85.8	pac-C30R1
				***	90.5	pac-C33R1
					***	pac-C39R1

Similarity: 83.4-98.6

S. palustre (= brevidens)-1, pal1

```
Majority   ----------------------------TGAA--------------------------------------TGAT------
                    10        20        30        40        50        60        70        80
           ---------+---------+---------+---------+---------+---------+---------+---------+
brev_5S.1  ----------------------------....--------------------------------------....------
brev_5S.2  ----------------------------....--------------------------------------....------
brev_5S.3  ----------------------------....--------------------------------------....------
brev_5S.6  ----------------------------....--------------------------------------....------
brev_5S.4  ACTTTTTGTAGAAATTCGGTCGTGTATT....AAATATATATATATTTTTTGCAGAAACGACATTTGGAT....CGGTCG
brev_5S.5  ACTTTTTGTAGAAATTCGGTCGTGTATT....AAATATATATATATTTTTTGCAGAAACGACATTTGGAT....CGGTCG

Majority-GTAGGACAGGTGACGGGGGCGCTTAGGATGGGCGTGAAAGGCGGCGTCAAGCGTCGGTGCGTGGAGGCTAGGTCGGTGG
                    90       100       110       120       130       140       150       160
           ---------+---------+---------+---------+---------+---------+---------+---------+
brev_5S.1  -...............................................................................
brev_5S.2  -...................................................G...........................
brev_5S.3  -..............................A................................................
brev_5S.6  -...............................................................................
brev_5S.4  T...............................................................................
brev_5S.5  T...............................................................................

Majority   GGGGCAGGCTAGGGCGTTGGGAGGAAGGAGGTGTTTAATAGAATTTAGAGTGCTATGAATGAT
                   170       180       190       200       210       220
           ---------+---------+---------+---------+---------+---------+---
brev_5S.1  ...............................................................    150 bp
brev_5S.2  ........T......................................................    150 bp
brev_5S.3  ...............................................................    150 bp
brev_5S.6  ...............................................................    150 bp
brev_5S.4  ...............................................................    223 bp
brev_5S.5  ...............................................................    223 bp
```

brev_5S.1	brev_5S.2	brev_5S.3	brev_5S.6	brev_5S.4	brev_5S.5	
***	99.1	99.6	100.0	67.3	67.3	brev_5S.1
	***	98.7	99.1	66.4	66.4	brev_5S.2
		***	99.6	66.8	66.8	brev_5S.3
			***	67.3	67.3	brev_5S.6
				***	100.0	brev_5S.4
					***	brev_5S.5

Similarity: 66.4-100

S. palustre (= brevidens)-2, pal2

```
Majority        ACTTTTTGTAGAAATTCGGTCGTGTATTTGAAAAATATATATATATXXTTTTTGCAGAAACGACATTCGGATTGATCGGT
                         10        20        30        40        50        60        70        80
                ---------+---------+---------+---------+---------+---------+---------+---------+
pal2-C1R1       ..............................................--....................A...........
pal2-C1R2       ...................................-..........AT................................
pal2-C1R3       ..............................................--................................
pal2-C1R4       ..............................................AT................................

Majority        CGTGTAGGACAGGTGACGGGGGCGCTTAGGATGGGCGTGAAAGGCGGCGTCAAGCGTCGGTGCGTGGAGGCTAGGTCGGT
                         90       100       110       120       130       140       150       160
                ---------+---------+---------+---------+---------+---------+---------+---------+
pal2-C1R1       ...................................G............................................
pal2-C1R2       ................................................................................
pal2-C1R3       .............................................................................A..
pal2-C1R4       .............................................A..................................

Majority        GGGGGGCAGGCTAGGGCGTTGGGAGGAAGGAGGTGTTTAATAGAATTTAGAGTGCTATGAATGAT
                        170       180       190       200       210       220
                ---------+---------+---------+---------+---------+---------+-----
pal2-C1R1       .....................................................C...........    223 bp
pal2-C1R2       .................................................................    224 bp
pal2-C1R3       .................................................................    223 bp
pal2-C1R4       .................................................................    225 bp
```

pal2-C1R1	pal2-C1R2	pal2-C1R3	pal2-C1R4	
***	97.3	98.2	97.3	pal2-C1R1
	***	98.2	99.1	pal2-C1R2
		***	98.2	pal2-C1R3
			***	pal2-C1R4

Similarity: 97.3-99.1

S. pampasense, pam

```
Majority  CCTTTTTGTCGAAATTCGGTCGTGTAATAGAAAAAATATTATTATTTATTTTTTGTAGAAACGACGT-CGTTAGGACAGG
                   10        20        30        40        50        60        70        80
          ---------+---------+---------+---------+---------+---------+---------+---------+
pam-C1R1  ......C...........................................................-...........A
pam-C1R2  ............................T.........-......AT.......C.........A.T..GATT...G..
pam-C1R3  ...................A...............................................-............
pam-C1R4  ........................C...T......GA...........A......C...........-..----------
pam-C3R1  .......................................C...........................-............

Majority  TGATGGGGGCGTTGAGGATGGGCGTGACGGGCGGCGTCATGCGTCGGTGCGCGTGGAGGCTAGGTCGGTGGGGGGCAGGC
                   90        100       110       120       130       140       150       160
          ---------+---------+---------+---------+---------+---------+---------+---------+
pam-C1R1  ...................................................T............................
pam-C1R2  ...................................................--...........................
pam-C1R3  .......................................A........................................
pam-C1R4  ----............................................................A.........-.....
pam-C3R1  .................C..................G.......G.C....G.........C..................

Majority  TAGGGCGTTGGGAGGAAGGAGGTGTTTAATAGAATTTAGAGTGCTATGAATGAT
                   170       180       190       200       210
          ---------+---------+---------+---------+---------+----
pam-C1R1  .......G.............................................    213 bp
pam-C1R2  C.....................A.........G................G...    211 bp
pam-C1R3  ............C........................................    213 bp
pam-C1R4  .....................................................    199 bp
pam-C3R1  .....................................................    213 bp
```

pam-C1R1	pam-C1R2	pam-C1R3	pam-C1R4	pam-C3R1	
***	90.2	96.7	88.8	95.3	pam-C1R1
	***	90.2	85.5	89.3	pam-C1R2
		***	88.8	95.3	pam-C1R3
			***	86.9	pam-C1R4
				***	pam-C3R1

Similarity: 85.5-96.7

S. paposanum, pap

```
Majority CCTTTTTGTCGAAATTCGGCATAATTTCGTCTATTTGATAATATATATTTTTTTGCAGAAACGACATTCGGXCGGGGACG
                 10        20        30        40        50        60        70        80
         ---------+---------+---------+---------+---------+---------+---------+---------+
pap-C1R1 .......................................................................G........
pap-C1R3 .....................G.................................................T........
pap-C2R1 ..............................G........................................T........
pap-C2R2 ............................................................G..........G........

Majority TCGTTAGGACAGGTGACGGAGGGGCTTGGGATGGGCGTGACAGGCGGCACCCACGCGTCGGCGAGTGCAGGCTAGGGCGG
                 90       100       110       120       130       140       150       160
         ---------+---------+---------+---------+---------+---------+---------+---------+
pap-C1R1 ................................................................................
pap-C1R3 ................................................................................
pap-C2R1 ........................G.......................................................
pap-C2R2 ................................................................................

Majy TGGGGGACAGGCTAGGGCGTTGGGAGGAGGGAGGTGTTTAATAGAATTTAGAGCGCTAGGAATGAT
                170       180       190       200       210       220
         ---------+---------+---------+---------+---------+---------+------
pap-C1R1 ..................................................................   226 bp
pap-C1R3 ..................................................................   226 bp
pap-C2R1 ..................................................................   226 bp
pap-C2R2 ....................G.............................................   226 bp
```

pap-C1R1	pap-C1R3	pap-C2R1	pap-C2R2	
***	99.1	98.7	99.1	pap-C1R1
	***	98.7	98.2	pap-C1R3
		***	97.8	pap-C2R1
			***	pap-C2R2

Similarity: 97.8-99.1

S. paucissectum, pcs

```
Majority  CCTTTTGGTCGAAATTCGGTCGAGTGATTGAAAAAATATATTATTATTTTTTGCAGAAACGACATTCGGATTGAGACGTC
                  10        20        30        40        50        60        70        80
          ---------+---------+---------+---------+---------+---------+---------+---------+
pcs-C1R1  ................................................................................
pcs-C2R1  ..............................................................................C.
pcs-C2R2  ................................................................................
pcs-C2R3  ..................................................C.............................
pcs-C3R1  ......................................................T.........................

Majority  GTTAGGACGGGTGATGGGGGXGTTGAGGATGGGCGTGACGGGCGGCGTCATGCGTCGGTGCGTGGAGGCTAGGCCGGTGG
                  90       100       110       120       130       140       150       160
          ---------+---------+---------+---------+---------+---------+---------+---------+
pcs-C1R1  ....................T...........................................................
pcs-C2R1  ....................T...........................................................
pcs-C2R2  ......-------------------------------------------------------------------.......
pcs-C2R3  ....................C...........................................................
pcs-C3R1  ....................C...........................................................

Majority  GGGTCAGGCTAGGGCGTTGGGAGGAAGGAGGTGTTTAATAGAATTTAGAGTGCAATGAATGAT
                 170       180       190       200       210       220
          ---------+---------+---------+---------+---------+---------+---
pcs-C1R1  ...............................................................    223 bp
pcs-C2R1  .....................G.........................................    223 bp
pcs-C2R2  ...............................................................    156 bp
pcs-C2R3  ..............................................G................    223 bp
pcs-C3R1  ......................................................G........    223 bp
```

pcs-C1R1	pcs-C2R1	pcs-C2R2	pcs-C2R3	pcs-C3R1	
***	99.1	70.0	98.7	98.7	pcs-C1R1
	***	69.1	97.8	97.8	pcs-C2R1
		***	69.1	69.1	pcs-C2R2
			***	98.2	pcs-C2R3
				***	pcs-C3R1

Similarity: 69.1-99.1

S. pennellii, pen

```
Majority   CCTTTTCGTTGAAATTCGATCTCGTAATTGAAAAAAATATACTCATCTATTTATTTTTGCGGAAAATACGTTCGGATTGA
                   10        20        30        40        50        60        70        80
           ---------+---------+---------+---------+---------+---------+---------+---------+
pen-C1R1   ................................................................................
pen-C1R2   .....................................C..........................................
pen-C1R3   ................A............T..................................................
pen-C1R4   ...................A............................................................

Majority   TGCGTCATTAGGATATGGGATGGTGGCGTCGGGGATGGGCGTGACGGGCGTCGTCGTGCGTCGGTGCGTGGAGGGTTTTA
                   90       100       110       120       130       140       150       160
           ---------+---------+---------+---------+---------+---------+---------+---------+
pen-C1R1   ..............................A.................................................
pen-C1R2   ................................................................................
pen-C1R3   ............................G...................................................
pen-C1R4   ....G................T..........................................................

Majority   AAGCGGGGGGCGAGCTAGGGCGTTGGGAGGAAGGTTGTGTTTAATAGATTTTAGAGTGCAATGAATGAC
                  170       180       190       200       210       220
           ---------+---------+---------+---------+---------+---------+---------
pen-C1R1   .....................................................................    229 bp
pen-C1R2   .....................................................................    229 bp
pen-C1R3   .....................................................................    229 bp
pen-C1R4   ...................................................................T.    229 bp
```

pen-C1R1	pen-C1R2	pen-C1R3	pen-C1R4	
***	99.1	98.3	97.8	pen-C1R1
	***	98.3	97.8	pen-C1R2
		***	96.9	pen-C1R3
			***	pen-C1R4

Similarity: 96.9-99.1

S. peruvianum, per

```
Majority   CCTTTTTGTTGAAATTTGATCTCGTAGTTGAAAAAAA-TATACTCATGTATTTATTTTTTGCGTTAAATACGTTCGGATT
                    10        20        30        40        50        60        70        80
           ---------+---------+---------+---------+---------+---------+---------+---------+
per-C1R1   .....................................A..........................................
per-C1R2   .....................................-..........................................
per-C1R3   ....................................G-..........................................
per-C3R3   .....................................-.....................-....................

Majority   GAGAGTTCATTAGGATATGGGATGGTGGCGTCGGGGATGGGCGTGACGGGCGTCGTCGTGCGTCGGTGCGTGGAGGGTTT
                    90       100       110       120       130       140       150       160
           ---------+---------+---------+---------+---------+---------+---------+---------+
per-C1R1   .............................A..................................................
per-C1R2   ................................................................................
per-C1R3   ................................................................................
per-C3R3   ................................................................................

Majority   TGAAGCGGGGGGCGGGCTAGGGCGTTGGGCGGAAGGTTGTGTTTAATAGATTTTAGAGTGCAATGAATGAC
                   170       180       190       200       210       220       230
           ---------+---------+---------+---------+---------+---------+---------+-
per-C1R1   .......................................................................    231 bp
per-C1R2   .......................................................................    230 bp
per-C1R3   ......................................................................A    230 bp
per-C3R3   .......................................................................    229 bp
```

per-C1R1	per-C1R2	per-C1R3	per-C3R3	
***	99.1	98.3	98.7	per-C1R1
	***	99.1	99.6	per-C1R2
		***	98.7	per-C1R3
			***	per-C3R3

Similarity: 98.3-99.6

S. phlomoides, phl

```
Majority   CTTTTTTXGCCGAAATTTGGCCGTGTATTCGGCGAGTGATTATTTATTTCGGCGGTCGGGTCGTTTGCTTGGGCAGAGAC
                    10        20        30        40        50        60        70        80
           ---------+---------+---------+---------+---------+---------+---------+---------+
phl-C1R1   .......G.............A..........................................................
phl-C1R3   .......T........................................................................
phl-C1R2   .......T........................................................................
phl-C2R1   .......G........................................................................

Majority   GTCGTTAGGAAGGGCGTCGCCGGCGCGTGGAGGCTAGGGCGXXXXXXXXXXXXXXXXXXXXTGGGGGG--AGGAATGAGG
                    90       100       110       120       130       140       150       160
           ---------+---------+---------+---------+---------+---------+---------+---------+
phl-C1R1   ...................T....................--------------------.......--..........
phl-C1R3   ........................................--------------------.......--..........
phl-C1R2   ........................................GTGGTGCGCAGGCTAGGGCG.......--..........
phl-C2R1   ........................................GTGGTGCGCAGGCTAGGGCG.......GG..........

Majority   TTTAATAGAATTAAGAGTGCTGGAAATGAT
                   170       180       190
           ---------+---------+---------+
phl-C1R1   ..............................    168 bp
phl-C1R3   ..............................    168 bp
phl-C1R2   ..............................    188 bp
phl-C2R1   ..............................    190 bp
```

phl-C1R1	phl-C1R3	phl-C1R2	phl-C2R1	
***	98.4	87.9	87.4	phl-C1R1
	***	89.5	87.9	phl-C1R3
		***	98.4	phl-C1R2
			***	phl-C2R1

Similarity: 87.4-98.4

S. phureja-1, phu1

DIRECT SEQUENCING - 212 bp

S. phureja-2, phu2

```
Majority   CCTTTTTGTCGAAATTCGGTCGTGTAATAGAAAAAATATTATTATTTATTTTTTGCAGAAACGACGTCGTTAGGACAGGT
                    10        20        30        40        50        60        70        80
           ---------+---------+---------+---------+---------+---------+---------+---------+
phu2-C1R1  ................................................................................
phu2-C1R2  ..........................................C.....................................
phu2-C1R3  ............C........................G.................T........................
phu2-C2R1  .........A......................................................................
phu2-C2R6  ................................................................................
phu2-C2R8  ..........A.........................C...........................................

Majority   GATGGGGGCGTTGAGGATGGGCGTGACGGGCGGCGTCATGCGTCGGTGCGCGTGGAGGCTAGGTCGGTGGGGGGCAGGCT
                    90        100       110       120       130       140       150       160
           ---------+---------+---------+---------+---------+---------+---------+---------+
phu2-C1R1  ................................................................................
phu2-C1R2  .......................................................................A........
phu2-C1R3  .........................................T..............T.......................
phu2-C2R1  ...................................G............................................
phu2-C2R6  .......................................A........................................
phu2-C2R8  ....................................................................T...........

Majority   AGGGCGTTGGGAGGAAGGAGGTGTTTAATAGAATTTAGAGTGCTATGAATGAT
                    170       180       190       200       210
           ---------+---------+---------+---------+---------+---
phu2-C1R1  .....................................................     213 bp
phu2-C1R2  .....................................................     213 bp
phu2-C1R3  .....................................................     213 bp
phu2-C2R1  .....................................................     213 bp
phu2-C2R6  .....................................................     213 bp
phu2-C2R8  .....................................................     213 bp
```

phu2-C1R1	phu2-C1R2	phu2-C1R3	phu2-C2R1	phu2-C2R6	phu2-C2R8	
***	99.1	97.7	99.1	99.5	98.6	phu2-C1R1
	***	96.7	98.1	98.6	97.7	phu2-C1R2
		***	96.7	97.2	96.2	phu2-C1R3
			***	98.6	97.7	phu2-C2R1
				***	98.1	phu2-C2R6
					***	phu2-C2R8

Similarity: 96.2-99.5

S. pimpinellifolium, pim

```
Majority  CCTTTTTGTTGAAATTTGATCTCGTAATTGAAAAAAAATATACTCATTTATTTTTTTTGCGGAAAATACGTTCGGATTGA
                  10        20        30        40        50        60        70        80
          ---------+---------+---------+---------+---------+---------+---------+---------+
pim-C1R1  ................................................................................
pim-C1R2  ................................................................................
pim-C2R1  A...............................................................................
pim-C3R1  .....................................-..........................................

Majority  GGCGTCATTAGGATATGGGATGGTGGCGTCGGGGATGGGCGTGACGGGCGTCGTCGTGCGTCGGTGCGTGGAGGGTTTTA
                  90        100       110       120       130       140       150       160
          ---------+---------+---------+---------+---------+---------+---------+---------+
pim-C1R1  ............................................................G...................
pim-C1R2  ................................................................................
pim-C2R1  .............................................................A..................
pim-C3R1  ................................................................................

Majority  AAGCGGGGGGCGGGCTAGGGCGTTGGGAGGAAGGTTGTGTTTAATAGATTTTAGAGTGCAATGAATGAC
                  170       180       190       200       210       220
          ---------+---------+---------+---------+---------+---------+---------
pim-C1R1  ....................................T................................    229 bp
pim-C1R2  .....................................................................    229 bp
pim-C2R1  .....................................................................    229 bp
pim-C3R1  .....................T...............................................    228 bp
```

pim-C1R1	pim-C1R2	pim-C2R1	pim-C3R1	
***	99.1	98.3	98.3	pim-C1R1
	***	99.1	99.1	pim-C1R2
		***	98.3	pim-C2R1
			***	pim-C3R1

Similarity: 98.3-99.1

S. pinnatisectum-1, pnt1

```
Majority CCTTTTTGTCGAAATTCGGTTCGTGTAXXTGAAAAAAATATXATTAXTTATTTTTTTGCAGAACGACATTCGATTGACGG
                10        20        30        40        50        60        70        80
         ---------+---------+---------+---------+---------+---------+---------+---------+
pSph6    ...........................AT............T....T.................................
pSph10   ...........................GA............T.A..A.................................
pSph32   ...........................GA............A..ATA.................................
pSph48   ...........................AT........TAT.A....T.................................

Majority GTGATGGGXXTTGXXGATGGCGTGACGGGCGGCGTCATGCGTCGGTGCGTGGAGGCTAGGTCGGTGGGGGGCAGGCCAGG
                90       100       110       120       130       140       150       160
         ---------+---------+---------+---------+---------+---------+---------+---------+
pSph6    ........CG...GA.................................................................
pSph10   ........CG...GA........C............G...........................................
pSph32   ........GC...AG........................................A........................
pSph48   ........GC...AG.................................................................

Majority GCGTTGGGAGGAAGGAGGTGTTTAATAGGATTTAGAGTGCTATGAXTGAT
               170       180       190       200       210
         ---------+---------+---------+---------+---------+
pSph6    .............................................A....     210 bp
pSph10   ............................................GG....     210 bp
pSph32   ..................A..........................G....     210 bp
pSph48   .............................................A....     210 bp
```

pSph6	pSph10	pSph32	pSph48	
***	96.2	93.8	96.2	pSph6
	***	93.8	92.4	pSph10
		***	94.8	pSph32
			***	pSph48

Similarity: 92.4-96.2

S. pinnatisectum-2, pnt2

```
Majority    CCTTTTTGTCGAAATTCGGTCGTGTAATTGAAAAAATATATTATTATTTTTTTGCAGAAACGACATTCGGATTGACGGGT
                     10        20        30        40        50        60        70        80
            ---------+---------+---------+---------+---------+---------+---------+---------+
pnt2-C3R1   ..........A.......C.............................................................
pnt2-C3R2   .A...................................................AT..........C..............
pnt2-C3R3   ....................................................................A...........
pnt2-C3R4   ................................................................................
pnt2-C3R5   ................................................................................
pnt2-C3R6   .....................................................A..........................
pnt2-C3R7   ................................................................................
pnt2-C3R8   ............G.........G..G......................................................
pnt2-C3R9   ..................A...................................T.........................
pnt2-C3R10  .A..............................................................................
pnt2-C3R11  ..........C.....................................................................
pnt2-C3R12  .A...................................................A..........................
pnt2-C3R13  T...........C......................................G.................A..........
pnt2-C3R14  .................................C..............................................
pnt2-C3R15  ................................................................................
pnt2-C3R16  ................................................................................
pnt2-C3R17  .A..............................................................................
pnt2-C3R18  ................................................................................

Majority    GATGGGGG-CGTTGAGGATGGGCGTGACGGGCGGCGTCATGCGTCGGTGCGTGGAGGCTAGGTCGGTGGGGGGCAGGCCA
                     90       100       110       120       130       140       150       160
            ---------+---------+---------+---------+---------+---------+---------+---------+
pnt2-C3R1   ........-T................................A.....................................
pnt2-C3R2   ..G.....-............................................................T..........
pnt2-C3R3   A.......G.........................................................G.............
pnt2-C3R4   ........G..............A........................................................
pnt2-C3R5   ........G................T........G.CG..CA.CGA.A...AC.C.CT.TCCCT.CAC.AC...C....G
pnt2-C3R6   ........-..................................................................A....
pnt2-C3R7   ........-.......................................................................
pnt2-C3R8   ........G..........................T............................................
pnt2-C3R9   ........-.......................................................................
pnt2-C3R10  ........-...G...................................................................
pnt2-C3R11  ........-.......................................................................
pnt2-C3R12  ........-.......................................................................
pnt2-C3R13  ........G..G................................................................T...
pnt2-C3R14  ........G..................................................G....................
pnt2-C3R15  ........-..............................C........................................
pnt2-C3R16  ........G.......................................................................
pnt2-C3R17  ........-.......................................................................
pnt2-C3R18  ........G.........................................A.............................

Majority    GGGCGTTGGGAGGAAGGAGGXGTTTAATAGGATTTAGAGTGCTATGAGTGAT
                     170       180       190       200       210
            ---------+---------+---------+---------+---------+--
pnt2-C3R1   ....A.............CAT..........................A....    211 bp
pnt2-C3R2   ....................A.................A........A....    211 bp
pnt2-C3R3   ....................T..........................A....    212 bp
pnt2-C3R4   ....................T..........................A....    212 bp
pnt2-C3R5   ....................T..........................A....    212 bp
pnt2-C3R6   ....................A.G.............................    211 bp
pnt2-C3R7   .......T............T..........................A...A    211 bp
pnt2-C3R8   ....................TC.........................A....    212 bp
pnt2-C3R9   ...................AA...............................    211 bp
pnt2-C3R10  ....................A...............................    211 bp
pnt2-C3R11  ...T................A...............................    211 bp
pnt2-C3R12  ....................T.............A.A...............    211 bp
pnt2-C3R13  ....................A..........................A....    212 bp
pnt2-C3R14  ....................T...............................    212 bp
pnt2-C3R15  ....................A...............................    211 bp
pnt2-C3R16  ....................T...............................    212 bp
pnt2-C3R17  ....................A...............................    211 bp
pnt2-C3R18  ....................A...............................    212 bp
```

pnt2-C3R1	pnt2-C3R2	pnt2-C3R3	pnt2-C3R4	pnt2-C3R5	pnt2-C3R6	pnt2-C3R7	pnt2-C3R8	pnt2-C3R9	pnt2-C3R10	pnt2-C3R11	pnt2-C3R12	pnt2-C3R13	pnt2-C3R14	pnt2-C3R15	pnt2-C3R16	pnt2-C3R17	pnt2-C3R18	
***	92.9	94.8	95.8	83.5	94.3	95.8	93.9	95.8	94.8	95.3	94.3	92.9	94.8	95.3	95.8	95.3	94.8	pnt2-C3R1
	***	94.3	95.3	83.5	95.8	95.3	93.4	95.8	96.2	95.3	95.8	93.4	94.3	95.8	95.3	96.7	95.3	pnt2-C3R2
		***	98.1	86.3	95.8	97.2	96.2	95.8	96.2	96.2	95.8	95.3	97.2	96.7	98.1	96.7	97.2	pnt2-C3R3
			***	86.8	96.7	98.1	97.2	96.7	97.2	97.2	96.7	96.2	98.1	97.6	99.1	97.6	98.1	pnt2-C3R4
				***	84.4	85.8	84.9	84.4	84.9	84.9	84.4	84.0	86.3	85.4	86.8	85.4	85.8	pnt2-C3R5
					***	96.7	94.8	97.2	97.6	97.6	97.2	94.8	96.7	98.1	97.6	98.1	97.6	pnt2-C3R6
						***	96.2	96.7	97.2	97.2	96.7	95.3	97.2	97.6	98.1	97.6	97.2	pnt2-C3R7
							***	94.8	95.3	95.3	94.8	94.8	96.2	95.8	97.2	95.8	96.2	pnt2-C3R8
								***	97.6	97.6	96.2	94.8	96.7	98.1	97.6	98.1	97.6	pnt2-C3R9
									***	98.1	97.6	95.3	97.2	98.6	98.1	99.5	98.1	pnt2-C3R10
										***	96.7	95.3	97.2	98.6	98.1	98.6	98.1	pnt2-C3R11
											***	93.9	96.7	97.2	97.6	98.1	96.7	pnt2-C3R12
												***	95.3	95.8	96.2	95.8	96.2	pnt2-C3R13
													***	97.6	99.1	97.6	98.1	pnt2-C3R14
														***	98.6	99.1	98.6	pnt2-C3R15
															***	98.6	99.1	pnt2-C3R16
																***	98.6	pnt2-C3R17
																	***	pnt2-C3R18

Similarity: 83.5-99.5

S. polyadenium-1, pld1

```
Majority   CTTTTTTGTCAAAATTCGATATTATAATTTTTTTGCAGAGACGACATTGGGATTGAGACGTCGTCAGGAAAGGTGATGGG
                    10        20        30        40        50        60        70        80
           ---------+---------+---------+---------+---------+---------+---------+---------+
pSpld5S11  ................................................................................
pSpld5S13  ..............................................G.................................
pSpld5S12  ................A...T......A.AC...........................G..........C..........

Majority   -------------GGCGTAACGGGCCGCGTGACGCGTCGGTGCGTGGAGGCTAGGTCGGTGGGGGGTAGGCTAGGGCGTT
                    90        100       110       120       130       140       150       160
           ---------+---------+---------+---------+---------+---------+---------+---------+
pSpld5S11  -------------...............................A.A........................A........
pSpld5S13  -------------......................A............C...............................
pSpld5S12  CGTGTTGAGGACG.....G......G............C.........................................

Majority   GGGAGGAAGGAGGTGTTTAATAGAATTTAGAGTGCTATGAATGGAT
                    170       180       190       200
           ---------+---------+---------+---------+------
pSpld5S11  ..............................................     193 bp
pSpld5S13  .A............................................     193 bp
pSpld5S12  ..............................................     206 bp
```

pSpld5S11	pSpld5S13	pSpld5S12	
***	96.6	87.4	pSpld5S11
	***	86.9	pSpld5S13
		***	pSpld5S12

Similarity: 86.9-96.6

S. polyadenium-2, pld2

```
Majority   CTTTTTTGTCAAAATTCGXTATTATAATTATTTTGCAGAGACGACATTGGGATTGAGACGTCGTCAGGAXAGGTGATGGG
                    10        20        30        40        50        60        70        80
           ---------+---------+---------+---------+---------+---------+---------+---------+
pld2-C1R1  ..........G.......G..................................................C..........
pld2-C1R2  ..................G......................T...........................C..........
pld2-C1R3  ..................A..........T.......................................A..........
pld2-C1R4  ..........G.......G......................T...........................A.......A..
pld2-C1R5  ...........C....A.A.-......A..C...................A.......G..........C..........
pld2-C1R6  A.................A..........T............................T..........A..........

Majority   CGTXTTGAGGACGGGCGTGACGGGCGGCGTGACGCGTCGGTGCGTGGAGGCTAGGTCGGTGGGGGGTAGGCTAGGG----
                    90        100       110       120       130       140       150       160
           ---------+---------+---------+---------+---------+---------+---------+---------+
pld2-C1R1  ...G........................................................................----
pld2-C1R2  ...C.........................................................A..............----
pld2-C1R3  -------------.....A......C.............................................A....TGTT
pld2-C1R4  ...C..........CT...................A............C.........T..A..............----
pld2-C1R5  ...G........................................................................----
pld2-C1R6  -------------.....A......C.............................................A....TGTT

Majority---CGTTGGGAGGAAGGAGGTGTTTAATAGAATTTAGAGTGCTATGAATGGAT
                    170       180       190       200       210
           ---------+---------+---------+---------+---------+---
pld2-C1R1  ---..................................................   206 bp
pld2-C1R2  ---..................................................   206 bp
pld2-C1R3  GGG..............................................T-..   199 bp
pld2-C1R4  ---..................................................   206 bp
pld2-C1R5  ---A.................................................   205 bp
pld2-C1R6  GGG..............................................T-..   199 bp
```

pld2-C1R1	pld2-C1R2	pld2-C1R3	pld2-C1R4	pld2-C1R5	pld2-C1R6	
***	98.1	86.4	95.3	95.3	85.4	pld2-C1R1
	***	85.9	96.2	94.4	85.0	pld2-C1R2
		***	83.1	83.6	99.1	pld2-C1R3
			***	90.6	82.2	pld2-C1R4
				***	83.1	pld2-C1R5
					***	pld2-C1R6

Similarity: 82.2-99.1

S. pseudocapsicum, pse

```
Majority      CCTTTTTGCCAGCAGAAACGGGGCGAGGAGGGGCGTCTGACGGCCGGAGCATTTTTTTTTCAGCAGAAACGGGGCGAGGA
                       10        20        30        40        50        60        70        80
              ---------+---------+---------+---------+---------+---------+---------+---------+
pSpse-5S5     ..C.............................................................................
pSpse-5S7(1)  T...............................................................................
pSpse-5S7(2)  ...........................................................-..........T.........

Majority      GGGGCGTGTCGGGGGTCACGACGCGCCGGGGCGGGGGGTGGAGGCTGGGGGAGGGGAGTGAGGCTTAATAGAATTTCGAG
                       90       100       110       120       130       140       150       160
              ---------+---------+---------+---------+---------+---------+---------+---------+
pSpse-5S5     ................................................................................
pSpse-5S7(1)  ..............A..............................G.....C............................
pSpse-5S7(2)  .........................................................TA.....................

Majority      CGCGCTGGATGAT
                       170
              ---------+---
pSpse-5S5     .............     173 bp
pSpse-5S7(1)  .............     173 bp
pSpse-5S7(2)  ......T......     172 bp
```

pSpse-5S5	pSpse-5S7(1)	pSpse-5S7(2)	
***	97.1	97.1	pSpse-5S5
	***	95.3	pSpse-5S7(1)
		***	pSpse-5S7(2)

Similarity: 95.3-97.1

S. pseudolulo, psl

```
Majority        CCTTTTTTTTGTCGAAACTCGGCATAATTTCGCCATCACTATTTTTTTTCC-CGGAAACGAXATTTGTTTGGGTCGAGGC
                        10        20        30        40        50        60        70        80
                ---------+---------+---------+---------+---------+---------+---------+---------+
pSpsl-2         ...................................................-..A...T..A..................
pSpsl-3         ...................................................-.........C..................
pSpsl-5S1       T...............................T..............C.TTG.........T..................

Majority        GTCGCTTGGGCAATTGAGGAGGGG-CGCCGCCGTGCGACGGTGCGTGGAGGCTAGGGCGGTGGTGCGCGGGCTAGGGCGT
                        90       100       110       120       130       140       150       160
                ---------+---------+---------+---------+---------+---------+---------+---------+
pSpsl-2         ........................-.......................................................
pSpsl-3         ........................-.......................................................
pSpsl-5S1       ......A.....G...........G............G........................A...............A.

Majority        AGGGAGGAATGAGGTTTAATAGAATTTAGAGTGGTAXGATTAAATAGAATTTAGAGTGCTACGAATGAC
                       170       180       190       200       210       220
                ---------+---------+---------+---------+---------+---------+---------
pSpsl-2         ....................................G................................   227 bp
pSpsl-3         ....................................C................................   227 bp
pSpsl-5S1       .....................C...........C..-------------------..------.....A   204 bp
```

pSpsl-2	pSpsl-3	pSpsl-5S1	
***	98.3	81.2	pSpsl-2
	***	82.1	pSpsl-3
		***	pSpsl-5S1

Similarity: 81.2-98.3

S. quitoense, qui

```
Majority    CCTTTTTTAT-TCGAAACTGGGCATAATTTTGTCATCATTATTTTTTTTGGAGGAAACGACATTTGTTTGGGCCGAGGCG
                     10        20        30        40        50        60        70        80
            ---------+---------+---------+---------+---------+---------+---------+---------+
pSqui-5S1   ........T.G........C..........C.C.....C..........TCC.A..........A.......T.......
pSqui-5S2   ..........-.....................................................................
pSqui-5S4   ..........-.....................................................................
pSqui-5S5   ..........-.....................................................................

Majority    TCGCTAGGAATGTTGATGAGGG-CGCCGC-----------GCGTGGAGGCGATGGTGGTGGTGCGCGGGCTAGGGCGTCG
                     90        100       110       120       130       140       150       160
            ---------+---------+---------+---------+---------+---------+---------+---------+
pSqui-5S1   .....T...CAA....G.....G......CGTGCGACGGT..........T.G..C.......A..............A.
pSqui-5S2   ......................-......-----------.-..............A.......................
pSqui-5S4   ......................-......-----------.........G..............................
pSqui-5S5   ......................-......-----------........................................

Majority    GGAGGAATGAGGTTTAATGGAATTTAGAGTGCTGGGA-------------------------ATGAC
                     170       180       190       200       210       220
            ---------+---------+---------+---------+---------+---------+-------
pSqui-5S1   ..................A............G.A...TTAAATAGAATTTAGAGTGCTACGA.....     227 bp
pSqui-5S2   ............................A........-------------------------.....     188 bp
pSqui-5S4   .....................................-------------------------.....     189 bp
pSqui-5S5   .....................................-------------------------.....     189 bp
```

pSqui-5S1	pSqui-5S2	pSqui-5S4	pSqui-5S5	
***	71.4	72.2	72.7	pSqui-5S1
	***	98.2	98.7	pSqui-5S2
		***	99.6	pSqui-5S4
			***	pSqui-5S5

Similarity: 71.4-99.6

S. raphanifolium-1, rap1

DIRECT SEQUENCING - **172 bp**

S. raphanifolium-2, rap2

```
Majority   CCTTTTTGTCGAAATTXGGXCGTGXAAXXXTXAAAXAXTATATTXXXXTTXTGXAGAAACGACXXXXXXXXXXXXXXGTC
                   10        20        30        40        50        60        70        80
           ---------+---------+---------+---------+---------+---------+---------+---------+
rap2-L-DS  ................C..T....T..TAT.T...T.T......ATTT..C..C.........ATTCGGATTGAGAC...
rap2-S-DS  ................G..C....C..A--.G...A.A......----..T..G.........--------------...

Majority   GTTXXGXXXXXXGATGGGGGCGTXGAGGATGGGCGTXXXXXXXXXXXXXXXXXXXCGGTGCGTGGAGGCTXGGTCGGXGG
                   90       100       110       120       130       140       150       160
           ---------+---------+---------+---------+---------+---------+---------+---------+
rap2-L-DS  ...AG.ACAGGT...........C............GACGGGCGGCGTCATGCGT...............A......T..
rap2-S-DS  ...GA.------...........T............-------------------...............G......C..

Majority   GGGGCAGGCTAGGGCGTTGGGAGGAAGGAGGTGTTTAATAGAATTTAGAGTGCTATGAATGAT
                  170       180       190       200       210       220
           ---------+---------+---------+---------+---------+---------+---
rap2-L-DS  ...............................................................    223 bp
rap2-S-DS  ...............................................................    178 bp
```

rap2-L-DS	rap2-S-DS	
***	73.5	rap2-L-DS
	***	rap2-S-DS

Similarity: 73.5

Note: DIRECT SEQUENCING of long (L) and short (S) bands isolated from gel

S. raphanifolium-3, rap3

```
Majority    CCTTTTTGTCGAAATTGGGTCGTGCAAATGAAAAAATATATTTTTTGGAGAAACGACGTCGTTGAGGATGGGGGCGTTGA
                     10        20        30        40        50        60        70        80
            ---------+---------+---------+---------+---------+---------+---------+---------+
rap3-C1R1   ......G.......................................................................T.
rap3-C1R2   ...................C............................................................
rap3-C1R3   ...........................................A....................................
rap3-C2R1   ...........................................A..............---------------.......
rap3-C2R2   ................................................................................
rap3-C3R1   ......G.........................................................................
rap3-C3R2   ..................................................................A.............
rap3-C4R1   ...................C............................................................

Majority    GGATGGGCGTCGGTGCGTGGAGGCTGGGTCGGCGGGGGGCAGGCTAGGGCGTTGGGAGGGAGGAGGTGTTTAATAGAATT
                     90       100       110       120       130       140       150       160
            ---------+---------+---------+---------+---------+---------+---------+---------+
rap3-C1R1   ................................................................................
rap3-C1R2   .................................T.........................A....................
rap3-C1R3   ...........................................................A....................
rap3-C2R1   ............................................................................T...
rap3-C2R2   ................................................................................
rap3-C3R1   ..................................................T......................C......
rap3-C3R2   ................................................................................
rap3-C4R1   ...........................................................A....................

Majority    TAGAGTGCTATGAATGAT
                   170
            ---------+--------
rap3-C1R1   ....A.............     178 bp
rap3-C1R2   ..................     178 bp
rap3-C1R3   ..................     178 bp
rap3-C2R1   ..................     163 bp
rap3-C2R2   ..................     178 bp
rap3-C3R1   ..................     178 bp
rap3-C3R2   .......G..........     178 bp
rap3-C4R1   ...............A..     178 bp
```

rap3-C1R1	rap3-C1R2	rap3-C1R3	rap3-C2R1	rap3-C2R2	rap3-C3R1	rap3-C3R2	rap3-C4R1	
***	96.6	97.2	88.8	98.3	97.8	97.2	96.6	rap3-C1R1
	***	98.3	88.8	98.3	96.6	97.2	98.9	rap3-C1R2
		***	90.4	98.9	97.2	97.8	98.3	rap3-C1R3
			***	90.4	88.8	89.9	88.8	rap3-C2R1
				***	98.3	98.9	98.3	rap3-C2R2
					***	97.2	96.6	rap3-C3R1
						***	97.2	rap3-C3R2
							***	rap3-C4R1

Similarity: 88.8-98.9

S. scabrum, sca

```
Majority  CCTTTTTGTCGAAATTCGGCATAATTXCGTCTATATGATAATATATATTATTATTATTTTTGCGGAAACGGCATTCGTGC
                   10        20        30        40        50        60        70        80
          ---------+---------+---------+---------+---------+---------+---------+---------+
sca-C1R1  .............G........G...T.......T....................-.......A......A.........
sca-C2R1  ..........................T............................T..A.....................
sca-C5R1  .................A........C.........................................T...........
sca-C5R2  .......................T..C...................T.................A...............

Majority  CTAGACGTCGCTAGGACGGGTGACGGAGGCGCTAAGGACGGGCGTGACAGGCATGCCGTCGTTGCGTGGAGGATAGGGCG
                   90       100       110       120       130       140       150       160
          ---------+---------+---------+---------+---------+---------+---------+---------+
sca-C1R1  ..................................................A.............................
sca-C2R1  .......................T......................................................T.
sca-C5R1  .................................................T..............................
sca-C5R2  ................................................................................

Majority  GCGXGGGAXATGCTAAGGCGTTTGGAGGAAGGAGGTGTTTAATAGAATTTAGAGCGCAATGAATGAT
                  170       180       190       200       210       220
          ---------+---------+---------+---------+---------+---------+-------
sca-C1R1  ...A....T......................A...................A...............    226 bp
sca-C2R1  ...A....T..............T...........................................    227 bp
sca-C5R1  ...G....C......T............................C......................    227 bp
sca-C5R2  ...G....C.........................................................C    227 bp
```

sca-C1R1	sca-C2R1	sca-C5R1	sca-C5R2	
***	94.3	92.5	93.0	sca-C1R1
	***	94.3	94.7	sca-C2R1
		***	96.0	sca-C5R1
			***	sca-C5R2

Similarity: 92.5-96.0

S. seaforthianum, sea

```
Majority TTTTTTGGTCGAAATTCGACATAATATTTTCGTTTGACAATGTATATAAATATATTTTGCAGAAACGACATTAGAAGCCC
                10        20        30        40        50        60        70        80
         ---------+---------+---------+---------+---------+---------+---------+---------+
pSsea-1  ..............................................A.................................
pSsea-3  ..........A.....................................................................
pSsea-4  ..A............................A.................C.........A...................T

Majority AGAGGTCGTTAGGATATGCGATTGAGATACTGGTTATAGACTAGACAGACGGCACCATXCGTCGATGCATGGAAGATTGG
                90       100       110       120       130       140       150       160
         ---------+---------+---------+---------+---------+---------+---------+---------+
pSsea-1  .........................................................AT.....................
pSsea-3  .............................G....C.......................C.................C.A.
pSsea-4  ...C..........C....................G....G....A.....A......G........T............

Majority GCGGTGAGGGACATGCTAGTGCGTTGTGAGGAAGGAGGTGTTTAATACAATTTAGGGTGCTAGGAATGAT
               170       180       190       200       210       220       230
         ---------+---------+---------+---------+---------+---------+---------+
pSsea-1  ....C..............G..............................C..TC.A.T...........    230 bp
pSsea-3  ...........G..........................................................    230 bp
pSsea-4  .............A................................T.......................    230 bp
```

pSsea-1	pSsea-3	pSsea-4	
***	93.0	89.6	pSsea-1
	***	90.9	pSsea-3
		***	pSsea-4

Similarity: 89.6-93.0

S. sejunctum, sej

```
Majority   CTTTTTTAGCCGAAATTTCGTCGTCTACTCGGCGGATGATTATTTTTTTTGGCGGTTGGGTCGTTTGCTTGGGCAGAGAC
                    10        20        30        40        50        60        70        80
           ---------+---------+---------+---------+---------+---------+---------+---------+
sej-C1R1   ..........................................A.....................................
sej-C2R1   ..........................................................................A......
sej-C6R1   ................................................................................
sej-C11R1  ................................................................................

Majority   GTCGTTAGGACGGTTGXGAAAGGGCGTCGCCGGCGCGTGGATACCCAGGCGGTGGTGTGCAGGCTTGGGCGTGGGTGAGT
                    90       100       110       120       130       140       150       160
           ---------+---------+---------+---------+---------+---------+---------+---------+
sej-C1R1   ............A...C...............................................................
sej-C2R1   ................G..............................A................................
sej-C6R1   ................C...............................................................
sej-C11R1  ................G...............................................................

Majority   XGAGGAATGAGGTTCAATAGAATTAAGAGTGCTAGGAATGCC
                    170       180       190       200
           ---------+---------+---------+---------+--
sej-C1R1   C.........................................     202 bp
sej-C2R1   G.........................................     202 bp
sej-C6R1   C.........................................     202 bp
sej-C11R1  G.........................................     202 bp
```

sej-C1R1	sej-C2R1	sej-C6R1	sej-C11R1	
***	97.0	99.0	98.0	sej-C1R1
	***	98.0	99.0	sej-C2R1
		***	99.0	sej-C6R1
			***	sej-C11R1

Similarity: 97.0-99.0

S. sisymbriifolium, sis

```
Majority CCTTTTTGTCGAAATTCGGCATAATATCGCCGTCTGTTTGATGAGATAXTTTTTTTTTCCGCGGGAACGACATTTTGGCC
                  10        20        30        40        50        60        70        80
         ---------+---------+---------+---------+---------+---------+---------+---------+
sis-C1R1 ................................................C...............................
sis-C2R1 ................................................T...............................

Majority GAGACGTCGACAGGACGACGGTTGAGGAAGGGCGTCACCGCGCGCCGGTGCGCGGAGGCTGGGGCGGTGGTGTGCAGGGC
                  90       100       110       120       130       140       150       160
         ---------+---------+---------+---------+---------+---------+---------+---------+
sis-C1R1 ................................................................................
sis-C2R1 ................................................................................

Majority AGGGCGCTGGGACGAATGAGGTTTAATAGAATTTAGAGCGCTAGGAATGAT
                 170       180       190       200       210
         ---------+---------+---------+---------+---------+-
sis-C1R1 ...................................................    211 bp
sis-C2R1 ...................................................    211 bp
```

sis-C1R1	sis-C2R1	
***	99.5	sis-C1R1
	***	sis-C2R1

Similarity: 99.5

S. sitiens, sit

```
Majority  CTTTTTTGTCTAAATTCGGTCGCGTGATTGAAAAAAATATACTTATTTATTTATTTTTGCAGAAAATACGTTCGGATTGA
                   10        20        30        40        50        60        70        80
          ---------+---------+---------+---------+---------+---------+---------+---------+
sit-C1R1  ................................................................................
sit-C1R2  ................................................................................
sit-C1R3  ..........G...C.................................................................
sit-C2R1  .......................................................................C........
sit-C3R1  ..........G.....................................................................

Majority  GACGTCATTAGGATATGGGACGGGGGCGTTGAGGATGGGCGTGACGTGCGGCGTCATGCGTCGGTGCGTGGAGGGTTTTA
                   90       100       110       120       130       140       150       160
          ---------+---------+---------+---------+---------+---------+---------+---------+
sit-C1R1  ................................................................................
sit-C1R2  ...........................................................................G....
sit-C1R3  ...............................................T................................
sit-C2R1  ................................................................................
sit-C3R1  ...............................................T................................

Majority  AAGTGGTGGACGGGCTAGGGCGTTGGGAGGAAGGATGTGCTTAATAGAATTTAGAGTGCAACGAATGAT
                  170       180       190       200       210       220
          ---------+---------+---------+---------+---------+---------+---------
sit-C1R1  .............................T.......................................    229 bp
sit-C1R2  .....................................................................    229 bp
sit-C1R3  .............................................................T.......    229 bp
sit-C2R1  .............................T.......................................    229 bp
sit-C3R1  .............................................................T.......
```

sit-C1R1	sit-C1R2	sit-C1R3	sit-C2R1	sit-C3R1	
***	99.1	97.8	99.6	98.3	sit-C1R1
	***	97.8	98.7	98.3	sit-C1R2
		***	97.4	99.6	sit-C1R3
			***	97.8	sit-C2R1
				***	sit-C3R1

Similarity: 97.4-99.6

S. sogarandinum-1, sgr1

```
Majority   CCTTTTTGTCGAAATTCGGTCGTGTAATTGAAGAAATATATTATTATTTTTTTGCAGAAACGACATTCGGATAGAGACGT
                    10        20        30        40        50        60        70        80
           ---------+---------+---------+---------+---------+---------+---------+---------+
sgr1-C1R1  ..................................................C.............................
sgr1-C2R2  -.................................................C.............................
sgr1-C6R1  .............................................T..G.C.............................
sgr1-C1R2  ................................A.G.............................................
sgr1-C1R4  ................................A.G.............................................
sgr1-C4R1  ................................A.G...............................G.............
sgr1-C1R3  ..............................G.................................................
sgr1-C2R1  .......---------------------------------------------............................
sgr1-C3R1  -...............................................................................
sgr1-C4R2  ................................................................................

Majority   CGTTAGGACAGGTGATGTGGGAGTTGAGGATGGGCGTGACGGGCGGCGTCACGCGTCGGTGCGTGGAGGCTAGGTCGGCG
                    90       100       110       120       130       140       150       160
           ---------+---------+---------+---------+---------+---------+---------+---------+
sgr1-C1R1  ....G...................G..............T........................................
sgr1-C2R2  ....G...........................................................................
sgr1-C6R1  ...............................................................C................
sgr1-C1R2  ...C.............C--------------................................................
sgr1-C1R4  ...C.............C--------------...............................................A
sgr1-C4R1  ...C.......C....C--------------................................................
sgr1-C1R3  ......................................................................G--------.
sgr1-C2R1  ......................................................................G--------.
sgr1-C3R1  ......................................................................G--------.
sgr1-C4R2  ...G..................................................................G--------.

Majority   GGGGGCAGGCTAGGGCGTTGGGAGGAAGGAGGTGTTTAATAGAATTTAGAGTGCTATGAATGAT
                   170       180       190       200       210       220
           ---------+---------+---------+---------+---------+---------+----
sgr1-C1R1  ............................................................G...    224 bp
sgr1-C2R2  ................................................................    224 bp
sgr1-C6R1  ................................................................    224 bp
sgr1-C1R2  ...................................C............................    210 bp
sgr1-C1R4  .A........CGACC.C.GA.A..T...C.C...CCC..C.CG.CGC.CT.C.G.TC.......    210 bp
sgr1-C4R1  ...................................C............................    210 bp
sgr1-C1R3  ....-..............................C............................    215 bp
sgr1-C2R1  ....-......G....................................................    170 bp
sgr1-C3R1  ................................................................    216 bp
sgr1-C4R2  ....-...............................C...........................    215 bp
```

sgr1-C1R1	sgr1-C2R2	sgr1-C6R1	sgr1-C1R2	sgr1-C1R4	sgr1-C4R1	sgr1-C1R3	sgr1-C2R1	sgr1-C3R1	sgr1-C4R2	
***	98.2	96.9	89.7	77.2	88.8	92.4	71.9	93.3	92.4	sgr1-C1R1
	***	97.8	90.2	77.7	89.3	93.3	73.7	95.1	93.3	sgr1-C2R2
		***	89.7	77.2	88.8	92.9	72.8	93.8	92.9	sgr1-C6R1
			***	87.5	99.1	87.1	66.5	87.1	86.6	sgr1-C1R2
				***	86.6	74.6	54.9	74.6	75.0	sgr1-C1R4
					***	86.2	65.6	86.2	85.7	sgr1-C4R1
						***	78.1	98.2	98.2	sgr1-C1R3
							***	78.6	77.7	sgr1-C2R1
								***	98.2	sgr1-C3R1
									***	sgr1-C4R2

Similarity: 54.9-99.1

S. sogarandinum2, sgr2

```
Majority     ACTTTTTGTCGAAATTCGGTCGTGTAATTGAAAAAATATATTATTATTATTTTGCAGAAACGAAATTCGGATTGAGACGT
                      10        20        30        40        50        60        70        80
             ---------+---------+---------+---------+---------+---------+---------+---------+
sgr2-C1R1    .............................................................................T..
sgr2-C1R2    ................................................................................
sgr2-C6R1    ................................................................................
sgr2-C6R3    .................................................A..............................
sgr2-C6R2    .......................A.......................................C................
sgr2-C1R3    ..................T............................................C................

Majority     CGTTAGGACAGGTGA------------------------------GCGTCACGCGTCGGTGCGTGGAGGCTAGGTCGGTG
                      90       100       110       120       130       140       150       160
             ---------+---------+---------+---------+---------+---------+---------+---------+
sgr2-C1R1    ...............------------------------------...................................
sgr2-C1R2    ...............------------------------------...................A...............
sgr2-C6R1    ...............------------------------------...................................
sgr2-C6R3    ...............------------------------------...................A...............
sgr2-C6R2    .......T.......TGTGGGAGTTGAGGATGGGCGTGACGGGCG...................................
sgr2-C1R3    .......T.......TGTGGGAGTTGAGGATGGGCGTGACGGGCG.........A.........................

Majority     GGGGGCAGGCTAGGGCGTTGGGAGGAAGGAGGTGTTTAATAGAATTTAGAGTGCTATGAATGAT
                     170       180       190       200       210       220
             ---------+---------+---------+---------+---------+---------+----
sgr2-C1R1    ................................................................   194 bp
sgr2-C1R2    ................................................................   194 bp
sgr2-C6R1    ................................................................   194 bp
sgr2-C6R3    ................................................................   194 bp
sgr2-C6R2    .................................................G..............   224 bp
sgr2-C1R3    .....A..........................................................   224 bp
```

sgr2-C1R1	sgr2-C1R2	sgr2-C6R1	sgr2-C6R3	sgr2-C6R2	sgr2-C1R3	
***	99.1	99.6	98.7	84.4	83.9	sgr2-C1R1
	***	99.6	99.6	84.4	83.9	sgr2-C1R2
		***	99.1	84.8	84.4	sgr2-C6R1
			***	83.9	83.5	sgr2-C6R3
				***	97.8	sgr2-C6R2
					***	sgr2-C1R3

Similarity: 83.5-99.6

S. sparsipilum-1, spl1

DIRECT SEQUENCING - **216 bp**

S. sparsipilum-2, spl2

```
Majority   CCTTTTTGTCGAAATTTGGXCGTGTAATTGAAAAAATATTATTATTTATTTTTTGCAGGAACGACGTCGTTAGGACAGGT
                    10        20        30        40        50        60        70        80
           ---------+---------+---------+---------+---------+---------+---------+---------+
spl2-C1R1  ..........A..T...T---------..-------.-.............G............................
spl2-C1R3  .G...........T...T---------..-------............................................
spl2-C1R2  ...................C........................................G...................
spl2-C1R4  ...................T........................................................G...
spl2-C1R5  ...................T............................................................

Majority   GATGGGGGCGTTGAGGATGGGCGTGACGGGCGGCGTCAAGCGTCGGTGCGCGTGGAGGCTAGGTCGGTGGGGGGCAGGCT
                    90       100       110       120       130       140       150       160
           ---------+---------+---------+---------+---------+---------+---------+---------+
spl2-C1R1  ...............................A.......T........................................
spl2-C1R3  ................................................................................
spl2-C1R2  ...................................G..T.........................................
spl2-C1R4  ...........G...............................................G....................
spl2-C1R5  ......................................T.........................................

Majority   AGGGCGTTGGGAGGAAGGAGGTGTTTAATAGAATTTAGAGTGCTATGAATGAT
                   170       180       190       200       210
           ---------+---------+---------+---------+---------+---
spl2-C1R1  ...........C.........................................   196 bp
spl2-C1R3  .....................G...............................   197 bp
spl2-C1R2  .....................................................   213 bp
spl2-C1R4  .....................................................   213 bp
spl2-C1R5  .....................................................   213 bp
```

spl2-C1R1	spl2-C1R3	spl2-C1R2	spl2-C1R4	spl2-C1R5	
***	96.2	87.3	87.3	88.3	spl2-C1R1
	***	89.2	89.2	90.1	spl2-C1R3
		***	96.7	98.6	spl2-C1R2
			***	98.1	spl2-C1R4
				***	spl2-C1R5

Similarity: 87.3-98.6

S. spegazzinii-1, spg1

DIRECT SEQUENCING - **205 bp**

S. spegazzinii-2, spg2

```
Majority      CCTTTTTGTCGAAATTCGGTCGTGTAATTGAAGAAATATATTTATTTATTTTTTGAAGAAAAGGCGACGTTAGGACAGGT
                       10        20        30        40        50        60        70        80
              ---------+---------+---------+---------+---------+---------+---------+---------+
spg2-C1R1     ...................................................A........................G...
spg2-C1R2     ................................................................................
spg2-C1R3     ................................................................................
spg2-C1R4     .............C..............................................C...................
spg2-C1R5     ................................................................................
spg2-C1R6     .....................................T..........................................

Majority      GATGGGGGCGTTGAGGATGGGCGTGACGGGCGGCGTCATGCGTCGGTGCGCGTGGAGGCTAGGTCGGTGGGGGGAGGGGG
                       90       100       110       120       130       140       150       160
              ---------+---------+---------+---------+---------+---------+---------+---------+
spg2-C1R1     ................................................................................
spg2-C1R2     ................................................................................
spg2-C1R3     ..........G......................................................AA.............
spg2-C1R4     ................................................................G..G............
spg2-C1R5     ...........A...............................................G....................
spg2-C1R6     ...........A....................................................................

Majority      CAGGCTAGGGCGTTGGGAGGAAGGAGGTGTTTAATAGAATTTAGAGTGCTATGAATGAT
                       170       180       190       200       210
              ---------+---------+---------+---------+---------+---------
spg2-C1R1     ...........................................................   219 bp
spg2-C1R2     ...........................................................   219 bp
spg2-C1R3     .....................................C.....................   219 bp
spg2-C1R4     ........................................C.........C........   219 bp
spg2-C1R5     ...........................................................   219 bp
spg2-C1R6     ..........................................CC...............   219 bp
```

spg2-C1R1	spg2-C1R2	spg2-C1R3	spg2-C1R4	spg2-C1R5	spg2-C1R6	
***	99.1	97.3	96.3	98.2	97.3	spg2-C1R1
	***	98.2	97.3	99.1	98.2	spg2-C1R2
		***	95.4	97.3	96.3	spg2-C1R3
			***	96.3	95.4	spg2-C1R4
				***	98.2	spg2-C1R5
					***	spg2-C1R6

Similarity: 95.4-99.1

.

S. spirale, spi

```
Majority   CTTTTTTCGTCG-----------TCTATTCGGCGAATGATTTTTTTTTTTT---GCGGTTGGGTCGTTTGCTTGGGGAGA
                    10        20        30        40        50        60        70        80
           ---------+---------+---------+---------+---------+---------+---------+---------+
spi-C1R1   ............-----------............................---.................G........
spi-C1R2   ............-----------...........................----..........................
spi-C5R1   ............-----------............................TTT..........T...............
spi-C5R2   ............-----------............................---..........................
spi-C15R1  ............-----------............................T--..........................
spi-C20R1  .........C..AAATTTTGTCA...........................----....C.....................

Majority   GACGTCGTTAGGACGGTTGGGGAAGGGCGTCGCGATAGCAAAAAGGGCTATGGCGGTGGTGTGCATGGCTAGGGCGTGGG
                    90       100       110       120       130       140       150       160
           ---------+---------+---------+---------+---------+---------+---------+---------+
spi-C1R1   ................................................................................
spi-C1R2   .........C......................................................................
spi-C5R1   ...................A..............G.............................................
spi-C5R2   ................................................................................
spi-C15R1  ................................................................................
spi-C20R1  A..............................A..G.............................................

Majority   GGXAGGAATGAGGTTTAATAGAATTAAGAGTGCTAGGAATGAT
                   170       180       190       200
           ---------+---------+---------+---------+---
spi-C1R1   ..-............................T...........     188 bp
spi-C1R2   ..G.............................G..........     188 bp
spi-C5R1   ..-............................T...T.......     191 bp
spi-C5R2   ..G.............................G..........     189 bp
spi-C15R1  ..G.............................A..........     190 bp
spi-C20R1  ..-........................................     198 bp
```

spi-C1R1	spi-C1R2	spi-C5R1	spi-C5R2	spi-C15R1	spi-C20R1	
***	97.0	96.1	98.0	97.5	89.7	spi-C1R1
	***	94.1	99.0	98.0	89.7	spi-C1R2
		***	95.1	95.6	88.7	spi-C5R1
			***	99.0	89.7	spi-C5R2
				***	89.2	spi-C15R1
					***	spi-C20R1

Similarity: 88.7-99.0

S. stenophyllidium, ste

```
Majority  CCTTTTTGTCGAAATTCGGTCGTGTAATTGAAAAAGTATATTATTATTTATTTGCAGAAACGACATTGGGATTGAGACGT
                   10        20        30        40        50        60        70        80
          ---------+---------+---------+---------+---------+---------+---------+---------+
ste-C1R1  ................................................................................
ste-C1R2  ................................................................................
ste-C2R1  ................................................................................

Majority  CGTTAGGACAGGTGATGGGGGGCGTTGAGGAGGGGCGTGACGGGCGGCGTCGTGCGTGGAGGCTAGGTCGGTGGGGGGGG
                   90       100       110       120       130       140       150       160
          ---------+---------+---------+---------+---------+---------+---------+---------+
ste-C1R1  ................................................................................
ste-C1R2  ................................................................................
ste-C2R1  ................................................................................

Majority  --AGGCTAGGGCGTTGGGAGGAAGGTGGTGTTTAATAGAATTTAGAGTGCTATGAATGAC
                  170       180       190       200       210       220
          ---------+---------+---------+---------+---------+---------+
ste-C1R1  --..........................................................    218 bp
ste-C1R2  GG..........................................................    220 bp
ste-C2R1  --.................A........................................    218 bp
```

ste-C1R1	ste-C1R2	ste-C2R1	
***	99.1	99.5	ste-C1R1
	***	98.6	ste-C1R2
		***	ste-C2R1

Similarity: 98.6-99.5

S. stenotomum subsp. goniocalyx, gon

```
Majority CCTTTTTGTCGAAATTCGGTCGTGTAATAGAAAAAATATTATTATTTATTTTTTGTAGAAACGACGTCGTTAGGACAGGT
                  10        20        30        40        50        60        70        80
         ---------+---------+---------+---------+---------+---------+---------+---------+
gon-C1R1 ....................................C.G.........................................
gon-C1R2 ...............................................G......A.........................
gon-C4R1 ................................................................................
gon-C4R2 .........................................A......................................

Majority GATGGGGGCGTTGAGGATGGGCGTGACGGGCGGCGTCATGCGTCGGTGCGCGTGGAGGCTAGGTCGGTGGGGGGCAGGCT
                  90       100       110       120       130       140       150       160
         ---------+---------+---------+---------+---------+---------+---------+---------+
gon-C1R1 ................................................................................
gon-C1R2 ..................T.............................................................
gon-C4R1 .....A..........................................................................
gon-C4R2 .............C........................................C.........................

Majority AGGGCGTTGGGAGGAAGGAGGTGTTTAATAGAATTTAGAGTGCTATGAATGAT
                 170       180       190       200       210
         ---------+---------+---------+---------+---------+---
gon-C1R1 .....................................................     213 bp
gon-C1R2 .......................C.............................     213 bp
gon-C4R1 .....................................................     213 bp
gon-C4R2 .....................................................     213 bp
```

gon-C1R1	gon-C1R2	gon-C4R1	gon-C4R2	
***	97.2	98.6	97.7	gon-C1R1
	***	97.7	96.7	gon-C1R2
		***	98.1	gon-C4R1
			***	gon-C4R2

Similarity: 96.7- 98.6

S. stenotomum-1, stn1

DIRECT SEQUENCING - 200 bp

S. stenotomum-2, stn2

```
Majority   CCTTTTTGTCGAAATTCGGTCGTGTAATAGAAAAAAAATTATTATTTATTTTTTGTAGAAACGACGTCGTTAGGACAGGT
                    10        20        30        40        50        60        70        80
           ---------+---------+---------+---------+---------+---------+---------+---------+
stn2-C1R1  ...................A............................................................
stn2-C1R2  ....................................T...........................................
stn2-C1R3  ...................A............................................................
stn2-C2R1  .T..............................................................................
stn2-C2R2  ................................................................................
stn2-C2R3  ................................................................................
stn2-C2R4  ....................................T..................C.C......................
stn2-C2R5  ..............................G.................................................

Majority   GATGGGGGCGTGGGGAGGATGGGCGTGACGGGCGGCGTCATGCGTCGGTGCGCGTGGAGGCTAGGTCGGTGGGGGGCAGG
                    90        100       110       120       130       140       150       160
           ---------+---------+---------+---------+---------+---------+---------+---------+
stn2-C1R1  ................................................................................
stn2-C1R2  ................................................................................
stn2-C1R3  ...................G............................................................
stn2-C2R1  ................................................................................
stn2-C2R2  ................................................................................
stn2-C2R3  ................................................................................
stn2-C2R4  ...........T.--.......................................................A.........
stn2-C2R5  ..............................................................G.................

Majority   CTAGGGCGTGGGGAGGAAGGAGGTGTTTAATAGAATTTAGAGTGCTATGAATGAT
                    170       180       190       200       210
           ---------+---------+---------+---------+---------+-----
stn2-C1R1  .............................................A.........    215 bp
stn2-C1R2  .............................................A.........    215 bp
stn2-C1R3  ..................A....................................    215 bp
stn2-C2R1  ....A......................C..............G............    215 bp
stn2-C2R2  .........T.............................................    215 bp
stn2-C2R3  .......................................................    215 bp
stn2-C2R4  .........T..................G..........................    213 bp
stn2-C2R5  .............................................A.........    215 bp
```

stn2-C1R1	stn2-C1R2	stn2-C1R3	stn2-C2R1	stn2-C2R2	stn2-C2R3	stn2-C2R4	stn2-C2R5	
***	99.1	98.6	97.2	98.6	99.1	94.9	98.6	stn2-C1R1
	***	97.7	97.2	98.6	99.1	95.8	98.6	stn2-C1R2
		***	96.7	98.1	98.6	94.4	97.2	stn2-C1R3
			***	97.7	98.1	94.0	96.7	stn2-C2R1
				***	99.5	96.3	98.1	stn2-C2R2
					***	95.8	98.6	stn2-C2R3
						***	94.4	stn2-C2R4
							***	stn2-C2R5

Similarity: 94.0-99.5

S. stoloniferum, sto

```
Majority  CCTTTTT-GTCGAAATTCGGTCGTGTAATAGAAAAAATATGATTATTTATTTTTTGTAGAAACGXCGTCGTTAGGACAGG
                  10        20        30        40        50        60        70        80
          ---------+---------+---------+---------+---------+---------+---------+---------+
sto-C2R1  .......-.....................T...G..G...--......---.....C.......G...T......G....
sto-C7R1  .......T.....................T...G..G...--......---.....C.......G...T......G....
sto-C9R1  .......-........................................................G...............
sto-C14R1 .......-........................................................A...............
sto-C18R1 .......-........................................................A...............
sto-C23R1 .......-........................................................A...............

Majority  TGATGGGGGCGT-------------GACGGGCGGCGTCATGCGTCGGTGCGCGTGGAGGCTAGGTCGGTGGGGGGCAGGC
                  90       100       110       120       130       140       150       160
          ---------+---------+---------+---------+---------+---------+---------+---------+
sto-C2R1  C...........TGAGGATGGGCGT.......................................................
sto-C7R1  C...........TGAGGATGGGCGT.......................................................
sto-C9R1  .A..........-------------.......................................................
sto-C14R1 ............-------------............................A..............A...........
sto-C18R1 ............-------------.......................................................
sto-C23R1 ............-------------............................A..........................

Majority  TAGGGCGTTGGGAGGAAGGAGGTGTTTAATAGAATTTAGAGTGCTATGAATGAT
                 170       180       190       200       210
          ---------+---------+---------+---------+---------+----
sto-C2R1  .......G................G.............................    208 bp
sto-C7R1  ......................................................    209 bp
sto-C9R1  ......................................................    200 bp
sto-C14R1 ......................................................    200 bp
sto-C18R1 ...........................C..........................    200 bp
sto-C23R1 ......................................................    200 bp
```

sto-C2R1	sto-C7R1	sto-C9R1	sto-C14R1	sto-C18R1	sto-C23R1	
***	98.6	86.9	86.0	86.4	86.4	sto-C2R1
	***	87.4	86.4	86.9	86.9	sto-C7R1
		***	98.1	98.6	98.6	sto-C9R1
			***	98.6	99.5	sto-C14R1
				***	99.1	sto-C18R1
					***	sto-C23R1

Similarity: 86.0-99.5

S. tarijense, trj

```
Majority  CTTTTTTGTCGAAATTCGGTCGTGTAATTGAAAGAATATATTCATTTATTTTTTGCAGGAACGACGTCGTTAGGACAGGT
                  10        20        30        40        50        60        70        80
          ---------+---------+---------+---------+---------+---------+---------+---------+
trj-C1R1  ...................G............................................................
trj-C1R2  ................................................................................
trj-C1R3  .......................................................T........................
trj-C4R1  T...............................................................................
trj-C7R1  ................................................................................
trj-C7R2  ................................................................................
trj-C8R1  ....................................................................A...........

Majority  GATGGGGGCGTTGAGGATGGGCGTGACGGGGGGCGTCATGCGTCGGTGCGCGTGGAGGCTAGGTCGGCGGGGGGCAGGCT
                  90       100       110       120       130       140       150       160
          ---------+---------+---------+---------+---------+---------+---------+---------+
trj-C1R1  ................G...........................................T...................
trj-C1R2  ................................................................................
trj-C1R3  ................................................................................
trj-C4R1  ...............................................................................C
trj-C7R1  ........................................A......T................................
trj-C7R2  ...........-------------........................................................
trj-C8R1  ................................................................................

Majority  AGGGCGTTGGGAGGAAGGAGGTGTTTAATAGAATTTAGAGTGCTATGAATGAT
                 170       180       190       200       210
          ---------+---------+---------+---------+---------+---
trj-C1R1  ..........................C..........................     213 bp
trj-C1R2  .....................................................     213 bp
trj-C1R3  .........................C...........................     213 bp
trj-C4R1  .....................................................     213 bp
trj-C7R1  .....................................................     213 bp
trj-C7R2  .....................................................     200 bp
trj-C8R1  ........................G............................     213 bp
```

trj-C1R1	trj-C1R2	trj-C1R3	trj-C4R1	trj-C7R1	trj-C7R2	trj-C8R1	
***	98.1	97.2	97.2	97.2	92.5	97.2	trj-C1R1
	***	99.1	99.1	99.1	93.9	99.1	trj-C1R2
		***	98.1	98.1	93.0	98.1	trj-C1R3
			***	98.1	93.0	98.1	trj-C4R1
				***	93.0	98.1	trj-C7R1
					***	93.0	trj-C7R2
						***	trj-C8R1

Similarity: 92.5-99.1

S. torvum, trv

```
Majority       CCTTTTTTGCGGAAATTCCGTCGTCTATTCTATTCGGCGAATCATAATTTTTTTTGGCGGAAACGACGTTTGCTTGGGAC
                        10        20        30        40        50        60        70        80
               ---------+---------+---------+---------+---------+---------+---------+---------+
trv-C2R1       ..........C.....................................................................
trv-C9R1       ................................................................................
trv-C23R1      .............................................T..................................
trv-C40R1      ..........C..................................T..................................
trv-C4R1       ......................................................G.........................

Majority       AGTTGAGGAAGGGCCAGGAACGCGCGCCGTCGCATATGGAGGCTAGGGGCGGTGGTGTGGAGGCTAGGAATGAGGCTTAA
                        90       100       110       120       130       140       150       160
               ---------+---------+---------+---------+---------+---------+---------+---------+
trv-C2R1       ................................................................................
trv-C9R1       ..........................T...............G..A........................A.........
trv-C23R1      .......................................................................A........
trv-C40R1      ................................................................................
trv-C4R1       ...........A..............T...............G..A........................A.........

Majority       TAGAATTAAGAGTGCTAGGGATGAT
                       170       180
               ---------+---------+-----
trv-C2R1       .........................   185 bp
trv-C9R1       .........................   185 bp
trv-C23R1      ................T........   185 bp
trv-C40R1      .........................   185 bp
trv-C4R1       .........................   185 bp
```

trv-C2R1	trv-C9R1	trv-C23R1	trv-C40R1	trv-C4R1	
***	97.3	97.8	99.5	96.2	trv-C2R1
	***	96.2	96.8	98.9	trv-C9R1
		***	98.4	95.1	trv-C23R1
			***	95.7	trv-C40R1
				***	trv-C4R1

Similarity: 95.1-99.5

S. tuberosum ssp. andigena, tbrA1

```
Majority    CCTTTTTGTCGAAATTCGGTCGTGTAATAGAAAAAAXATTATTATTTATTTTTTGTAGAAACGACGTCGTTAGGACAGGT
                     10        20        30        40        50        60        70        80
            ---------+---------+---------+---------+---------+---------+---------+---------+
tbrA-C1R1   ....................................A...........................................
tbrA-C2R1   ....................................T...........................................
tbrA-C4R1   ...................A................A...........................................
tbrA-C8R1   ....................................C.G.........................................
tbrA-C10R1  ................T...........T.......T..................C..G.....................

Majority    GATGGGGGCGTGGGGAGGATGGGCGTGACGGGCGGCGTCATGCGTCGGTGCGCGTGGAGGCTAGGTCGGTGGGGGGCAGG
                     90       100       110       120       130       140       150       160
            ---------+---------+---------+---------+---------+---------+---------+---------+
tbrA-C1R1   ................................................................................
tbrA-C2R1   ................................................................................
tbrA-C4R1   ..................................................................T.............
tbrA-C8R1   ...........T.--.................................................................
tbrA-C10R1  ...........T.--.................A...............................................

Majority    CTAGGGCGTTGGGAGGAAGGAGGTGTTTAATAGAATTTAGAGTGCTATGAATGAT
                    170       180       190       200       210
            ---------+---------+---------+---------+---------+-----
tbrA-C1R1   .........G.............................................   215 bp
tbrA-C2R1   .........G...................................A.........   215 bp
tbrA-C4R1   ..................................................G....   215 bp
tbrA-C8R1   .....................A............................G....   213 bp
tbrA-C10R1  .....................A........................T........   213 bp
```

tbrA-C1R1	tbrA-C2R1	tbrA-C4R1	tbrA-C8R1	tbrA-C10R1	
***	99.1	98.1	96.3	94.4	tbrA-C1R1
	***	97.2	95.8	94.4	tbrA-C2R1
		***	96.3	93.5	tbrA-C4R1
			***	95.8	tbrA-C8R1
				***	tbrA-C10R1

Similarity: 93.5-99.1

S. tuberosum ssp. andigena, tbrA2

```
Majority  CCTTTTTGTCGAAATTCGGTCGTGTAAXXXXXXXXATATAXTTATTTATTTTTTGXAGAAACGXCGTCGTTAGGACXXXX
                   10        20        30        40        50        60        70        80
          ---------+---------+---------+---------+---------+---------+---------+---------+
tbrT-C1R1 ...........................TAGAAAAA....TA.............T.......A.............TAAC
tbrT-C7R1 ...........................TAGAAAAA....CA.............T.......A.............TAAC
tbrT-C2R1 ...........................--------.....T.............C.......G.............----
tbrT-C5R1 ...........................--------.....T.............C.......G.............----

Majority  AGGXGATGGGGGCGTTGXXAGGATGGGCGTGACGGGCGGCGTCATGCGTCGGTGCGCGTGGAGGCTAGGTCGGTGGGGGG
                   90       100       110       120       130       140       150       160
          ---------+---------+---------+---------+---------+---------+---------+---------+
tbrT-C1R1 ...T.............GG.............................................................
tbrT-C7R1 ...T.............GG.............................................................
tbrT-C2R1 ...C.............--..............................................G..............
tbrT-C5R1 ...C.............--.............................................................

Majority  CAGGCTAGGGCGTTGGGAGGAAGGAGGTGTTTAATAGAATTTAGAGTGCTATGAATGAT
                  170       180       190       200       210
          ---------+---------+---------+---------+---------+---------
tbrT-C1R1 ...........................................................    219 bp
tbrT-C7R1 ................................G..........................    219 bp
tbrT-C2R1 ...........................................................    205 bp
tbrT-C5R1 ...........................................................    205 bp
```

tbrT-C1R1	tbrT-C7R1	tbrT-C2R1	tbrT-C5R1	
***	99.1	90.9	91.3	tbrT-C1R1
	***	90.4	90.9	tbrT-C7R1
		***	99.5	tbrT-C2R1
			***	tbrT-C5R1

Similarity: 90.4-99.5

S. tuberosum-1, tbr1

```
Majority  CCTTTTTGTCGAAATTCGGTCGTGTAAATATATTTATTTATTTTTTGCAGAAACGGCGTCGTTAGGACAGGCGATGGGGG
                  10        20        30        40        50        60        70        80
          ---------+---------+---------+---------+---------+---------+---------+---------+
pStB15.1  ................................................................................
pStB15.2  .............................................G..................................
pStB15.3  ................................................................................

Majority  CGTTGAGGATGGGCGTGACGGGCGGCGTCATGCGTCGGTGCGCGTGGAGGCTAGGTCGGTGGGGGGCAGGCTAGGGCGTT
                  90       100       110       120       130       140       150       160
          ---------+---------+---------+---------+---------+---------+---------+---------+
pStB15.1  ................................................................................
pStB15.2  ................................................................................
pStB15.3  ................................................................................

Majority  GGGAGGAAGGAGGTGTTTAATAGAATTTAGAGTGCTATGAATGAT
                 170       180       190       200
          ---------+---------+---------+---------+-----
pStB15.1  .............................................  205 bp
pStB15.2  .............................................  205 bp
pStB15.3  .............................................  205 bp
```

pStB15.1	pStB15.2	pStB15.3	
***	99.5	100.0	pStB15.1
	***	99.5	pStB15.2
		***	pStB15.3

Similarity: 99.5-100.0

S. tuberosum-2, tbr2

ONE CLONE - 216 bp

S. tuberosum-3, tbr3

```
Majority        CCTTTTTGTCGAAATTCGGTCGTGTAAATATATTTATTTATTTTTTGCAGAAACGGCGTCGTTAGGACAGGCGATGGGGG
                         10        20        30        40        50        60        70        80
                ---------+---------+---------+---------+---------+---------+---------+---------+
pStbr3-B1076_5  ................................................................................
pStbr3-B1076_2  ................................................................................
pStbr3-B1076_1  ................................................................................
pStbr3-B1076_3  ................................................................................

Majority        CGTTGAGGATGGGCGTGACGGGCGGCGTCATGCGTCGGTGCGCGTGGAGGCTAGGTCGGTGGGGGGCAGGCTAGGGCGTT
                         90        100       110       120       130       140       150       160
                ---------+---------+---------+---------+---------+---------+---------+---------+
pStbr3-B1076_5  ................................................................................
pStbr3-B1076_2  ................................................................................
pStbr3-B1076_1  ................................................................................
pStbr3-B1076_3  ...........................C....................................................

Majority        GGGAGGAAGGAGGTGTTTAATAGAATTTAGAGTGCTATGAATGAT
                         170       180       190       200
                ---------+---------+---------+---------+-----
pStbr3-B1076_5  .............................................      205 bp
pStbr3-B1076_2  .............................................      205 bp
pStbr3-B1076_1  .............................................      205 bp
pStbr3-B1076_3  .............................................      205 bp
```

tbr3-B1076_5	tbr3-B1076_2	tbr3-B1076_1	tbr3-B1076_3	
***	100.0	100.0	99.5	tbr3-B1076_5
	***	100.0	99.5	tbr3-B1076_2
		***	99.5	tbr3-B1076_1
			***	tbr3-B1076_3

Similarity: 99.5-100

S. tuberosum-4, tbr4

```
Majority        CCTTTTTGTCGAAATTCGGTCGTGTAATAGAAAAAATATTATTATTTATTTTTTGCAGAAACGACGTCGTTAGGACAGGT
                        10        20        30        40        50        60        70        80
                ---------+---------+---------+---------+---------+---------+---------+---------+
pStbr4-B1.1     .T......................................T.....................C.T...C.A.........
pStbr4-B1.2     ..............................................................................T.
pStbr4-B1.4     .................C..........................................-...........C.......
pStbr4-B1.5     .......................................G........................................

Majority        GATGGGGGCGTTGAGGATGGGCGTGACGGGCGGCGTCATGCGTCGGTGCGCGTGGAGGCTAGGTCGGTGGGGGGCAGGCT
                        90       100       110       120       130       140       150       160
                ---------+---------+---------+---------+---------+---------+---------+---------+
pStbr4-B1.1     ....A......................T....A...............................................
pStbr4-B1.2     .............T.......A..........................................................
pStbr4-B1.4     ..........................................................................G.....
pStbr4-B1.5     ................................................................................

Majority        AGGGCGTTGGGAGGAAGGAGGTGTTTAATAGAATTTAGAGTGCTATGAATGAT
                       170       180       190       200       210
                ---------+---------+---------+---------+---------+---
pStbr4-B1.1     ..........................G...............G..........    213 bp
pStbr4-B1.2     .....................................................    213 bp
pStbr4-B1.4     .....................C..C............................    212 bp
pStbr4-B1.5     .....................................................    213 bp
```

tbr4-B1.1	tbr4-B1.2	tbr4-B1.4	tbr4-B1.5	
***	93.4	92.0	94.4	tbr4-B1.1
	***	95.8	98.1	tbr4-B1.2
		***	96.7	tbr4-B1.4
			***	tbr4-B1.5

Similarity: 92.0-98.1

S. valdiviense, val

```
Majority  CCTTTTTGTCGAAATTCGGCGTAACTTTGATAATATATATATATTTTTGCAGAAAGGACATTCGGGCCGAGACGTCGT
                   10        20        30        40        50        60        70        80
          ---------+---------+---------+---------+---------+---------+---------+---------+
val-C2R1  ................................................................................
val-C7R1  T...............................................................................
val-C11R1 ................................................................................

Majority  TAGGACAGGTGATGGAGGCGCTTAGGTTGGGCGTGACAGGCGGCGGTGCGTGGAGGCTAGCGCGGCGGGGGACAGGCTAG
                   90       100       110       120       130       140       150       160
          ---------+---------+---------+---------+---------+---------+---------+---------+
val-C2R1  ................................................................................
val-C7R1  ................................................................................
val-C11R1 .................................G..............................................

Majority  GGCGTTGGGGGGAAGGAGTCGTTTAATAGAATTTAGAGTGGTAGGAATGAC
                  170       180       190       200       210
          ---------+---------+---------+---------+---------+-
val-C2R1  ...................................................    211 bp
val-C7R1  ........T..........................................    211 bp
val-C11R1 ...................................................    211 bp
```

val-C2R1	val-C7R1	val-C11R1	
***	99.1	99.5	val-C2R1
	***	98.6	val-C7R1
		***	val-C11R1

Similarity: 98.6-99.5

S. venturii, vnt

```
Majority  CCTTTTTGTCGAAATTCGGTCGTGTAATTGAAGAAATAAAATATATTAATATTTATTTTTTGCAGAAAGGGCGTCGTTAG
                  10        20        30        40        50        60        70        80
          ---------+---------+---------+---------+---------+---------+---------+---------+
vnt-C1R1  ................................................................................
vnt-C1R2  ..................................................................G.............
vnt-C2R1  ................................................................................
vnt-C2R2  ...........................................G....................................
vnt-C5R1  ..................................................G.............................
vnt-C5R3  ................................................................................
vnt-C2R3  ................................--------........................................
vnt-C5R2  ................................--------..........................G.............


Majority  GACAGGCGATGGGGGCGTTGAGGATGGGCGTGACGGGCGGCGTCATGCGTCGGTGCGCGTGGAGGATAGGTCGGTGGGGG
                  90       100       110       120       130       140       150       160
          ---------+---------+---------+---------+---------+---------+---------+---------+
vnt-C1R1  .......T.........................................................C..............
vnt-C1R2  ................................................................................
vnt-C2R1  ................................................................................
vnt-C2R2  .................................................................C..........T...
vnt-C5R1  ................................................................................
vnt-C5R3  ................................................................................
vnt-C2R3  ...............T..................T.............................................
vnt-C5R2  .................................................................C..............

Majority  GCAGGCTAGGGCGTTGGGAGGAAGGAGGTGTTTAATAGAATTTAGAGTGCTATGAATGAT
                 170       180       190       200       210       220
          ---------+---------+---------+---------+---------+---------+
vnt-C1R1  ..G...................G.....................................     220 bp
vnt-C1R2  ............................................................     220 bp
vnt-C2R1  ............................................................     220 bp
vnt-C2R2  ............................................................     220 bp
vnt-C5R1  .............................TC...................A.........     220 bp
vnt-C5R3  ..............................C.............................     220 bp
vnt-C2R3  ............................................................     212 bp
vnt-C5R2  ............................................................     212 bp
```

vnt-C1R1	vnt-C1R2	vnt-C2R1	vnt-C2R2	vnt-C5R1	vnt-C5R3	vnt-C2R3	vnt-C5R2	
***	97.7	98.2	97.7	96.4	97.7	93.6	94.5	vnt-C1R1
	***	99.5	98.2	97.7	99.1	95.0	95.9	vnt-C1R2
		***	98.6	98.2	99.5	95.5	95.5	vnt-C2R1
			***	96.8	98.2	94.1	95.0	vnt-C2R2
				***	98.6	93.6	93.6	vnt-C5R1
					***	95.0	95.0	vnt-C5R3
						***	98.2	vnt-C2R3
							***	vnt-C5R2

Similarity: 93.6-99.5

S. vernei-1, vrn1

DIRECT SEQUENCING - 202 bp

S. vernei-2, vrn2

```
Majority   CCTTTTTGTCGAAATTCGGTCGTGTAATTGAAAAAATATATTTATTTATTTACTGCXXXXXXXXXXXXXXXXGGACXGGT
                   10        20        30        40        50        60        70        80
           ---------+---------+---------+---------+---------+---------+---------+---------+
vrn2-C1R2  ......G................C................................AGAAATTCGGTCGTTA....A...
vrn2-C1R3  ........................................................AGAAATTCGGTCGTTA....A...
vrn2-C1R4  ........................................................----------------....G...
vrn2-C1R1  .........................C..............................----------------....G...

Majority   GATGGGGGCGTTGGTGATGGGCGTGACGGGCGGCGTCATGXGTCGGTGCGCGCGGAGGCTAGGACGGTGGGGGGCAGGCT
                   90       100       110       120       130       140       150       160
           ---------+---------+---------+---------+---------+---------+---------+---------+
vrn2-C1R2  ........................................C...........T.....A.............A.......
vrn2-C1R3  ......---------------...................C.......................................
vrn2-C1R4  ........................................G.......................................
vrn2-C1R1  ........................................G..........................G............

Majority   AGGGCGTTGGGAGGAAGGAGGTGTTTAATAGAATTTAGAGCGCAATGAATGAT
                  170       180       190       200       210
           ---------+---------+---------+---------+---------+---
vrn2-C1R2  ..........................G..........................    213 bp
vrn2-C1R3  .....................................................    198 bp
vrn2-C1R4  .....................................................    197 bp
vrn2-C1R1  ....................................................C    197 bp
```

vrn2-C1R2	vrn2-C1R3	vrn2-C1R4	vrn2-C1R1	
***	90.1	88.7	87.3	vrn2-C1R2
	***	84.5	83.1	vrn2-C1R3
		***	98.6	vrn2-C1R4
			***	vrn2-C1R1

Similarity: 83.1-98.6

S. vernei-3, vrn3

```
Majority    CCTTTTTGTCGAAATTCGGTCGTGTAATTGATTTTAATATATTTATTTATTTACTGCA----TT--ATCAT-AGGACAGG
                    10        20        30        40        50        60        70        80
            ---------+---------+---------+---------+---------+---------+---------+---------+
vrn3-C1R5   .T........................................................GAAA..CGG..G.T........
vrn3-C1R1   ...................G...........AAA-G......................GAAA..CGG..G.T........
vrn3-C1R2   ...............................AAA-G.........G............GAAA..CGG..G.T........
vrn3-C1R3   .............G............G........G..G..CG...............----..--.....-........
vrn3-C1R4   ..........................................................----..--.....-...G....
vrn3-C1R6   ..........................................G...............----..--.....-........
vrn3-C1R7   ......................G...................................----..--.....-........
vrn3-C1R8   ..........................................................----..--.....-........
vrn3-C1R9   ...................C......................................----..--.....-........

Majority    TGATGGGGGCGTTGGTGATGGGCGTGACGGGCGGCGTCATGCGTCGGTGCGCGCGGAGGCTAGGACGGTGGGGGGCAGGC
                    90        100       110       120       130       140       150       160
            ---------+---------+---------+---------+---------+---------+---------+---------+
vrn3-C1R5   ................................................................................
vrn3-C1R1   .......------------------------T................................................
vrn3-C1R2   .......------------------------...........................................T.....
vrn3-C1R3   ...............................................G.TCG.......G.C..G...G......G....
vrn3-C1R4   ..............................................................T...A.G....C..C...
vrn3-C1R6   ...........G...............................G....................................
vrn3-C1R7   ...........................................................A....................
vrn3-C1R8   ................................................................................
vrn3-C1R9   ................................................................................

Majority    TAGGGCGTTGGGAGGAAGGAGGTGTTTGATAGAATTTAGAGCGCAATGAATGAT
                    170       180       190       200       210
            ---------+---------+---------+---------+---------+----
vrn3-C1R5   ...................................G..................     214 bp
vrn3-C1R1   ...........................A..........................     189 bp
vrn3-C1R2   .............................................C........     189 bp
vrn3-C1R3   G..C...G....C..CG..G....A.........................G...     207 bp
vrn3-C1R4   G.T.T.....C....C...C..G..G..C..C....C........G........     207 bp
vrn3-C1R6   ..............................................C.......     207 bp
vrn3-C1R7   ......................................................     207 bp
vrn3-C1R8   ......................................................     207 bp
vrn3-C1R9   ..........................C...........................     207 bp
```

vrn3-C1R5	vrn3-C1R1	vrn3-C1R2	vrn3-C1R3	vrn3-C1R4	vrn3-C1R6	vrn3-C1R7	vrn3-C1R8	vrn3-C1R9	
***	84.1	84.1	83.2	86.0	92.5	93.5	94.4	93.5	vrn3-C1R5
	***	97.2	70.1	72.0	79.0	79.4	80.4	79.9	vrn3-C1R1
		***	70.1	72.4	79.0	79.4	80.4	79.4	vrn3-C1R2
			***	83.6	87.9	88.3	88.8	87.9	vrn3-C1R3
				***	89.7	90.7	91.6	90.7	vrn3-C1R4
					***	97.2	98.1	97.2	vrn3-C1R6
						***	99.1	98.1	vrn3-C1R7
							***	99.1	vrn3-C1R8
								***	vrn3-C1R9

Similarity: 70.1-99.1

S. verrucosum, ver

```
Majority   CCTTTTTGTCGAAATTTGGTCGTGTAATTGAAAAAATATTATTATTTGTTTTTTGCATGAACGACGTCGTTAGGACAGGT
                   10        20        30        40        50        60        70        80
           ---------+---------+---------+---------+---------+---------+---------+---------+
ver-C2R1   .................................T..............G...............................
ver-C2R2   .-.............................................A.........G......................
ver-C6R1   .........................................C......................................
ver-C6R2   .........................................C......................................
ver-C13R1  .......-......................................................................A.
ver-C13R2  ................................................G...............................
ver-C13R3  .................................T..............G.A......G......................

Majority   GATGGGGGCGTTGAGGATGGGCGTGACGGGCGGCGTCATGCGTCGGTGCGCGTGGAGGCTAGGTCGGTGGGGGGCAGGCT
                   90       100       110       120       130       140       150       160
           ---------+---------+---------+---------+---------+---------+---------+---------+
ver-C2R1   ....................C...........................................................
ver-C2R2   .....T..........................A...............................................
ver-C6R1   ................................................................................
ver-C6R2   ..............................................G.................................
ver-C13R1  .............................C..................................................
ver-C13R2  ................................................................................
ver-C13R3  ....................C...............................................A...........

Majority   AGGGCGTTGGGAGGAAGGAGGTGTTTAATAGAATTTAGAGTGCTATGAATGAT
                  170       180       190       200       210
           ---------+---------+---------+---------+---------+---
ver-C2R1   ................T.........G..........................    213 bp
ver-C2R2   ........................C............................    213 bp
ver-C6R1   .........A...........................................    213 bp
ver-C6R2   .....................................................    213 bp
ver-C13R1  ................T....................................    212 bp
ver-C13R2  ...........................G.........TT..............    213 bp
ver-C13R3  ...........................G.........TT..............    213 bp
```

ver-C2R1	ver-C2R2	ver-C6R1	ver-C6R2	ver-C13R1	ver-C13R2	ver-C13R3	
***	94.8	96.7	96.7	96.7	96.7	96.2	ver-C2R1
	***	96.2	96.2	95.3	95.3	93.9	ver-C2R2
		***	99.1	97.2	97.2	94.8	ver-C6R1
			***	97.2	97.2	94.8	ver-C6R2
				***	96.2	93.9	ver-C13R1
					***	97.7	ver-C13R2
						***	ver-C13R3

Similarity: 93.9-99.1

S. vespertilio, ves

```
Majority      TTTTTTTT-GCCAAAATTTCGTCGTCTATTCGGCGAATGATTATTTTTTTTXGCGGTTGGGTCGTTTGCTTGGGGGGAGA
                      10        20        30        40        50        60        70        80
              ---------+---------+---------+---------+---------+---------+---------+---------+
pSves-8(1)    ....G...-.............G.........A.......A..........T............................
pSves-8(2)    ........-.........C................................G...............T............
pSves-10      ........T.A.G......................................-.................A..........

Majority      CGTCGTTAGGACGGTTGAAGAAGGGCGTCGCGGTAGCAAAAAGGCCTATGGCGGGCGGTGGTGTGCATGGCTAGGGCGAG
                      90       100       110       120       130       140       150       160
              ---------+---------+---------+---------+---------+---------+---------+---------+
pSves-8(1)    ........T...................................................................T...
pSves-8(2)    ..........................................................A.....................
pSves-10      ................................................................................

Majority      GGGGGAGGAATGAGGTCTAATAGAATTAAGAGTGCTAGGAATGAT
                     170       180       190       200
              ---------+---------+---------+---------+-----
pSves-8(1)    ..C..........................................      204 bp
pSves-8(2)    ....................................T....C...      204 bp
pSves-10      ....A...........G....................A......A      204 bp
```

pSves-8(1)	pSves-8(2)	pSves-10	
***	93.7	92.2	pSves-8(1)
	***	93.2	pSves-8(2)
		***	pSves-10

Similarity: 92.2-93.7

S. villosum, vil

```
Majority CCTTTTTGTCGAAGTTCGGCATXATTTCGTCTATTTGATAATATATATTATTATTATTTTTGXAGAAACGXCATTCGTGC
                  10        20        30        40        50        60        70        80
         ---------+---------+---------+---------+---------+---------+---------+---------+
pSvil-7  ......................A.......................................T.......A.........
pSvil-8  ......................G.......................................C.......G.........

Majority CTAGACGTCGCTAGGACGGGTGACGGAGCGCTGAGGACGGGCGTGACAGGCATGCCGTCGGTGCGTGGAGGCTAGGGCGG
                  90       100       110       120       130       140       150       160
         ---------+---------+---------+---------+---------+---------+---------+---------+
pSvil-7  ................................................................................
pSvil-8  ................................................................................

Majority CGTGGXACATGCTATGGCGTTGGGAGGAAGGAXGTGTTTAATAXAATTTAGAGCGCAATGAATGAX
                 170       180       190       200       210       220
         ---------+---------+---------+---------+---------+---------+------
pSvil-7  .....A..........................G..........G.....................C     226 bp
pSvil-8  .....G..........................C..........T.....................T     226 bp
```

pSvil-7	pSvil-8	
***	96.9	pSvil-7
	***	pSvil-8

Similarity: 96.9

S. violaceimarmoratum, vio

```
Majority  CCTTTTTGTCGAAATTCGGTCGTGTAATTGAAAAAAAATATATXTATTTATTTTTTGCAGAAACGACGTCGTTAGGACAG
                  10        20        30        40        50        60        70        80
          ---------+---------+---------+---------+---------+---------+---------+---------+
vio-C1R1  ............C......C........C...C..........A...............T..G.................
vio-C1R2  ...........................................A....................................
vio-C1R3  .........................................C.T..................T.....G...........
vio-C3R1  ...........................................T.............A......................

Majority  GCGATGGGGGCGTTGAGGAAGGGCGTGACGGGCGTCGGTGCGTGGAGGCTAGGTCGGTGGGGGGCAGGCTAGGGCGTTGG
                  90       100       110       120       130       140       150       160
          ---------+---------+---------+---------+---------+---------+---------+---------+
vio-C1R1  .T.................T...........................................AA.........A.....
vio-C1R2  ................................................................................
vio-C1R3  .............G.T..GG............................................................
vio-C3R1  ................................................................................

Majority  GATGAAGGAGGTGTTTAATAGAATTTAGAGTGGTATGAATGAT
                 170       180       190       200
          ---------+---------+---------+---------+---
vio-C1R1  .......C...................................      203 bp
vio-C1R2  ...............C...........................      203 bp
vio-C1R3  ...........................................      203 bp
vio-C3R1  ...........................................      203 bp
```

vio-C1R1	vio-C1R2	vio-C1R3	vio-C3R1	
***	93.6	91.1	93.1	vio-C1R1
	***	95.6	98.5	vio-C1R2
		***	96.1	vio-C1R3
			***	vio-C3R1

Similarity: 91.1-98.5

S. wendlandii, wen

```
Majority     CCTTTTTGTCGAAATTCGGAATAATTTTGT-CTATTCGATGGTA-TTTTTTGGCAGAAATGACATTTGGGCGGAGACGTC
                     10        20        30        40        50        60        70        80
             ---------+---------+---------+---------+---------+---------+---------+---------+
pSowen-3.1   ......C..............................-.............-...................................
pSowen-3.2   ..................C.......C..-.........A.A.G......C.............................
pSowen-13.1  ............................T.T.............-.........................A.........T
pSowen-13.2  .................A.............-.............-........T..........................
pSowen-13.3  ......G...A...................-.....T.......-........A..........................
pSowen-14    .T.........C..................-.............-...............................A..
pSowen-18.1  .T...............A.............-.............-...................................
pSowen-18.2  .................A.C.......CA.-T......G.A.A.G......T.......CT.......A...........

Majority     GTTAGGACAGGCGCTGAGGATGGGCGTGTCGGGCGTCACCACGTGCCGGTGCX---------------------------
                     90       100       110       120       130       140       150       160
             ---------+---------+---------+---------+---------+---------+---------+---------+
pSowen-3.1   A.......................................................A---------------------------
pSowen-3.2   ..........T.A.....C.............A.A...A....C...A....GTGTCGGGCGTCACCACACTCCGGTGCG
pSowen-13.1  .........................................T........A.T---------------------------
pSowen-13.2  ..............................T...........AC....A...G---------------------------
pSowen-13.3  ...........TA.......................................A---------------------------
pSowen-14    ........................T................T..A.....A.G---------------------------
pSowen-18.1  ...........................T........................A---------------------------
pSowen-18.2  A......A..T.TT.A.......A...................CA.T..-------------------------------

Majority     ------------------------------------------------------TGGGGC-TAGGXTGGTGGGGTGCAGG
                    170       180       190       200       210       220       230       240
             ---------+---------+---------+---------+---------+---------+---------+---------+
pSowen-3.1   ------------------------------------------------------......-....C....T.........
pSowen-3.2   TGTCGGGCGTCACCACGCGCCGGTGCGTGTCGTGCGTCACCATGCGTCAGTGCG.....GA..T.GC...........T.
pSowen-13.1  ------------------------------------------------------....T.-....G.T............
pSowen-13.2  ------------------------------------------------------.....GA.T..GC......T.T....
pSowen-13.3  ------------------------------------------------------......-....C....T.........
pSowen-14    ------------------------------------------------------......-....G..............
pSowen-18.1  ------------------------------------------------------......-G...C....T........A
pSowen-18.2  ----------------GC--CGGAG-------GG---------------------....GC..T.CC.............

Majority    CTAGGGCATTGGGAGGAACGAGGTTAAAGG-AATTTAGAGTGATAGGAATGAT
                    250       260       270       280       290
             ---------+---------+---------+---------+---------+---
pSowen-3.1   ..............................-..............A....T..     208bp
pSowen-3.2   ..........C....A..T......TG.TAG......T....C...A......     292bp
pSowen-13.1  T.............................-.................T....     209bp
pSowen-13.2  .......G..........T.C....T..TAC...........C..........     210bp
pSowen-13.3  ..............................-..............AT......     208bp
pSowen-14    T.............................-......................     208bp
pSowen-18.1  ..............................-......................     208bp
pSowen-18.2  ..T..T.G..CA......T......T..TAG....G......C.C.A.....A     215bp
```

pSwen-3.1	pSwen-3.2	pSwen-13.1	pSwen-13.2	pSwen-13.3	pSwen-14	pSwen-18.1	pSwen-18.2	
***	59.7	94.2	90.8	96.9	94.9	96.9	79.9	pSwen-3.1
	***	58.4	61.8	59.4	59.4	59.4	61.4	pSwen-3.2
		***	89.4	92.8	95.6	93.9	77.8	pSwen-13.1
			***	89.8	90.4	91.1	81.2	pSwen-13.2
				***	93.5	95.6	78.5	pSwen-13.3
					***	95.2	78.8	pSwen-14
						***	79.5	pSwen-18.1
							***	pSwen-18.2

Similarity: 58.4-96.9

S. wrightii, wri

ONE RIBOTYPE -183 bp
